# Blood cell traits and risk of glaucoma: A two-sample mendelian randomization study

**DOI:** 10.3389/fgene.2023.1142773

**Published:** 2023-04-12

**Authors:** De-Juan Song, Bin Fan, Guang-Yu Li

**Affiliations:** Department of Ophthalmology, The Second Hospital of Jilin University, Changchun, China

**Keywords:** blood cell traits, glaucoma, basophil cell count, lymphocyte cell count, platelet count, plateletcrit, mendelian randomization

## Abstract

**Importance:** Glaucoma is the second leading cause of blindness in the world. The causal direction and magnitude of the association between blood cell traits and glaucoma is uncertain because of the susceptibility of observational studies to confounding and reverse causation.

**Objective:** To explore whether there is a causal relationship of blood cell traits including white blood cell (WBC) count (WBCC) and its subtypes [basophil cell count (BASO), monocyte cell count (MONO), lymphocyte cell count (LYMPH), eosinophil cell count (EOS), neutrophil cell count (NEUT)], red blood cell (RBC) count (RBCC), red blood distribution width (RDW), platelet count (PLT), and plateletcrit (PCT) on glaucoma risk.

**Methods:** A two-sample Mendelian randomization (MR) analysis was conducted. Genome-wide significant single nucleotide polymorphisms (SNPs) from published genome-wide association studies (GWAS) on human blood cell traits were utilized as exposure instruments and the dataset for outcome was from the GWAS summary data of glaucoma. In the univariable MR analysis, we examined the association between genetic evidence of blood cell traits and glaucoma. To further investigate the potential causal mechanisms underlying the observed association, we performed multivariable MR analysis with three models, taking into account the mediator effect of inflammation and oxidative stress. According to Bonferroni-corrected for the 10 exposures in 3 methods, the MR study yielded a statistically significant *p*-value of 0.0017.

**Results:** Genetically BASO, PCT, LYMPH, and PLT were potentially positively associated with glaucoma in the European ancestry [BASO: Odds ratio (OR) = 1.00122, 95% confidence interval (CI), 1.00003–1.00242, *p* = 0.045; PCT: OR = 1.00078, 95% CI, 1.00012–1.00143, *p* = 0.019; LYMPH: OR = 1.00076, 95% CI, 1.00002–1.00151, *p* = 0.045; PLT: OR = 1.00065, 95% CI, 1.00006–1.00123, *p* = 0.030], There was insufficient evidence to support a causal association of MONO, NEUT, EOS, WBCC, RBCC and RDW (MONO: OR = 1.00050, *p* = 0.098; NEUT: OR = 1.00028, *p* = 0.524; EOS: OR = 1.00020, *p* = 0.562; WBCC: OR = 1.00008, *p* = 0.830; RBCC: OR = 0.99996, *p* = 0.920; RDW: OR = 0.99987, *p* = 0.734) with glaucoma. The multivariable MR with model 1, 2, and 3 demonstrated that BASO, PCT, LYMPH, and PLT were still potentially genetically associated with the risk of glaucoma.

**Conclusion:** Our study reveals a genetic predisposition to higher LYMPH, BASO, PLT, and PCT are associated with a higher risk of glaucoma, whereas WBCC, MONO, EOS, NEUT, RBCC, and RDW are not associated with the occurrence of glaucoma. This finding also supports previous observational studies associating immune components with glaucoma, thus provide guidance on the predication and prevention for glaucoma.

## Introduction

Glaucoma is a chronic condition of progressive optic neuropathy characterized by degeneration of retinal ganglion cells, which leads to loss of visual field and irreversible blindness (Kountouras et al., 2004). The worldwide prevalence has been estimated to 111.8 million people by 2040, making glaucoma the leading cause of irreversible vision loss in the world (Tham et al., 2014). Glaucoma consists of open-angle glaucoma and angle-closure glaucoma. Reducing intraocular pressure (IOP) is the only proven method to treat glaucoma through medical treatment and surgeries (Jonas et al., 2017). Although substantial IOP reductions can be achieved in the majority of patients, the effect decreases gradually over time with a failure rate of about 10% per year. Moreover, some cases could be asymptomatic until a relatively late stage (Weinreb et al., 2014). Therefore, more public health efforts are required to focus on glaucoma prevention. Evidence that identifies the causal risk factors for glaucoma is needed urgently for better prevention.

Circulating blood cells, including RBCs, WBCs, and platelets, could be counted and sized easily, which has been one of the most common laboratory tests in medicine (Tefferi et al., 2005). Abnormalities in them usually gives important clues for the diagnosis of different diseases (Tefferi et al., 2005). Moreover, the relationships between blood cell components with glaucoma have been reported in some studies. For example, several clinical observational studies have investigated the relationships between some immune components [WBC, neutrophil, and neutrophil-to-lymphocyte ratio (NLR) (Li et al., 2017a); subsets of lymphocytes, levels of cytokine IL-2, and the soluble IL-2 receptor (Yang et al., 2001)]; platelet parameters [PLT, PCT, platelet distribution width, mean platelet volume, platelet large cell ratio, and coagulation function) (Ma et al., 2019)] with glaucoma. Despite numerous observational studies investigating the potential risk factors for glaucoma, the causal relationship between these risk factors and the development of glaucoma remains unclear. The limited number of glaucoma patients and inconsistent results in these studies make it difficult to draw definitive conclusions about the causal nature of these risk factors. To address the potential confounding and reverse causality issues that are inherent in observational studies, some randomized clinical trials have been conducted to evaluate the effects of interventions on glaucoma progression. For example, several randomized clinical trials have demonstrated that topical prostaglandin analog can reduce visual field deterioration in open-angle glaucoma patients (Garway-Heath et al., 2013; Founti et al., 2020) However, randomized clinical trials are often prohibitively expensive, time consuming, and may not be practical for diseases like glaucoma, which has substantial lag time between exposure to risk factors and clinical manifestation of the disease.

MR is a genetic epidemiological approach to assess the causality between exposures (in most cases risk factors) and outcomes (diseases) by using genetic variants as instrumental variables (IVs) (Bowden and Holmes, 2019). By resembling a naturally designed randomized clinical trial, the MR approach is a valuable tool to identify risk factors and causal associations with less confounders than classical observational epidemiological studies (Emdin et al., 2017). Besides, MR studies avoids reverse causality because of the genetic variant used as IVs were generated earlier than most of outcomes happening (Hingorani and Humphries, 2005). Recent MR studies have demonstrated that type 2 diabetes (Hu et al., 2022), myopic refractive error (Choquet et al., 2022), coffee consumption (Li et al., 2022), and blood lipid-related metabolites (Nusinovici et al., 2022) have potential causal associations with glaucoma.

No previous MR studies have explored the causal relationship between blood cell traits and glaucoma. Therefore, in this study, we use MR to evaluate the potential relationship between blood cell traits and glaucoma. Multivariable MR analysis were also carried out to detect potential confounders of inflammatory and oxidative stress.

## Materials and methods

### Study design

**FIGURE 1:**
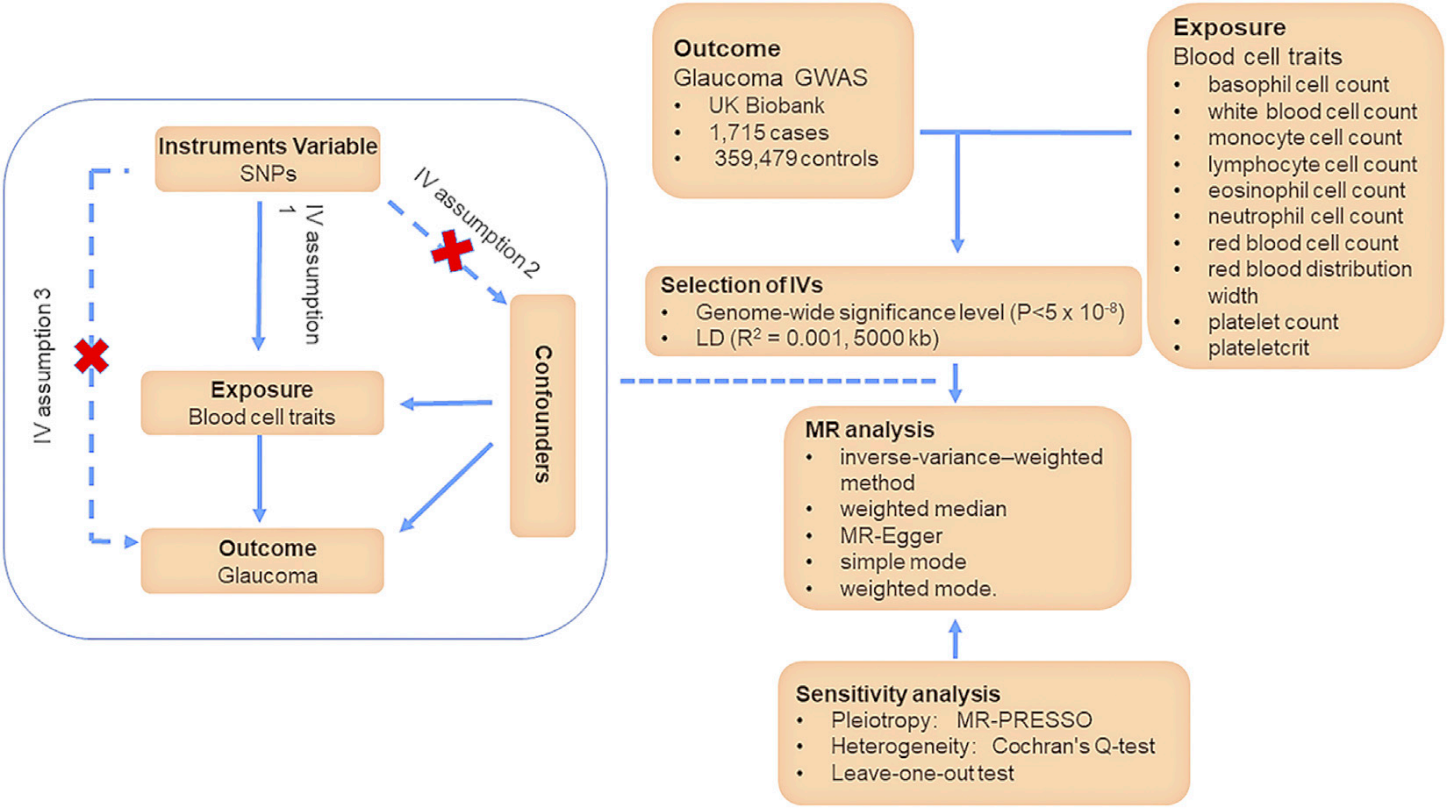
Acyclic graph interpretation of Mendelian randomization analysis.

Two-sample MR allows the use of publicly available summary-level data from multiple sources to infer causal relationships between various blood cell traits and glaucoma by using SNPs as IVs to avoid accidental influence (Davey Smith and Hemani, 2014). Our MR study is based on three assumptions: 1) The IVs are robustly associated with blood cell traits; 2) The IVs are independent of any confounders between blood cell traits and glaucoma; 3) the IVs affect glaucoma exclusively *via* their effects on blood cell traits, rather than through any direct pathways (Figure 1) (Lee and Lim, 2019). To investigate the causal effects of multiple risk factors jointly, we employed a multivariable MR approach, which is an extension of univariable MR (Sanderson, 2021). The total effect of blood cell traits on glaucoma can be decomposed into direct and indirect effects (Burgess et al., 2015). To specifically examine the direct effects of blood cell traits, we conducted a two-step MR analysis (Relton and Davey Smith, 2012). Firstly, we estimated the casual effect of blood cell traits on glaucoma. Next, we filtered positive results of blood cell traits. Finally, we carried out a multivariable MR with three models to determine the direct effects of positive results on glaucoma, after adjusting for inflammatory and oxidative stress factors. In the model 1, the inflammatory effect was adjusted; in the model 2, the oxidative stress effect was adjusted; in the model 3, both of them were adjusted.

### Selection of genetic instrumental variants

SNPs associated with blood cell traits were selected as IVs for the modifiable risk factors in the study if they meet genome-wide significant association (*p* < 5 × 10^−8^) with the respective traits. The SNPs were gathered by aggregating all SNPs according to LD (*R* ^2^ = 0.001, 5,000 kb). Palindromic and ambiguous SNPs were discarded. In a two-sample MR, to ensure the consistency of the affected alleles in the instrumental variables (IVs) used in our study, we harmonized the data across different databases using allele frequency information. Moreover, we also excluded weak IVs through the F-statistics of each SNP (Baum et al., 2007). $F\;statistics=R^{2}\left(n-k-1\right)/k\left(1-R^{2}\right)$ , where *R* ^2^ refers to variance of exposure explained by selected IVs, n refers to the sample size, and k refers to the number of IVs (Stock et al., 2002). The large F statistics suggested that these analyses would not be affected by weak instrument bias (Burgess et al., 2011). The details of genetic variants in univariable MR are shown in Supplementary Material S1.

### Data source

Summary-level genetic data were obtained from the database of IEU Open GWAS (https://gwas.mrcieu.ac.uk/) and original GWAS datasets. In our study, we chose the whole subjects based on individuals from Europe. SNPs association with BASO, WBCC, MONO, LYMPH, EOS, and NEUT were derived from data aggregated from the Blood Cell Consortium (Vuckovic et al., 2020). SNPs association with RBCC, RDW, PLT, and PCT were from the European Bioinformatics Institute (Cantelli et al., 2022). In addition, the association of exposure-relevant SNPs with glaucoma was obtained from the UK Biobank (Sudlow et al., 2015). The UK Biobank contains 1,715 glaucoma patients and 359,479 health participants from the European ancestry. The dataset is accessible in public. The characteristics for each phenotype are provided in the Table 1. The SNPs used as IVs for the exposures in this study were obtained from the studies listed in the Table 1.

**TABLE 1:** Details of the summary-level data.

Traits or disease	Source	No. of participants and participant race or ethnicity	No. of SNVs included in the instrumental variable
basophil cell count	Blood Cell Consortium	563,946 European	216
white blood cell count	Blood Cell Consortium	563,946 European	599
monocyte cell count	Blood Cell Consortium	563,946 European	584
lymphocyte cell count	Blood Cell Consortium	563,946 European	584
eosinophil cell count	Blood Cell Consortium	563,946 European	526
neutrophil cell count	Blood Cell Consortium	563,946 European	486
red blood count	European Bioinformatics Institute	172,952 European	193
red cell distribution width	European Bioinformatics Institute	116,666 European	151
platelet count	European Bioinformatics Institute	166,066 European	241
plateletcrit	European Bioinformatics Institute	164,339 European	228
glaucoma	UK Biobank	361,194 European (cases: *n* = 1,715; controls: *n* = 359,479)	NA

### Statistical analysis

The Wald ratio is often accustomed to deriving causal estimates for a single SNP. The inverse-variance–weighted (IVW) method is a method to calculated the Wald ratio of each SNP to assess the causal effects of each SNP on outcome, and finally, the inverse variances of SNPs were used as weights for meta-analysis to evaluate the combined causal effect (Bowden and Holmes, 2019). IVW method under a multiplicative random-effects model to was used to examine the potential causal associations of blood cell parameters with the risk of glaucoma. The results of IVW method were considered as primary results (Burgess et al., 2013). In addition, weighted median accurate MR estimates when less than 50% of IVs have pleiotropic effects (Bowden et al., 2016). Weighted mode is sensitive to the difficult bandwidth selection for mode estimation and simple mode provides robustness for pleiotropy (Hartwig et al., 2017). These three methods were carried out as complementary analyses.

We performed sensitivity analysis to test the robustness of the MR estimates. To assess the robustness of the IVW results, weighted median, MR-Egger and Mendelian randomization pleiotropy residual sum and outlier (MR-PRESSO) methods were conducted (Bowden et al., 2016; Hemani et al., 2018a). The MR-Egger method can still obtain a consistent estimate of the causality even if all selected IVs have pleiotropic effects (Burgess and Thompson, 2017). Moreover, even if at least 50% of the IVs are invalid, the Weighted median still provides unbiased estimates. MR-PRESSO evaluates whether there is a significant difference in the causal estimate before and after adjusting for outliers (Verbanck et al., 2018). Meanwhile, Cochran’s Q-test was performed for the assessment of heterogeneity (Greco et al., 2015). Finally, leave-one-out test was carried out to assess whether the results would be affected by specific SNPs, which test the robustness of the results indirectly.

We analyzed 10 potentially blood cell components and glaucoma (Table 1). All analyses were completed using RStudio software (version 4.2) with the R packages “TwosampleMR” (Hemani et al., 2018b).

### Correction for multiple testing

We employed Holm–Bonferroni method to account for multiple comparisons and adjust statistical significance. Since we analyzed 10 exposures in 3 methods in our MR study, the *p*-value was statistically significant at 0.0017 (where *p* = 0.05/30) after Bonferroni correction (Armstrong, 2014).

### Adjustment for inflammation and oxidative stress

As we hypothesized that inflammatory and oxidative stress may act as confounders of the exposure-outcome relationship, we selected superoxide dismutase (SOD) and C-reactive protein levels (CRP) as instruments and employed multivariable IVW method with three models to analyze the direct effects of blood cell traits on glaucoma. Summary statistics for SOD and CRP are also publicly available (Cantelli et al., 2022). For both exposures in MVMR, SNPs were aggregated based on linkage disequilibrium (LD) (*R* ^2^ = 0.001, 5,000 kb) for both exposures.

## Results

### Instrumental variable selection

In order to perform the MR analysis, we included significant and independent SNPs, and those with an F-statistics <10 were excluded. Finally, a total of 216 BASO-related SNPs, 599 WBCC-related SNPs, 584 LYMPH-related SNPs, 526 EOS-related SNPs, 486 NEUT-related SNPs, 584 MONO-related SNPs, 193 RBCC-related SNPs, 151 RDW-related SNPs, 241 PLT-related SNPs, and 228 PCT-related SNPs were selected for two-sample MR analysis. All these SNPs are listed in Supplementary Material S1.

### Association of basophil cell count, plateletcrit, lymphocyte cell count and platelet count with risk of glaucoma

**FIGURE 2:**
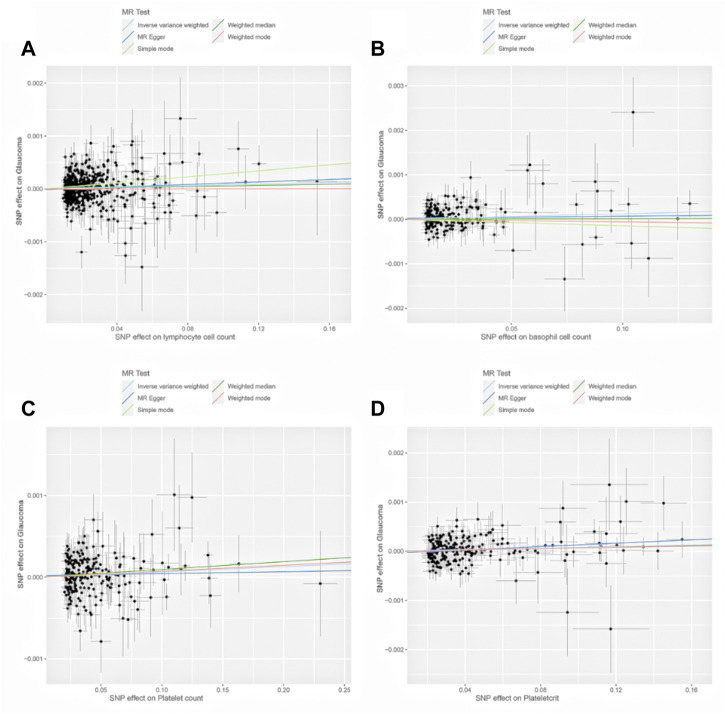
Scatter plots of the genetic associations of lymphocyte cell count **(A)**, basophil cell count **(B)**, platelet count **(C)**, and plateletcrit **(D)** associated SNPs against the genetic associations of glaucoma. The slopes of each line represent the causal association for each method.

Due to our selected dataset, the effect values were generally low, resulting in a much lower likelihood of absolute significance, with all our significant outcomes at the suggestive level. We found evidence for a potential causal effect of BASO on increased risk of glaucoma (IVW model: OR = 1.00122; 95% CI, 1.00003–1.00242, *p* = 0.0451). We also found that genetically assessed PCT showed a positive potential association with glaucoma risk (IVW model: OR = 1.00078, 95% CI, 1.00012–1.00143, *p* = 0.019). Genetically predicted LYMPH was potentially positively associated with glaucoma using the IVW method for European ancestry (LYMPH: OR = 1.00076, 95% CI, 1.00002–1.00151, *p* = 0.045). PLT was potentially associated with glaucoma under the IVW method (PLT: OR = 1.00065, 95% CI, 1.00006–1.00123, *p* = 0.030). Other results from the weighted median, MR-Egger, simple mode, and weighted mode are listed in Supplementary Material S1 (Figure 2; Supplementary Table S11).

### Association of white blood cell count, monocyte cell count, eosinophil cell count, neutrophil cell count, red blood cell count, red blood distribution width with risk of glaucoma

**FIGURE 3:**
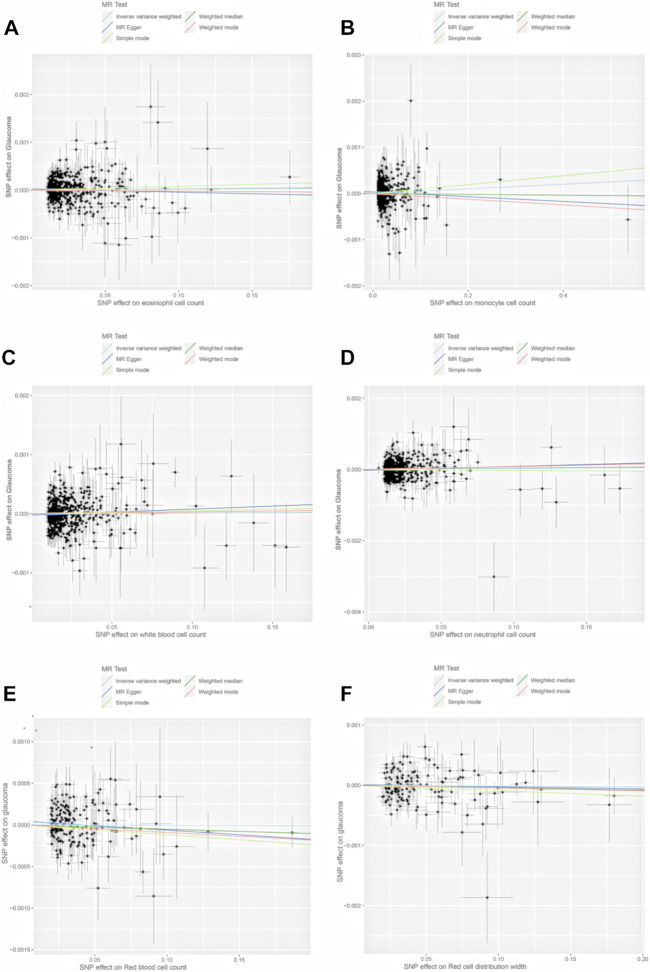
Scatter plots of the genetic associations of eosinophil cell count **(A)**, monocyte cell count **(B)**, white blood cell count **(C)**, neutrophil cell count **(D)**, red blood cell count **(E)**, and red blood distribution width **(F)** associated SNPs against the genetic associations of glaucoma. The slopes of each line represent the causal association for each method.

There was no evidence that WBCC, MONO, EOS, NEUT, RBCC, RDW had causal association with the risk of glaucoma using MR. (Figure 3; Supplementary Table S11).

### Multivariable MR analyses

The association was still robust in the multivariable MR analyses. In model 1, after adjustment for just CRP, BASO, LYMPH, PLT, and PCT still had a potential direct effect on glaucoma [BASO: *p* = 0.048; PCT: *p* = 0.025; LYMPH: *p* = 0.031; PLT: *p* = 0.026]. In model 2 with adjustment for just SOD, the causal association between blood cell traits (BASO, PLT and PCT) and the risk of glaucoma remained potential significant [BASO: *p* = 0.040; PCT: *p* = 0.032; PLT: *p* = 0.049], while LYMPH did not (LYMPH: *p* = 0.063). In the model 3 with adjustment for both CRP and SOD, there was still a potential direct effect of blood cell traits on glaucoma [BASO: *p* = 0.040; PCT: *p* = 0.020; LYMPH: *p* = 0.035; PLT: *p* = 0.040]. Our results are represented in Supplementary Table S12.

### Sensitivity analyses

Sensitivity analyses were conducted to assess the stability and reliability of the results. The heterogeneity test was performed to evaluate potential heterogeneities in the effects of the instruments (Bowden and Holmes, 2019). The random-effects model was applied when heterogeneity was detected. The directional pleiotropies tested by calculating the MR–Egger intercept also showed that there were no pleiotropies. The leave-one-out analysis confirmed the robustness of our MR results by testing the impact of each SNP individually. Finally, we conducted a MR-PRESSO model and identify no outliers and potential pleiotropy. (Supplementary Table S11).

## Discussion

### Key results

Based on comprehensive genetic data from nearly 362,000 individuals, after adjusting for inflammatory and oxidative stress, our study provides evidence supporting a potential causal association between LYMPH, BASO, PLT, PCT, and glaucoma risk, which were also mentioned in previous studies.

### Results in context with the published literature

Observational and experimental studies demonstrate that the immune system, especially T lymphocytes, plays a role in the pathogenesis of glaucoma (Stepp and Menko, 2021; DeMaio et al., 2022). A recent study of the peripheral blood of glaucoma patients performed by Yang et al. reveals that patients of primary open angle glaucoma (POAG) and normal tension glaucoma (NTG) with exhibited a significant increase in CD3+/CD8+ T-lymphocytes and the soluble interleukin-2 receptor, a marker for T lymphocyte activation (Yang et al., 2001). Similar result was found for increased numbers of CD4 (+) CD25 (+) T-cells in glaucoma patients (Bell et al., 2017). *In vivo*, adoptive transfer of T-cells from glaucomatous mice causes a progressive loss of retinal ganglion cell and their axons in recipient mice with a normal IOP (Gramlich et al., 2015). Using MR methods, we found genetic evidence to support that lymphocytes may be causally associated with risk of glaucoma. Chen et al. have been proposed to explain the theory between T lymphocytes and glaucoma. A transient elevation of IOP induces T-cell infiltration into the retina, resulting in a prolonged phase of retinal neurodegeneration (Chen et al., 2018). T-cells are specific for heat shock proteins, which have been recognized as primary autoantigens in glaucoma pathogenesis in both animal disease models (Wax et al., 2008; Geyer and Levo, 2020) and patients (Bell et al., 2013; Gramlich et al., 2013). Furthermore, activated T-cells cross-react with human and bacterial heat shock proteins. Although our finding shows that lymphocyte has a great potential association with glaucoma, Chen et al. (2018) showed changes of T-cell populations in glaucoma especially in observational studies could be explained as a secondary, epiphenomenal effect. We also found evidence for shared genetic influences between BASO and glaucoma, but no studies have explored the specific relationship between BASO and glaucoma. The role of basophils in adaptive immunity *via* the IgE effector arm has been recognized (Blank et al., 2013), suggesting that IgE may play a role in the mechanism of glaucoma, where further investigation is required to provide.

Reports on the association between PLT, PCT and glaucoma are controversial. A recent observational study in POAG patients found that PLT were significantly lower than those of the healthy control group, and PCT were slightly lower than the control group. Notably, the authors suggested that platelet activation, rather than the number of platelets, was more involved in pathomechanisms of POAG by observing significantly elevated platelet distribution width and mean platelet volume in POAG, and platelet distribution width associated with disease severity (Ma et al., 2019). Other study has reported that abnormalities in the coagulation function are significantly associated with the development of primary angle closure glaucoma (PACG), and may be a secondary factor to increase the risk of PACG because only less than 2% PACG patients have abnormal coagulation function parameter values (Li et al., 2017b). However, the MR estimates for PLT and PCT analyzed in this study did reach statistical significance, which provides a new perspective to understanding the pathophysiological mechanisms of glaucoma. Considering platelets play a vital role in the coagulation cascade and in vascular pathophysiology, it is worth investigating the potential mechanism of vascular dysregulation in glaucoma. Additionally, platelet parameters and coagulation function can be easily monitored in clinic, which deserve our further explorations of the pathogenic or protective effects.

Moreover, other indirect parameters concerning blood cell count also support abnormal activity of immune system in glaucoma patients. NLR and platelet-to-lymphocyte ratio have been defined as inflammation biomarkers. Although they have usually been investigated as predictors of prognosis of some diseases, such as cancer (Diem et al., 2017; Graziano et al., 2019; Mungan et al., 2020) and acute ischemic stroke (Chen et al., 2020; Gong et al., 2021), papers for glaucoma are limited (Ozgonul et al., 2016b; Li et al., 2021; Shirvani et al., 2022). A retrospective study investigated the predictive value of NLR and platelet-to-lymphocyte ratio levels in the progression and prognosis of patients with POAG and ocular hypertension (Ozgonul et al., 2016a). Li et al. (2017a) also investigated that NLR was significantly and positively associated with PACG severity, whereas lymphocyte to monocyte ratio was negatively associated with PACG severity. Identifying patients at high risk for glaucoma using easily approachable and affordable biomarkers could have significant clinical implications.

The univariable MR analysis demonstrated a significant association between genetically predicted blood cell traits and the risk of glaucoma. However, this could be explained by the confounding factors including inflammatory and oxidative stress, as previously discussed. Notably, CRP is widely used as an acute marker of inflammation in clinical settings, exhibiting substantial increases during infections and inflammatory conditions (Pathak and Agrawal, 2019). SOD, on the other hand, plays a crucial role in the defense against reactive oxygen species, and its dysfunction is implicated in various oxidative stress-related diseases (Offer et al., 2000). Furthermore, by removing bias caused by CRP and SOD, the multivariable MR analysis revealed consistent and robust causal associations between blood cell traits and the risk of glaucoma.

The effects of RBC traits and WBCC on glaucoma are uncertain. Some observational studies have found increased RBCC (Cohen et al., 2014), RDW (Chen et al., 2019), and WBCC (Li et al., 2017a) are associated with increased glaucoma risk, whereas another showed no association (Firat et al., 2018). The RDW shows the variability in the size of the circulating RBCs, which has a high predictive value for diagnose and prognosis, even for death in the general population (Salvagno et al., 2015). A large-sample case-control study found an elevated RDW was associated with PACG and its severity (Chen et al., 2019). The association between RDW and glaucoma has been suggested to be mediated through oxidative stress (Chidlow et al., 2017), inflammation (Wang et al., 2018), and microangiopathy (Li et al., 2016). Although our MR estimates did not indicate potential causal associations for RDW with risk of glaucoma, more studies are required to clarify the effect of reducing RDW, regardless of whether RDW is considered as a cause or an effect of glaucoma.

Given the relative scarcity of studies on this topic, our findings offer important insights into disease management and progression control. While blood cell counts may appear to be a routine aspect of clinical practice, our MR study sheds light on the potential utility of these accessible and affordable biomarkers in identifying patients at risk for glaucoma. Moreover, our results underscore the need for further research on the underlying mechanisms linking blood cell traits and glaucoma, which may ultimately lead to improved diagnosis, treatment, and outcomes for patients.

### Strengths and limitations

First, to infer causality between blood cell traits and glaucoma, the MR design reduces bias from reverse causality and confounding from observational studies. Multivariable MR analysis accounts for potential confounding caused by inflammatory and oxidative stress. We demonstrated the association between blood cell traits and glaucoma. Second, the 2-sample MR approach included a the largest possible and well-powered genetic variants as IVs, which increase the power to assess the link between each modifiable exposures and glaucoma (Lawlor, 2016). Third, we used different methods including IVW, weighted-median, MR-Egger, and weighted-mode method, the causal estimates of which were consistent, allowing us to employ sensitivity analyses to identify and correct for directional pleiotropy. Lastly, the majority of participants were of European ancestry, thus eliminating bias owning to population stratification.

Our current MR study still has some limitations. While we used summary statistics from the largest known GWAS to date, we did not conduct a stratification analysis of glaucoma. This might cause insufficient statistical power to detect correlations between blood cell traits and different types of glaucoma in different regions. We only use CRP and SOD as mediated effects, where other inflammation and oxidative stress risk factors linked to the genetic variants might not be included in the MVMR models. Since causality could be associated with race, further studies are required in non-European populations. Findings from our study may not introduce into other populations. Besides, since the MR is more considered as a test statistic for a causal hypothesis instead of the expected effect of a clinic intervention, effect estimates from MR analysis should also be regarded cautiously (Didelez and Sheehan, 2007). Although some sensitivity analyses including MR-Egger regression analysis, weighted median, and leave-one-out sensitivity analysis were performed to identify and adjust pleiotropy (Davies et al., 2018), it is not possible to completely rule out the bias from the potential use of invalid IVs (Slob and Burgess, 2020).

In conclusion, the results of this study provide genetic evidence for a potential causal association between increased LYMPH, BASO, PLT, and PCT and the risk of glaucoma, which did not appear to be mediated by CRP and SOD. This was the first two-sample MR investigation into the causal effect of blood cell traits on glaucoma. Altogether, our results may open new avenues of investigation into the specific mechanisms underlying glaucoma, which may lead to guidance on the predication and prevention strategies of this disease.

## Data Availability

The original contributions presented in the study are included in the article/Supplementary Material, further inquiries can be directed to the corresponding author.
