# Supplementary material for: Blood cell traits and risk of glaucoma: A two-sample mendelian randomization study
Source: Front Genet. 2023 Apr 12;14:1142773. doi: 10.3389/fgene.2023.1142773 (PMC10130872; doi:10.3389/fgene.2023.1142773)
Supplement: Supplementary file 1 [file DataSheet1.ZIP › eTable12 Mutivariable Mendelian randomization analysis of modifiable risk factors for glaucoma..pdf]

Associations of lymphocyte cell count with glaucoma in multivariable MR with inverse-variance weighted method with three models.

Model	Exposure	Outcome	nSNP	Beta	SE	OR (95%CI)	P-value
Model 1	C-reactive protein levels	Glaucoma	517	0.0003	0.0004	1.0003（0.9996，1.0011）	0.382
	lymphocyte cell count	Glaucoma	517	0.0009	0.0004	1.0009（1.0001，1.0017）	0.031
Model 2	Superoxide dismutase [Mn],mitochondrial levels	Glaucoma	563	-0.0002	0.0002	0.9998（0.9995，1.0001）	0.256
	lymphocyte cell count	Glaucoma	563	0.0007	0.0004	1.0007（1.0000，1.0015）	0.063
Model 3	C-reactive protein levels	Glaucoma	502	0.0003	0.0004	1.0003（0.9996，1.0010）	0.440
	Superoxide dismutase [Mn],mitochondrial levels	Glaucoma	502	-0.0002	0.0002	0.9998（0.9995，1.0002）	0.317
	lymphocyte cell count	Glaucoma	502	0.0009	0.0004	1.0009（1.0001，1.0016）	0.035

Associations of basophil cell count with glaucoma in multivariable MR with inverse-variance weighted method with three models.

Model	Exposure	Outcome	nSNP	Beta	SE	OR (95%CI)	P-value
Model 1	C-reactive protein levels	Glaucoma	183	0.0003	0.0006	1.0003（0.9990，1.0015）	0.663
	basophil cell count	Glaucoma	183	0.0013	0.0007	1.0013（1.0000，1.0026）	0.048
Model 2	Superoxide dismutase [Mn],mitochondrial levels	Glaucoma	210	0.0003	0.0003	1.0003（0.9998，1.0009）	0.250
	basophil cell count	Glaucoma	210	0.0013	0.0006	1.0013（1.0001，1.0025）	0.040
Model 3	C-reactive protein levels	Glaucoma	178	0.0004	0.0007	1.0004（0.9991，1.0017）	0.530
	Superoxide dismutase [Mn],mitochondrial levels	Glaucoma	178	0.0003	0.0003	1.0003（0.9997，1.0009）	0.350
	basophil cell count	Glaucoma	178	0.0014	0.0007	1.0014（1.0001，1.0027）	0.040

Associations of platelet count with glaucoma in multivariable MR with inverse-variance weighted method with three models.

Model	Exposure	Outcome	nSNP	Beta	SE	OR (95%CI)	P-value
Model 1	Platelet count	Glaucoma	213	0.0007	0.0003	1.0007（1.0001，1.0013）	0.026
	C-reactive protein levels	Glaucoma	213	-0.0005	0.0004	0.9995（0.9986，1.0003）	0.236
Model 2	Platelet count	Glaucoma	229	0.0006	0.0003	1.0006（1.0000，1.0012）	0.049

	Superoxide dismutase [Mn],mitochondrial levels	Glaucoma	229	0.0000	0.0003	1.0000（0.9994，1.0005）	0.847
Model 3	Platelet count	Glaucoma	204	0.0006	0.0003	1.0006（1.0000，1.0013）	0.040
	C-reactive protein levels	Glaucoma	204	-0.0005	0.0005	0.9995（0.9986，1.0004）	0.242
	Superoxide dismutase [Mn],mitochondrial levels	Glaucoma	204	-0.0001	0.0003	0.9999（0.9993，1.0004）	0.589

Associations of plateletcrit with glaucoma in multivariable MR with inverse-variance weighted method with three models.

Model	Exposure	Outcome	nSNP	Beta	SE	OR (95%CI)	P-value
Model 1	Plateletcrit	Glaucoma	207	0.0008	0.0003	1.0008（1.0001，1.0014）	0.025
	C-reactive protein levels	Glaucoma	207	-0.0008	0.0005	0.9992（0.9983，1.0001）	0.076
Model 2	Plateletcrit	Glaucoma	221	0.0007	0.0003	1.0007（1.0001，1.0014）	0.032
	Superoxide dismutase [Mn],mitochondrial levels	Glaucoma	221	0.0000	0.0003	1.0000（0.9995，1.0005）	0.951
Model 3	Plateletcrit	Glaucoma	200	0.0008	0.0003	1.0008（1.0001，1.0015）	0.020
	C-reactive protein levels	Glaucoma	200	-0.0008	0.0005	0.9992（0.9982，1.0001）	0.074
	Superoxide dismutase [Mn],mitochondrial levels	Glaucoma	200	0.0002	0.0003	1.0002（0.9996，1.0007）	0.508
