# Supplementary material for: Blood cell traits and risk of glaucoma: A two-sample mendelian randomization study
Source: Front Genet. 2023 Apr 12;14:1142773. doi: 10.3389/fgene.2023.1142773 (PMC10130872; doi:10.3389/fgene.2023.1142773)
Supplement: Supplementary file 1 [file DataSheet1.ZIP › supplemental online content.docx]

Supplemental Online Content

eTable 1. Basophil cell count exposure SNPs and their association with glaucoma.

eTable 2. Eosinophil cell count exposure SNPs and their association with glaucoma.

eTable 3. Lymphocyte cell count exposure SNPs and their association with glaucoma.

eTable 4. Monocyte cell count exposure SNPs and their association with glaucoma.

eTable 5. Neutrophil cell count exposure SNPs and their association with glaucoma.

eTable 6. White blood cell count exposure SNPs and their association with glaucoma.

eTable 7. Plateletcrit exposure SNPs and their association with glaucoma.

eTable 8. Platelet count exposure SNPs and their association with glaucoma.

eTable 9. Red blood cell count exposure SNPs and their association with glaucoma.

eTable 10. Red cell distribution width exposure SNPs and their association with glaucoma.

eTable 11. Univariable Mendelian randomization analysis of modifiable risk factors for glaucoma.

eTable12 Mutivariable Mendelian randomization analysis of modifiable risk factors for glaucoma.

eFigure1. Leave-one-out permutation analysis plots for basophil cell count on glaucoma.

eFigure2. Leave-one-out permutation analysis plots for lymphocyte cell count on glaucoma.

eFigure3. Leave-one-out permutation analysis plots for plateletcrit on glaucoma.

eFigure4. Leave-one-out permutation analysis plots for platelet count on glaucoma.

eFigure5. Forrest plot of the causal effects of basophil cell count on glaucoma.

eFigure6. Forrest plot of the causal effects of lymphocyte cell count on glaucoma.

eFigure7. Forrest plot of the causal effects of plateletcrit on glaucoma.

eFigure8. Forrest plot of the causal effects of platelet count on glaucoma.

This supplemental material has been provided by the authors to give readers additional information about their work.
