# Supplementary material for: Blood cell traits and risk of glaucoma: A two-sample mendelian randomization study
Source: Front Genet. 2023 Apr 12;14:1142773. doi: 10.3389/fgene.2023.1142773 (PMC10130872; doi:10.3389/fgene.2023.1142773)
Supplement: Supplementary file 1 [file DataSheet1.ZIP › eTable 11. Mendelian randomization analysis of modifiable risk factors for glaucoma..pdf]

eTable 11. Mendelian randomization analysis of modifiable risk factors for glaucoma.

Risk Factor	Method	Glaucoma	
		P value	OR(95%CI)
Basophil cell count	MR Egger	0.687	1.00048(0.99813,1.00284)
	Weighted median	0.905	1.00012(0.99819,1.00205)
	Inverse variance weighted	0.045	1.00122(1.00003,1.00242)
	Simple mode	0.541	0.99854(0.99388,1.00322)
	Weighted mode	0.697	0.99942(0.99649,1.00235)
	MR-PRESSO global test	0.093	NA
	MR-PRESSO outlier test	NA	NA
	MR-PRESSO distortion test	NA	NA
White blood cell count	MR Egger	0.223	1.00101(0.99939,1.00264)
	Weighted median	0.879	1.0001(0.99887,1.00132)
	Inverse variance weighted	0.831	1.00008(0.99932,1.00085)
	Simple mode	0.779	1.00051(0.99698,1.00405)
	Weighted mode	0.814	1.0003(0.99778,1.00283)
	MR-PRESSO global test	0.022	NA
	MR-PRESSO outlier test	1	NA
	MR-PRESSO distortion test	0.745	NA
Monocyte cell count	MR Egger	0.325	0.99949(0.99846,1.00051)
	Weighted median	0.86	0.99991(0.99893,1.00089)
	Inverse variance weighted	0.098	1.0005(0.99991,1.0011)
	Simple mode	0.441	1.00096(0.99852,1.00342)
	Weighted mode	0.385	0.99938(0.998,1.00077)
	MR-PRESSO global test	0.498	NA
	MR-PRESSO outlier test	NA	NA

	MR-PRESSO distortion test	NA	NA
**Lymphocyte cell count**	MR Egger	0.138	1.00118(0.99962,1.00274)
	Weighted median	0.338	1.00055(0.99942,1.00168)
	Inverse variance weighted	0.045	1.00076(1.00002,1.00151)
	Simple mode	0.092	1.00285(0.99954,1.00616)
	Weighted mode	0.985	1.00002(0.9978,1.00225)
	MR-PRESSO global test	0.035	NA
	MR-PRESSO outlier test	1	NA
	MR-PRESSO distortion test	0.926	NA
**Eosinophil cell count**	MR Egger	0.333	0.99933(0.99799,1.00068)
	Weighted median	0.661	1.00024(0.99917,1.00131)
	Inverse variance weighted	0.562	1.0002(0.99952,1.00088)
	Simple mode	0.626	1.00081(0.99757,1.00405)
	Weighted mode	0.848	0.9998(0.99778,1.00183)
	MR-PRESSO global test	0.432	NA
	MR-PRESSO outlier test	NA	NA
	MR-PRESSO distortion test	NA	NA
**Neutrophil cell count**	MR Egger	0.246	1.00107(0.99926,1.00288)
	Weighted median	0.611	1.00035(0.99899,1.00172)
	Inverse variance weighted	0.524	1.00028(0.99943,1.00113)
	Simple mode	0.898	0.99975(0.99591,1.0036)
	Weighted mode	0.458	1.00082(0.99866,1.00299)
	MR-PRESSO global test	0.004	NA
	MR-PRESSO outlier test	1	NA
	MR-PRESSO distortion test	0.963	NA
	MR Egger	0.155	0.99891(0.99740,1.00041)
	Weighted median	0.461	0.99948(0.99811,1.00086)
	Inverse variance weighted	0.920	0.99996(0.99925,1.00068)

**Red blood cell count**	Simple mode	0.416	0.99882(0.99598,1.00167)
	Weighted mode	0.215	0.99909(0.99766,1.00052)
	MR-PRESSO global test	0.323	NA
	MR-PRESSO outlier test	NA	NA
	MR-PRESSO distortion test	NA	NA
**Red cell distribution width**	MR Egger	0.6525	0.99964(0.99807,1.00121)
	Weighted median	0.4883	0.99958(0.99841,1.00076)
	Inverse variance weighted	0.7344	0.99987(0.99911,1.00063)
	Simple mode	0.4581	0.99909(0.99670,1.00149)
	Weighted mode	0.5361	0.99954(0.99810,1.00099)
	MR-PRESSO global test	0.11	NA
	MR-PRESSO outlier test	NA	NA
	MR-PRESSO distortion test	NA	NA
**Platelet count**	MR Egger	0.708	1.00023(0.99902,1.00144)
	Weighted median	0.05	1.00095(1,1.00189)
	Inverse variance weighted	0.03	1.00064(1.00006,1.00123)
	Simple mode	0.471	1.00073(0.99875,1.00271)
	Weighted mode	0.276	1.00073(0.99942,1.00204)
	MR-PRESSO global test	0.391	NA
	MR-PRESSO outlier test	NA	NA
	MR-PRESSO distortion test	NA	NA
**Plateletcrit**	MR Egger	0.014	1.00171(1.00035,1.00307)
	Weighted median	0.191	1.00069(0.99965,1.00173)
	Inverse variance weighted	0.019	1.00078(1.00013,1.00143)
	Simple mode	0.554	1.00065(0.9985,1.0028)
	Weighted mode	0.332	1.00065(0.99934,1.00196)
	MR-PRESSO global test	0.279	NA
	MR-PRESSO outlier test	NA	NA

MR-PRESSO distortion test	NA	NA
