# Supplementary material for: Blood cell traits and risk of glaucoma: A two-sample mendelian randomization study
Source: Front Genet. 2023 Apr 12;14:1142773. doi: 10.3389/fgene.2023.1142773 (PMC10130872; doi:10.3389/fgene.2023.1142773)
Supplement: Supplementary file 1 [file DataSheet1.ZIP › eTable 10. Red cell distribution width exposure SNPs and their association with glaucoma..pdf]

eTable 10. Red cell distribution width exposure SNPs and their association with glaucoma.
Chr = chromosome; POS = position ; EA = effect allele; NEA = non-effect allele; EAF = effect allele frequency; SE = standard error.

SNP	Chr	POS	EA	NEA	EAF	Red cell distribution width		Glaucoma	
						Beta	SE	Beta	SE
rs10900586	1	203654085	A	G	0.9021	0.0609	0.0067	-0.0001	0.0003
rs10923398	1	118251175	C	A	0.1833	-0.0334	0.0051	0.0001	0.0002
rs116831330	1	3738762	C	G	0.0217	0.0749	0.0136	-0.0008	0.0006
rs1172134	1	205248032	G	A	0.9104	-0.0389	0.0069	-0.0004	0.0003
rs1175550	1	3691528	G	A	0.2311	-0.0367	0.0047	0.0002	0.0002
rs1546954	1	230303848	G	T	0.6143	0.0300	0.0041	0.0001	0.0002
rs2022003	1	158586966	T	A	0.2792	0.0439	0.0044	0.0001	0.0002
rs2067054	1	23885230	G	C	0.3417	0.0279	0.0042	0.0003	0.0002
rs3811444	1	248039451	T	C	0.3335	-0.0664	0.0042	-0.0002	0.0002
rs61777615	1	25732087	A	G	0.4214	-0.0400	0.0040	0.0000	0.0002
rs6691993	1	172426998	G	T	0.4934	0.0286	0.0040	0.0000	0.0002
rs701929	1	211846876	G	A	0.4735	-0.0245	0.0040	0.0001	0.0002
rs710214	1	43439282	T	C	0.2196	0.0413	0.0048	0.0000	0.0002
rs72637908	1	40023047	A	T	0.2584	0.0319	0.0045	-0.0003	0.0002
rs11688492	2	64865804	C	T	0.4552	0.0239	0.0040	-0.0001	0.0002
rs183034862	2	169313518	T	C	0.0217	0.1029	0.0145	-0.0001	0.0005
rs1877712	2	219168432	A	G	0.5634	0.0255	0.0040	0.0000	0.0002
rs4672497	2	62523565	G	C	0.2207	-0.0366	0.0048	0.0001	0.0002
rs6730558	2	8756183	T	C	0.3798	-0.0562	0.0041	0.0001	0.0002
rs7606173	2	60725451	C	G	0.4309	-0.0404	0.0040	0.0000	0.0002
rs80250978	2	70027919	T	C	0.1494	0.0307	0.0057	-0.0002	0.0002
rs13078949	3	49361791	G	A	0.4496	0.0198	0.0040	-0.0002	0.0002
rs16839317	3	132218883	C	T	0.1313	0.0371	0.0059	0.0000	0.0002
rs16856859	3	171784736	G	A	0.5824	0.0312	0.0040	0.0000	0.0002
rs2068229	3	141623304	G	A	0.2697	0.0314	0.0045	0.0000	0.0002
rs360408	3	171338569	A	T	0.5635	-0.0416	0.0041	0.0000	0.0002
rs41300435	3	195804021	A	G	0.2401	0.0675	0.0046	-0.0001	0.0002
rs4135265	3	12431296	C	T	0.0326	-0.0608	0.0113	-0.0001	0.0005
rs6779917	3	142347208	G	A	0.5319	0.0231	0.0041	0.0002	0.0002
rs9837644	3	12715292	A	G	0.4241	0.0206	0.0040	-0.0002	0.0002
rs12505188	4	153662239	C	T	0.3989	0.0305	0.0041	0.0003	0.0002
rs2460251	4	39964815	C	G	0.7006	0.0246	0.0044	0.0000	0.0002
rs77829136	4	69734159	A	C	0.1189	0.0338	0.0062	0.0002	0.0003
rs116822287	5	1042549	A	G	0.0672	-0.0588	0.0092	0.0002	0.0004
rs11954023	5	36118981	C	G	0.3903	0.0223	0.0041	-0.0001	0.0002
rs1993878	5	127476971	A	C	0.7535	0.1126	0.0046	0.0000	0.0002
rs2578377	5	153413390	T	C	0.6328	0.0261	0.0041	0.0003	0.0002
rs266428	5	72128767	A	G	0.6840	-0.0269	0.0043	-0.0004	0.0002
rs35188965	5	1104938	T	C	0.5823	-0.0736	0.0040	-0.0001	0.0002
rs7735282	5	175616413	T	G	0.4243	0.0259	0.0040	0.0001	0.0002
rs11758702	6	26120085	A	C	0.0207	-0.1767	0.0156	0.0003	0.0006
rs12661281	6	31842598	A	T	0.1298	-0.0336	0.0059	-0.0001	0.0002
rs141597251	6	26012263	G	C	0.0237	-0.0891	0.0132	0.0006	0.0005
rs145496147	6	26099201	T	C	0.0122	-0.0922	0.0180	0.0019	0.0008
rs190399027	6	26077482	A	G	0.0124	-0.1273	0.0185	0.0003	0.0007
rs3128981	6	31405620	G	A	0.2978	0.0417	0.0043	-0.0001	0.0002
rs35351985	6	3166968	A	G	0.6805	0.0303	0.0043	0.0000	0.0002
rs3813356	6	132834518	T	C	0.4507	0.0222	0.0040	-0.0003	0.0002
rs4554318	6	42012159	T	C	0.4670	-0.0242	0.0041	0.0000	0.0002
rs79220007	6	26098474	C	T	0.0775	-0.1801	0.0074	0.0002	0.0003
rs9349205	6	41925159	A	G	0.2517	0.0535	0.0045	-0.0001	0.0002
rs9376091	6	135419636	T	C	0.2600	-0.0974	0.0045	0.0001	0.0002

rs9487023	6	109590004	G	A	0.4482	-0.0607	0.0040	0.0002	0.0002
rs1049673	7	80306350	G	C	0.4329	0.0345	0.0040	0.0002	0.0002
rs11557288	7	129396174	G	C	0.3374	-0.0225	0.0042	-0.0003	0.0002
rs2178658	7	87122022	T	G	0.2594	-0.0235	0.0046	0.0001	0.0002
rs4316067	7	33081514	G	A	0.2980	0.0699	0.0044	-0.0003	0.0002
rs739385	7	92682609	A	G	0.1496	0.0309	0.0056	-0.0001	0.0002
rs801011	7	74236723	T	C	0.7091	0.0327	0.0045	0.0000	0.0002
rs9801017	7	100236202	A	G	0.6239	-0.0437	0.0041	-0.0002	0.0002
rs1064807	8	26236978	T	C	0.7041	0.0378	0.0044	0.0002	0.0002
rs115849089	8	19912370	A	G	0.1157	0.0557	0.0062	0.0003	0.0003
rs11784833	8	145063412	C	T	0.3820	-0.0221	0.0041	0.0003	0.0002
rs1567668	8	23418122	G	A	0.5717	-0.0264	0.0040	0.0001	0.0002
rs2468024	8	98664653	A	G	0.0674	-0.0721	0.0079	-0.0001	0.0003
rs2954021	8	126482077	G	A	0.5061	0.0422	0.0040	0.0002	0.0002
rs2980478	8	8088230	C	T	0.4766	0.0407	0.0040	0.0002	0.0002
rs3210186	8	144682311	C	G	0.6153	0.0298	0.0041	-0.0004	0.0002
rs4737010	8	41630447	A	G	0.2281	0.0319	0.0048	0.0002	0.0002
rs66593272	8	41548911	T	A	0.0376	-0.0580	0.0104	-0.0003	0.0004
rs10739069	9	4863305	A	T	0.2065	0.0408	0.0049	0.0000	0.0002
rs10988207	9	131867130	A	G	0.6833	0.0385	0.0043	0.0000	0.0002
rs144721045	9	114686305	A	T	0.0137	0.1242	0.0174	0.0002	0.0007
rs61750929	9	91495135	T	C	0.0555	-0.0580	0.0087	0.0004	0.0004
rs72766638	9	136931778	A	C	0.1643	-0.0370	0.0054	0.0003	0.0002
rs8176644	9	136149150	T	C	0.0587	-0.0874	0.0084	0.0001	0.0003
rs9409793	9	97271155	G	C	0.6108	-0.0221	0.0041	0.0000	0.0002
rs11258564	10	13772905	G	A	0.3407	0.0246	0.0043	0.0001	0.0002
rs4332427	10	24858390	G	A	0.2459	0.0754	0.0047	0.0002	0.0002
rs4980169	10	124692586	G	A	0.5780	-0.0242	0.0040	0.0002	0.0002
rs67183237	10	96245896	T	C	0.4838	0.0295	0.0041	0.0000	0.0002
rs77677521	10	45952896	G	C	0.0231	0.1014	0.0133	0.0002	0.0005
rs10160292	11	44595966	G	T	0.2513	-0.0381	0.0046	0.0002	0.0002
rs11606601	11	65354439	G	C	0.4933	-0.0386	0.0040	0.0001	0.0002
rs174559	11	61581656	A	G	0.2822	-0.0588	0.0044	0.0001	0.0002
rs17462448	11	16359567	A	G	0.0821	0.0924	0.0073	-0.0004	0.0003
rs34295904	11	4206701	A	C	0.3631	0.0249	0.0041	-0.0003	0.0002
rs374499153	11	258023	C	T	0.1178	0.0496	0.0063	0.0004	0.0003
rs4910735	11	5258852	A	G	0.4693	0.0255	0.0040	-0.0001	0.0002
rs7115329	11	122949545	G	C	0.0402	0.0610	0.0101	-0.0003	0.0004
rs717662	11	100493995	T	C	0.1077	-0.0501	0.0065	0.0005	0.0003
rs964184	11	116648917	C	G	0.8669	0.0416	0.0059	0.0002	0.0002
rs117444538	12	57414995	C	T	0.0354	0.0616	0.0109	0.0001	0.0004
rs12318272	12	132533737	G	A	0.0246	-0.0914	0.0129	0.0004	0.0005
rs2886271	12	6734313	T	C	0.1701	-0.0304	0.0054	0.0003	0.0002
rs35173156	12	50968330	C	T	0.1958	0.0330	0.0050	-0.0002	0.0002
rs3742015	12	110017125	C	T	0.4394	0.0227	0.0040	0.0000	0.0002
rs55730005	12	121157536	A	C	0.3870	0.0328	0.0041	-0.0002	0.0002
rs79755767	12	54698408	A	G	0.1001	-0.0753	0.0067	-0.0001	0.0003
rs368865	13	113479820	G	A	0.7243	0.0265	0.0044	-0.0001	0.0002
rs76533333	13	113352916	G	A	0.0899	0.0635	0.0070	0.0001	0.0003
rs12883091	14	102669022	A	G	0.1980	0.0310	0.0050	0.0000	0.0002
rs17767662	14	65280824	T	C	0.2891	0.0442	0.0044	0.0001	0.0002
rs2145600	14	58750902	G	C	0.7625	0.0359	0.0047	0.0001	0.0002
rs35372182	14	64236157	G	A	0.1576	0.0344	0.0055	0.0005	0.0002
rs4903088	14	73472776	T	C	0.0250	-0.0834	0.0128	-0.0002	0.0005
rs55796722	14	70328410	T	A	0.8329	-0.0394	0.0054	0.0000	0.0002
rs7148979	14	74547318	A	T	0.1214	-0.0745	0.0061	-0.0005	0.0003
rs8013143	14	23494277	G	A	0.2762	0.0837	0.0044	-0.0002	0.0002
rs12164905	15	40300691	T	G	0.1881	-0.0398	0.0051	-0.0001	0.0002

rs1961489	15	56669395	C	A	0.4345	0.0285	0.0040	-0.0001	0.0002
rs3169166	15	78563103	C	A	0.4159	-0.0504	0.0040	0.0000	0.0002
rs6493544	15	31537559	G	C	0.8813	-0.0310	0.0062	0.0001	0.0003
rs8028409	15	91506422	T	A	0.1291	0.1296	0.0060	-0.0001	0.0002
rs112225541	16	68755165	T	C	0.2832	0.0343	0.0044	-0.0003	0.0002
rs28429148	16	53798319	A	G	0.4330	0.0211	0.0041	0.0002	0.0002
rs7194649	16	215106	A	C	0.1049	-0.0440	0.0067	0.0004	0.0003
rs8046856	16	50098887	C	A	0.7140	0.0314	0.0044	-0.0002	0.0002
rs8050500	16	31404571	C	T	0.4458	-0.0298	0.0040	-0.0001	0.0002
rs12943388	17	27168486	A	G	0.2032	-0.0785	0.0049	0.0005	0.0002
rs225245	17	33946107	G	A	0.4565	0.0259	0.0040	0.0002	0.0002
rs2302783	17	66447073	C	T	0.7150	0.0273	0.0044	0.0003	0.0002
rs2645490	17	57878509	A	G	0.2498	0.0324	0.0046	0.0000	0.0002
rs406400	17	43723930	G	A	0.2203	0.0521	0.0048	0.0004	0.0002
rs429216	17	76126204	G	T	0.1531	0.0369	0.0055	0.0005	0.0002
rs4559945	17	42324057	C	G	0.3082	-0.0277	0.0044	-0.0003	0.0002
rs77183763	17	76183894	A	G	0.0143	-0.0996	0.0171	0.0000	0.0007
rs78079206	17	31298323	A	G	0.1501	-0.0492	0.0056	-0.0006	0.0002
rs8067342	17	21156860	T	C	0.3187	-0.0221	0.0043	-0.0001	0.0002
rs9898189	17	80480516	G	C	0.6448	0.0307	0.0042	0.0000	0.0002
rs12963943	18	43805661	T	C	0.7352	0.0862	0.0045	0.0002	0.0002
rs10405535	19	33072085	G	A	0.6842	-0.0284	0.0044	0.0001	0.0002
rs10410869	19	58141281	T	G	0.1051	-0.0398	0.0065	-0.0001	0.0003
rs10421599	19	35767933	A	G	0.2244	0.0258	0.0048	-0.0005	0.0002
rs11672387	19	12981253	G	C	0.2171	0.0297	0.0048	-0.0001	0.0002
rs11881955	19	17254508	G	A	0.3903	0.0302	0.0041	0.0001	0.0002
rs17678521	19	11293809	G	A	0.0702	-0.0432	0.0079	0.0004	0.0003
rs4420638	19	45422946	G	A	0.1898	-0.0513	0.0051	-0.0002	0.0002
rs4808955	19	19552413	C	T	0.3484	-0.0332	0.0042	-0.0001	0.0002
rs556052	19	49377436	C	G	0.3330	-0.0283	0.0042	0.0001	0.0002
rs62130978	19	4429007	T	C	0.2539	0.0337	0.0046	-0.0001	0.0002
rs7146	19	1014398	G	A	0.6674	-0.0235	0.0042	0.0002	0.0002
rs7252007	19	10687757	C	T	0.8608	-0.0378	0.0058	0.0003	0.0002
rs6014993	20	55991637	G	A	0.4881	-0.0259	0.0040	0.0002	0.0002
rs6124524	20	35626627	T	A	0.1493	-0.0387	0.0056	0.0001	0.0002
rs910889	20	31104950	C	T	0.2201	0.0461	0.0048	0.0000	0.0002
rs7280640	21	35125078	G	C	0.3457	0.0371	0.0042	0.0001	0.0002
rs741951	21	46269526	A	G	0.1502	0.0337	0.0056	0.0003	0.0002
rs140522	22	50971266	C	T	0.6732	-0.0320	0.0042	-0.0004	0.0002
rs28715	22	31730169	T	C	0.4889	0.0227	0.0040	-0.0001	0.0002
rs5995384	22	37519667	A	G	0.3761	0.0225	0.0041	0.0003	0.0002
rs738409	22	44324727	G	C	0.2155	-0.0289	0.0048	0.0001	0.0002
rs855791	22	37462936	G	A	0.5607	-0.1188	0.0040	0.0000	0.0002
rs878825	22	21982249	C	T	0.1930	0.0293	0.0050	-0.0001	0.0002
rs9608946	22	30892255	G	A	0.2345	0.0374	0.0047	0.0001	0.0002
