# Supplementary material for: Blood cell traits and risk of glaucoma: A two-sample mendelian randomization study
Source: Front Genet. 2023 Apr 12;14:1142773. doi: 10.3389/fgene.2023.1142773 (PMC10130872; doi:10.3389/fgene.2023.1142773)
Supplement: Supplementary file 1 [file DataSheet1.ZIP › eTable 9. Red blood cell count exposure SNPs and their association with glaucoma..pdf]

eTable 9. Red blood cell count exposure SNPs and their association with glaucoma.
Chr = chromosome; POS = position ; EA = effect allele; NEA = non-effect allele; EAF = effect allele frequency; SE = standard error.

SNP	Chr	POS	EA	NEA	EAF	Red blood cell count		Glucoma	
						Beta	SE	Beta	SE
rs112682076	1	10357806	C	G	0.0114	-0.0951	0.0171	-0.0003	0.0008
rs1175550	1	3691528	G	A	0.2321	0.0301	0.0042	0.0002	0.0002
rs12069680	1	147284943	A	G	0.1558	0.0278	0.0049	-0.0002	0.0002
rs12121202	1	231442436	T	C	0.1486	0.0323	0.0052	0.0001	0.0002
rs1434282	1	199010721	T	C	0.7265	-0.0448	0.0040	0.0004	0.0002
rs144126567	1	161510516	G	C	0.0741	0.0389	0.0069	-0.0002	0.0003
rs1569419	1	2996602	C	T	0.7653	0.0270	0.0042	-0.0001	0.0002
rs2003943	1	16366365	G	C	0.3672	0.0224	0.0038	0.0000	0.0002
rs3767844	1	214180721	G	A	0.2877	0.0328	0.0040	0.0001	0.0002
rs3811444	1	248039451	T	C	0.3360	0.0424	0.0038	-0.0002	0.0002
rs523395	1	120272477	C	T	0.5339	0.0219	0.0036	-0.0001	0.0002
rs6540556	1	209936631	A	G	0.2311	0.0262	0.0042	0.0000	0.0002
rs6676150	1	155123837	C	G	0.3937	-0.0236	0.0036	-0.0001	0.0002
rs6696846	1	205041952	T	C	0.4870	-0.0249	0.0035	0.0000	0.0002
rs7520050	1	46432105	C	A	0.4138	-0.0344	0.0037	0.0002	0.0002
rs10168349	2	46360907	C	G	0.3309	-0.0642	0.0037	0.0001	0.0002
rs10191559	2	181940642	A	G	0.6988	0.0243	0.0039	-0.0004	0.0002
rs112257498	2	190432985	T	C	0.6135	0.0214	0.0037	-0.0001	0.0002
rs11689538	2	121995638	C	G	0.1401	0.0337	0.0051	-0.0002	0.0002
rs13032454	2	100756009	C	T	0.3600	-0.0260	0.0037	0.0000	0.0002
rs13413838	2	111694797	C	G	0.3383	-0.0285	0.0038	-0.0002	0.0002
rs138308793	2	112250758	A	G	0.1311	-0.0647	0.0054	-0.0002	0.0002
rs143800403	2	112145488	T	C	0.1749	-0.0704	0.0055	0.0000	0.0002
rs17773190	2	47030363	G	A	0.4839	0.0197	0.0036	0.0001	0.0002
rs1966820	2	62357544	G	C	0.3458	-0.0262	0.0037	-0.0002	0.0002
rs243068	2	60621375	T	G	0.4055	-0.0412	0.0036	0.0001	0.0002
rs2665668	2	60768978	A	G	0.6372	0.0244	0.0038	0.0003	0.0002
rs28387101	2	127420426	A	G	0.0447	-0.0488	0.0089	-0.0003	0.0004
rs4669306	2	8754206	A	G	0.6376	-0.0229	0.0037	0.0000	0.0002
rs56262900	2	23897725	A	G	0.1027	0.0558	0.0059	0.0003	0.0003
rs62160676	2	112167931	C	T	0.2974	-0.0583	0.0039	-0.0002	0.0002
rs6740057	2	46292043	A	G	0.0220	-0.0816	0.0122	0.0000	0.0006
rs7583880	2	207999713	A	G	0.6915	-0.0225	0.0039	0.0000	0.0002
rs7607369	2	219279097	G	A	0.5673	0.0333	0.0036	0.0000	0.0002
rs1010553	3	52540773	C	T	0.4846	0.0198	0.0035	0.0002	0.0002
rs1106479	3	195925355	T	C	0.1369	0.0488	0.0053	-0.0002	0.0002
rs17699658	3	71256309	T	C	0.2230	0.0325	0.0043	0.0001	0.0002
rs2361016	3	24341268	T	A	0.6784	-0.0442	0.0039	-0.0002	0.0002
rs35060063	3	194676805	A	G	0.5023	-0.0214	0.0036	0.0000	0.0002
rs4955647	3	169121931	T	A	0.5421	0.0237	0.0036	0.0000	0.0002
rs6791816	3	142233990	C	T	0.5940	0.0372	0.0036	0.0002	0.0002
rs68072945	3	56737268	C	T	0.3018	-0.0358	0.0039	-0.0002	0.0002
rs73146904	3	16932989	A	G	0.0737	0.0486	0.0068	0.0003	0.0003
rs74203182	3	195830380	T	A	0.6959	-0.0486	0.0039	0.0002	0.0002
rs7610312	3	58374029	G	A	0.3704	-0.0216	0.0037	-0.0001	0.0002
rs7625643	3	141150026	G	A	0.4457	0.0340	0.0036	-0.0001	0.0002
rs13103322	4	83895232	G	A	0.1997	-0.0315	0.0046	-0.0001	0.0002
rs13133899	4	115582309	G	A	0.1813	-0.0267	0.0046	-0.0002	0.0002
rs218265	4	55408999	C	T	0.1571	-0.1284	0.0050	0.0001	0.0002
rs4447863	4	9938969	C	T	0.4922	-0.0208	0.0036	0.0001	0.0002
rs4859682	4	77410318	A	C	0.4549	0.0264	0.0036	-0.0003	0.0002

rs58422016	4	55239763	A	G	0.2858	0.0308	0.0040	0.0002	0.0002
rs6414767	4	15655721	G	A	0.6629	0.0229	0.0037	0.0005	0.0002
rs67775544	4	122796917	A	G	0.3958	-0.0207	0.0036	-0.0002	0.0002
rs6816963	4	55378565	C	A	0.1028	-0.0429	0.0059	0.0003	0.0003
rs1542069	5	34507460	G	A	0.4678	-0.0213	0.0036	-0.0001	0.0002
rs2067663	5	88191635	T	C	0.2213	-0.0323	0.0043	0.0002	0.0002
rs2928167	5	76477820	G	A	0.1277	0.0329	0.0053	0.0003	0.0002
rs35188965	5	1104938	T	C	0.5794	-0.0260	0.0036	-0.0001	0.0002
rs4131289	5	176780545	A	G	0.3086	0.0238	0.0039	0.0001	0.0002
rs4449583	5	1284135	T	C	0.3259	0.0398	0.0038	-0.0001	0.0002
rs4958754	5	154027749	C	T	0.3437	-0.0237	0.0038	0.0000	0.0002
rs71624119	5	55440730	A	G	0.2402	0.0238	0.0042	0.0005	0.0002
rs114760566	6	34192036	A	C	0.0453	0.0523	0.0086	-0.0008	0.0004
rs115986297	6	2050791	G	A	0.5428	-0.0317	0.0036	-0.0002	0.0002
rs13213324	6	31327763	G	C	0.1723	0.0399	0.0047	0.0001	0.0002
rs1936807	6	127448249	G	C	0.5182	0.0234	0.0036	0.0000	0.0002
rs34509409	6	31313466	C	T	0.2876	0.0288	0.0040	0.0000	0.0002
rs381500	6	164478388	A	C	0.4520	0.0276	0.0036	0.0004	0.0002
rs636202	6	139843583	C	T	0.5211	0.0439	0.0036	0.0000	0.0002
rs68137036	6	43820215	G	A	0.2869	-0.0330	0.0039	-0.0001	0.0002
rs707892	6	26109233	G	T	0.1618	0.0411	0.0048	-0.0005	0.0002
rs72980278	6	135812251	T	G	0.0538	0.0609	0.0080	0.0006	0.0004
rs75757892	6	7232389	T	C	0.1826	0.0318	0.0046	0.0001	0.0002
rs79920061	6	25857920	A	G	0.0798	-0.0445	0.0066	0.0001	0.0003
rs833806	6	44031083	G	T	0.8828	0.0528	0.0060	0.0002	0.0003
rs9381093	6	41754576	T	C	0.3336	0.0652	0.0041	-0.0001	0.0002
rs9383146	6	16267475	T	C	0.3536	0.0212	0.0037	0.0001	0.0002
rs9399136	6	135402339	C	T	0.2570	-0.1866	0.0041	0.0001	0.0002
rs9464759	6	15177099	C	T	0.0773	0.0479	0.0067	-0.0001	0.0003
rs9471709	6	41956585	C	T	0.2717	0.0787	0.0040	-0.0001	0.0002
rs9487023	6	109590004	G	A	0.4456	-0.0608	0.0036	0.0002	0.0002
rs10224210	7	151413194	C	T	0.2822	-0.0595	0.0040	-0.0001	0.0002
rs1050338	7	44808223	A	G	0.4560	-0.0306	0.0036	-0.0001	0.0002
rs2227665	7	100774596	G	A	0.0255	-0.0788	0.0118	-0.0002	0.0005
rs2237336	7	27208018	T	C	0.4069	0.0200	0.0036	0.0001	0.0002
rs3173804	7	80299850	A	T	0.4345	-0.0247	0.0036	0.0002	0.0002
rs34812229	7	150764388	T	G	0.2498	0.0228	0.0041	0.0003	0.0002
rs551238	7	100321528	T	G	0.5983	-0.0743	0.0036	-0.0002	0.0002
rs62435145	7	1286567	T	G	0.6916	-0.0316	0.0039	-0.0002	0.0002
rs6462990	7	40913646	C	T	0.4112	0.0199	0.0036	-0.0001	0.0002
rs6592965	7	50427982	A	G	0.4547	-0.0495	0.0036	0.0001	0.0002
rs9771385	7	672509	A	G	0.6042	0.0247	0.0037	0.0002	0.0002
rs1905376	8	116533758	G	A	0.5473	0.0267	0.0036	0.0002	0.0002
rs28601761	8	126500031	G	C	0.4184	0.0261	0.0036	0.0000	0.0002
rs2923411	8	42455206	C	T	0.5932	0.0247	0.0036	0.0000	0.0002
rs2978482	8	23427928	A	G	0.4023	-0.0258	0.0037	0.0001	0.0002
rs58141407	8	21791772	T	C	0.1599	-0.0380	0.0049	0.0003	0.0002
rs762679	8	48885436	A	T	0.8533	-0.0319	0.0050	0.0000	0.0002
rs1074449	9	4104571	G	C	0.3405	0.0232	0.0038	-0.0001	0.0002
rs10974716	9	4661574	G	C	0.2583	-0.0296	0.0043	-0.0001	0.0002
rs115478735	9	136149711	T	A	0.1816	-0.0756	0.0046	0.0002	0.0002
rs12001675	9	100793707	A	G	0.2205	0.0279	0.0043	0.0002	0.0002
rs41307428	9	136336804	T	C	0.0196	0.1066	0.0131	-0.0003	0.0006
rs67145503	9	130628524	A	T	0.1169	0.0361	0.0056	0.0001	0.0003
rs7045087	9	32455262	C	T	0.2980	-0.0263	0.0039	0.0003	0.0002
rs7874244	9	4847570	C	T	0.2097	0.0619	0.0044	-0.0001	0.0002
rs7875291	9	13980152	A	G	0.3628	-0.0294	0.0037	-0.0002	0.0002
rs11002791	10	80800318	A	G	0.3158	-0.0249	0.0038	0.0002	0.0002

rs17115100	10	104591393	T	G	0.0851	-0.0347	0.0064	0.0001	0.0003
rs17476364	10	71094504	C	T	0.1103	0.0833	0.0057	-0.0006	0.0003
rs2281841	10	45406608	C	T	0.5803	-0.0474	0.0037	0.0000	0.0002
rs4934494	10	91447419	A	G	0.2262	-0.0242	0.0041	0.0000	0.0002
rs71496605	10	46009818	T	C	0.0677	-0.0594	0.0072	0.0004	0.0003
rs989978	10	101322555	G	A	0.2589	-0.0241	0.0041	0.0001	0.0002
rs10766533	11	19224677	A	T	0.7204	0.0320	0.0040	0.0002	0.0002
rs10836128	11	33909294	G	T	0.6297	-0.0230	0.0037	0.0001	0.0002
rs10890839	11	108306236	A	C	0.4097	0.0243	0.0036	0.0000	0.0002
rs11024008	11	16667834	T	C	0.4139	-0.0202	0.0036	0.0000	0.0002
rs174533	11	61549025	A	G	0.3459	0.0352	0.0037	0.0001	0.0002
rs55893317	11	10180991	G	A	0.2444	-0.0448	0.0041	0.0002	0.0002
rs6484504	11	31424823	C	T	0.7250	0.0285	0.0040	0.0003	0.0002
rs76335321	11	67097778	C	T	0.0829	0.0414	0.0065	0.0000	0.0003
rs7938521	11	8913085	A	G	0.5009	0.0259	0.0036	-0.0002	0.0002
rs10849020	12	4332009	G	C	0.2089	-0.0594	0.0044	-0.0001	0.0002
rs10880864	12	46261301	G	T	0.5017	0.0199	0.0036	-0.0001	0.0002
rs2239760	12	121163518	A	C	0.3969	0.0358	0.0036	-0.0002	0.0002
rs2446066	12	53778650	T	G	0.1738	0.0302	0.0047	0.0005	0.0002
rs2732480	12	48736303	A	C	0.4274	0.0306	0.0036	0.0002	0.0002
rs3184504	12	111884608	C	T	0.5174	-0.0490	0.0035	0.0002	0.0002
rs35407591	12	2517887	A	G	0.3734	0.0304	0.0037	-0.0001	0.0002
rs4242906	12	133105852	G	A	0.2298	0.0317	0.0042	-0.0005	0.0002
rs6538148	12	88818479	G	C	0.7032	-0.0295	0.0039	-0.0001	0.0002
rs1340817	13	29230581	G	A	0.3535	-0.0205	0.0037	0.0001	0.0002
rs6602909	13	114551993	C	T	0.3251	0.0244	0.0038	0.0000	0.0002
rs9521011	13	109412704	G	A	0.5272	0.0238	0.0036	0.0000	0.0002
rs9549260	13	41254104	A	C	0.2153	0.0260	0.0043	-0.0003	0.0002
rs9573567	13	76057383	A	G	0.0992	0.0512	0.0060	0.0000	0.0003
rs11627485	14	65487694	C	T	0.4468	-0.0275	0.0036	-0.0001	0.0002
rs1256061	14	64703593	T	G	0.4769	-0.0218	0.0036	-0.0003	0.0002
rs45569432	14	74666225	C	T	0.1173	-0.0304	0.0056	-0.0004	0.0003
rs72725174	14	68510773	T	C	0.1629	0.0379	0.0048	0.0000	0.0002
rs11072506	15	75052994	G	A	0.7126	-0.0235	0.0039	-0.0002	0.0002
rs11072567	15	76298744	G	A	0.5129	-0.0328	0.0036	0.0001	0.0002
rs28552840	15	66066618	C	G	0.2421	0.0332	0.0042	-0.0003	0.0002
rs116971887	16	51170026	T	G	0.0455	-0.0501	0.0087	-0.0002	0.0004
rs12448902	16	28871191	G	C	0.3968	-0.0238	0.0037	0.0000	0.0002
rs2238368	16	170328	T	C	0.4608	0.0438	0.0036	0.0003	0.0002
rs28647874	16	67876823	G	A	0.0917	0.0551	0.0069	0.0000	0.0003
rs3809627	16	30103160	A	C	0.4017	0.0392	0.0036	-0.0002	0.0002
rs4889604	16	30985994	T	G	0.6132	0.0204	0.0034	0.0000	0.0002
rs74035509	16	88567333	T	C	0.0790	0.0445	0.0067	0.0001	0.0003
rs837763	16	88853729	T	C	0.5541	-0.0391	0.0036	0.0002	0.0002
rs12453682	17	37770005	T	C	0.6933	-0.0299	0.0039	0.0000	0.0002
rs2106786	17	43919096	G	A	0.2242	0.0537	0.0043	0.0004	0.0002
rs2748427	17	76121864	G	A	0.2187	0.0354	0.0043	0.0003	0.0002
rs34121753	17	7733833	G	A	0.5768	-0.0204	0.0036	-0.0003	0.0002
rs35999311	17	46219650	G	C	0.2448	-0.0266	0.0041	0.0000	0.0002
rs4791641	17	8161149	T	C	0.5020	-0.0233	0.0036	-0.0002	0.0002
rs7213285	17	27206029	A	G	0.1646	-0.0337	0.0048	0.0004	0.0002
rs72834846	17	53242391	T	A	0.2033	-0.0263	0.0044	0.0002	0.0002
rs80014635	17	20197983	C	T	0.2786	0.0243	0.0045	-0.0004	0.0002
rs9895661	17	59456589	T	C	0.8295	-0.0327	0.0047	0.0001	0.0002
rs17758695	18	60920854	T	C	0.0300	-0.0925	0.0105	0.0000	0.0005
rs78415359	18	46207268	A	G	0.0324	-0.0641	0.0101	-0.0005	0.0005
rs8093407	18	43849464	G	A	0.7491	0.0427	0.0041	0.0002	0.0002
rs9952412	18	46465540	C	A	0.5181	-0.0204	0.0036	-0.0002	0.0002

rs10415135	19	4061544	T	C	0.1907	0.0509	0.0046	0.0000	0.0002
rs10426059	19	50090607	G	C	0.2327	0.0288	0.0042	-0.0001	0.0002
rs113125564	19	47596131	C	T	0.0446	0.0632	0.0086	0.0001	0.0004
rs11667352	19	33918423	G	A	0.3934	-0.0214	0.0037	0.0001	0.0002
rs12052092	19	41318899	A	T	0.3338	0.0238	0.0038	-0.0002	0.0002
rs123698	19	807442	C	G	0.6049	0.0203	0.0036	-0.0002	0.0002
rs148614081	19	41204070	A	G	0.0216	-0.0907	0.0131	0.0009	0.0006
rs16989695	19	4505445	A	G	0.5178	-0.0265	0.0035	0.0000	0.0002
rs2853333	19	35747663	T	C	0.5432	-0.0235	0.0036	0.0003	0.0002
rs56397034	19	13000550	C	G	0.3891	-0.0495	0.0036	0.0000	0.0002
rs57908212	19	2161321	C	T	0.4747	0.0281	0.0036	0.0000	0.0002
rs74929147	19	18413061	A	G	0.0600	0.0474	0.0077	0.0000	0.0004
rs78744187	19	33754548	T	C	0.0818	0.0975	0.0065	-0.0004	0.0003
rs159058	20	31108108	C	A	0.2995	0.0247	0.0039	-0.0002	0.0002
rs6126019	20	49101590	C	T	0.5467	0.0342	0.0036	0.0000	0.0002
rs6138599	20	25517425	G	T	0.1802	-0.0326	0.0046	-0.0005	0.0002
rs737092	20	55990405	C	T	0.4891	-0.0355	0.0036	0.0002	0.0002
rs766622	20	42673848	T	C	0.7019	-0.0229	0.0039	-0.0001	0.0002
rs1997595	21	16578159	C	A	0.3405	-0.0297	0.0038	-0.0002	0.0002
rs2037977	21	16785682	A	G	0.2836	-0.0256	0.0040	-0.0001	0.0002
rs2269188	21	38072356	C	G	0.2783	-0.0276	0.0041	0.0002	0.0002
rs2834253	21	35093213	C	T	0.3774	0.0204	0.0037	0.0001	0.0002
rs2835349	21	37814114	C	T	0.5448	-0.0229	0.0036	0.0000	0.0002
rs743417	21	35347960	T	C	0.5762	-0.0237	0.0036	0.0000	0.0002
rs140522	22	50971266	C	T	0.6734	-0.0523	0.0038	-0.0004	0.0002
rs28496879	22	46390243	T	C	0.6009	-0.0203	0.0037	0.0001	0.0002
rs5998517	22	32899045	C	T	0.5986	0.0270	0.0037	0.0001	0.0002
rs8138197	22	43114551	A	G	0.4713	0.0354	0.0036	0.0000	0.0002
rs9330787	22	46319722	A	T	0.2345	0.0277	0.0042	-0.0001	0.0002
