# Supplementary material for: Blood cell traits and risk of glaucoma: A two-sample mendelian randomization study
Source: Front Genet. 2023 Apr 12;14:1142773. doi: 10.3389/fgene.2023.1142773 (PMC10130872; doi:10.3389/fgene.2023.1142773)
Supplement: Supplementary file 1 [file DataSheet1.ZIP › eTable 8. Platelet count exposure SNPs and their association with glaucoma.pdf]

eTable 8. Platelet count exposure SNPs and their association with glaucoma.
Chr = chromosome; POS = position ; EA = effect allele; NEA = non-effect allele; EAF = effect allele
frequency; SE = standard error.

SNP	Chr	POS	EA	NEA	EAF	Platelet Count		Glaucoma	
						Beta	SE	Beta	SE
rs1050316	1	156434703	T	G	0.6515	-0.0254	0.0038	0.0001	0.0002
rs10923354	1	118152241	C	T	0.2227	0.0331	0.0044	0.0002	0.0002
rs11121559	1	10455148	A	G	0.0805	-0.0421	0.0068	0.0005	0.0003
rs11121845	1	12028655	T	C	0.4631	-0.0391	0.0037	0.0000	0.0002
rs111941366	1	25736369	T	C	0.4501	-0.0432	0.0037	-0.0001	0.0002
rs11207138	1	40392157	C	G	0.2656	0.0267	0.0041	0.0000	0.0002
rs1172137	1	205246061	T	G	0.3893	0.0459	0.0037	0.0001	0.0002
rs1184547	1	63007228	A	G	0.6445	-0.0211	0.0038	0.0001	0.0002
rs12061136	1	248023684	G	T	0.291	0.0378	0.0040	0.0002	0.0002
rs12140829	1	43849917	G	A	0.0327	0.0563	0.0102	0.0001	0.0005
rs12744266	1	248047667	A	G	0.331	-0.0244	0.0040	-0.0002	0.0002
rs140584594	1	110232983	G	A	0.7047	0.0255	0.0046	-0.0002	0.0002
rs1538970	1	45847562	A	G	0.2329	-0.0394	0.0044	-0.0001	0.0002
rs1819663	1	154025891	G	A	0.4886	0.0201	0.0036	-0.0002	0.0002
rs1826164	1	88104648	G	A	0.0567	-0.0437	0.0079	-0.0004	0.0004
rs2932536	1	113202417	A	G	0.5261	-0.0346	0.0036	0.0000	0.0002
rs4631704	1	230293530	T	C	0.6104	0.0238	0.0038	0.0001	0.0002
rs56043070	1	247719769	A	G	0.0714	-0.1388	0.0070	0.0000	0.0003
rs6425521	1	171942783	A	C	0.8012	-0.0652	0.0046	0.0003	0.0002
rs6667939	1	198994619	T	C	0.7195	0.0338	0.0041	0.0003	0.0002
rs7516301	1	172372260	C	T	0.5249	0.0212	0.0037	0.0000	0.0002
rs76452111	1	171810155	T	A	0.0433	-0.0626	0.0092	-0.0002	0.0004
rs78265569	1	2146165	A	C	0.0894	-0.0458	0.0065	0.0003	0.0003
rs834232	1	150354682	C	T	0.395	0.0223	0.0037	0.0001	0.0002
rs928392	1	156770816	C	T	0.7437	0.0267	0.0042	-0.0002	0.0002
rs9438594	1	28242412	C	A	0.3692	-0.0217	0.0038	0.0000	0.0002
rs945631	1	93426167	A	G	0.0419	-0.0514	0.0093	-0.0004	0.0004
rs10048745	2	68962137	A	G	0.2597	0.0293	0.0043	0.0000	0.0002
rs1047891	2	211540507	A	C	0.3159	-0.0342	0.0039	0.0002	0.0002
rs11684299	2	227296995	T	C	0.1942	0.0356	0.0046	0.0002	0.0002
rs12052715	2	160677375	G	C	0.7247	-0.0344	0.0041	0.0000	0.0002
rs1260326	2	27730940	C	T	0.605	-0.0386	0.0037	0.0000	0.0002
rs149290349	2	43451957	A	G	0.0759	-0.0827	0.0071	-0.0001	0.0003
rs17572109	2	219093934	A	G	0.2317	0.0388	0.0044	0.0002	0.0002
rs4670779	2	38044344	T	C	0.3161	-0.0244	0.0040	0.0001	0.0002
rs62160676	2	112167931	C	T	0.2975	0.0299	0.0040	-0.0002	0.0002
rs655029	2	31477838	A	G	0.7113	0.0735	0.0041	-0.0001	0.0002
rs7585866	2	192696255	G	A	0.3532	0.0234	0.0038	0.0001	0.0002
rs78909033	2	241510903	A	G	0.1361	0.0653	0.0053	0.0001	0.0002
rs80352135	2	70149873	A	G	0.1957	-0.0285	0.0046	-0.0002	0.0002
rs934725	2	121012902	T	C	0.6884	-0.0351	0.0039	-0.0004	0.0002
rs10513797	3	184092481	A	G	0.0833	0.0449	0.0066	0.0000	0.0003
rs115243514	3	183926133	C	G	0.0177	0.1004	0.0139	0.0002	0.0006
rs13092376	3	196516288	C	A	0.4195	-0.0246	0.0037	0.0000	0.0002
rs1354034	3	56849749	C	T	0.6023	0.1379	0.0037	0.0003	0.0002
rs167924	3	107379837	G	A	0.6215	0.0211	0.0038	-0.0001	0.0002
rs2046823	3	56779011	A	G	0.2624	0.0285	0.0041	-0.0001	0.0002
rs3804749	3	122833003	T	C	0.5929	-0.0413	0.0037	0.0004	0.0002
rs6141	3	184090266	T	C	0.5321	0.0621	0.0037	0.0000	0.0002
rs619460	3	124345877	A	G	0.6228	-0.0237	0.0037	0.0002	0.0002
rs62259323	3	58291047	G	A	0.3219	-0.0305	0.0039	0.0000	0.0002
rs6444472	3	167373162	A	G	0.259	0.0229	0.0042	-0.0001	0.0002

rs6768432	3	168815374	T	G	0.6058	-0.0293	0.0037	0.0000	0.0002
rs7641175	3	18311412	A	G	0.7809	0.0458	0.0044	-0.0002	0.0002
rs7641761	3	178740422	A	T	0.6974	-0.0276	0.0040	-0.0003	0.0002
rs7646123	3	56937119	C	G	0.4123	-0.0212	0.0038	0.0000	0.0002
rs9809116	3	72397279	G	A	0.4076	0.0265	0.0037	0.0001	0.0002
rs9810259	3	12268191	G	C	0.4213	-0.0409	0.0037	0.0000	0.0002
rs111856038	4	124745030	A	G	0.1092	0.0348	0.0059	0.0003	0.0003
rs113128512	4	157682188	C	T	0.0918	-0.0384	0.0063	0.0000	0.0003
rs1155577	4	120449810	T	C	0.4868	0.0217	0.0036	0.0000	0.0002
rs11723371	4	145025810	C	T	0.4732	0.0227	0.0037	0.0002	0.0002
rs11734099	4	6891435	A	G	0.176	0.0574	0.0048	-0.0001	0.0002
rs2724563	4	152349959	C	G	0.5577	0.0257	0.0037	-0.0001	0.0002
rs34080918	4	17779295	T	A	0.2521	0.0236	0.0043	0.0001	0.0002
rs3752440	4	3446754	A	G	0.0844	0.0391	0.0066	0.0002	0.0003
rs71633359	4	88183820	C	T	0.318	0.0251	0.0040	-0.0001	0.0002
rs72952320	4	106211980	A	G	0.0321	-0.0681	0.0103	0.0005	0.0005
rs7665147	4	57767327	A	T	0.1855	-0.0317	0.0047	-0.0004	0.0002
rs7698924	4	110942102	C	G	0.3163	-0.0310	0.0039	-0.0002	0.0002
rs10058074	5	131686146	A	G	0.4743	-0.0317	0.0036	-0.0004	0.0002
rs10075570	5	158599358	A	G	0.2503	-0.0278	0.0042	0.0000	0.0002
rs10514301	5	87939654	T	C	0.1177	0.0394	0.0057	0.0001	0.0003
rs10940072	5	65916784	A	G	0.3989	-0.0267	0.0037	-0.0001	0.0002
rs114694170	5	88180196	C	T	0.059	0.1632	0.0079	0.0002	0.0003
rs2042974	5	77817169	C	G	0.3292	0.0226	0.0040	-0.0004	0.0002
rs34592828	5	75996909	A	G	0.044	-0.1399	0.0089	0.0002	0.0004
rs3916720	5	72022772	A	C	0.1313	-0.0330	0.0055	0.0007	0.0002
rs457648	5	34658419	T	C	0.4623	0.0221	0.0037	-0.0001	0.0002
rs4704734	5	156480406	A	G	0.8279	-0.0299	0.0048	0.0004	0.0002
rs59739601	5	111061017	G	A	0.0765	-0.0551	0.0069	0.0000	0.0003
rs6556471	5	159598264	C	T	0.6776	-0.0462	0.0039	-0.0002	0.0002
rs6860138	5	141509985	A	G	0.6166	0.0312	0.0037	-0.0005	0.0002
rs7705526	5	1285974	A	C	0.3289	0.0367	0.0040	0.0000	0.0002
rs1385742	6	47595155	T	A	0.6484	-0.0296	0.0039	-0.0002	0.0002
rs1406833	6	113463868	G	A	0.2059	-0.0246	0.0045	0.0002	0.0002
rs16896742	6	29922740	G	A	0.361	0.0306	0.0038	0.0003	0.0002
rs198812	6	26128410	C	G	0.8598	0.0347	0.0052	-0.0001	0.0002
rs2057149	6	110717493	T	C	0.6485	0.0288	0.0038	0.0000	0.0002
rs2078064	6	52267254	A	G	0.11	0.0380	0.0058	0.0001	0.0003
rs210143	6	33546930	C	T	0.7021	0.1020	0.0040	0.0000	0.0002
rs210798	6	135514558	G	T	0.5204	-0.0209	0.0037	-0.0002	0.0002
rs214053	6	25527963	C	T	0.4414	-0.0479	0.0037	-0.0001	0.0002
rs28366156	6	31671498	C	T	0.1302	-0.0348	0.0054	-0.0002	0.0002
rs2894802	6	52656169	G	T	0.5733	-0.0280	0.0037	-0.0002	0.0002
rs3132520	6	31140008	T	C	0.5029	-0.0267	0.0037	0.0000	0.0002
rs4709819	6	164463355	A	G	0.4063	0.0317	0.0037	0.0003	0.0002
rs4711493	6	37293733	A	G	0.6499	-0.0253	0.0038	0.0000	0.0002
rs487358	6	33570932	A	G	0.4522	0.0330	0.0037	0.0000	0.0002
rs6925716	6	109597641	C	T	0.5132	0.0300	0.0037	0.0002	0.0002
rs7776054	6	135418916	G	A	0.2605	0.1192	0.0042	0.0001	0.0002
rs9266658	6	31347644	A	G	0.1627	0.0581	0.0049	-0.0001	0.0002
rs9376060	6	135052237	G	A	0.2192	0.0331	0.0044	0.0000	0.0002
rs11556924	7	129663496	T	C	0.3886	0.0264	0.0037	-0.0002	0.0002
rs11562010	7	116521644	A	T	0.4374	0.0264	0.0037	-0.0001	0.0002
rs1182180	7	2873279	T	G	0.4316	0.0292	0.0037	-0.0001	0.0002
rs17145750	7	73026378	T	C	0.1611	-0.0294	0.0050	0.0001	0.0002
rs2331174	7	44926827	A	G	0.434	-0.0363	0.0037	-0.0002	0.0002
rs2710804	7	36084529	C	T	0.3777	0.0223	0.0038	0.0004	0.0002
rs342292	7	106370644	G	C	0.4534	-0.0716	0.0037	0.0002	0.0002

rs4434553	7	100240191	G	A	0.4941	0.0235	0.0036	-0.0002	0.0002
rs6961069	7	80218961	T	C	0.4042	0.0261	0.0038	0.0000	0.0002
rs73162998	7	135628370	C	T	0.0763	0.0401	0.0069	0.0002	0.0003
rs77300440	7	123411910	T	C	0.0807	0.0686	0.0067	0.0003	0.0003
rs7788849	7	129281919	A	C	0.9023	-0.0443	0.0062	-0.0001	0.0003
rs7811142	7	100065443	T	A	0.1968	0.0358	0.0046	0.0004	0.0002
rs11993146	8	30319537	A	G	0.2328	-0.0266	0.0043	0.0000	0.0002
rs11993233	8	145002283	G	A	0.428	0.0440	0.0037	0.0002	0.0002
rs60148718	8	66871899	A	T	0.3579	-0.0229	0.0038	-0.0002	0.0002
rs6470811	8	131338933	T	G	0.4879	-0.0232	0.0036	-0.0002	0.0002
rs6993770	8	106581528	T	A	0.2857	-0.0700	0.0040	0.0001	0.0002
rs10118655	9	38196563	G	A	0.4888	-0.0288	0.0037	-0.0002	0.0002
rs10811664	9	22142907	A	G	0.1565	-0.0587	0.0050	0.0001	0.0002
rs10818964	9	127190340	G	A	0.6522	-0.0230	0.0039	0.0002	0.0002
rs10820606	9	99192919	C	A	0.2333	0.0499	0.0044	-0.0003	0.0002
rs11142447	9	73070284	T	C	0.4371	-0.0240	0.0037	-0.0001	0.0002
rs114968084	9	136022954	A	G	0.0279	-0.0776	0.0119	0.0003	0.0005
rs2157770	9	136921464	G	A	0.2858	0.0383	0.0040	0.0004	0.0002
rs2296825	9	327938	C	G	0.6501	0.0281	0.0038	0.0000	0.0002
rs35774855	9	4887465	C	T	0.5223	0.0395	0.0037	0.0001	0.0002
rs385893	9	4763176	C	T	0.5222	0.1055	0.0036	0.0001	0.0002
rs60757417	9	135864436	G	C	0.0607	-0.0852	0.0078	-0.0002	0.0003
rs7028112	9	5048814	A	G	0.4975	-0.0416	0.0036	-0.0001	0.0002
rs7874405	9	21980944	T	C	0.7117	0.0399	0.0040	-0.0001	0.0002
rs905358	9	91505634	G	A	0.0587	-0.0723	0.0078	0.0005	0.0004
rs10761741	10	65066186	T	G	0.4176	0.0769	0.0037	0.0002	0.0002
rs116052829	10	81164146	T	C	0.1048	0.0376	0.0060	-0.0004	0.0003
rs12266014	10	25211291	T	C	0.369	-0.0233	0.0038	0.0003	0.0002
rs1720607	10	30511198	A	T	0.6715	0.0219	0.0040	0.0001	0.0002
rs2068888	10	94839642	A	G	0.4509	-0.0237	0.0037	0.0001	0.0002
rs4272720	10	50263201	G	A	0.2363	-0.0350	0.0043	-0.0003	0.0002
rs7067734	10	14615358	T	G	0.3335	0.0216	0.0039	0.0002	0.0002
rs10769960	11	8819003	C	T	0.4368	-0.0331	0.0037	0.0001	0.0002
rs10893909	11	128565034	T	C	0.2522	-0.0317	0.0042	-0.0001	0.0002
rs1111890	11	100501571	G	C	0.3653	0.0209	0.0038	0.0000	0.0002
rs1150339	11	94833689	G	A	0.7907	0.0254	0.0045	0.0000	0.0002
rs11604127	11	196944	T	C	0.2356	0.0931	0.0043	0.0001	0.0002
rs174548	11	61571348	G	C	0.3146	0.0386	0.0039	0.0001	0.0002
rs192022	11	108248774	G	C	0.4528	0.0304	0.0038	-0.0002	0.0002
rs36109901	11	119083318	C	A	0.2722	0.0565	0.0041	0.0000	0.0002
rs3741404	11	63999240	C	G	0.3596	-0.0224	0.0038	-0.0003	0.0002
rs4937127	11	126290510	G	A	0.5219	-0.0320	0.0036	-0.0002	0.0002
rs4937333	11	128330520	C	T	0.5264	-0.0322	0.0037	0.0000	0.0002
rs573589	11	65483981	T	C	0.4398	-0.0279	0.0037	0.0000	0.0002
rs645901	11	116702362	C	T	0.8657	0.0442	0.0054	0.0002	0.0002
rs655641	11	85731286	G	C	0.8086	0.0266	0.0045	0.0000	0.0002
rs73000929	11	113953622	A	G	0.0374	-0.0919	0.0098	-0.0005	0.0004
rs7950696	11	47481533	C	T	0.445	0.0274	0.0037	0.0001	0.0002
rs10466905	12	6502832	A	G	0.1919	0.0276	0.0047	0.0000	0.0002
rs10783794	12	56987179	G	A	0.3891	0.0346	0.0038	0.0000	0.0002
rs113825134	12	78220740	A	G	0.2335	-0.0248	0.0043	0.0003	0.0002
rs11553699	12	122216910	G	A	0.1364	-0.0801	0.0056	0.0004	0.0002
rs11559982	12	54711574	G	A	0.5575	0.0571	0.0037	0.0000	0.0002
rs12824685	12	123817569	T	G	0.205	-0.0324	0.0045	-0.0001	0.0002
rs1716505	12	65005079	G	C	0.3217	0.0440	0.0040	-0.0001	0.0002
rs2015599	12	29435480	A	G	0.4585	-0.0445	0.0036	-0.0002	0.0002
rs2255531	12	121414915	A	G	0.3495	-0.0242	0.0038	0.0003	0.0002
rs3184504	12	111884608	C	T	0.5174	-0.1039	0.0036	0.0002	0.0002

rs34038797	12	740009	G	C	0.4858	-0.0297	0.0038	0.0001	0.0002
rs35277580	12	6294534	A	G	0.4908	-0.0461	0.0036	0.0000	0.0002
rs35427	12	115556307	G	T	0.3828	-0.0256	0.0039	0.0003	0.0002
rs35624680	12	51201607	G	T	0.3686	0.0213	0.0039	0.0003	0.0002
rs4388979	12	109475012	T	G	0.5834	-0.0435	0.0037	-0.0001	0.0002
rs73109811	12	48212719	T	C	0.1982	0.0407	0.0047	-0.0002	0.0002
rs7958679	12	49662391	T	C	0.0743	-0.0438	0.0070	-0.0007	0.0003
rs11841319	13	110492626	T	C	0.1028	-0.0659	0.0061	-0.0002	0.0003
rs374039502	13	108960385	A	T	0.021	-0.0816	0.0137	0.0001	0.0006
rs4148435	13	95899716	A	C	0.9161	0.0747	0.0066	-0.0002	0.0003
rs61966631	13	114015702	C	T	0.3562	0.0280	0.0038	-0.0002	0.0002
rs670179	13	71236607	A	T	0.5722	0.0282	0.0037	0.0002	0.0002
rs7332763	13	33147888	G	T	0.3714	0.0237	0.0038	0.0002	0.0002
rs10144272	14	103086289	G	T	0.6984	0.0486	0.0040	0.0000	0.0002
rs10220411	14	69452088	G	A	0.2615	0.0330	0.0042	-0.0003	0.0002
rs11627485	14	65487694	C	T	0.4476	0.0258	0.0037	-0.0001	0.0002
rs17580	14	94847262	A	T	0.0482	0.0500	0.0085	-0.0008	0.0004
rs2297066	14	103566835	G	C	0.2436	0.0555	0.0042	-0.0001	0.0002
rs36084521	14	93516398	G	T	0.1097	0.0327	0.0058	0.0003	0.0003
rs3844535	14	81884515	G	A	0.6999	0.0237	0.0040	0.0000	0.0002
rs4470077	14	55897538	G	A	0.1909	0.0293	0.0047	-0.0002	0.0002
rs7142089	14	101172229	A	T	0.2148	-0.0541	0.0047	-0.0001	0.0002
rs7146395	14	75228647	C	A	0.6738	-0.0227	0.0039	0.0001	0.0002
rs72725172	14	68509318	G	A	0.1703	-0.0545	0.0048	0.0000	0.0002
rs11071720	15	63341996	C	T	0.6998	0.0496	0.0040	0.0002	0.0002
rs1631677	15	65198591	G	A	0.1432	0.0474	0.0052	0.0006	0.0002
rs4924314	15	39248441	T	C	0.3788	-0.0250	0.0038	-0.0002	0.0002
rs4965426	15	99248041	A	G	0.1429	-0.0365	0.0052	-0.0005	0.0002
rs55707100	15	43820717	T	C	0.0261	0.1143	0.0114	0.0006	0.0005
rs62027291	15	77266051	A	G	0.1628	-0.0280	0.0050	-0.0001	0.0002
rs7178196	15	57078278	A	G	0.1669	-0.0345	0.0049	0.0001	0.0002
rs8037137	15	91506637	C	T	0.1299	-0.0354	0.0054	-0.0001	0.0002
rs13336575	16	89034068	A	G	0.1627	0.0284	0.0050	-0.0001	0.0002
rs141759085	16	530967	G	A	0.081	0.0480	0.0067	0.0003	0.0003
rs151233	16	28506428	T	C	0.1308	0.0648	0.0054	-0.0002	0.0002
rs183725	16	9048035	C	T	0.7676	0.0260	0.0044	0.0002	0.0002
rs191157391	16	85443198	C	T	0.2954	0.0239	0.0043	0.0000	0.0002
rs4334315	16	79756197	T	A	0.3051	0.0223	0.0041	-0.0001	0.0002
rs4783186	16	85415734	C	T	0.8767	-0.0422	0.0056	0.0000	0.0002
rs59865663	16	88558312	A	G	0.2042	0.0408	0.0046	0.0001	0.0002
rs11653357	17	33923607	A	G	0.1737	0.0640	0.0048	0.0000	0.0002
rs12325879	17	57627786	C	T	0.2924	-0.0225	0.0040	-0.0003	0.0002
rs1801689	17	64210580	C	A	0.0301	0.0908	0.0107	-0.0002	0.0005
rs216191	17	2188639	T	C	0.6474	0.0370	0.0038	0.0000	0.0002
rs516051	17	27882705	C	A	0.5172	-0.0648	0.0037	0.0001	0.0002
rs7221186	17	4813685	G	C	0.1383	0.0363	0.0053	-0.0001	0.0002
rs850729	17	42454806	T	C	0.3738	-0.0346	0.0038	-0.0002	0.0002
rs860335	17	19837679	G	A	0.3969	0.0243	0.0037	0.0002	0.0002
rs11082304	18	20720973	T	G	0.5126	-0.0507	0.0036	0.0002	0.0002
rs12458093	18	48679150	G	T	0.4794	0.0229	0.0038	0.0003	0.0002
rs16977972	18	41980501	T	G	0.1445	0.0449	0.0055	0.0000	0.0002
rs17758695	18	60920854	T	C	0.0302	-0.0691	0.0107	0.0000	0.0005
rs9952970	18	9620505	C	T	0.7397	0.0236	0.0042	0.0003	0.0002
rs12459847	19	45751157	C	G	0.2557	-0.0516	0.0042	0.0000	0.0002
rs12608697	19	38765660	A	C	0.4059	0.0305	0.0037	0.0000	0.0002
rs12721051	19	45422160	G	C	0.1838	-0.0275	0.0047	-0.0002	0.0002
rs12976598	19	39229959	A	G	0.0644	0.0512	0.0075	0.0001	0.0003
rs1697553	19	51727322	G	A	0.426	0.0214	0.0037	-0.0002	0.0002

rs2288419	19	55693244	C	T	0.1936	-0.0256	0.0046	-0.0003	0.0002
rs34536443	19	10463118	C	G	0.0479	-0.0614	0.0086	-0.0003	0.0004
rs34847357	19	2003261	A	G	0.1667	0.0286	0.0049	0.0000	0.0002
rs59508494	19	16211630	G	A	0.0192	-0.2302	0.0140	0.0001	0.0006
rs7249692	19	19670688	C	T	0.6722	-0.0285	0.0039	0.0001	0.0002
rs8107037	19	10727626	T	C	0.7352	-0.0245	0.0042	0.0000	0.0002
rs16979901	20	54988877	G	A	0.1026	0.0421	0.0060	-0.0001	0.0003
rs4411786	20	1930897	C	T	0.2634	-0.0459	0.0041	-0.0002	0.0002
rs4432538	20	8607393	A	G	0.5077	-0.0242	0.0037	0.0000	0.0002
rs6010986	20	62275844	C	T	0.7684	0.0243	0.0043	0.0002	0.0002
rs6060976	20	30414942	A	G	0.2778	-0.0344	0.0041	-0.0002	0.0002
rs6070697	20	57599402	A	G	0.1833	0.0505	0.0047	-0.0001	0.0002
rs76171326	20	25194611	A	G	0.0166	0.1102	0.0148	0.0010	0.0007
rs80054178	20	30294682	C	T	0.0226	0.1248	0.0123	0.0010	0.0006
rs147412694	21	40702786	A	G	0.1499	-0.0362	0.0052	0.0004	0.0002
rs2836441	21	39870310	A	G	0.8517	-0.0435	0.0052	-0.0001	0.0002
rs9636888	21	36367434	T	C	0.623	0.0217	0.0039	0.0001	0.0002
rs12158292	22	29633234	C	T	0.5643	-0.0208	0.0037	0.0000	0.0002
rs1474745	22	44349236	C	T	0.1531	-0.0316	0.0051	0.0003	0.0002
rs2283847	22	28181399	T	C	0.5548	-0.0255	0.0038	0.0000	0.0002
rs75107793	22	50628937	A	G	0.0725	0.1157	0.0071	0.0001	0.0003
rs8137128	22	43385996	T	A	0.5617	-0.0418	0.0037	0.0000	0.0002
rs855791	22	37462936	G	A	0.561	-0.0321	0.0037	0.0000	0.0002
