# Supplementary material for: Blood cell traits and risk of glaucoma: A two-sample mendelian randomization study
Source: Front Genet. 2023 Apr 12;14:1142773. doi: 10.3389/fgene.2023.1142773 (PMC10130872; doi:10.3389/fgene.2023.1142773)
Supplement: Supplementary file 1 [file DataSheet1.ZIP › eTable 7. Plateletcrit exposure SNPs and their association with glaucoma.pdf]

eTable 7. Plateletcrit exposure SNPs and their association with glaucoma.
Chr = chromosome; POS = position ; EA = effect allele; NEA = non-effect allele; EAF = effect allele frequency; SE = standard error.

SNP	Chr	POS	EA	NEA	EAF	Plateletcrit		Glaucoma	
						Beta	SE	Beta	SE
rs10908505	1	156468243	A	T	0.6508	-0.0296	0.0038	0.0001	0.0002
rs114982150	1	199178460	C	T	0.0214	-0.0785	0.0132	0.0004	0.0006
rs12040949	1	150447462	T	C	0.3835	0.0237	0.0037	0.0001	0.0002
rs12145626	1	153645548	T	G	0.4517	0.0203	0.0037	0.0000	0.0002
rs1538970	1	45847562	A	G	0.2328	-0.0339	0.0044	-0.0001	0.0002
rs1539019	1	247600301	C	A	0.6181	0.0250	0.0038	-0.0003	0.0002
rs1569419	1	2996602	C	T	0.7644	0.0439	0.0044	-0.0001	0.0002
rs1758186	1	156758864	T	C	0.2569	-0.0293	0.0042	0.0002	0.0002
rs182050989	1	27262545	T	C	0.0292	0.0772	0.0110	0.0000	0.0005
rs1826164	1	88104648	G	A	0.0567	-0.0525	0.0079	-0.0004	0.0004
rs200713481	1	204283964	C	T	0.2569	-0.0284	0.0042	-0.0003	0.0002
rs2071412	1	8928125	T	G	0.2681	0.0226	0.0041	0.0003	0.0002
rs2932536	1	113202417	A	G	0.5259	-0.0253	0.0036	0.0000	0.0002
rs55794721	1	25561667	A	G	0.4119	-0.0474	0.0037	-0.0001	0.0002
rs56043070	1	247719769	A	G	0.0712	-0.0975	0.0071	0.0000	0.0003
rs6667939	1	198994619	T	C	0.7193	0.0512	0.0041	0.0003	0.0002
rs6696074	1	225962690	T	C	0.5651	0.0224	0.0037	-0.0001	0.0002
rs71512947	1	19749550	G	C	0.3455	0.0254	0.0038	0.0002	0.0002
rs72654647	1	25022314	A	G	0.2460	-0.0315	0.0043	-0.0004	0.0002
rs75139539	1	43840982	T	C	0.0322	0.0626	0.0104	0.0002	0.0005
rs7535217	1	66166601	T	A	0.6031	0.0207	0.0038	-0.0001	0.0002
rs7538358	1	12033512	G	A	0.4677	-0.0484	0.0037	0.0000	0.0002
rs78265569	1	2146165	A	C	0.0894	-0.0404	0.0065	0.0003	0.0003
rs945631	1	93426167	A	G	0.0419	-0.0535	0.0093	-0.0004	0.0004
rs10048745	2	68962137	A	G	0.2596	0.0402	0.0043	0.0000	0.0002
rs113542380	2	43464818	A	G	0.0760	-0.0825	0.0070	-0.0001	0.0003
rs11681227	2	100754997	G	A	0.3600	0.0233	0.0038	0.0000	0.0002
rs11684299	2	227296995	T	C	0.1941	0.0425	0.0046	0.0002	0.0002
rs12052715	2	160677375	G	C	0.7248	-0.0503	0.0041	0.0000	0.0002
rs1260326	2	27730940	C	T	0.6049	-0.0356	0.0037	0.0000	0.0002
rs2113814	2	12951752	T	C	0.4772	0.0210	0.0037	0.0000	0.0002
rs28665899	2	219123043	A	T	0.5514	-0.0232	0.0037	0.0000	0.0002
rs56414520	2	120553107	C	T	0.2604	0.0246	0.0042	0.0002	0.0002
rs60485593	2	234279261	C	G	0.1196	-0.0392	0.0057	-0.0003	0.0003
rs62160676	2	112167931	C	T	0.2975	0.0347	0.0040	-0.0002	0.0002
rs655029	2	31477838	A	G	0.7118	0.0361	0.0041	-0.0001	0.0002
rs6712091	2	202169132	C	T	0.6432	0.0231	0.0038	0.0002	0.0002
rs715	2	211543055	C	T	0.3126	-0.0241	0.0040	0.0002	0.0002
rs7567043	2	23899095	C	T	0.1925	-0.0288	0.0046	0.0001	0.0002
rs76840964	2	160754947	G	A	0.1249	-0.0345	0.0055	0.0001	0.0002
rs1346272	3	107229242	T	C	0.3117	0.0228	0.0040	-0.0002	0.0002
rs1354034	3	56849749	C	T	0.6034	0.0354	0.0037	0.0003	0.0002
rs16858720	3	184066615	G	A	0.1263	0.0452	0.0057	0.0003	0.0003
rs2089979	3	196501413	G	A	0.4183	-0.0330	0.0037	0.0000	0.0002
rs2901381	3	168845418	G	T	0.6060	-0.0379	0.0038	0.0000	0.0002
rs332507	3	124435110	C	T	0.2229	-0.0301	0.0044	-0.0001	0.0002
rs3804749	3	122833003	T	C	0.5927	-0.0245	0.0037	0.0004	0.0002
rs4686388	3	185484257	A	G	0.6347	-0.0232	0.0038	-0.0001	0.0002
rs55983424	3	183748913	C	T	0.5473	0.0280	0.0037	0.0000	0.0002
rs62259323	3	58291047	G	A	0.3218	-0.0222	0.0039	0.0000	0.0002
rs648103	3	27550301	C	T	0.6356	0.0218	0.0038	0.0000	0.0002
rs7427439	3	18318129	G	A	0.7431	0.0303	0.0042	-0.0003	0.0002

rs78565404	3	184090242	T	C	0.0538	0.1550	0.0085	0.0002	0.0004
rs9809116	3	72397279	G	A	0.4078	0.0229	0.0037	0.0001	0.0002
rs9810259	3	12268191	G	C	0.4212	-0.0446	0.0037	0.0000	0.0002
rs10004764	4	7041377	G	A	0.5716	0.0271	0.0037	-0.0003	0.0002
rs11723550	4	120289961	C	T	0.4730	-0.0226	0.0037	0.0001	0.0002
rs13144869	4	141851432	C	A	0.5009	-0.0279	0.0037	0.0001	0.0002
rs218265	4	55408999	C	T	0.1571	0.0320	0.0051	0.0001	0.0002
rs2298993	4	110895113	A	G	0.4033	-0.0323	0.0037	-0.0003	0.0002
rs2724564	4	152349962	T	G	0.5581	0.0234	0.0037	-0.0001	0.0002
rs28636836	4	88231865	T	C	0.2717	0.0235	0.0042	-0.0002	0.0002
rs4240356	4	145042250	G	C	0.5503	-0.0252	0.0037	-0.0002	0.0002
rs58530613	4	124727524	C	T	0.1119	0.0322	0.0059	0.0003	0.0003
rs60786079	4	106197750	A	G	0.0307	-0.0670	0.0106	0.0006	0.0005
rs7685731	4	185784169	T	G	0.1922	-0.0270	0.0047	-0.0001	0.0002
rs79389047	4	157678774	T	C	0.0920	-0.0437	0.0063	0.0000	0.0003
rs10514301	5	87939654	T	C	0.1179	0.0372	0.0057	0.0001	0.0003
rs10940473	5	54873285	T	C	0.6905	-0.0224	0.0040	-0.0002	0.0002
rs114694170	5	88180196	C	T	0.0592	0.1111	0.0079	0.0002	0.0003
rs1968382	5	77721404	C	A	0.2948	-0.0386	0.0040	0.0001	0.0002
rs2070724	5	131822072	G	A	0.3323	0.0267	0.0039	0.0003	0.0002
rs27740	5	122204772	T	A	0.5551	0.0271	0.0037	0.0001	0.0002
rs457648	5	34658419	T	C	0.4625	0.0208	0.0037	-0.0001	0.0002
rs56084922	5	111061883	G	A	0.0769	-0.0785	0.0069	0.0000	0.0003
rs6556471	5	159598264	C	T	0.6773	-0.0271	0.0039	-0.0002	0.0002
rs6860138	5	141509985	A	G	0.6165	0.0418	0.0038	-0.0005	0.0002
rs6878680	5	156387137	C	G	0.6370	-0.0224	0.0038	0.0000	0.0002
rs6897795	5	177606419	A	G	0.1137	-0.0483	0.0059	-0.0003	0.0003
rs7705526	5	1285974	A	C	0.3288	0.0537	0.0040	0.0000	0.0002
rs116234817	6	31321364	A	G	0.0518	0.0904	0.0082	0.0006	0.0004
rs12526480	6	25533534	G	T	0.3382	-0.0342	0.0039	-0.0001	0.0002
rs210142	6	33546837	C	T	0.7016	0.1114	0.0040	0.0000	0.0002
rs210798	6	135514558	G	T	0.5204	-0.0258	0.0037	-0.0002	0.0002
rs2894802	6	52656169	G	T	0.5733	-0.0300	0.0037	-0.0002	0.0002
rs3132520	6	31140008	T	C	0.5029	-0.0281	0.0037	0.0000	0.0002
rs4709819	6	164463355	A	G	0.4063	0.0421	0.0037	0.0003	0.0002
rs487358	6	33570932	A	G	0.4522	0.0337	0.0037	0.0000	0.0002
rs61025394	6	7085817	A	G	0.2118	-0.0266	0.0045	0.0003	0.0002
rs6910696	6	109592329	T	A	0.4782	0.0273	0.0037	0.0002	0.0002
rs6919440	6	43352898	A	G	0.5671	0.0218	0.0037	0.0002	0.0002
rs6923682	6	34603346	A	G	0.2300	-0.0310	0.0045	0.0002	0.0002
rs6926219	6	122720806	A	G	0.5471	0.0231	0.0037	0.0001	0.0002
rs73735248	6	21809195	T	C	0.1587	0.0301	0.0050	0.0000	0.0002
rs78399616	6	33635565	A	C	0.0146	0.0942	0.0167	-0.0012	0.0009
rs9376060	6	135052237	G	A	0.2191	0.0438	0.0044	0.0000	0.0002
rs9399136	6	135402339	C	T	0.2571	0.1345	0.0042	0.0001	0.0002
rs1019068	7	18200463	C	A	0.2118	-0.0343	0.0045	-0.0001	0.0002
rs1182196	7	2866478	G	T	0.4249	0.0281	0.0037	-0.0002	0.0002
rs12669378	7	130758576	T	C	0.7355	-0.0301	0.0041	0.0000	0.0002
rs141721165	7	129254056	G	A	0.0888	0.0356	0.0065	0.0000	0.0003
rs2700937	7	36085061	T	A	0.4626	0.0211	0.0037	0.0002	0.0002
rs35150201	7	135346262	G	T	0.4835	-0.0281	0.0037	0.0002	0.0002
rs41785	7	116486020	A	C	0.4172	0.0241	0.0037	0.0000	0.0002
rs4947490	7	55160538	G	A	0.6798	0.0215	0.0039	-0.0001	0.0002
rs68116612	7	100006493	A	G	0.1948	0.0333	0.0046	0.0004	0.0002
rs714543	7	44887076	G	A	0.4346	-0.0549	0.0037	-0.0002	0.0002
rs725860	7	123424709	C	T	0.0800	0.0597	0.0068	0.0003	0.0003
rs75612655	7	101594247	T	C	0.0085	0.1172	0.0202	-0.0016	0.0009
rs13274496	8	22440898	A	G	0.2238	0.0253	0.0044	0.0005	0.0002

rs2979489	8	30280833	A	G	0.7422	0.0314	0.0042	0.0000	0.0002
rs3135109	8	145001784	G	A	0.4239	0.0324	0.0037	0.0002	0.0002
rs4961345	8	142280765	T	C	0.2763	-0.0260	0.0041	-0.0001	0.0002
rs6472235	8	66822030	G	T	0.3457	-0.0265	0.0038	-0.0002	0.0002
rs6993770	8	106581528	T	A	0.2854	-0.0534	0.0040	0.0001	0.0002
rs7829132	8	128833477	A	G	0.5879	-0.0213	0.0037	0.0004	0.0002
rs10116352	9	4939466	C	G	0.8994	0.0374	0.0062	-0.0005	0.0003
rs11794772	9	99118445	A	G	0.2593	0.0365	0.0042	-0.0003	0.0002
rs12343705	9	38197187	T	A	0.4893	-0.0301	0.0037	-0.0002	0.0002
rs2518721	9	21979204	A	G	0.8853	0.0435	0.0058	-0.0003	0.0003
rs296852	9	4787167	A	G	0.6323	-0.0489	0.0039	0.0000	0.0002
rs3758253	9	100700654	T	C	0.3633	0.0372	0.0038	-0.0002	0.0002
rs385893	9	4763176	C	T	0.5223	0.1204	0.0037	0.0001	0.0002
rs4587410	9	91394827	C	T	0.0637	-0.0929	0.0076	0.0002	0.0003
rs467317	9	136907108	A	G	0.3540	0.0210	0.0038	0.0002	0.0002
rs532861	9	136171344	T	C	0.3848	0.0207	0.0038	0.0000	0.0002
rs6475611	9	22151139	A	G	0.1619	-0.0633	0.0050	0.0001	0.0002
rs7033052	9	5042981	C	G	0.5296	-0.0441	0.0037	-0.0001	0.0002
rs7034359	9	135875618	C	T	0.4432	0.0206	0.0037	-0.0002	0.0002
rs7860776	9	4836566	A	G	0.2927	-0.0384	0.0042	-0.0004	0.0002
rs9697210	9	131468740	A	G	0.1454	-0.0339	0.0052	0.0002	0.0002
rs10821952	10	63818425	A	T	0.5667	-0.0229	0.0037	-0.0002	0.0002
rs11187838	10	96038686	A	G	0.4348	-0.0250	0.0037	0.0000	0.0002
rs11190133	10	101278725	T	C	0.2842	-0.0253	0.0040	-0.0003	0.0002
rs116052829	10	81164146	T	C	0.1049	0.0385	0.0060	-0.0004	0.0003
rs12266014	10	25211291	T	C	0.3690	-0.0271	0.0038	0.0003	0.0002
rs4272720	10	50263201	G	A	0.2362	-0.0388	0.0043	-0.0003	0.0002
rs10769960	11	8819003	C	T	0.4367	-0.0299	0.0037	0.0001	0.0002
rs10893909	11	128565034	T	C	0.2522	-0.0327	0.0042	-0.0001	0.0002
rs1111890	11	100501571	G	C	0.3655	0.0279	0.0038	0.0000	0.0002
rs11235688	11	72947934	A	G	0.4175	-0.0231	0.0037	0.0003	0.0002
rs11604127	11	196944	T	C	0.2361	0.0441	0.0043	0.0001	0.0002
rs12223381	11	108354102	T	C	0.4089	0.0400	0.0037	-0.0001	0.0002
rs174594	11	61619829	A	C	0.6242	-0.0231	0.0038	0.0000	0.0002
rs2239897	11	2891965	G	A	0.0637	-0.0467	0.0079	-0.0006	0.0004
rs231353	11	2709019	G	A	0.3493	-0.0231	0.0038	-0.0002	0.0002
rs45535039	11	119060963	C	T	0.2722	0.0658	0.0041	0.0000	0.0002
rs4937333	11	128330520	C	T	0.5265	-0.0313	0.0037	0.0000	0.0002
rs587080	11	65253800	C	A	0.3356	-0.0247	0.0039	-0.0003	0.0002
rs645901	11	116702362	C	T	0.8657	0.0346	0.0054	0.0002	0.0002
rs73000929	11	113953622	A	G	0.0373	-0.0597	0.0098	-0.0005	0.0004
rs7950696	11	47481533	C	T	0.4452	0.0260	0.0037	0.0001	0.0002
rs113825134	12	78220740	A	G	0.2336	-0.0261	0.0043	0.0003	0.0002
rs11553699	12	122216910	G	A	0.1352	0.1085	0.0057	0.0004	0.0002
rs11614212	12	50900106	C	T	0.2482	-0.0257	0.0042	-0.0001	0.0002
rs1716182	12	123686127	C	A	0.7883	0.0315	0.0045	0.0003	0.0002
rs2255531	12	121414915	A	G	0.3495	-0.0312	0.0038	0.0003	0.0002
rs2277339	12	57146069	G	T	0.1038	0.0385	0.0060	0.0001	0.0003
rs2284344	12	6445329	C	G	0.5180	-0.0305	0.0037	0.0002	0.0002
rs34038797	12	740009	G	C	0.4857	-0.0246	0.0038	0.0001	0.0002
rs34922454	12	48192062	C	A	0.3039	0.0296	0.0040	-0.0001	0.0002
rs4388979	12	109475012	T	G	0.5834	-0.0492	0.0037	-0.0001	0.0002
rs7310615	12	111865049	G	C	0.5161	-0.1136	0.0037	0.0002	0.0002
rs73135275	12	62683167	C	T	0.1064	-0.0356	0.0059	-0.0006	0.0003
rs78691875	12	113070264	A	C	0.0219	-0.0918	0.0125	-0.0009	0.0006
rs78719031	12	46997111	A	C	0.0712	0.0390	0.0071	0.0000	0.0003
rs7954567	12	6491125	A	G	0.3520	-0.0222	0.0039	0.0000	0.0002
rs79755767	12	54698408	A	G	0.1013	0.0706	0.0062	-0.0001	0.0003

rs11617297	13	41494023	G	T	0.6801	0.0230	0.0039	-0.0004	0.0002
rs11841319	13	110492626	T	C	0.1027	-0.0595	0.0061	-0.0002	0.0003
rs374039502	13	108960385	A	T	0.0210	-0.0939	0.0137	0.0001	0.0006
rs4773860	13	95901241	T	C	0.5186	0.0423	0.0037	0.0002	0.0002
rs670180	13	71236611	A	T	0.5723	0.0379	0.0037	0.0002	0.0002
rs9572786	13	72342605	G	A	0.3775	-0.0252	0.0038	0.0002	0.0002
rs11158588	14	65799876	A	G	0.7872	0.0312	0.0045	0.0002	0.0002
rs112560164	14	93112924	A	G	0.1912	-0.0354	0.0047	-0.0001	0.0002
rs113599586	14	68435810	T	C	0.1735	-0.0422	0.0048	0.0000	0.0002
rs12884793	14	103095785	A	C	0.6899	0.0414	0.0040	0.0000	0.0002
rs1555405	14	101176769	A	G	0.2489	-0.0664	0.0042	0.0000	0.0002
rs2180369	14	93516465	C	T	0.1107	0.0412	0.0058	0.0003	0.0003
rs3844535	14	81884515	G	A	0.6999	0.0239	0.0040	0.0000	0.0002
rs8012145	14	55172429	T	C	0.7325	0.0246	0.0041	0.0001	0.0002
rs929579	14	75330697	T	C	0.3296	0.0215	0.0039	-0.0001	0.0002
rs11071720	15	63341996	C	T	0.7002	0.0229	0.0040	0.0002	0.0002
rs11072748	15	78542971	T	A	0.6269	-0.0207	0.0038	0.0001	0.0002
rs11856829	15	39277781	T	C	0.5131	0.0268	0.0039	0.0000	0.0002
rs4965426	15	99248041	A	G	0.1428	-0.0354	0.0052	-0.0005	0.0002
rs55707100	15	43820717	T	C	0.0261	0.1223	0.0115	0.0006	0.0005
rs62027291	15	77266051	A	G	0.1628	-0.0292	0.0050	-0.0001	0.0002
rs4334315	16	79756197	T	A	0.3052	0.0241	0.0041	-0.0001	0.0002
rs4783187	16	85415838	C	T	0.8779	-0.0356	0.0056	0.0000	0.0002
rs59865663	16	88558312	A	G	0.2043	0.0419	0.0046	0.0001	0.0002
rs8050260	16	68551277	A	T	0.2093	0.0273	0.0046	-0.0002	0.0002
rs9923283	16	29661294	T	C	0.0634	0.0586	0.0075	-0.0002	0.0003
rs12451471	17	78102517	T	C	0.3743	-0.0223	0.0038	0.0002	0.0002
rs12943566	17	2157774	G	A	0.6526	0.0459	0.0038	0.0000	0.0002
rs12946564	17	1963961	A	G	0.7301	-0.0259	0.0041	0.0002	0.0002
rs147635058	17	16235031	A	C	0.0125	-0.1164	0.0205	-0.0014	0.0009
rs150568286	17	42594755	A	G	0.0126	-0.1148	0.0167	-0.0004	0.0007
rs1801689	17	64210580	C	A	0.0301	0.1148	0.0107	-0.0002	0.0005
rs188761458	17	55463857	T	C	0.0488	-0.0909	0.0086	-0.0002	0.0004
rs2034309	17	72688065	T	C	0.7844	0.0269	0.0045	0.0001	0.0002
rs2526377	17	56410041	G	A	0.4428	0.0237	0.0037	-0.0001	0.0002
rs2748424	17	76124865	G	C	0.1894	-0.0326	0.0047	0.0004	0.0002
rs34121753	17	7733833	G	A	0.5768	-0.0207	0.0037	-0.0003	0.0002
rs7503168	17	33885904	G	A	0.1734	0.0406	0.0048	0.0001	0.0002
rs865483	17	35851177	C	A	0.6416	-0.0249	0.0038	0.0000	0.0002
rs11082304	18	20720973	T	G	0.5125	-0.0351	0.0037	0.0002	0.0002
rs11082383	18	42014479	C	T	0.1321	0.0729	0.0055	0.0000	0.0002
rs1124980	18	67548627	A	G	0.6087	0.0320	0.0037	-0.0003	0.0002
rs12458093	18	48679150	G	T	0.4794	0.0224	0.0038	0.0003	0.0002
rs17758236	18	41515925	C	T	0.0706	0.0422	0.0072	-0.0002	0.0003
rs17758695	18	60920854	T	C	0.0303	-0.1239	0.0107	0.0000	0.0005
rs12459847	19	45751157	C	G	0.2555	-0.0452	0.0042	0.0000	0.0002
rs12462978	19	10690113	T	A	0.3803	-0.0305	0.0038	-0.0001	0.0002
rs2288419	19	55693244	C	T	0.1935	-0.0267	0.0046	-0.0003	0.0002
rs34912569	19	15371482	A	G	0.1698	0.0275	0.0049	0.0003	0.0002
rs36125859	19	39233969	G	T	0.0636	0.0434	0.0078	-0.0001	0.0004
rs3865444	19	51727962	A	C	0.3242	-0.0251	0.0039	0.0003	0.0002
rs4420638	19	45422946	G	A	0.1883	-0.0381	0.0047	-0.0002	0.0002
rs4808579	19	17255734	C	T	0.3897	0.0279	0.0038	0.0001	0.0002
rs564726571	19	19376215	T	C	0.1366	0.0428	0.0055	0.0000	0.0002
rs58434384	19	19786099	G	A	0.0832	0.0690	0.0066	0.0000	0.0003
rs7253820	19	38726076	G	A	0.4678	0.0256	0.0037	0.0001	0.0002
rs156355	20	1818613	C	T	0.4630	0.0414	0.0038	0.0001	0.0002
rs16979901	20	54988877	G	A	0.1027	0.0433	0.0060	-0.0001	0.0003

rs2243423	20	1559989	T	G	0.6919	0.0235	0.0041	0.0000	0.0002
rs34524896	20	57594684	T	C	0.0477	-0.1421	0.0086	0.0000	0.0004
rs3787096	20	62308158	G	C	0.5597	0.0251	0.0038	0.0002	0.0002
rs4432538	20	8607393	A	G	0.5078	-0.0378	0.0037	0.0000	0.0002
rs4814779	20	1923271	A	C	0.2670	-0.0864	0.0041	-0.0001	0.0002
rs6060976	20	30414942	A	G	0.2779	-0.0393	0.0042	-0.0002	0.0002
rs76171326	20	25194611	A	G	0.0166	0.1254	0.0149	0.0010	0.0007
rs80054178	20	30294682	C	T	0.0226	0.1452	0.0123	0.0010	0.0006
rs2836441	21	39870310	A	G	0.8519	-0.0573	0.0052	-0.0001	0.0002
rs75967349	21	36400441	G	C	0.0499	-0.0546	0.0085	-0.0004	0.0004
rs11704518	22	29638470	A	G	0.3706	-0.0217	0.0038	0.0001	0.0002
rs382819	22	28181436	C	A	0.7172	-0.0329	0.0041	0.0000	0.0002
rs738408	22	44324730	T	C	0.2156	-0.0290	0.0044	0.0001	0.0002
rs75107793	22	50628937	A	G	0.0726	0.1121	0.0071	0.0001	0.0003
rs855791	22	37462936	G	A	0.5610	-0.0336	0.0037	0.0000	0.0002
rs9605049	22	20011159	T	C	0.2652	0.0286	0.0041	0.0000	0.0002
