# Supplementary material for: Blood cell traits and risk of glaucoma: A two-sample mendelian randomization study
Source: Front Genet. 2023 Apr 12;14:1142773. doi: 10.3389/fgene.2023.1142773 (PMC10130872; doi:10.3389/fgene.2023.1142773)
Supplement: Supplementary file 1 [file DataSheet1.ZIP › eTable 6. White blood cell count exposure SNPs and their association with glaucoma.pdf]

eTable 6. White blood cell count exposure SNPs and their association with glaucoma.
Chr = chromosome; POS = position ; EA = effect allele; NEA = non-effect allele; EAF = effect allele frequency; SE = standard error.

SNP	Chr	POS	EA	NEA	EAF	White Blood Cell Count		Glaucoma	
						Beta	SE	Beta	SE
rs10494783	1	198663661	A	G	0.0521	-0.0438	0.0041	-0.0003	0.0004
rs10864368	1	8918313	C	T	0.5028	0.0215	0.0018	0.0001	0.0002
rs10889574	1	66149341	A	G	0.3566	-0.0437	0.0020	0.0001	0.0002
rs10919231	1	169721166	T	C	0.1907	0.0146	0.0023	-0.0004	0.0002
rs111449026	1	158557592	T	C	0.0552	0.0231	0.0042	0.0000	0.0004
rs11204662	1	150514741	G	T	0.6081	0.0186	0.0019	0.0001	0.0002
rs11208559	1	65435283	G	C	0.2585	0.0129	0.0022	-0.0001	0.0002
rs113644382	1	43430349	A	G	0.2691	-0.0142	0.0021	-0.0003	0.0002
rs11547648	1	101106268	T	G	0.4645	-0.0148	0.0018	0.0000	0.0002
rs11579633	1	167600842	T	C	0.4219	0.0162	0.0019	-0.0001	0.0002
rs12138789	1	9192307	C	T	0.2377	-0.0146	0.0022	-0.0002	0.0002
rs12142474	1	68056314	G	A	0.0861	-0.0216	0.0034	0.0005	0.0003
rs12145789	1	56607069	T	C	0.3516	0.0144	0.0019	0.0000	0.0002
rs13303224	1	35291225	T	C	0.2315	0.0145	0.0022	-0.0003	0.0002
rs139795227	1	92842367	C	A	0.0153	0.0685	0.0079	0.0006	0.0007
rs1539009	1	213953479	T	C	0.6039	-0.0147	0.0019	-0.0001	0.0002
rs1543314	1	166844024	A	C	0.8104	-0.0161	0.0023	0.0001	0.0002
rs1779809	1	182145545	T	C	0.3337	0.0144	0.0019	-0.0001	0.0002
rs1886654	1	236105910	C	T	0.8922	-0.0575	0.0030	0.0003	0.0003
rs1933295	1	62107021	G	A	0.7768	-0.0155	0.0023	0.0002	0.0002
rs1953012	1	227206038	C	T	0.6366	-0.0178	0.0019	0.0003	0.0002
rs2104415	1	67369398	A	G	0.6762	-0.0124	0.0021	-0.0001	0.0002
rs2476601	1	114377568	G	A	0.8998	0.0528	0.0030	0.0001	0.0003
rs2501309	1	22341714	C	T	0.6536	-0.0154	0.0019	0.0000	0.0002
rs2615061	1	225895806	A	G	0.1175	-0.0276	0.0029	0.0005	0.0003
rs2790122	1	200296339	C	T	0.7124	0.0134	0.0020	-0.0002	0.0002
rs284317	1	10731625	G	A	0.4976	0.0174	0.0019	0.0001	0.0002
rs301819	1	8501786	G	A	0.5826	-0.0158	0.0019	0.0001	0.0002
rs3134615	1	40362066	A	C	0.2494	0.0124	0.0021	-0.0001	0.0002
rs34298354	1	247588053	T	C	0.1235	-0.0242	0.0028	-0.0001	0.0003
rs34599082	1	159175494	T	C	0.0134	-0.1515	0.0081	0.0005	0.0007
rs35571080	1	224637790	C	T	0.2127	0.0249	0.0022	-0.0002	0.0002
rs3762297	1	31231680	T	C	0.1833	0.0248	0.0024	0.0000	0.0002
rs3795503	1	180905694	T	C	0.3146	-0.0154	0.0020	0.0000	0.0002
rs3917932	1	36943916	G	C	0.5775	-0.0448	0.0019	0.0000	0.0002
rs41272536	1	183440531	G	A	0.0457	-0.0344	0.0047	-0.0001	0.0004
rs41313381	1	79411968	A	C	0.0304	0.0485	0.0055	-0.0001	0.0005
rs4443935	1	26875433	G	A	0.7236	-0.0124	0.0021	0.0000	0.0002
rs4626924	1	234909298	T	C	0.5510	0.0168	0.0019	0.0000	0.0002
rs4844390	1	207934849	G	A	0.2202	-0.0265	0.0022	0.0000	0.0002
rs4925756	1	248048016	T	C	0.6919	-0.0146	0.0020	0.0002	0.0002
rs501791	1	156089873	T	C	0.0454	-0.0335	0.0044	0.0000	0.0004
rs533483	1	234765256	A	G	0.2434	-0.0174	0.0022	0.0003	0.0002
rs547364	1	108742123	G	C	0.4631	0.0164	0.0018	-0.0001	0.0002
rs56188865	1	247606276	C	T	0.3742	-0.0270	0.0019	0.0003	0.0002
rs57287386	1	42202114	A	G	0.3523	-0.0146	0.0019	0.0001	0.0002
rs60124939	1	174075302	T	C	0.1768	-0.0165	0.0024	0.0003	0.0002
rs60314236	1	27155166	T	C	0.1623	-0.0195	0.0025	0.0001	0.0002
rs630505	1	111737916	C	T	0.2654	0.0125	0.0021	-0.0002	0.0002
rs6429582	1	46321843	C	T	0.4351	-0.0188	0.0019	0.0002	0.0002
rs6684709	1	23850023	C	G	0.6205	-0.0206	0.0019	-0.0004	0.0002
rs6690335	1	39291708	G	A	0.2264	0.0195	0.0022	0.0003	0.0002

rs6696259	1	101221482	C	G	0.4089	0.0181	0.0019	0.0002	0.0002
rs7537229	1	56906274	A	G	0.8963	0.0274	0.0030	0.0001	0.0003
rs7538081	1	101592299	C	T	0.2342	-0.0130	0.0022	0.0000	0.0002
rs79635379	1	91588063	T	A	0.2459	0.0137	0.0021	-0.0002	0.0002
rs896319	1	205205651	T	G	0.9112	0.0199	0.0032	-0.0003	0.0003
rs903123	1	212405792	T	C	0.8182	0.0135	0.0024	-0.0001	0.0002
rs9429767	1	110496087	A	G	0.1972	0.0174	0.0023	-0.0001	0.0002
rs9430574	1	9709072	A	G	0.3304	-0.0140	0.0020	-0.0001	0.0002
rs9701457	1	28216736	T	C	0.3658	-0.0185	0.0019	0.0000	0.0002
rs10048745	2	68962137	A	G	0.2570	0.0116	0.0021	0.0000	0.0002
rs10164769	2	237779229	T	C	0.7431	0.0272	0.0021	0.0002	0.0002
rs10203838	2	213935900	C	T	0.4179	0.0123	0.0019	0.0000	0.0002
rs10208769	2	61605614	A	T	0.5156	0.0220	0.0018	-0.0001	0.0002
rs1047891	2	211540507	A	C	0.3150	-0.0211	0.0020	0.0002	0.0002
rs10931933	2	202112471	T	G	0.6053	0.0154	0.0019	0.0002	0.0002
rs114050631	2	219020958	T	C	0.0111	-0.1382	0.0098	0.0002	0.0008
rs11676156	2	234259001	T	G	0.3945	-0.0107	0.0019	0.0000	0.0002
rs11678685	2	169710607	A	G	0.3176	0.0244	0.0020	-0.0003	0.0002
rs11688303	2	128410244	T	C	0.1203	0.0211	0.0029	-0.0001	0.0003
rs1260326	2	27730940	C	T	0.6013	-0.0329	0.0019	0.0000	0.0002
rs13388622	2	225756681	A	T	0.1566	-0.0148	0.0025	0.0000	0.0002
rs13392977	2	192514856	A	G	0.0537	0.0309	0.0041	0.0002	0.0004
rs13410405	2	106197541	G	T	0.1093	0.0191	0.0030	-0.0001	0.0003
rs1371045	2	145787050	C	T	0.7445	-0.0143	0.0021	0.0002	0.0002
rs139369470	2	26054731	C	T	0.0480	0.0253	0.0044	-0.0002	0.0004
rs141314120	2	70407205	G	C	0.0561	-0.0327	0.0040	0.0008	0.0004
rs17026212	2	85551696	G	T	0.5135	-0.0147	0.0018	-0.0007	0.0002
rs17270882	2	182218046	C	G	0.2391	0.0308	0.0022	0.0004	0.0002
rs17655123	2	220037444	A	G	0.1356	-0.0180	0.0027	0.0001	0.0002
rs1863176	2	12931092	C	G	0.4969	0.0185	0.0018	0.0000	0.0002
rs2068330	2	163237390	G	C	0.3606	0.0187	0.0019	-0.0002	0.0002
rs2729707	2	160687231	G	A	0.8301	-0.0309	0.0024	-0.0002	0.0002
rs34562738	2	102376755	A	G	0.7720	0.0187	0.0022	0.0001	0.0002
rs4422151	2	198544183	T	A	0.1947	-0.0144	0.0023	0.0000	0.0002
rs4632345	2	16702654	A	G	0.6776	-0.0137	0.0020	-0.0004	0.0002
rs4666068	2	28623982	G	A	0.5089	-0.0119	0.0018	-0.0004	0.0002
rs4672564	2	211143185	C	A	0.5009	-0.0102	0.0018	0.0004	0.0002
rs57871178	2	85425048	C	T	0.1763	-0.0133	0.0024	0.0003	0.0002
rs62172372	2	188242369	G	A	0.1994	-0.0200	0.0023	-0.0002	0.0002
rs62173240	2	160352482	A	G	0.0229	-0.0466	0.0062	-0.0007	0.0006
rs62194505	2	238864939	A	G	0.3823	-0.0109	0.0019	0.0001	0.0002
rs633323	2	31463044	T	A	0.6975	0.0147	0.0020	0.0000	0.0002
rs6715102	2	218944308	T	C	0.0793	-0.0260	0.0034	-0.0004	0.0003
rs6731993	2	65642097	T	A	0.4078	0.0225	0.0019	0.0001	0.0002
rs6734238	2	113841030	G	A	0.4022	0.0365	0.0020	-0.0001	0.0002
rs67364818	2	111613307	A	G	0.0850	0.0219	0.0035	0.0001	0.0003
rs6746368	2	161222998	T	A	0.3342	0.0115	0.0019	0.0002	0.0002
rs6755895	2	232579795	C	T	0.2318	-0.0233	0.0022	0.0002	0.0002
rs72780125	2	24000145	C	T	0.1311	0.0346	0.0028	0.0002	0.0002
rs75475627	2	54787592	G	C	0.0766	0.0328	0.0035	0.0000	0.0003
rs75685631	2	37392825	A	G	0.0335	-0.0292	0.0051	0.0001	0.0005
rs7572278	2	8563029	A	T	0.2073	0.0159	0.0023	0.0001	0.0002
rs7573465	2	182315885	T	G	0.5542	0.0358	0.0019	0.0003	0.0002
rs7579497	2	71677946	A	G	0.1855	-0.0138	0.0024	0.0000	0.0002
rs7586834	2	153282226	T	C	0.1557	-0.0213	0.0025	-0.0002	0.0002
rs796056	2	101735276	C	A	0.6235	0.0206	0.0019	0.0002	0.0002
rs79716587	2	143886819	A	G	0.1241	-0.0359	0.0028	-0.0004	0.0002
rs80350872	2	5922918	T	C	0.2656	-0.0117	0.0021	-0.0003	0.0002

rs871375	2	242421866	A	G	0.6588	0.0114	0.0020	0.0000	0.0002
rs935655	2	46067445	G	T	0.7620	0.0148	0.0022	0.0001	0.0002
rs10049210	3	156797373	C	T	0.4045	0.0147	0.0019	-0.0002	0.0002
rs10935473	3	98416900	T	G	0.4422	0.0146	0.0019	-0.0001	0.0002
rs10936588	3	169319801	A	G	0.6363	-0.0143	0.0019	-0.0001	0.0002
rs114353727	3	49089064	G	C	0.0420	0.0301	0.0047	-0.0010	0.0004
rs115433278	3	70940474	A	G	0.0531	-0.0242	0.0042	-0.0001	0.0004
rs11915472	3	132250941	G	A	0.0903	0.0259	0.0032	0.0000	0.0003
rs11927257	3	27334294	T	C	0.2822	-0.0129	0.0020	0.0001	0.0002
rs12630592	3	119768246	T	G	0.3731	-0.0123	0.0020	0.0000	0.0002
rs1366045	3	42909050	C	T	0.3843	-0.0247	0.0019	0.0001	0.0002
rs1621453	3	194389876	G	A	0.5632	0.0105	0.0019	0.0001	0.0002
rs17295246	3	123105721	A	G	0.2252	-0.0143	0.0022	0.0001	0.0002
rs1822534	3	12266804	G	A	0.3942	-0.0258	0.0019	0.0000	0.0002
rs2012610	3	140933565	T	G	0.1273	0.0275	0.0028	-0.0002	0.0002
rs2268829	3	185989567	A	G	0.7848	-0.0141	0.0022	0.0003	0.0002
rs2371108	3	27757018	T	G	0.3890	0.0192	0.0019	0.0000	0.0002
rs2713573	3	128317273	T	C	0.6892	0.0246	0.0020	0.0003	0.0002
rs35592432	3	71355240	C	G	0.0272	0.0399	0.0061	0.0008	0.0005
rs3732378	3	39307162	A	G	0.1725	0.0203	0.0024	0.0005	0.0002
rs3773312	3	12946142	A	G	0.1341	0.0179	0.0029	0.0004	0.0002
rs3792386	3	122853680	A	G	0.5847	-0.0105	0.0019	0.0004	0.0002
rs4074672	3	183730295	T	C	0.3695	0.0180	0.0019	-0.0001	0.0002
rs4234465	3	47026885	C	G	0.4227	-0.0260	0.0020	-0.0002	0.0002
rs4615073	3	64701436	T	C	0.2431	-0.0132	0.0021	0.0000	0.0002
rs58146266	3	18759764	A	G	0.1763	0.0183	0.0024	0.0001	0.0002
rs62270938	3	128187057	T	C	0.1771	0.0160	0.0025	-0.0002	0.0002
rs6440732	3	150990510	A	C	0.8277	0.0233	0.0024	-0.0003	0.0002
rs6445826	3	56848999	C	T	0.4985	0.0125	0.0019	0.0002	0.0002
rs6779340	3	58033701	G	C	0.3369	-0.0175	0.0020	0.0003	0.0002
rs73028871	3	14277058	T	C	0.1339	-0.0168	0.0027	0.0003	0.0002
rs7429516	3	136583687	T	C	0.6897	0.0155	0.0020	0.0001	0.0002
rs7639292	3	107295665	T	C	0.1685	-0.0240	0.0025	0.0005	0.0002
rs7652649	3	150707915	T	G	0.5837	0.0128	0.0020	-0.0003	0.0002
rs830623	3	71677746	C	A	0.1697	0.0145	0.0025	0.0000	0.0002
rs832190	3	63842629	T	C	0.6333	-0.0133	0.0019	0.0004	0.0002
rs9819371	3	141206800	T	C	0.0648	-0.0444	0.0038	0.0000	0.0003
rs9829114	3	196518623	A	G	0.4141	-0.0336	0.0019	0.0000	0.0002
rs9867398	3	185912816	T	C	0.0942	0.0223	0.0032	0.0000	0.0003
rs10006495	4	120263315	G	T	0.3539	-0.0157	0.0020	0.0001	0.0002
rs10027407	4	80897999	G	C	0.5303	-0.0134	0.0018	-0.0002	0.0002
rs10804990	4	6919661	A	G	0.6174	0.0185	0.0019	-0.0003	0.0002
rs10939663	4	10032516	G	T	0.2799	0.0126	0.0021	0.0000	0.0002
rs112221620	4	157703536	A	G	0.0935	-0.0186	0.0032	0.0000	0.0003
rs113473633	4	103449131	G	A	0.0255	-0.0508	0.0062	0.0000	0.0005
rs116622346	4	6973449	T	C	0.0203	-0.0384	0.0065	-0.0001	0.0006
rs11735662	4	145026126	T	C	0.0332	0.0522	0.0052	0.0000	0.0004
rs11935575	4	56019596	T	A	0.3751	0.0106	0.0019	-0.0001	0.0002
rs12643013	4	144206948	C	T	0.4995	0.0107	0.0018	0.0003	0.0002
rs13132853	4	38680015	G	A	0.3551	0.0201	0.0019	-0.0002	0.0002
rs140311179	4	26295540	G	C	0.0512	-0.0256	0.0042	0.0008	0.0004
rs144317085	4	105806108	T	A	0.0344	0.0498	0.0051	-0.0003	0.0005
rs1563645	4	83941387	G	A	0.8134	0.0140	0.0024	0.0001	0.0002
rs16846876	4	72592491	T	A	0.3294	-0.0211	0.0020	0.0001	0.0002
rs16850073	4	74703999	T	C	0.3744	0.0426	0.0019	-0.0001	0.0002
rs16850408	4	74932807	A	C	0.3685	0.0593	0.0019	0.0001	0.0002
rs17005891	4	83547862	A	G	0.1847	-0.0464	0.0024	0.0000	0.0002
rs17588356	4	151210677	T	G	0.2916	0.0168	0.0020	0.0003	0.0002

rs191180456	4	74826316	T	C	0.0100	-0.0721	0.0098	-0.0001	0.0009
rs2174326	4	90223611	G	A	0.4534	0.0118	0.0019	0.0002	0.0002
rs218264	4	55408875	T	A	0.2509	0.0258	0.0021	0.0001	0.0002
rs2412771	4	57761417	C	T	0.4167	-0.0182	0.0019	-0.0002	0.0002
rs2609279	4	89855495	C	T	0.7860	0.0157	0.0022	0.0002	0.0002
rs28530750	4	36312542	A	G	0.0430	0.0556	0.0046	-0.0006	0.0004
rs35734242	4	706700	C	T	0.4280	0.0218	0.0019	-0.0001	0.0002
rs3775967	4	110852459	G	A	0.3458	-0.0129	0.0019	-0.0004	0.0002
rs4696256	4	152291432	A	G	0.5248	0.0179	0.0018	-0.0001	0.0002
rs4998802	4	48800910	A	G	0.2794	-0.0121	0.0021	0.0000	0.0002
rs6554195	4	55500714	T	G	0.4836	-0.0195	0.0018	0.0002	0.0002
rs6844859	4	3190486	C	T	0.4225	-0.0132	0.0019	-0.0002	0.0002
rs987121	4	38329783	T	A	0.6062	0.0155	0.0019	0.0001	0.0002
rs10054235	5	173087427	C	A	0.3487	0.0129	0.0020	0.0001	0.0002
rs10940473	5	54873285	T	C	0.6917	-0.0136	0.0020	-0.0002	0.0002
rs11242109	5	131677047	T	G	0.4772	-0.0331	0.0018	-0.0004	0.0002
rs114064810	5	108167603	A	G	0.0255	0.0346	0.0061	0.0004	0.0005
rs11744663	5	57315635	A	G	0.1905	-0.0187	0.0024	-0.0001	0.0002
rs13180726	5	179126457	A	G	0.8256	-0.0213	0.0024	-0.0004	0.0002
rs1902800	5	110996178	G	C	0.3426	-0.0135	0.0019	0.0001	0.0002
rs1948760	5	156442784	A	T	0.8288	-0.0221	0.0024	0.0003	0.0002
rs2082382	5	148200553	A	G	0.5541	-0.0179	0.0019	-0.0003	0.0002
rs2432142	5	96275201	A	G	0.4337	0.0127	0.0019	-0.0001	0.0002
rs2561758	5	173205282	G	A	0.7231	-0.0363	0.0021	-0.0001	0.0002
rs2910580	5	57527886	A	T	0.6593	-0.0153	0.0019	0.0000	0.0002
rs307808	5	180069124	A	G	0.4562	-0.0107	0.0019	0.0001	0.0002
rs3749748	5	127350549	T	C	0.2459	-0.0142	0.0021	0.0001	0.0002
rs4391200	5	141509537	G	A	0.6190	0.0230	0.0019	-0.0005	0.0002
rs4535497	5	1107428	A	C	0.5700	-0.0133	0.0020	-0.0001	0.0002
rs464609	5	34654477	A	G	0.5445	0.0129	0.0019	-0.0003	0.0002
rs4703541	5	71743755	A	G	0.8788	-0.0362	0.0028	-0.0003	0.0003
rs60580948	5	148340270	G	A	0.1407	-0.0199	0.0027	0.0002	0.0002
rs62387565	5	100024839	T	C	0.4840	0.0142	0.0018	-0.0001	0.0002
rs6877725	5	68610623	C	T	0.4454	-0.0206	0.0019	-0.0002	0.0002
rs6878780	5	122093740	C	T	0.4268	0.0133	0.0019	0.0002	0.0002
rs740474	5	140925362	T	C	0.6052	0.0109	0.0019	-0.0004	0.0002
rs7705526	5	1285974	A	C	0.3272	0.0291	0.0021	0.0000	0.0002
rs79237520	5	134712566	T	C	0.0228	0.0401	0.0063	0.0000	0.0005
rs79272926	5	118726637	A	C	0.2216	-0.0223	0.0022	-0.0001	0.0002
rs888957	5	147966674	G	A	0.4995	-0.0121	0.0019	0.0001	0.0002
rs9313822	5	159207694	A	G	0.0521	0.0251	0.0041	0.0000	0.0004
rs987107	5	35875227	A	G	0.2611	0.0204	0.0021	-0.0002	0.0002
rs10945542	6	158752931	T	C	0.5203	0.0109	0.0018	0.0000	0.0002
rs10948036	6	42510305	A	C	0.2104	0.0231	0.0023	-0.0003	0.0002
rs11752200	6	130377847	C	T	0.6892	0.0158	0.0020	-0.0002	0.0002
rs12214269	6	135846518	A	G	0.5459	0.0182	0.0018	0.0003	0.0002
rs12215332	6	121788972	A	G	0.1998	0.0143	0.0023	0.0000	0.0002
rs1285886	6	7140831	A	G	0.1968	0.0265	0.0023	0.0000	0.0002
rs13207171	6	41165819	T	C	0.1328	-0.0239	0.0027	-0.0008	0.0002
rs1322599	6	16758425	T	C	0.1743	-0.0307	0.0025	0.0001	0.0002
rs1738074	6	159465977	C	T	0.5678	-0.0204	0.0019	-0.0002	0.0002
rs17710008	6	153043035	A	G	0.1805	0.0219	0.0024	-0.0004	0.0002
rs2746177	6	41639575	A	G	0.3578	-0.0115	0.0020	-0.0003	0.0002
rs28732146	6	31561353	A	T	0.1972	0.0405	0.0023	0.0000	0.0002
rs2935104	6	170491076	G	A	0.9069	0.0196	0.0033	0.0001	0.0003
rs35467127	6	32522148	T	C	0.1910	0.0704	0.0026	0.0004	0.0002
rs364663	6	105443189	A	T	0.5589	-0.0102	0.0019	0.0001	0.0002
rs3857488	6	88001064	G	A	0.5276	-0.0160	0.0018	0.0003	0.0002

rs4339503	6	31227759	T	C	0.0353	-0.0325	0.0050	0.0000	0.0005
rs4707609	6	90946479	C	T	0.3601	-0.0211	0.0019	0.0000	0.0002
rs4712614	6	21382765	G	T	0.6200	-0.0216	0.0019	-0.0002	0.0002
rs549302	6	122734972	A	G	0.5450	0.0202	0.0018	0.0001	0.0002
rs560194	6	10528794	C	T	0.5300	-0.0136	0.0018	-0.0001	0.0002
rs610604	6	138199417	T	G	0.6744	-0.0152	0.0020	-0.0001	0.0002
rs62393605	6	7275121	C	A	0.1660	-0.0153	0.0025	0.0000	0.0002
rs639728	6	44239922	T	C	0.4410	-0.0117	0.0019	0.0003	0.0002
rs6924387	6	137082948	G	A	0.4125	0.0165	0.0019	0.0000	0.0002
rs707927	6	31745518	G	A	0.0272	0.1023	0.0057	0.0001	0.0005
rs719395	6	52290927	C	T	0.4582	-0.0121	0.0018	0.0001	0.0002
rs72834627	6	26019411	C	G	0.1217	0.0210	0.0028	0.0003	0.0003
rs729761	6	43804571	G	T	0.7133	0.0150	0.0021	0.0001	0.0002
rs72992130	6	144441671	T	C	0.0453	-0.0247	0.0045	-0.0001	0.0004
rs73414461	6	29974346	C	G	0.0547	0.0275	0.0041	-0.0004	0.0004
rs73562268	6	138128078	A	G	0.0859	-0.0214	0.0033	-0.0004	0.0003
rs74679834	6	86819356	G	C	0.2771	0.0126	0.0021	-0.0001	0.0002
rs7738702	6	113413977	T	C	0.2004	-0.0144	0.0023	0.0003	0.0002
rs7770443	6	34246714	A	C	0.1444	-0.0157	0.0026	-0.0001	0.0002
rs7776054	6	135418916	G	A	0.2611	-0.0466	0.0021	0.0001	0.0002
rs9258375	6	29752808	G	A	0.1200	-0.0894	0.0029	-0.0007	0.0003
rs9366427	6	22096613	G	C	0.5536	0.0143	0.0019	0.0000	0.0002
rs9380500	6	35266231	C	G	0.8238	0.0330	0.0024	0.0001	0.0002
rs9390461	6	147701217	G	A	0.5383	0.0154	0.0018	0.0001	0.0002
rs9487043	6	109610474	T	C	0.4865	0.0231	0.0018	0.0002	0.0002
rs998584	6	43757896	A	C	0.4843	0.0158	0.0019	-0.0002	0.0002
rs10252457	7	47337530	G	A	0.4255	-0.0141	0.0019	-0.0001	0.0002
rs10260281	7	38270550	C	G	0.2370	0.0134	0.0022	0.0003	0.0002
rs10264505	7	99137874	A	C	0.0910	-0.0245	0.0032	0.0005	0.0003
rs10268450	7	80122641	A	G	0.0144	0.0427	0.0078	0.0008	0.0007
rs10273974	7	138735512	C	A	0.4914	-0.0124	0.0018	-0.0003	0.0002
rs10808139	7	104709946	A	G	0.1185	0.0176	0.0028	-0.0001	0.0003
rs12540307	7	65662767	T	C	0.0488	-0.0387	0.0045	-0.0002	0.0004
rs1474419	7	6692605	C	T	0.5798	0.0183	0.0019	-0.0003	0.0002
rs1476081	7	17279294	T	G	0.2947	0.0114	0.0021	0.0000	0.0002
rs149007767	7	50370254	T	C	0.1619	0.0152	0.0026	-0.0001	0.0002
rs17138597	7	18148091	A	G	0.1948	-0.0133	0.0023	-0.0001	0.0002
rs17449024	7	27136308	A	G	0.0723	-0.0201	0.0036	0.0002	0.0003
rs182090955	7	92239892	A	G	0.0098	-0.0758	0.0097	-0.0008	0.0009
rs2158799	7	28277107	G	C	0.6118	0.0430	0.0019	0.0001	0.0002
rs2214683	7	14019242	G	A	0.7196	-0.0148	0.0020	-0.0001	0.0002
rs2282986	7	92299545	C	T	0.0185	-0.1243	0.0069	-0.0006	0.0006
rs2710804	7	36084529	C	T	0.3759	0.0206	0.0019	0.0004	0.0002
rs34121650	7	149172966	C	A	0.6306	-0.0119	0.0019	0.0004	0.0002
rs342294	7	106372622	C	T	0.4559	-0.0174	0.0018	0.0002	0.0002
rs3731332	7	92300568	T	C	0.0232	-0.1212	0.0062	0.0005	0.0006
rs3735485	7	45009341	G	A	0.8447	0.0410	0.0026	0.0001	0.0002
rs38859	7	116379094	T	C	0.4489	0.0113	0.0019	-0.0001	0.0002
rs4729046	7	92223957	C	T	0.9241	-0.0366	0.0035	0.0000	0.0003
rs4948097	7	56056571	G	A	0.7583	-0.0176	0.0021	0.0001	0.0002
rs56388170	7	28724374	T	G	0.2945	0.0593	0.0020	0.0000	0.0002
rs60466842	7	8024840	A	C	0.0876	-0.0297	0.0033	0.0000	0.0003
rs62445884	7	21920556	A	G	0.2437	0.0127	0.0021	-0.0001	0.0002
rs62466318	7	73042085	T	C	0.2022	-0.0212	0.0024	0.0000	0.0002
rs6948695	7	129648708	C	T	0.4139	-0.0110	0.0019	0.0003	0.0002
rs6952262	7	139719439	G	C	0.2052	-0.0162	0.0023	-0.0002	0.0002
rs74607840	7	148781247	A	G	0.6259	-0.0154	0.0019	0.0001	0.0002
rs757670	7	97932200	G	T	0.9250	-0.0217	0.0036	0.0004	0.0003

rs7776857	7	22754768	T	G	0.6581	-0.0185	0.0019	0.0000	0.0002
rs7781268	7	75748183	A	G	0.2458	-0.0133	0.0023	0.0000	0.0002
rs7792934	7	2393016	G	A	0.7892	-0.0151	0.0023	-0.0001	0.0002
rs7803075	7	130742066	G	A	0.7342	-0.0244	0.0021	0.0000	0.0002
rs78234380	7	87222477	A	T	0.0400	0.0282	0.0047	0.0000	0.0004
rs798555	7	2759473	C	T	0.2950	0.0188	0.0021	0.0000	0.0002
rs9656395	7	130575884	G	A	0.0950	-0.0275	0.0032	-0.0002	0.0003
rs9656588	7	50306780	C	T	0.6715	0.0177	0.0020	0.0002	0.0002
rs10087240	8	129012574	T	C	0.4576	0.0200	0.0019	0.0001	0.0002
rs10102877	8	79598362	A	G	0.6847	-0.0132	0.0020	0.0000	0.0002
rs11250076	8	10647823	G	A	0.5747	0.0214	0.0019	-0.0001	0.0002
rs11993347	8	103919090	C	T	0.2305	-0.0197	0.0022	-0.0002	0.0002
rs12216862	8	41828063	T	C	0.2901	0.0114	0.0020	-0.0001	0.0002
rs12550612	8	22966769	A	G	0.8213	-0.0328	0.0024	-0.0001	0.0002
rs12716647	8	6901304	C	G	0.6375	0.0124	0.0019	0.0000	0.0002
rs13248936	8	30316162	G	A	0.4983	0.0131	0.0018	0.0000	0.0002
rs145209947	8	130694185	A	C	0.0140	-0.0754	0.0087	0.0000	0.0008
rs1511724	8	56796697	G	C	0.4221	-0.0172	0.0019	0.0001	0.0002
rs17209630	8	106296090	A	G	0.2813	0.0143	0.0020	0.0002	0.0002
rs1947897	8	78946874	C	G	0.3364	-0.0144	0.0019	0.0003	0.0002
rs28615248	8	55451193	C	T	0.1947	-0.0228	0.0024	-0.0002	0.0002
rs2875974	8	61419728	A	G	0.3734	0.0214	0.0019	0.0001	0.0002
rs34178379	8	66849617	G	A	0.0615	-0.0240	0.0038	-0.0001	0.0003
rs34685450	8	61916962	A	T	0.2548	-0.0137	0.0022	0.0001	0.0002
rs3802225	8	87060672	G	A	0.3685	-0.0124	0.0019	0.0002	0.0002
rs3847147	8	105784723	A	G	0.3070	-0.0119	0.0020	-0.0001	0.0002
rs4734879	8	106583124	G	A	0.2762	-0.0220	0.0021	0.0001	0.0002
rs4870937	8	126337758	T	A	0.5438	0.0143	0.0018	0.0001	0.0002
rs55964818	8	130605871	C	T	0.5673	-0.0363	0.0019	-0.0001	0.0002
rs56094005	8	21769432	G	A	0.0427	-0.0274	0.0046	-0.0001	0.0004
rs62510269	8	79012343	G	A	0.1457	-0.0361	0.0026	0.0001	0.0002
rs62511286	8	68809812	T	C	0.3980	-0.0277	0.0019	0.0001	0.0002
rs6474514	8	38835269	C	T	0.4117	0.0111	0.0019	0.0000	0.0002
rs6985508	8	142337734	A	G	0.3580	-0.0266	0.0020	0.0003	0.0002
rs7005996	8	142241681	T	C	0.9063	0.0241	0.0033	0.0003	0.0003
rs74553750	8	100749570	G	A	0.0441	0.0278	0.0045	-0.0002	0.0004
rs755951	8	27226790	C	A	0.4028	-0.0135	0.0019	-0.0001	0.0002
rs7846314	8	61650831	T	A	0.1873	0.0468	0.0024	-0.0001	0.0002
rs958416	8	19530983	T	G	0.5369	-0.0103	0.0019	0.0001	0.0002
rs10810099	9	14161927	G	A	0.7193	-0.0114	0.0021	-0.0002	0.0002
rs10814191	9	34999007	T	C	0.7134	0.0112	0.0020	-0.0001	0.0002
rs10818482	9	123648085	G	A	0.5691	0.0140	0.0019	0.0005	0.0002
rs10973700	9	38196117	C	G	0.4864	-0.0131	0.0018	-0.0002	0.0002
rs10980797	9	113912553	G	A	0.4879	-0.0270	0.0018	0.0003	0.0002
rs10986338	9	127191232	A	G	0.6498	-0.0166	0.0020	0.0001	0.0002
rs12343532	9	115978022	A	T	0.2723	-0.0152	0.0021	0.0002	0.0002
rs12376511	9	22142756	C	T	0.1644	-0.0342	0.0025	0.0002	0.0002
rs12683699	9	112738298	G	A	0.1603	0.0218	0.0025	0.0001	0.0002
rs13291664	9	282738	G	A	0.1869	0.0262	0.0024	-0.0002	0.0002
rs13296795	9	16886538	A	G	0.0384	0.0265	0.0049	0.0002	0.0004
rs1333633	9	93567572	A	G	0.5573	0.0112	0.0019	-0.0001	0.0002
rs1571142	9	130660810	G	A	0.9326	-0.0222	0.0039	-0.0001	0.0003
rs1887428	9	4984530	C	G	0.6251	-0.0150	0.0019	0.0000	0.0002
rs2148537	9	95380726	A	G	0.1663	0.0176	0.0025	0.0007	0.0002
rs2165951	9	13724282	C	A	0.6746	-0.0111	0.0020	-0.0001	0.0002
rs2506699	9	136946799	G	A	0.4839	0.0143	0.0019	-0.0001	0.0002
rs2519093	9	136141870	T	C	0.1856	-0.0412	0.0024	0.0002	0.0002
rs2583839	9	133593939	A	G	0.4018	-0.0153	0.0019	0.0002	0.0002

rs34990336	9	110273582	G	A	0.1952	0.0137	0.0024	0.0000	0.0002
rs3793537	9	35687556	C	G	0.2904	0.0198	0.0020	0.0005	0.0002
rs41317014	9	139929080	T	C	0.2476	-0.0201	0.0022	-0.0002	0.0002
rs4413892	9	139330158	A	G	0.2761	0.0311	0.0022	0.0004	0.0002
rs447124	9	4746539	T	C	0.4906	0.0170	0.0019	-0.0001	0.0002
rs626416	9	79326680	C	G	0.7629	-0.0166	0.0022	0.0001	0.0002
rs6476883	9	4654774	A	G	0.4372	-0.0115	0.0019	0.0000	0.0002
rs7036656	9	21990457	T	C	0.7215	0.0281	0.0021	-0.0002	0.0002
rs72747074	9	91545621	A	G	0.3124	0.0179	0.0020	-0.0001	0.0002
rs72759273	9	126973873	G	A	0.1948	-0.0149	0.0023	-0.0003	0.0002
rs7852409	9	2621482	G	C	0.7647	0.0156	0.0023	-0.0002	0.0002
rs7855091	9	114655098	A	G	0.3070	0.0148	0.0020	0.0000	0.0002
rs7864482	9	102268861	G	A	0.3773	0.0128	0.0019	-0.0003	0.0002
rs7873409	9	73080367	G	T	0.5843	-0.0110	0.0019	0.0000	0.0002
rs796003	9	86595801	T	G	0.2584	-0.0254	0.0021	-0.0003	0.0002
rs10761659	10	64445564	G	A	0.5394	-0.0125	0.0018	0.0000	0.0002
rs10787428	10	113935379	C	T	0.6064	0.0131	0.0019	-0.0001	0.0002
rs10828725	10	25218243	T	G	0.3648	-0.0497	0.0019	0.0003	0.0002
rs10906393	10	13536512	T	A	0.5755	0.0153	0.0019	0.0000	0.0002
rs11002309	10	79594931	T	C	0.3401	0.0127	0.0020	-0.0001	0.0002
rs11202074	10	88332371	A	G	0.2919	-0.0127	0.0020	-0.0001	0.0002
rs11252331	10	4129296	G	T	0.8366	0.0167	0.0025	-0.0007	0.0002
rs11255698	10	5920893	G	A	0.3438	0.0107	0.0019	-0.0001	0.0002
rs12784071	10	115854445	T	C	0.2221	0.0181	0.0022	0.0001	0.0002
rs1413611	10	36466891	C	T	0.3805	0.0106	0.0019	-0.0003	0.0002
rs17011726	10	50264204	G	C	0.2326	-0.0236	0.0022	-0.0003	0.0002
rs17109869	10	96000162	A	G	0.3232	-0.0135	0.0020	0.0000	0.0002
rs180941	10	115720674	A	G	0.6250	-0.0169	0.0019	0.0002	0.0002
rs1878036	10	82280137	G	T	0.7950	0.0196	0.0023	0.0001	0.0002
rs1885474	10	69566751	G	T	0.1029	-0.0216	0.0030	-0.0002	0.0003
rs2091084	10	30492267	C	T	0.6532	0.0174	0.0020	0.0000	0.0002
rs2993986	10	28800023	T	C	0.7741	0.0301	0.0022	-0.0001	0.0002
rs34179455	10	120897479	G	A	0.5683	-0.0121	0.0019	0.0000	0.0002
rs3747869	10	73520632	C	A	0.9004	0.0388	0.0032	-0.0003	0.0003
rs3781454	10	126348565	A	G	0.6768	0.0252	0.0020	-0.0003	0.0002
rs41295055	10	6111622	T	C	0.1870	-0.0137	0.0023	0.0000	0.0002
rs5030937	10	70975897	T	C	0.6791	-0.0129	0.0020	0.0000	0.0002
rs56278466	10	17875857	G	T	0.6166	-0.0142	0.0021	0.0003	0.0002
rs58452254	10	112014007	C	T	0.0716	-0.0210	0.0036	0.0004	0.0003
rs59085061	10	89681458	G	A	0.0405	-0.0503	0.0047	-0.0004	0.0004
rs61863767	10	99084426	T	C	0.5962	0.0431	0.0019	0.0001	0.0002
rs692594	10	18265893	C	G	0.4888	0.0130	0.0018	-0.0003	0.0002
rs703005	10	26758576	T	C	0.5982	-0.0155	0.0019	0.0003	0.0002
rs7082470	10	65277026	A	G	0.4741	-0.0290	0.0018	0.0002	0.0002
rs72790862	10	44880260	C	T	0.3069	-0.0258	0.0020	-0.0001	0.0002
rs7917772	10	104487443	A	G	0.6308	0.0166	0.0019	-0.0002	0.0002
rs79615245	10	121266907	C	T	0.0249	0.0356	0.0059	0.0003	0.0005
rs79780963	10	104952499	T	C	0.0974	-0.0236	0.0035	0.0004	0.0003
rs9633675	10	96210400	C	G	0.4789	-0.0189	0.0018	0.0000	0.0002
rs10796828	11	69490346	G	T	0.6354	-0.0164	0.0019	0.0002	0.0002
rs10840147	11	8920704	G	A	0.4568	-0.0104	0.0019	0.0003	0.0002
rs10896441	11	68953908	A	G	0.5789	0.0106	0.0019	0.0004	0.0002
rs11022177	11	12133478	G	C	0.3111	-0.0181	0.0020	0.0003	0.0002
rs11025068	11	19261797	T	A	0.2285	-0.0123	0.0022	0.0001	0.0002
rs113519804	11	76020088	A	C	0.0287	-0.0324	0.0057	-0.0003	0.0005
rs11602323	11	122519281	G	T	0.1233	0.0330	0.0028	0.0001	0.0002
rs12226331	11	102070976	T	A	0.3527	-0.0130	0.0019	0.0003	0.0002
rs1228024	11	47951353	A	C	0.6613	0.0252	0.0019	-0.0001	0.0002

rs1232050	11	30798288	C	G	0.4298	-0.0118	0.0020	-0.0002	0.0002
rs12792460	11	18076439	G	C	0.2712	0.0157	0.0021	0.0001	0.0002
rs1468102	11	3004526	G	C	0.3063	0.0149	0.0020	0.0002	0.0002
rs1715429	11	118083664	G	A	0.7526	0.0169	0.0021	-0.0004	0.0002
rs174548	11	61571348	G	C	0.3139	-0.0249	0.0020	0.0001	0.0002
rs1783921	11	128094803	C	T	0.2081	0.0178	0.0023	0.0000	0.0002
rs2870434	11	56071557	A	G	0.5804	-0.0106	0.0019	0.0000	0.0002
rs4909932	11	10475967	G	A	0.5981	0.0138	0.0019	0.0003	0.0002
rs4909945	11	10673739	C	T	0.6889	0.0115	0.0020	-0.0003	0.0002
rs579721	11	59834318	A	G	0.2837	-0.0123	0.0020	0.0000	0.0002
rs58833930	11	2325997	T	C	0.1131	-0.0174	0.0030	-0.0003	0.0003
rs617791	11	65702523	C	G	0.4833	0.0189	0.0019	0.0001	0.0002
rs61904448	11	113958121	C	T	0.2844	-0.0137	0.0021	-0.0004	0.0002
rs6421984	11	305619	C	T	0.5145	0.0387	0.0020	-0.0001	0.0002
rs672058	11	116764021	T	C	0.8805	0.0182	0.0028	0.0006	0.0003
rs7127313	11	100508897	T	C	0.3455	0.0164	0.0019	0.0000	0.0002
rs73000965	11	113982321	A	T	0.3148	0.0288	0.0020	-0.0002	0.0002
rs7934719	11	108341864	T	C	0.4143	0.0179	0.0019	-0.0001	0.0002
rs7940191	11	12874053	A	G	0.6802	-0.0131	0.0020	0.0000	0.0002
rs8705	11	128328913	A	G	0.3166	-0.0264	0.0020	-0.0001	0.0002
rs907612	11	1874221	T	C	0.3802	-0.0110	0.0020	0.0000	0.0002
rs934177	11	44612714	G	C	0.4363	-0.0115	0.0019	0.0000	0.0002
rs1007938	12	26802549	G	A	0.4049	-0.0112	0.0019	-0.0001	0.0002
rs10774624	12	111833788	A	G	0.5139	-0.0645	0.0019	0.0003	0.0002
rs11045171	12	20470199	G	A	0.1983	-0.0142	0.0023	0.0001	0.0002
rs11048425	12	26339986	G	C	0.4912	0.0131	0.0018	0.0001	0.0002
rs11064881	12	120146925	A	G	0.0735	-0.0323	0.0035	-0.0003	0.0003
rs11065394	12	121446922	G	A	0.2108	0.0142	0.0023	0.0001	0.0002
rs11104881	12	88843474	C	T	0.7020	-0.0247	0.0020	-0.0001	0.0002
rs11169302	12	50578705	G	T	0.3924	0.0186	0.0019	0.0003	0.0002
rs12313762	12	57692470	T	C	0.2356	-0.0148	0.0022	0.0001	0.0002
rs1245035	12	64976049	A	C	0.6274	0.0144	0.0019	0.0000	0.0002
rs12580347	12	3388932	C	T	0.5834	-0.0119	0.0019	0.0002	0.0002
rs145781730	12	109842112	C	G	0.0430	0.0320	0.0045	-0.0001	0.0004
rs17041439	12	101873240	C	A	0.0566	0.0379	0.0040	0.0000	0.0004
rs1895994	12	49056663	A	G	0.3245	-0.0130	0.0020	0.0002	0.0002
rs2024385	12	12888438	A	T	0.4271	0.0170	0.0019	-0.0001	0.0002
rs2286599	12	6499533	A	G	0.1415	0.0386	0.0028	-0.0001	0.0002
rs4761234	12	69732105	C	T	0.4824	0.0152	0.0018	-0.0002	0.0002
rs4842266	12	79951566	A	G	0.6876	-0.0142	0.0020	0.0003	0.0002
rs60437214	12	71111551	A	G	0.5285	-0.0113	0.0019	0.0000	0.0002
rs61754230	12	72179446	T	C	0.0191	0.0390	0.0070	-0.0005	0.0006
rs61907807	12	2508296	A	G	0.1008	-0.0175	0.0031	0.0000	0.0003
rs61916677	12	759441	A	G	0.1722	-0.0141	0.0025	-0.0001	0.0002
rs6538677	12	96254528	G	A	0.5991	0.0104	0.0019	0.0000	0.0002
rs706809	12	52294257	C	T	0.7779	0.0223	0.0023	0.0003	0.0002
rs7303131	12	10110724	G	A	0.2672	0.0141	0.0021	-0.0004	0.0002
rs7308123	12	122225225	C	T	0.1812	0.0186	0.0024	0.0001	0.0002
rs73207610	12	112521849	T	G	0.0267	-0.0349	0.0058	-0.0005	0.0005
rs739842	12	48202046	C	T	0.6395	-0.0155	0.0020	-0.0003	0.0002
rs7488780	12	20579392	C	G	0.2050	-0.0163	0.0023	-0.0001	0.0002
rs759488	12	68587155	C	T	0.7049	0.0135	0.0020	0.0000	0.0002
rs78426500	12	123997770	G	T	0.2007	0.0141	0.0023	-0.0004	0.0002
rs78983078	12	112478316	A	G	0.0094	-0.0552	0.0096	0.0006	0.0008
rs7955734	12	4333159	G	C	0.2099	-0.0288	0.0023	-0.0001	0.0002
rs9863	12	124421453	C	T	0.3293	-0.0187	0.0020	0.0000	0.0002
rs12427846	13	37491650	C	T	0.2485	0.0129	0.0022	-0.0001	0.0002
rs12429714	13	99649831	T	C	0.4244	-0.0111	0.0019	0.0001	0.0002

rs138028125	13	28712689	G	C	0.0343	0.0541	0.0056	-0.0001	0.0005
rs150861794	13	109003805	T	C	0.0202	-0.0652	0.0071	-0.0001	0.0007
rs2265146	13	114178222	A	T	0.2806	0.0225	0.0020	-0.0002	0.0002
rs2439963	13	78638416	G	A	0.7366	-0.0124	0.0021	0.0000	0.0002
rs3812849	13	74701736	C	A	0.2665	0.0196	0.0021	0.0001	0.0002
rs55978995	13	73626897	A	C	0.1691	0.0139	0.0025	0.0000	0.0002
rs7326825	13	50113450	A	G	0.7036	0.0207	0.0020	0.0001	0.0002
rs76428106	13	28604007	C	T	0.0135	0.1584	0.0085	-0.0006	0.0007
rs78738581	13	42843630	A	G	0.2313	0.0153	0.0022	0.0004	0.0002
rs806316	13	50838230	G	C	0.5318	0.0160	0.0018	0.0000	0.0002
rs9508005	13	28789794	G	T	0.0942	-0.0225	0.0034	-0.0002	0.0003
rs9526795	13	52341181	C	T	0.2589	-0.0141	0.0021	-0.0002	0.0002
rs9590390	13	114920867	A	G	0.2915	0.0154	0.0021	0.0002	0.0002
rs10138752	14	69179971	T	C	0.0773	-0.0403	0.0035	-0.0003	0.0003
rs10146962	14	101170540	C	T	0.3383	-0.0154	0.0020	-0.0001	0.0002
rs10498635	14	93103309	T	C	0.1832	-0.0312	0.0024	-0.0001	0.0002
rs11620826	14	70120615	C	T	0.0465	-0.0245	0.0044	0.0002	0.0004
rs12898000	14	103835128	C	G	0.7009	0.0187	0.0020	-0.0001	0.0002
rs2038700	14	25461989	C	T	0.3946	0.0344	0.0019	0.0000	0.0002
rs2180369	14	93516465	C	T	0.1108	0.0230	0.0031	0.0003	0.0003
rs2241621	14	81737076	C	A	0.5876	-0.0152	0.0019	-0.0002	0.0002
rs2494747	14	105258437	T	G	0.6149	0.0129	0.0020	0.0000	0.0002
rs4903580	14	77850978	T	C	0.4562	0.0161	0.0018	0.0000	0.0002
rs696	14	35871093	T	C	0.3658	0.0150	0.0019	0.0001	0.0002
rs7159281	14	23756604	G	A	0.6718	-0.0129	0.0020	0.0002	0.0002
rs72664840	14	35596323	T	C	0.1790	0.0206	0.0024	-0.0001	0.0002
rs9323285	14	55863829	A	C	0.3380	0.0121	0.0020	0.0002	0.0002
rs9323612	14	75968608	G	A	0.3295	-0.0114	0.0020	0.0001	0.0002
rs11638256	15	58772807	C	T	0.1205	0.0188	0.0029	-0.0002	0.0003
rs11854390	15	80224817	T	C	0.5622	0.0137	0.0019	-0.0001	0.0002
rs12912487	15	70032794	A	G	0.1006	-0.0180	0.0031	-0.0004	0.0003
rs2062250	15	64672002	A	G	0.9384	0.0506	0.0039	0.0004	0.0003
rs28576226	15	101713668	A	G	0.1221	0.0313	0.0029	-0.0002	0.0002
rs4924450	15	40597229	A	G	0.7065	0.0137	0.0021	0.0002	0.0002
rs60695341	15	51010271	T	C	0.1973	-0.0299	0.0023	0.0001	0.0002
rs62007171	15	77306285	C	T	0.2911	-0.0155	0.0021	-0.0001	0.0002
rs631864	15	70376441	C	T	0.4721	0.0127	0.0019	0.0001	0.0002
rs7183988	15	91428589	G	T	0.5266	-0.0158	0.0019	-0.0002	0.0002
rs72726027	15	42248826	C	T	0.1119	-0.0426	0.0029	-0.0002	0.0003
rs780142	15	62797964	G	T	0.2740	0.0145	0.0021	-0.0001	0.0002
rs8030736	15	90885866	C	T	0.1707	0.0136	0.0025	0.0001	0.0002
rs8034776	15	81895458	A	G	0.7728	-0.0136	0.0022	0.0000	0.0002
rs11574938	16	30485393	C	G	0.5208	0.0377	0.0019	0.0000	0.0002
rs11644125	16	57058974	T	C	0.5974	-0.0178	0.0019	0.0002	0.0002
rs117556162	16	67680806	A	G	0.0569	0.0272	0.0041	-0.0003	0.0003
rs12923943	16	49884569	C	G	0.2089	0.0152	0.0023	0.0001	0.0002
rs12927351	16	74596618	A	C	0.1988	0.0151	0.0023	0.0002	0.0002
rs12929950	16	2144894	A	G	0.0774	-0.0302	0.0036	-0.0001	0.0003
rs12935169	16	67010113	T	C	0.0163	-0.0561	0.0077	-0.0006	0.0007
rs2080506	16	50164672	T	C	0.1560	-0.0165	0.0025	-0.0001	0.0002
rs247826	16	84582965	T	C	0.2206	0.0338	0.0022	-0.0002	0.0002
rs2667648	16	78581138	C	T	0.7676	0.0134	0.0022	0.0000	0.0002
rs28853644	16	30801027	T	C	0.2720	-0.0177	0.0021	0.0002	0.0002
rs4984768	16	1253907	T	G	0.3634	0.0120	0.0020	0.0002	0.0002
rs55857430	16	89379071	A	G	0.0403	0.0278	0.0047	-0.0001	0.0004
rs56308051	16	53141008	T	A	0.3926	-0.0106	0.0019	0.0000	0.0002
rs58124292	16	81890435	A	G	0.1914	-0.0143	0.0024	-0.0003	0.0002
rs59404182	16	88852866	G	A	0.2000	-0.0185	0.0025	0.0000	0.0002

rs61739285	16	27480797	T	C	0.0333	-0.0337	0.0052	-0.0002	0.0005
rs6500550	16	3746241	T	C	0.3045	-0.0207	0.0020	-0.0003	0.0002
rs7198940	16	81605559	T	C	0.5224	0.0156	0.0019	0.0000	0.0002
rs78784853	16	11817466	C	A	0.0326	0.0327	0.0054	0.0003	0.0005
rs9925985	16	31305593	C	A	0.2695	-0.0141	0.0021	0.0000	0.0002
rs9926183	16	1363878	T	C	0.2627	0.0186	0.0022	-0.0004	0.0002
rs9933582	16	86016026	G	T	0.2296	0.0215	0.0022	0.0000	0.0002
rs1024091	17	40771994	T	C	0.5577	0.0138	0.0018	0.0001	0.0002
rs1034686	17	35899141	A	T	0.5838	-0.0135	0.0019	-0.0001	0.0002
rs1109278	17	76248229	G	A	0.4838	-0.0144	0.0019	-0.0001	0.0002
rs11871779	17	37269770	T	C	0.0315	-0.0357	0.0055	0.0001	0.0005
rs12935890	17	46090769	T	G	0.2738	0.0148	0.0021	0.0000	0.0002
rs12936529	17	16168784	T	C	0.4788	-0.0252	0.0018	0.0003	0.0002
rs12941069	17	74460585	C	T	0.6497	-0.0116	0.0020	0.0001	0.0002
rs12946510	17	37912377	T	C	0.4720	-0.0590	0.0018	-0.0001	0.0002
rs12950923	17	2868935	C	T	0.8317	0.0149	0.0026	-0.0002	0.0002
rs147180766	17	37835355	A	C	0.0182	-0.0541	0.0071	-0.0005	0.0006
rs16964983	17	27610862	C	T	0.1729	0.0219	0.0024	-0.0001	0.0002
rs2084312	17	72695211	T	C	0.8004	0.0289	0.0023	0.0001	0.0002
rs2259855	17	28097860	C	T	0.5253	-0.0236	0.0018	-0.0003	0.0002
rs2376263	17	33763678	G	A	0.2799	0.0114	0.0020	-0.0001	0.0002
rs2665405	17	57875292	A	G	0.5477	0.0354	0.0018	0.0000	0.0002
rs28461839	17	5180538	T	G	0.2091	0.0196	0.0023	-0.0002	0.0002
rs35111447	17	62372190	T	C	0.0983	-0.0179	0.0031	-0.0002	0.0003
rs4792687	17	8160602	C	T	0.4997	0.0106	0.0019	-0.0003	0.0002
rs4985828	17	20994235	G	T	0.5130	0.0108	0.0019	0.0002	0.0002
rs55767800	17	17674967	C	T	0.6323	-0.0184	0.0020	0.0002	0.0002
rs56378716	17	56356502	G	A	0.0128	0.1076	0.0083	-0.0009	0.0007
rs61759532	17	7240391	T	C	0.2401	-0.0257	0.0023	-0.0003	0.0002
rs6504179	17	61812995	G	T	0.6403	-0.0125	0.0019	0.0003	0.0002
rs7225843	17	2001825	C	T	0.2031	-0.0315	0.0023	0.0003	0.0002
rs74480102	17	7742601	A	G	0.0411	-0.0489	0.0047	0.0004	0.0004
rs74725931	17	38196327	C	T	0.0373	0.0614	0.0051	-0.0003	0.0004
rs78357146	17	64305051	G	A	0.0302	0.0298	0.0054	-0.0003	0.0005
rs8067958	17	1390029	C	G	0.8562	0.0165	0.0028	-0.0004	0.0002
rs820384	17	73755392	C	G	0.6634	0.0134	0.0021	0.0002	0.0002
rs9912009	17	74694320	T	C	0.4897	-0.0124	0.0018	0.0000	0.0002
rs9972936	17	43353677	A	G	0.5491	0.0154	0.0019	0.0001	0.0002
rs11664534	18	43724520	T	C	0.5246	-0.0159	0.0019	0.0002	0.0002
rs11874453	18	20750400	A	G	0.5326	-0.0116	0.0019	0.0003	0.0002
rs12956324	18	67537270	A	C	0.4030	0.0138	0.0019	0.0004	0.0002
rs17758695	18	60920854	T	C	0.0286	-0.0559	0.0057	0.0000	0.0005
rs303753	18	21074922	A	G	0.3447	-0.0196	0.0020	0.0000	0.0002
rs4468717	18	3457606	T	C	0.0778	-0.0200	0.0035	0.0006	0.0003
rs4940320	18	51776316	A	T	0.3011	0.0169	0.0020	0.0001	0.0002
rs67491878	18	78003207	C	A	0.6044	0.0111	0.0019	-0.0002	0.0002
rs7231404	18	60123141	G	C	0.3011	-0.0128	0.0020	0.0000	0.0002
rs7235882	18	19684119	T	C	0.0938	0.0178	0.0032	0.0000	0.0003
rs72973711	18	74072245	T	A	0.0671	-0.0318	0.0037	-0.0007	0.0003
rs78285907	18	21622003	T	A	0.0890	0.0198	0.0033	-0.0001	0.0003
rs784257	18	53397199	C	T	0.8142	0.0137	0.0024	0.0000	0.0002
rs8084255	18	48144754	T	C	0.3756	0.0199	0.0019	0.0000	0.0002
rs9965539	18	41973779	A	G	0.1338	0.0251	0.0027	0.0000	0.0002
rs1010047	19	7793502	G	A	0.5507	-0.0104	0.0019	-0.0002	0.0002
rs12459419	19	51728477	T	C	0.3216	-0.0271	0.0020	0.0003	0.0002
rs2290669	19	16495774	C	A	0.7864	-0.0476	0.0023	0.0000	0.0002
rs2304130	19	19789528	G	A	0.0836	0.0216	0.0033	0.0000	0.0003
rs2358581	19	10391611	G	T	0.7329	-0.0282	0.0022	-0.0001	0.0002

rs237698	19	13933384	C	T	0.3365	0.0176	0.0020	0.0000	0.0002
rs2617802	19	49114479	G	T	0.2069	-0.0150	0.0023	0.0004	0.0002
rs309190	19	47629264	C	T	0.8939	-0.0305	0.0032	0.0001	0.0003
rs31725	19	39914308	T	C	0.4388	-0.0130	0.0019	0.0000	0.0002
rs35665378	19	1092837	A	G	0.1323	-0.0270	0.0030	0.0001	0.0002
rs4760	19	44153100	G	A	0.1527	-0.0702	0.0026	-0.0002	0.0002
rs4805881	19	33896432	C	A	0.6653	-0.0164	0.0020	0.0000	0.0002
rs4807440	19	1026477	T	G	0.6367	0.0253	0.0020	0.0003	0.0002
rs4808683	19	17862925	G	C	0.4583	-0.0128	0.0019	0.0000	0.0002
rs56121005	19	11414706	T	C	0.0226	0.0402	0.0068	-0.0006	0.0006
rs571497	19	7827830	A	G	0.1548	-0.0364	0.0026	-0.0004	0.0002
rs61377791	19	35525322	C	A	0.3146	0.0137	0.0020	-0.0001	0.0002
rs73036517	19	45744842	G	A	0.2588	-0.0527	0.0021	0.0000	0.0002
rs851612	19	42769693	G	A	0.0548	0.0296	0.0042	0.0001	0.0004
rs1800961	20	43042364	T	C	0.0311	-0.0378	0.0053	-0.0001	0.0005
rs2327028	20	8140925	T	C	0.4757	-0.0135	0.0019	-0.0002	0.0002
rs2427599	20	62687921	T	C	0.0107	0.0557	0.0095	0.0012	0.0008
rs2904270	20	49185272	A	G	0.5171	-0.0153	0.0018	0.0000	0.0002
rs439749	20	55968113	A	G	0.6497	-0.0149	0.0020	0.0001	0.0002
rs4411786	20	1930897	C	T	0.2661	-0.0206	0.0021	-0.0002	0.0002
rs513583	20	2904768	T	C	0.1738	0.0151	0.0025	0.0002	0.0002
rs6029234	20	39259278	C	G	0.6242	0.0242	0.0019	0.0001	0.0002
rs6055955	20	8604181	T	C	0.5086	-0.0222	0.0018	0.0000	0.0002
rs6059938	20	33187130	A	G	0.4864	0.0108	0.0018	0.0000	0.0002
rs6062468	20	62268955	T	C	0.5081	0.0108	0.0019	0.0001	0.0002
rs611847	20	3684022	G	A	0.6360	0.0118	0.0020	0.0005	0.0002
rs62210106	20	47339149	G	A	0.0320	-0.0301	0.0053	0.0005	0.0005
rs67139609	20	36888321	G	C	0.1446	0.0169	0.0027	-0.0001	0.0002
rs916410	20	42203237	T	C	0.7608	0.0123	0.0022	-0.0004	0.0002
rs1803439	21	38885442	G	A	0.3457	-0.0113	0.0019	0.0000	0.0002
rs2096507	21	47941916	A	G	0.4695	-0.0172	0.0018	0.0000	0.0002
rs2832258	21	30582732	G	A	0.7862	-0.0139	0.0023	0.0000	0.0002
rs28574812	21	16384940	G	A	0.1579	-0.0160	0.0025	0.0000	0.0002
rs35990176	21	44472118	C	A	0.4511	0.0116	0.0019	-0.0002	0.0002
rs990558	21	36914669	T	C	0.3614	-0.0111	0.0019	-0.0001	0.0002
rs9977672	21	40463283	A	G	0.2591	-0.0219	0.0021	0.0001	0.0002
rs1033415	22	39888774	G	A	0.6504	0.0127	0.0020	0.0001	0.0002
rs140489	22	21921294	A	G	0.2033	-0.0143	0.0024	-0.0001	0.0002
rs2076211	22	44329078	T	C	0.1585	-0.0180	0.0025	0.0001	0.0002
rs2180142	22	31855248	A	T	0.0506	-0.0250	0.0042	-0.0004	0.0004
rs34505104	22	24624609	G	A	0.3049	-0.0204	0.0021	0.0000	0.0002
rs389347	22	18294697	T	C	0.3646	0.0143	0.0020	0.0000	0.0002
rs47341	22	43560763	T	C	0.3964	0.0159	0.0020	0.0000	0.0002
rs5746451	22	18126020	C	T	0.5042	0.0195	0.0018	0.0001	0.0002
rs5760175	22	24399655	G	T	0.5272	0.0106	0.0019	-0.0001	0.0002
rs5994158	22	17567898	G	A	0.8093	-0.0206	0.0024	-0.0002	0.0002
rs713875	22	30592487	G	C	0.5515	-0.0151	0.0019	-0.0004	0.0002
rs713909	22	39532420	C	G	0.4320	-0.0202	0.0019	0.0000	0.0002
rs9625746	22	29637658	C	G	0.4119	-0.0184	0.0019	0.0001	0.0002
