# Supplementary material for: Blood cell traits and risk of glaucoma: A two-sample mendelian randomization study
Source: Front Genet. 2023 Apr 12;14:1142773. doi: 10.3389/fgene.2023.1142773 (PMC10130872; doi:10.3389/fgene.2023.1142773)
Supplement: Supplementary file 1 [file DataSheet1.ZIP › eTable 5. Neutrophil cell count exposure SNPs and their association with glaucoma.pdf]

eTable 5. Neutrophil cell count exposure SNPs and their association with glaucoma.
Chr = chromosome; POS = position ; EA = effect allele; NEA = non-effect allele; EAF = effect allele frequency; SE = standard error.

SNP	Chr	POS	EA	NEA	EAF	Neutrophil Cell Count		Glaucoma	
						Beta	SE	Beta	SE
rs10489182	1	169710669	G	A	0.1919	0.0141	0.0024	-0.0004	0.0002
rs10864368	1	8918313	C	T	0.5027	0.0237	0.0019	0.0001	0.0002
rs11205310	1	149965170	T	G	0.1444	-0.0180	0.0027	0.0000	0.0002
rs1135172	1	11301714	G	A	0.7300	0.0121	0.0022	-0.0001	0.0002
rs114427170	1	159240076	A	G	0.0366	0.0319	0.0054	0.0004	0.0005
rs11587827	1	17671382	G	C	0.5829	-0.0114	0.0020	-0.0002	0.0002
rs116631966	1	93229909	A	G	0.0248	0.0403	0.0062	-0.0002	0.0005
rs12118277	1	56609296	T	C	0.3519	0.0149	0.0020	0.0000	0.0002
rs12730312	1	27378338	A	G	0.0729	-0.0154	0.0027	-0.0003	0.0003
rs140391508	1	160913404	G	C	0.0183	-0.0443	0.0076	-0.0001	0.0006
rs150649461	1	92925654	C	G	0.0150	0.0528	0.0083	0.0005	0.0007
rs16843350	1	198543095	T	A	0.0302	-0.0338	0.0057	0.0002	0.0005
rs1707304	1	46598269	A	C	0.4368	-0.0193	0.0019	0.0002	0.0002
rs17440390	1	67190563	T	C	0.1262	0.0165	0.0029	-0.0004	0.0002
rs17537224	1	167587813	G	T	0.1727	0.0155	0.0025	0.0000	0.0002
rs17728	1	205241659	C	T	0.9090	0.0238	0.0033	-0.0004	0.0003
rs1776270	1	150369337	A	G	0.6314	0.0160	0.0020	0.0001	0.0002
rs1886654	1	236105910	C	T	0.8920	-0.0585	0.0031	0.0003	0.0003
rs2476601	1	114377568	G	A	0.8997	0.0380	0.0032	0.0001	0.0003
rs2501309	1	22341714	C	T	0.6547	-0.0068	0.0012	0.0000	0.0002
rs2808519	1	200361914	T	G	0.7376	0.0161	0.0022	-0.0002	0.0002
rs284317	1	10731625	G	A	0.4960	0.0136	0.0020	0.0001	0.0002
rs301819	1	8501786	G	A	0.5838	-0.0140	0.0020	0.0001	0.0002
rs34599082	1	159175494	T	C	0.0134	-0.1730	0.0084	0.0005	0.0007
rs35020138	1	247570221	C	T	0.6753	-0.0176	0.0021	0.0003	0.0002
rs35571080	1	224637790	C	T	0.2122	0.0245	0.0023	-0.0002	0.0002
rs3738182	1	221057662	A	G	0.1936	0.0219	0.0024	0.0001	0.0002
rs3754224	1	43423622	C	T	0.2700	-0.0167	0.0022	-0.0003	0.0002
rs3762297	1	31231680	T	C	0.1833	0.0239	0.0025	0.0000	0.0002
rs3795445	1	227180947	C	A	0.5098	-0.0236	0.0020	0.0003	0.0002
rs3843301	1	161614401	G	T	0.3648	-0.0189	0.0021	0.0000	0.0002
rs3917932	1	36943916	G	C	0.5771	-0.0515	0.0019	0.0000	0.0002
rs41272536	1	183440531	G	A	0.0460	-0.0359	0.0049	-0.0001	0.0004
rs41313381	1	79411968	A	C	0.0305	0.0587	0.0055	-0.0001	0.0005
rs4626924	1	234909298	T	C	0.5513	0.0194	0.0020	0.0000	0.0002
rs4655802	1	65888231	A	G	0.5898	-0.0131	0.0020	0.0001	0.0002
rs4657609	1	166839174	A	G	0.2001	-0.0219	0.0024	0.0002	0.0002
rs4844622	1	208034329	T	C	0.2403	-0.0234	0.0022	0.0001	0.0002
rs4925756	1	248048016	T	C	0.6919	-0.0130	0.0021	0.0002	0.0002
rs501791	1	156089873	T	C	0.0450	-0.0313	0.0046	0.0000	0.0004
rs533483	1	234765256	A	G	0.2440	-0.0171	0.0023	0.0003	0.0002
rs547364	1	108742123	G	C	0.4633	0.0196	0.0019	-0.0001	0.0002
rs55873273	1	36972428	C	T	0.0202	0.0590	0.0068	-0.0003	0.0006
rs56188865	1	247606276	C	T	0.3737	-0.0274	0.0020	0.0003	0.0002
rs57257429	1	42202144	A	G	0.3531	-0.0170	0.0020	0.0001	0.0002
rs6678033	1	66077624	A	G	0.3679	-0.0427	0.0020	0.0000	0.0002
rs6696163	1	174015631	C	T	0.1797	-0.0177	0.0025	0.0003	0.0002
rs694180	1	111726213	G	A	0.6563	0.0188	0.0020	-0.0003	0.0002
rs7537229	1	56906274	A	G	0.8961	0.0423	0.0032	0.0001	0.0003
rs76469486	1	27015338	T	A	0.0803	-0.0124	0.0021	0.0006	0.0003
rs10048745	2	68962137	A	G	0.2569	0.0150	0.0022	0.0000	0.0002
rs10181102	2	111835489	C	T	0.2724	0.0157	0.0022	0.0000	0.0002

rs1047891	2	211540507	A	C	0.3151	-0.0193	0.0021	0.0002	0.0002
rs1057258	2	234115629	T	C	0.1781	0.0165	0.0026	0.0000	0.0002
rs114050631	2	219020958	T	C	0.0111	-0.1626	0.0101	0.0002	0.0008
rs11678685	2	169710607	A	G	0.3177	0.0229	0.0021	-0.0003	0.0002
rs11683933	2	127886955	A	C	0.0551	0.0306	0.0042	0.0010	0.0004
rs11894199	2	128543369	A	C	0.3876	0.0110	0.0020	0.0000	0.0002
rs12328347	2	43351269	G	T	0.3727	-0.0161	0.0020	0.0000	0.0002
rs1260326	2	27730940	C	T	0.6020	-0.0325	0.0020	0.0000	0.0002
rs13392977	2	192514856	A	G	0.0540	0.0282	0.0043	0.0002	0.0004
rs13430448	2	153276084	A	G	0.0924	-0.0206	0.0033	-0.0001	0.0003
rs1521134	2	219995186	T	G	0.6421	0.0123	0.0020	-0.0001	0.0002
rs17026212	2	85551696	G	T	0.5129	-0.0168	0.0019	-0.0007	0.0002
rs188653407	2	160671563	G	C	0.0229	-0.0512	0.0065	-0.0005	0.0005
rs190940054	2	37399898	A	G	0.0336	-0.0391	0.0053	0.0001	0.0005
rs2052249	2	202665227	A	C	0.2509	-0.0127	0.0022	0.0003	0.0002
rs2421200	2	61711815	T	G	0.4866	-0.0205	0.0019	0.0002	0.0002
rs2729707	2	160687231	G	A	0.8308	-0.0357	0.0026	-0.0002	0.0002
rs309180	2	136614255	A	G	0.7681	-0.0142	0.0023	0.0002	0.0002
rs354703	2	143883952	C	T	0.5935	0.0148	0.0020	0.0000	0.0002
rs35789178	2	102603925	G	T	0.1862	0.0180	0.0025	0.0001	0.0002
rs3856364	2	145477217	G	C	0.6797	0.0143	0.0021	-0.0002	0.0002
rs4074882	2	237780727	C	T	0.7418	0.0279	0.0022	0.0002	0.0002
rs4632345	2	16702654	A	G	0.6780	-0.0122	0.0021	-0.0004	0.0002
rs55729107	2	232582085	G	A	0.2256	-0.0175	0.0023	0.0002	0.0002
rs58926146	2	60640503	T	C	0.2900	0.0116	0.0021	0.0002	0.0002
rs62189859	2	182254087	C	T	0.2444	0.0141	0.0022	0.0004	0.0002
rs633323	2	31463044	T	A	0.6973	0.0162	0.0021	0.0000	0.0002
rs6432335	2	12928971	G	T	0.4516	0.0122	0.0019	-0.0001	0.0002
rs6723009	2	42474615	C	T	0.7174	0.0121	0.0022	-0.0001	0.0002
rs6731993	2	65642097	T	A	0.4083	0.0228	0.0020	0.0001	0.0002
rs6734238	2	113841030	G	A	0.4030	0.0394	0.0020	-0.0001	0.0002
rs6740847	2	182308352	G	A	0.5621	0.0217	0.0019	0.0003	0.0002
rs72789923	2	24394504	C	A	0.3074	0.0205	0.0021	-0.0002	0.0002
rs75166367	2	162964301	A	G	0.0598	-0.0306	0.0041	-0.0001	0.0003
rs75475627	2	54787592	G	C	0.0768	0.0330	0.0037	0.0000	0.0003
rs7569453	2	70402180	A	T	0.0578	-0.0303	0.0041	0.0008	0.0004
rs7575465	2	27291661	A	G	0.5534	-0.0127	0.0019	0.0001	0.0002
rs77552263	2	43786818	A	G	0.0780	0.0486	0.0036	0.0002	0.0003
rs79047930	2	220165527	T	C	0.0147	0.0534	0.0084	-0.0002	0.0007
rs796056	2	101735276	C	A	0.6243	0.0223	0.0020	0.0002	0.0002
rs79710932	2	218947121	T	G	0.0778	-0.0304	0.0036	-0.0004	0.0003
rs10049210	3	156797373	C	T	0.4034	0.0153	0.0020	-0.0002	0.0002
rs10935473	3	98416900	T	G	0.4415	0.0160	0.0019	-0.0001	0.0002
rs11710513	3	150707804	G	A	0.0911	0.0206	0.0036	0.0000	0.0003
rs11712552	3	140957762	G	A	0.1262	0.0305	0.0029	-0.0003	0.0002
rs11716015	3	169320058	T	C	0.6357	-0.0129	0.0020	-0.0001	0.0002
rs12487658	3	71528943	T	C	0.8016	0.0170	0.0024	-0.0001	0.0002
rs1366045	3	42909050	C	T	0.3850	-0.0219	0.0020	0.0001	0.0002
rs2046934	3	151058247	A	G	0.8232	0.0150	0.0025	-0.0003	0.0002
rs2284837	3	185999077	G	A	0.2203	0.0149	0.0023	-0.0003	0.0002
rs2734031	3	128301390	C	T	0.9080	-0.0675	0.0033	-0.0005	0.0003
rs3749440	3	183702089	G	A	0.3724	0.0174	0.0020	-0.0001	0.0002
rs56217149	3	12962914	A	G	0.1507	0.0174	0.0028	0.0004	0.0002
rs58605236	3	119697032	A	T	0.3731	-0.0139	0.0020	0.0000	0.0002
rs6414435	3	47121140	G	A	0.5918	0.0171	0.0020	0.0002	0.0002
rs6764912	3	128372487	A	G	0.0653	-0.0372	0.0040	-0.0004	0.0003
rs6779340	3	58033701	G	C	0.3369	-0.0152	0.0020	0.0003	0.0002
rs73222236	3	135888642	G	A	0.3356	-0.0111	0.0020	-0.0004	0.0002

rs7626444	3	196504902	C	G	0.4215	-0.0208	0.0019	0.0000	0.0002
rs7639292	3	107295665	T	C	0.1691	-0.0223	0.0026	0.0005	0.0002
rs7652649	3	150707915	T	G	0.5845	0.0139	0.0021	-0.0003	0.0002
rs789858	3	194405966	T	C	0.4037	0.0140	0.0020	0.0002	0.0002
rs79953286	3	132226100	G	A	0.0587	0.0285	0.0041	0.0000	0.0004
rs832187	3	63833050	T	C	0.6311	-0.0131	0.0020	0.0004	0.0002
rs9819371	3	141206800	T	C	0.0649	-0.0431	0.0039	0.0000	0.0003
rs9867398	3	185912816	T	C	0.0943	0.0206	0.0033	0.0000	0.0003
rs10012827	4	41543318	T	C	0.1243	0.0163	0.0030	0.0007	0.0003
rs10804990	4	6919661	A	G	0.6175	0.0207	0.0020	-0.0003	0.0002
rs10939663	4	10032516	G	T	0.2794	0.0118	0.0021	0.0000	0.0002
rs114628112	4	7054867	A	C	0.0159	-0.0476	0.0078	-0.0002	0.0007
rs11723621	4	72615362	G	A	0.2908	-0.0269	0.0021	0.0000	0.0002
rs11735662	4	145026126	T	C	0.0336	0.0476	0.0053	0.0000	0.0004
rs140311179	4	26295540	G	C	0.0512	-0.0249	0.0044	0.0008	0.0004
rs1563645	4	83941387	G	A	0.8139	0.0149	0.0025	0.0001	0.0002
rs16850073	4	74703999	T	C	0.3745	0.0521	0.0020	-0.0001	0.0002
rs17005891	4	83547862	A	G	0.1842	-0.0286	0.0025	0.0000	0.0002
rs17213043	4	105546979	G	C	0.0283	0.0460	0.0058	0.0003	0.0005
rs218264	4	55408875	T	A	0.2508	0.0318	0.0022	0.0001	0.0002
rs2290846	4	151199080	A	G	0.2843	0.0188	0.0021	0.0004	0.0002
rs2522440	4	108644404	C	T	0.9119	0.0189	0.0034	0.0003	0.0003
rs28530750	4	36312542	A	G	0.0431	0.0561	0.0048	-0.0006	0.0004
rs35734242	4	706700	C	T	0.4278	0.0234	0.0020	-0.0001	0.0002
rs370655	4	74903134	C	T	0.3945	0.0619	0.0020	0.0003	0.0002
rs4145952	4	120155806	A	C	0.3993	-0.0133	0.0020	0.0001	0.0002
rs4696256	4	152291432	A	G	0.5258	0.0151	0.0019	-0.0001	0.0002
rs723585	4	55503194	G	A	0.4835	-0.0223	0.0019	0.0002	0.0002
rs762855	4	3074795	G	A	0.4569	-0.0138	0.0019	-0.0001	0.0002
rs7658676	4	39603132	T	C	0.3530	0.0111	0.0020	0.0000	0.0002
rs7679673	4	106061534	A	C	0.3798	-0.0238	0.0020	0.0001	0.0002
rs7684253	4	57727311	T	C	0.5504	0.0142	0.0019	0.0002	0.0002
rs114792	5	149460953	T	C	0.5404	-0.0112	0.0019	0.0003	0.0002
rs11738307	5	173108632	T	G	0.3529	0.0143	0.0020	0.0000	0.0002
rs12658947	5	57552464	G	A	0.2568	0.0130	0.0022	0.0001	0.0002
rs13171895	5	131841136	A	G	0.5117	0.0139	0.0019	-0.0002	0.0002
rs1445171	5	100046075	C	T	0.5169	0.0143	0.0019	0.0000	0.0002
rs1948760	5	156442784	A	T	0.8288	-0.0218	0.0025	0.0003	0.0002
rs1966479	5	118627271	G	A	0.6602	0.0144	0.0020	0.0003	0.0002
rs2082382	5	148200553	A	G	0.5524	-0.0295	0.0019	-0.0003	0.0002
rs2432142	5	96275201	A	G	0.4350	0.0149	0.0019	-0.0001	0.0002
rs2522051	5	131797578	C	T	0.4544	0.0240	0.0019	0.0002	0.0002
rs2561758	5	173205282	G	A	0.7230	-0.0340	0.0022	-0.0001	0.0002
rs257063	5	114806819	T	C	0.7412	-0.0148	0.0022	0.0001	0.0002
rs407883	5	176529705	C	G	0.6924	0.0123	0.0021	0.0003	0.0002
rs4391200	5	141509537	G	A	0.6184	0.0238	0.0020	-0.0005	0.0002
rs4535497	5	1107428	A	C	0.5699	-0.0144	0.0020	-0.0001	0.0002
rs464609	5	34654477	A	G	0.5444	0.0107	0.0019	-0.0003	0.0002
rs5745297	5	10680997	A	G	0.0881	-0.0197	0.0035	-0.0008	0.0003
rs59327154	5	156944895	G	A	0.1780	-0.0157	0.0025	0.0000	0.0002
rs62360185	5	57274612	G	A	0.1907	-0.0159	0.0025	-0.0001	0.0002
rs62406226	5	179237968	T	G	0.3418	-0.0179	0.0021	-0.0002	0.0002
rs6859727	5	71742622	C	T	0.8780	-0.0404	0.0029	-0.0003	0.0003
rs6877725	5	68610623	C	T	0.4448	-0.0251	0.0019	-0.0002	0.0002
rs6878780	5	122093740	C	T	0.4265	0.0133	0.0019	0.0002	0.0002
rs6891328	5	133851526	C	G	0.5831	0.0123	0.0019	0.0002	0.0002
rs7705526	5	1285974	A	C	0.3270	0.0336	0.0022	0.0000	0.0002
rs78404578	5	86398011	A	G	0.1765	0.0166	0.0026	0.0000	0.0002

rs986564	5	142547161	T	A	0.0734	0.0237	0.0037	0.0005	0.0003
rs10807272	6	41983835	G	A	0.2294	0.0150	0.0023	0.0000	0.0002
rs10945542	6	158752931	T	C	0.5212	0.0120	0.0019	0.0000	0.0002
rs10948036	6	42510305	A	C	0.2102	0.0297	0.0024	-0.0003	0.0002
rs12110437	6	29964829	T	A	0.0676	0.0257	0.0039	0.0000	0.0003
rs12206743	6	52285394	C	G	0.2487	-0.0158	0.0022	0.0000	0.0002
rs12214269	6	135846518	A	G	0.5455	0.0194	0.0019	0.0003	0.0002
rs12215332	6	121788972	A	G	0.1998	0.0174	0.0024	0.0000	0.0002
rs12527929	6	2768816	T	G	0.1578	-0.0153	0.0026	-0.0002	0.0002
rs13207689	6	27369704	G	C	0.1067	-0.0450	0.0031	-0.0004	0.0003
rs144721194	6	7223858	G	T	0.0229	-0.0365	0.0066	0.0003	0.0006
rs1490384	6	126851160	T	C	0.5014	-0.0147	0.0019	0.0003	0.0002
rs17710008	6	153043035	A	G	0.1816	0.0148	0.0025	-0.0004	0.0002
rs212409	6	159470058	A	G	0.5542	-0.0212	0.0019	-0.0003	0.0002
rs2230365	6	31525448	T	C	0.1432	0.0246	0.0027	0.0001	0.0002
rs2844594	6	31258393	A	G	0.1986	0.0559	0.0024	0.0004	0.0002
rs3128932	6	33078082	T	C	0.1948	0.0176	0.0024	0.0000	0.0002
rs3777755	6	12159699	T	C	0.3114	0.0137	0.0021	0.0000	0.0002
rs4142967	6	90996349	T	C	0.4627	-0.0137	0.0019	0.0002	0.0002
rs4712614	6	21382765	G	T	0.6203	-0.0199	0.0020	-0.0002	0.0002
rs4713999	6	36633069	G	A	0.6615	0.0151	0.0020	0.0000	0.0002
rs610604	6	138199417	T	G	0.6745	-0.0161	0.0020	-0.0001	0.0002
rs6454596	6	87980735	C	G	0.4897	-0.0253	0.0019	0.0002	0.0002
rs68016381	6	43761645	T	C	0.0513	-0.0338	0.0044	0.0003	0.0004
rs6901423	6	22086292	G	A	0.5674	0.0123	0.0019	0.0000	0.0002
rs6907749	6	138129456	T	G	0.0860	-0.0207	0.0034	-0.0004	0.0003
rs6915310	6	16758002	T	C	0.1740	-0.0323	0.0026	0.0001	0.0002
rs6924387	6	137082948	G	A	0.4122	0.0173	0.0020	0.0000	0.0002
rs6927569	6	109621494	C	T	0.5232	0.0259	0.0019	0.0002	0.0002
rs6936191	6	7201660	C	T	0.6080	0.0181	0.0020	-0.0002	0.0002
rs72992130	6	144441671	T	C	0.0454	-0.0469	0.0047	-0.0001	0.0004
rs7746069	6	35236306	C	T	0.9651	0.0300	0.0053	0.0000	0.0004
rs7750910	6	14723543	C	G	0.1877	-0.0136	0.0025	-0.0003	0.0002
rs915125	6	82463376	T	C	0.2820	-0.0176	0.0021	0.0001	0.0002
rs9268839	6	32428772	G	A	0.4690	0.0591	0.0019	0.0002	0.0002
rs9296383	6	42283391	C	T	0.2709	-0.0131	0.0022	-0.0001	0.0002
rs9350276	6	20740296	T	C	0.3949	0.0113	0.0020	-0.0001	0.0002
rs9390461	6	147701217	G	A	0.5389	0.0177	0.0019	0.0001	0.0002
rs9402685	6	135419688	C	T	0.2560	-0.0338	0.0022	0.0001	0.0002
rs10252457	7	47337530	G	A	0.4256	-0.0138	0.0020	-0.0001	0.0002
rs10260281	7	38270550	C	G	0.2371	0.0141	0.0023	0.0003	0.0002
rs1045916	7	97933601	T	C	0.7479	-0.0190	0.0023	0.0003	0.0002
rs11983987	7	75657850	G	A	0.1668	-0.0172	0.0026	0.0001	0.0002
rs13235246	7	65161580	A	G	0.1015	0.0278	0.0032	0.0003	0.0003
rs1474419	7	6692605	C	T	0.5788	0.0177	0.0020	-0.0003	0.0002
rs149007767	7	50370254	T	C	0.1621	0.0298	0.0027	-0.0001	0.0002
rs17139597	7	64731746	G	T	0.0399	-0.0341	0.0049	-0.0004	0.0004
rs182090955	7	92239892	A	G	0.0099	-0.0689	0.0100	-0.0008	0.0009
rs2158799	7	28277107	G	C	0.6114	0.0520	0.0020	0.0001	0.0002
rs2301557	7	92300863	T	C	0.0182	-0.1258	0.0072	-0.0006	0.0006
rs2644312	7	2841164	A	G	0.2987	0.0141	0.0021	0.0001	0.0002
rs2710804	7	36084529	C	T	0.3764	0.0197	0.0020	0.0004	0.0002
rs33951980	7	73029437	T	C	0.1294	-0.0276	0.0029	0.0002	0.0002
rs342242	7	106338989	C	T	0.4531	-0.0158	0.0019	0.0001	0.0002
rs35759345	7	116388021	T	C	0.4499	0.0126	0.0019	-0.0001	0.0002
rs3731332	7	92300568	T	C	0.0229	-0.1196	0.0065	0.0005	0.0006
rs3735311	7	148877737	C	T	0.6487	-0.0195	0.0020	0.0001	0.0002
rs3735485	7	45009341	G	A	0.8449	0.0295	0.0027	0.0001	0.0002

rs4721668	7	17942406	T	C	0.1215	-0.0215	0.0029	0.0001	0.0003
rs4722166	7	22738762	A	C	0.6380	-0.0136	0.0020	0.0001	0.0002
rs4729046	7	92223957	C	T	0.9242	-0.0428	0.0036	0.0000	0.0003
rs4948097	7	56056571	G	A	0.7585	-0.0149	0.0022	0.0001	0.0002
rs56388170	7	28724374	T	G	0.2940	0.0700	0.0021	0.0000	0.0002
rs6463566	7	6528174	G	C	0.2297	-0.0170	0.0023	0.0000	0.0002
rs73049276	7	8022016	A	G	0.0585	-0.0395	0.0041	0.0001	0.0003
rs74621719	7	137942779	A	G	0.1750	0.0171	0.0025	0.0001	0.0002
rs77487661	7	80102233	T	C	0.0105	0.0585	0.0096	0.0012	0.0009
rs7803075	7	130742066	G	A	0.7352	-0.0193	0.0022	0.0000	0.0002
rs9656395	7	130575884	G	A	0.0953	-0.0228	0.0033	-0.0002	0.0003
rs9656588	7	50306780	C	T	0.6700	0.0221	0.0020	0.0002	0.0002
rs11774578	8	5543809	C	T	0.1913	0.0170	0.0025	-0.0002	0.0002
rs11993347	8	103919090	C	T	0.2301	-0.0216	0.0023	-0.0002	0.0002
rs11997631	8	129000499	G	C	0.2736	0.0166	0.0022	0.0000	0.0002
rs12216862	8	41828063	T	C	0.2887	0.0137	0.0022	-0.0001	0.0002
rs12541521	8	129986235	G	A	0.4502	0.0127	0.0019	-0.0003	0.0002
rs12550612	8	22966769	A	G	0.8220	-0.0308	0.0025	-0.0001	0.0002
rs12716647	8	6901304	C	G	0.6374	0.0143	0.0020	0.0000	0.0002
rs16939607	8	79013333	A	G	0.1460	-0.0284	0.0027	0.0001	0.0002
rs1991651	8	10706411	G	C	0.6180	0.0229	0.0020	-0.0001	0.0002
rs28615248	8	55451193	C	T	0.1940	-0.0234	0.0024	-0.0002	0.0002
rs34215892	8	21767240	A	G	0.0285	0.0381	0.0060	-0.0005	0.0005
rs3802225	8	87060672	G	A	0.3686	-0.0144	0.0020	0.0002	0.0002
rs3829054	8	61766431	T	C	0.6778	-0.0140	0.0021	-0.0001	0.0002
rs45577137	8	48651633	G	A	0.0451	0.0364	0.0052	-0.0002	0.0004
rs4734879	8	106583124	G	A	0.2765	-0.0213	0.0021	0.0001	0.0002
rs55964818	8	130605871	C	T	0.5677	-0.0365	0.0019	-0.0001	0.0002
rs59321109	8	66837444	T	C	0.0632	-0.0229	0.0039	-0.0002	0.0003
rs6468341	8	30279355	C	T	0.7428	0.0190	0.0022	0.0000	0.0002
rs67241230	8	68842927	G	T	0.4001	-0.0268	0.0020	0.0001	0.0002
rs6985508	8	142337734	A	G	0.3580	-0.0274	0.0021	0.0003	0.0002
rs6998846	8	9193341	A	G	0.7828	0.0154	0.0023	0.0003	0.0002
rs7005996	8	142241681	T	C	0.9060	0.0229	0.0035	0.0003	0.0003
rs73271394	8	100853860	C	T	0.0455	0.0255	0.0046	0.0000	0.0004
rs73562553	8	13177156	A	T	0.0965	-0.0179	0.0032	0.0003	0.0003
rs7816785	8	56797418	T	C	0.6058	-0.0212	0.0020	0.0001	0.0002
rs7846314	8	61650831	T	A	0.1873	0.0639	0.0025	-0.0001	0.0002
rs10760690	9	102281113	T	G	0.3740	0.0115	0.0020	-0.0003	0.0002
rs10811668	9	22164991	A	C	0.2085	-0.0135	0.0024	0.0002	0.0002
rs10818883	9	126610982	C	A	0.8649	-0.0157	0.0028	-0.0004	0.0002
rs10992394	9	95433830	A	G	0.2308	0.0139	0.0023	0.0003	0.0002
rs117468663	9	112745175	T	A	0.1339	0.0195	0.0029	0.0001	0.0002
rs12000252	9	73037963	G	A	0.5482	-0.0110	0.0020	-0.0001	0.0002
rs13291664	9	282738	G	A	0.1880	0.0293	0.0025	-0.0002	0.0002
rs1411424	9	113892963	A	G	0.5231	-0.0115	0.0019	0.0003	0.0002
rs17831500	9	116047472	C	A	0.2236	-0.0199	0.0023	0.0003	0.0002
rs1887428	9	4984530	C	G	0.6252	-0.0143	0.0020	0.0000	0.0002
rs2157770	9	136921464	G	A	0.2872	0.0210	0.0022	0.0004	0.0002
rs2519093	9	136141870	T	C	0.1847	-0.0401	0.0025	0.0002	0.0002
rs35782171	9	131391202	T	C	0.0449	-0.0274	0.0049	-0.0002	0.0004
rs3739873	9	34978431	A	G	0.2203	-0.0139	0.0023	0.0001	0.0002
rs3793537	9	35687556	C	G	0.2912	0.0126	0.0021	0.0005	0.0002
rs409801	9	4744743	C	T	0.5028	0.0211	0.0019	-0.0001	0.0002
rs4413892	9	139330158	A	G	0.2779	0.0337	0.0022	0.0004	0.0002
rs626416	9	79326680	C	G	0.7620	-0.0197	0.0023	0.0001	0.0002
rs6476883	9	4654774	A	G	0.4386	-0.0140	0.0019	0.0000	0.0002
rs72759286	9	126985858	C	T	0.1985	-0.0236	0.0024	-0.0003	0.0002

rs7852409	9	2621482	G	C	0.7651	0.0179	0.0024	-0.0002	0.0002
rs7866863	9	114657707	A	G	0.3414	0.0131	0.0020	-0.0001	0.0002
rs7869378	9	130768821	G	A	0.2444	0.0152	0.0023	0.0001	0.0002
rs796007	9	86577541	A	G	0.2528	-0.0207	0.0022	-0.0003	0.0002
rs9411293	9	139929015	G	C	0.3371	-0.0140	0.0021	-0.0003	0.0002
rs10509912	10	112029405	A	T	0.1349	-0.0211	0.0028	0.0004	0.0002
rs10786325	10	99068738	G	C	0.5959	0.0423	0.0020	0.0002	0.0002
rs10906393	10	13536512	T	A	0.5776	0.0172	0.0019	0.0000	0.0002
rs10995477	10	65010672	C	T	0.4735	-0.0304	0.0019	0.0002	0.0002
rs1250568	10	81045280	C	T	0.4351	-0.0138	0.0020	0.0000	0.0002
rs1412445	10	91002804	T	C	0.3381	0.0211	0.0020	0.0001	0.0002
rs1537746	10	26726606	C	T	0.3371	-0.0119	0.0020	0.0001	0.0002
rs1571956	10	30504708	A	G	0.6461	0.0137	0.0020	0.0001	0.0002
rs17113735	10	102896849	A	G	0.3271	0.0118	0.0021	0.0002	0.0002
rs180941	10	115720674	A	G	0.6252	-0.0130	0.0020	0.0002	0.0002
rs1885474	10	69566751	G	T	0.1031	-0.0235	0.0032	-0.0002	0.0003
rs1977289	10	96301907	C	T	0.4785	-0.0182	0.0019	0.0000	0.0002
rs2263608	10	113944940	A	T	0.7215	0.0153	0.0022	-0.0002	0.0002
rs2807742	10	28781367	A	G	0.7714	0.0326	0.0023	-0.0001	0.0002
rs35755883	10	89720292	A	T	0.0711	-0.0225	0.0037	-0.0002	0.0003
rs3747869	10	73520632	C	A	0.9009	0.0327	0.0033	-0.0003	0.0003
rs3781454	10	126348565	A	G	0.6779	0.0242	0.0021	-0.0003	0.0002
rs4751697	10	120897063	T	C	0.5677	-0.0132	0.0019	0.0000	0.0002
rs56278466	10	17875857	G	T	0.6365	-0.0138	0.0022	0.0003	0.0002
rs692594	10	18265893	C	G	0.4898	0.0125	0.0019	-0.0003	0.0002
rs72790862	10	44880260	C	T	0.3071	-0.0210	0.0021	-0.0001	0.0002
rs72825306	10	93748263	G	A	0.1675	0.0151	0.0026	0.0000	0.0002
rs7917772	10	104487443	A	G	0.6313	0.0161	0.0020	-0.0002	0.0002
rs79615245	10	121266907	C	T	0.0251	0.0377	0.0062	0.0003	0.0005
rs9419387	10	133758358	A	G	0.4052	-0.0124	0.0020	0.0003	0.0002
rs9804265	10	25214050	C	A	0.3670	-0.0400	0.0020	0.0003	0.0002
rs10743129	11	10130363	A	G	0.2986	-0.0145	0.0021	-0.0001	0.0002
rs10796828	11	69490346	G	T	0.6350	-0.0139	0.0020	0.0002	0.0002
rs10833024	11	3010390	T	C	0.3101	0.0163	0.0021	0.0002	0.0002
rs11039195	11	47375193	A	G	0.3174	0.0185	0.0021	-0.0002	0.0002
rs12226331	11	102070976	T	A	0.3529	-0.0122	0.0020	0.0003	0.0002
rs1451724	11	3856668	A	G	0.4700	0.0111	0.0019	0.0002	0.0002
rs1715429	11	118083664	G	A	0.7527	0.0194	0.0022	-0.0004	0.0002
rs174548	11	61571348	G	C	0.3130	-0.0239	0.0021	0.0001	0.0002
rs1783921	11	128094803	C	T	0.2073	0.0158	0.0024	0.0000	0.0002
rs2049045	11	27694241	C	G	0.1852	0.0145	0.0025	-0.0001	0.0002
rs2236658	11	122933132	C	T	0.0431	-0.0263	0.0048	-0.0003	0.0004
rs2282611	11	76154846	G	T	0.3199	-0.0126	0.0021	-0.0001	0.0002
rs2468832	11	18163882	A	C	0.2389	0.0152	0.0023	0.0001	0.0002
rs58984522	11	43490624	C	T	0.3461	-0.0110	0.0020	-0.0001	0.0002
rs617791	11	65702523	C	G	0.4825	0.0134	0.0019	0.0001	0.0002
rs61904448	11	113958121	C	T	0.2844	-0.0148	0.0022	-0.0004	0.0002
rs655231	11	60013857	A	G	0.5914	0.0143	0.0020	0.0001	0.0002
rs672058	11	116764021	T	C	0.8815	0.0164	0.0030	0.0006	0.0003
rs7105709	11	101787112	G	A	0.4411	-0.0108	0.0019	0.0001	0.0002
rs7115703	11	306920	A	T	0.5136	0.0444	0.0020	-0.0001	0.0002
rs7120300	11	8823493	T	C	0.7349	-0.0167	0.0022	0.0003	0.0002
rs72857653	11	11991690	T	C	0.1475	-0.0154	0.0027	-0.0003	0.0002
rs72919477	11	62042403	C	G	0.1161	-0.0178	0.0030	0.0004	0.0003
rs73000965	11	113982321	A	T	0.3157	0.0292	0.0021	-0.0002	0.0002
rs7934719	11	108341864	T	C	0.4135	0.0181	0.0020	-0.0001	0.0002
rs7939337	11	122519288	T	C	0.2644	-0.0184	0.0022	-0.0001	0.0002
rs7947419	11	134006964	T	C	0.0596	0.0225	0.0041	0.0001	0.0003

rs8705	11	128328913	A	G	0.3168	-0.0183	0.0021	-0.0001	0.0002
rs9988894	11	12876944	G	C	0.3235	0.0150	0.0021	0.0001	0.0002
rs1042725	12	66358347	T	C	0.4944	0.0128	0.0019	0.0001	0.0002
rs10849020	12	4332009	G	C	0.2101	-0.0241	0.0024	-0.0001	0.0002
rs11064881	12	120146925	A	G	0.0735	-0.0342	0.0037	-0.0003	0.0003
rs11104881	12	88843474	C	T	0.7017	-0.0259	0.0021	-0.0001	0.0002
rs1168669	12	122228694	C	T	0.8136	-0.0147	0.0025	0.0000	0.0002
rs1245035	12	64976049	A	C	0.6281	0.0163	0.0020	0.0000	0.0002
rs17041439	12	101873240	C	A	0.0566	0.0239	0.0042	0.0000	0.0004
rs28588142	12	10097609	T	C	0.1884	0.0152	0.0025	-0.0003	0.0002
rs3184504	12	111884608	C	T	0.5175	-0.0294	0.0019	0.0002	0.0002
rs35864914	12	108159191	T	C	0.3765	-0.0114	0.0020	0.0002	0.0002
rs3861100	12	50599219	G	A	0.3479	0.0181	0.0020	0.0005	0.0002
rs4761234	12	69732105	C	T	0.4837	0.0216	0.0019	-0.0002	0.0002
rs4842266	12	79951566	A	G	0.6891	-0.0123	0.0021	0.0003	0.0002
rs610578	12	121194565	G	A	0.6627	-0.0146	0.0020	0.0000	0.0002
rs61754230	12	72179446	T	C	0.0194	0.0400	0.0071	-0.0005	0.0006
rs61955089	12	123851372	C	T	0.0269	0.0437	0.0060	0.0007	0.0005
rs632887	12	3392351	G	A	0.4107	0.0143	0.0020	-0.0002	0.0002
rs706809	12	52294257	C	T	0.7772	0.0206	0.0024	0.0003	0.0002
rs7488780	12	20579392	C	G	0.2045	-0.0137	0.0024	-0.0001	0.0002
rs7969023	12	26803949	C	T	0.4057	-0.0114	0.0020	-0.0001	0.0002
rs150861794	13	109003805	T	C	0.0198	-0.0526	0.0075	-0.0001	0.0007
rs2260766	13	114186800	G	A	0.2820	0.0230	0.0021	-0.0001	0.0002
rs2296028	13	52345637	C	G	0.1750	-0.0153	0.0026	-0.0003	0.0002
rs556429	13	37487021	A	C	0.2468	0.0123	0.0022	-0.0001	0.0002
rs7139746	13	49784367	A	G	0.6780	-0.0123	0.0021	-0.0001	0.0002
rs76428106	13	28604007	C	T	0.0134	0.1045	0.0089	-0.0006	0.0007
rs76603681	13	28496468	A	G	0.0320	0.0408	0.0056	0.0003	0.0005
rs78738581	13	42843630	A	G	0.2319	0.0213	0.0023	0.0004	0.0002
rs7996207	13	50122681	A	G	0.6936	0.0194	0.0021	0.0002	0.0002
rs9508005	13	28789794	G	T	0.0945	-0.0199	0.0036	-0.0002	0.0003
rs9543219	13	73638478	T	C	0.5741	0.0128	0.0020	-0.0006	0.0002
rs10498635	14	93103309	T	C	0.1837	-0.0302	0.0025	-0.0001	0.0002
rs11625865	14	105644421	A	G	0.6103	-0.0153	0.0021	-0.0004	0.0002
rs12588718	14	101149605	C	G	0.6656	0.0145	0.0021	0.0001	0.0002
rs2038700	14	25461989	C	T	0.3943	0.0335	0.0020	0.0000	0.0002
rs2241621	14	81737076	C	A	0.5882	-0.0149	0.0020	-0.0002	0.0002
rs34765661	14	69852940	C	T	0.0653	0.0417	0.0039	-0.0004	0.0003
rs45490496	14	105272678	T	A	0.6124	0.0130	0.0021	0.0000	0.0002
rs4903580	14	77850978	T	C	0.4561	0.0165	0.0019	0.0000	0.0002
rs72664840	14	35596323	T	C	0.1790	0.0209	0.0025	-0.0001	0.0002
rs72731564	14	69280158	T	C	0.1870	0.0202	0.0025	0.0004	0.0002
rs8009601	14	69090756	T	C	0.0595	-0.0273	0.0041	-0.0005	0.0003
rs11073373	15	95152242	A	G	0.2464	0.0127	0.0022	0.0000	0.0002
rs112597175	15	77828079	A	G	0.3869	0.0129	0.0020	0.0001	0.0002
rs12909505	15	51104350	C	T	0.4286	-0.0214	0.0019	0.0003	0.0002
rs17184256	15	63785421	G	A	0.3112	0.0117	0.0021	0.0001	0.0002
rs2062250	15	64672002	A	G	0.9385	0.0470	0.0041	0.0004	0.0003
rs2955958	15	101916347	A	G	0.3152	0.0123	0.0021	0.0000	0.0002
rs4843073	15	86124419	T	C	0.6449	0.0137	0.0020	-0.0002	0.0002
rs4924450	15	40597229	A	G	0.7055	0.0138	0.0022	0.0002	0.0002
rs67175901	15	101748227	T	C	0.1075	0.0436	0.0032	-0.0001	0.0003
rs7183988	15	91428589	G	T	0.5265	-0.0170	0.0020	-0.0002	0.0002
rs72726027	15	42248826	C	T	0.1117	-0.0432	0.0031	-0.0002	0.0003
rs7496362	15	65758874	G	C	0.3664	0.0175	0.0020	0.0000	0.0002
rs780142	15	62797964	G	T	0.2742	0.0127	0.0022	-0.0001	0.0002
rs8030089	15	75290377	T	C	0.1954	-0.0189	0.0024	0.0001	0.0002

rs11574938	16	30485393	C	G	0.5198	0.0203	0.0019	0.0000	0.0002
rs11644125	16	57058974	T	C	0.5982	-0.0145	0.0020	0.0002	0.0002
rs11648664	16	53122486	A	G	0.4021	-0.0142	0.0020	0.0000	0.0002
rs12923918	16	24844403	G	A	0.5094	-0.0105	0.0019	-0.0001	0.0002
rs12927351	16	74596618	A	C	0.1986	0.0216	0.0024	0.0002	0.0002
rs12930850	16	81602212	G	A	0.5371	0.0190	0.0019	0.0000	0.0002
rs1362623	16	49885785	T	C	0.2078	0.0183	0.0024	0.0001	0.0002
rs16958642	16	11864032	A	G	0.0826	0.0223	0.0035	-0.0003	0.0003
rs305082	16	85936978	C	T	0.1723	0.0366	0.0026	-0.0001	0.0002
rs35929659	16	2165630	C	T	0.1798	-0.0196	0.0026	0.0002	0.0002
rs4258607	16	81867410	T	C	0.3220	-0.0125	0.0021	0.0000	0.0002
rs4984803	16	1349929	A	G	0.5956	-0.0175	0.0021	0.0002	0.0002
rs61739285	16	27480797	T	C	0.0335	-0.0401	0.0054	-0.0002	0.0005
rs6500550	16	3746241	T	C	0.3043	-0.0224	0.0021	-0.0003	0.0002
rs72819343	16	89234723	G	A	0.0578	0.0321	0.0043	-0.0003	0.0004
rs74250734	16	50212589	G	T	0.0940	-0.0213	0.0033	-0.0003	0.0003
rs9936870	16	87827042	C	G	0.3939	-0.0115	0.0021	0.0000	0.0002
rs10852834	17	16174506	G	C	0.4780	-0.0371	0.0019	0.0003	0.0002
rs11078004	17	80563300	G	A	0.3256	0.0122	0.0021	0.0000	0.0002
rs11651011	17	21095654	T	C	0.3403	0.0117	0.0020	0.0001	0.0002
rs11653826	17	27653016	T	C	0.0983	0.0251	0.0033	0.0001	0.0003
rs12946510	17	37912377	T	C	0.4729	-0.0594	0.0019	-0.0001	0.0002
rs147180766	17	37835355	A	C	0.0182	-0.0636	0.0074	-0.0005	0.0006
rs149157044	17	56816881	T	C	0.0394	-0.0312	0.0051	-0.0005	0.0004
rs16961474	17	17146619	A	G	0.1368	-0.0170	0.0028	-0.0002	0.0002
rs2001613	17	75383679	T	C	0.4721	0.0120	0.0020	0.0002	0.0002
rs2250320	17	28128657	C	G	0.4963	-0.0198	0.0019	-0.0002	0.0002
rs2665405	17	57875292	A	G	0.5484	0.0295	0.0019	0.0000	0.0002
rs33983467	17	46102904	A	G	0.2879	0.0138	0.0021	0.0000	0.0002
rs56301633	17	44077850	A	G	0.2243	0.0237	0.0023	0.0004	0.0002
rs56378716	17	56356502	G	A	0.0127	0.1292	0.0087	-0.0009	0.0007
rs61759532	17	7240391	T	C	0.2421	-0.0232	0.0023	-0.0003	0.0002
rs668799	17	40716235	T	C	0.2761	0.0125	0.0021	0.0001	0.0002
rs7225843	17	2001825	C	T	0.2032	-0.0221	0.0024	0.0003	0.0002
rs72901753	17	76244283	C	G	0.3269	-0.0145	0.0021	0.0001	0.0002
rs74480102	17	7742601	A	G	0.0417	-0.0463	0.0049	0.0004	0.0004
rs74725931	17	38196327	C	T	0.0374	0.0673	0.0053	-0.0003	0.0004
rs749780	17	72699384	A	C	0.7319	0.0189	0.0022	0.0002	0.0002
rs79730542	17	37298789	C	T	0.0302	-0.0426	0.0058	0.0001	0.0005
rs820384	17	73755392	C	G	0.6632	0.0119	0.0021	0.0002	0.0002
rs9905106	17	1373518	C	T	0.7336	0.0228	0.0022	0.0000	0.0002
rs1108167	18	60163025	G	A	0.2840	-0.0159	0.0021	0.0000	0.0002
rs11664534	18	43724520	T	C	0.5250	-0.0160	0.0019	0.0002	0.0002
rs2202555	18	61559135	C	T	0.1886	0.0135	0.0025	0.0002	0.0002
rs303753	18	21074922	A	G	0.3451	-0.0228	0.0020	0.0000	0.0002
rs4468717	18	3457606	T	C	0.0777	-0.0250	0.0036	0.0006	0.0003
rs4940320	18	51776316	A	T	0.3002	0.0169	0.0021	0.0001	0.0002
rs7235882	18	19684119	T	C	0.0935	0.0233	0.0033	0.0000	0.0003
rs72973711	18	74072245	T	A	0.0669	-0.0365	0.0039	-0.0007	0.0003
rs75429956	18	74258187	G	T	0.1737	0.0153	0.0026	-0.0001	0.0002
rs763362	18	67531797	G	A	0.3987	0.0156	0.0020	0.0004	0.0002
rs78285907	18	21622003	T	A	0.0890	0.0219	0.0035	-0.0001	0.0003
rs8084255	18	48144754	T	C	0.3753	0.0213	0.0020	0.0000	0.0002
rs9965539	18	41973779	A	G	0.1337	0.0311	0.0029	0.0000	0.0002
rs11668882	19	54675097	C	T	0.4393	-0.0121	0.0020	-0.0001	0.0002
rs11673093	19	45742094	A	G	0.2599	-0.0488	0.0022	0.0000	0.0002
rs1985157	19	18513594	C	T	0.4104	0.0265	0.0020	0.0001	0.0002
rs2074959	19	2111649	C	T	0.2292	0.0147	0.0024	-0.0002	0.0002

rs2194067	19	35464727	T	C	0.4154	0.0110	0.0020	-0.0001	0.0002
rs28540102	19	4975763	C	T	0.6586	0.0171	0.0021	-0.0001	0.0002
rs31725	19	39914308	T	C	0.4378	-0.0142	0.0020	0.0000	0.0002
rs386243	19	49127490	T	C	0.2501	-0.0264	0.0023	0.0003	0.0002
rs4760	19	44153100	G	A	0.1534	-0.0752	0.0027	-0.0002	0.0002
rs4804496	19	10338764	G	A	0.8076	-0.0266	0.0026	-0.0002	0.0002
rs4805881	19	33896432	C	A	0.6653	-0.0144	0.0021	0.0000	0.0002
rs4807440	19	1026477	T	G	0.6376	0.0224	0.0021	0.0003	0.0002
rs4808683	19	17862925	G	C	0.4582	-0.0123	0.0020	0.0000	0.0002
rs56048141	19	11317744	T	C	0.0194	0.0471	0.0076	-0.0004	0.0006
rs56408111	19	19793545	C	T	0.0837	0.0311	0.0035	0.0000	0.0003
rs571497	19	7827830	A	G	0.1546	-0.0379	0.0027	-0.0004	0.0002
rs61658003	19	55544203	A	C	0.8440	0.0159	0.0027	0.0002	0.0002
rs12481262	20	8126920	C	T	0.4600	-0.0146	0.0020	-0.0003	0.0002
rs13043334	20	48991215	C	A	0.6704	-0.0127	0.0021	0.0002	0.0002
rs143003731	20	47936696	T	C	0.0087	-0.0863	0.0113	0.0030	0.0010
rs156334	20	1827809	T	C	0.4293	-0.0118	0.0020	-0.0001	0.0002
rs1800961	20	43042364	T	C	0.0310	-0.0519	0.0056	-0.0001	0.0005
rs2179593	20	42660286	A	C	0.7159	-0.0123	0.0021	0.0000	0.0002
rs2254458	20	1551485	T	C	0.6446	-0.0134	0.0021	0.0000	0.0002
rs2315008	20	62343956	G	T	0.6670	0.0164	0.0021	0.0002	0.0002
rs328491	20	55969492	T	C	0.5983	-0.0134	0.0020	0.0001	0.0002
rs4812447	20	39272620	G	A	0.4404	0.0171	0.0019	0.0000	0.0002
rs619450	20	2894790	C	T	0.5032	0.0142	0.0020	0.0002	0.0002
rs78358631	20	10719307	T	A	0.0731	0.0232	0.0037	0.0000	0.0003
rs1788493	21	44469063	T	C	0.4998	0.0132	0.0020	0.0000	0.0002
rs1803439	21	38885442	G	A	0.3464	-0.0124	0.0020	0.0000	0.0002
rs2096507	21	47941916	A	G	0.4692	-0.0139	0.0019	0.0000	0.0002
rs2824372	21	18947037	C	G	0.2027	-0.0155	0.0024	0.0000	0.0002
rs2832258	21	30582732	G	A	0.7872	-0.0137	0.0024	0.0000	0.0002
rs9977672	21	40463283	A	G	0.2588	-0.0223	0.0022	0.0001	0.0002
rs139386	22	39530859	C	T	0.4451	-0.0109	0.0020	0.0000	0.0002
rs35284073	22	17624425	A	G	0.2170	0.0147	0.0024	0.0000	0.0002
rs41433144	22	17569993	A	T	0.0429	-0.0346	0.0049	-0.0005	0.0004
rs4347951	22	29513326	C	T	0.3108	0.0118	0.0021	-0.0002	0.0002
rs47341	22	43560763	T	C	0.3959	0.0130	0.0021	0.0000	0.0002
rs5747308	22	18133500	C	A	0.5045	0.0221	0.0019	0.0001	0.0002
rs5753576	22	31757735	T	C	0.4803	-0.0178	0.0019	-0.0001	0.0002
rs5760147	22	24334948	C	A	0.3810	-0.0126	0.0020	0.0002	0.0002
rs738408	22	44324730	T	C	0.2169	-0.0161	0.0024	0.0001	0.0002
