# Supplementary material for: Blood cell traits and risk of glaucoma: A two-sample mendelian randomization study
Source: Front Genet. 2023 Apr 12;14:1142773. doi: 10.3389/fgene.2023.1142773 (PMC10130872; doi:10.3389/fgene.2023.1142773)
Supplement: Supplementary file 1 [file DataSheet1.ZIP › eTable 4. Monocyte cell count exposure SNPs and their association with glaucoma..pdf]

eTable 4. Monocyte cell count exposure SNPs and their association with glaucoma.
Chr = chromosome; POS = position ; EA = effect allele; NEA = non-effect allele; EAF = effect allele frequency; SE = standard error.

SNP	Chr	POS	EA	NEA	EAF	Monocyte Cell Count		Glaucoma	
						Beta	SE	Beta	SE
rs10888850	1	54883715	G	A	0.4772	-0.0154	0.0019	0.0001	0.0002
rs1115163	1	19976782	G	A	0.4756	0.0108	0.0019	0.0001	0.0002
rs11247908	1	26638222	A	G	0.1729	-0.0292	0.0025	0.0002	0.0002
rs114425738	1	92171673	C	G	0.0135	0.2675	0.0083	0.0003	0.0007
rs115340020	1	92766438	A	G	0.0272	-0.0943	0.0058	-0.0002	0.0005
rs11547648	1	101106268	T	G	0.4632	-0.0237	0.0019	0.0000	0.0002
rs11590380	1	185400939	C	T	0.6794	0.0247	0.0021	0.0002	0.0002
rs12022930	1	221140832	C	T	0.5706	0.0167	0.0019	-0.0002	0.0002
rs12025621	1	46228391	A	G	0.4573	-0.0124	0.0019	0.0001	0.0002
rs12075	1	159175354	A	G	0.5795	0.0270	0.0019	-0.0002	0.0002
rs1212036	1	23690216	T	C	0.1136	-0.0177	0.0029	0.0005	0.0003
rs12144117	1	9182141	T	C	0.2154	-0.0141	0.0023	-0.0004	0.0002
rs12742428	1	67426487	G	A	0.6506	-0.0167	0.0020	-0.0001	0.0002
rs12747432	1	198566554	T	G	0.0666	-0.0240	0.0037	0.0001	0.0003
rs12756133	1	77949824	A	G	0.2038	0.0129	0.0023	0.0000	0.0002
rs139862824	1	161596032	C	G	0.0149	-0.0539	0.0082	0.0008	0.0007
rs150006408	1	150658444	C	T	0.0114	0.0797	0.0088	0.0020	0.0008
rs16837408	1	156425048	T	C	0.1561	0.0190	0.0027	-0.0002	0.0002
rs16844299	1	235109359	C	T	0.2017	-0.0141	0.0023	0.0001	0.0002
rs17387886	1	43419705	C	G	0.1736	-0.0278	0.0025	-0.0006	0.0002
rs17405580	1	204273188	C	T	0.2659	-0.0162	0.0021	-0.0001	0.0002
rs1772143	1	205799987	A	T	0.4132	0.0199	0.0019	-0.0002	0.0002
rs188393352	1	28254136	G	C	0.0101	0.0865	0.0102	0.0003	0.0009
rs1933295	1	62107021	G	A	0.7766	-0.0244	0.0023	0.0002	0.0002
rs2001814	1	200101433	T	C	0.5113	-0.0117	0.0019	0.0000	0.0002
rs2064897	1	35305803	A	G	0.2279	0.0132	0.0022	-0.0003	0.0002
rs2072732	1	2980277	C	T	0.1926	0.0213	0.0024	0.0001	0.0002
rs2406105	1	47851881	T	C	0.8488	0.0151	0.0026	0.0005	0.0002
rs2433279	1	93907909	G	T	0.8786	0.0228	0.0029	-0.0003	0.0003
rs2782640	1	44009033	T	C	0.6227	-0.0110	0.0019	-0.0001	0.0002
rs2786487	1	42368339	C	G	0.5586	0.0148	0.0019	-0.0003	0.0002
rs2807844	1	221047581	T	C	0.1304	-0.0180	0.0028	0.0002	0.0002
rs284324	1	10724236	A	G	0.4953	0.0212	0.0020	0.0001	0.0002
rs3026940	1	159130696	T	A	0.0081	-0.0902	0.0117	0.0005	0.0010
rs3027063	1	159186781	T	C	0.3549	0.0148	0.0019	0.0004	0.0002
rs333947	1	110470764	A	G	0.1502	0.0368	0.0026	-0.0001	0.0002
rs3795503	1	180905694	T	C	0.3145	-0.0189	0.0020	0.0000	0.0002
rs41268099	1	41328644	A	G	0.1109	0.0290	0.0030	0.0000	0.0003
rs41313381	1	79411968	A	C	0.0305	0.0430	0.0053	-0.0001	0.0005
rs4269828	1	235257838	G	A	0.5601	0.0122	0.0019	0.0001	0.0002
rs4335411	1	249191706	A	G	0.7625	-0.0174	0.0024	-0.0002	0.0002
rs4568851	1	101237172	A	G	0.4315	-0.0105	0.0019	-0.0002	0.0002
rs4626924	1	234909298	T	C	0.5510	-0.0183	0.0019	0.0000	0.0002
rs4656291	1	161153836	A	G	0.0747	-0.0215	0.0036	-0.0002	0.0003
rs485742	1	110366044	C	T	0.3283	-0.0149	0.0020	-0.0001	0.0002
rs4987353	1	169666987	A	G	0.3104	-0.0219	0.0020	-0.0001	0.0002
rs506166	1	53327330	C	T	0.6639	0.0174	0.0020	0.0001	0.0002
rs55646278	1	162130805	C	A	0.1175	-0.0192	0.0029	0.0002	0.0003
rs55857101	1	160651980	T	G	0.1813	0.0155	0.0025	-0.0003	0.0002
rs60752752	1	153339782	G	A	0.1180	0.0250	0.0030	0.0001	0.0003
rs61165644	1	244487940	G	A	0.1408	-0.0264	0.0027	0.0002	0.0002
rs61850655	1	225887954	T	C	0.1162	-0.0279	0.0029	0.0005	0.0003

rs6429432	1	236107241	C	A	0.8915	-0.0797	0.0030	0.0004	0.0003
rs6429438	1	243987674	G	A	0.8864	0.0208	0.0030	-0.0001	0.0003
rs6664626	1	66128239	T	G	0.1430	0.0189	0.0029	-0.0002	0.0002
rs6665912	1	150544093	C	T	0.2227	-0.0721	0.0023	0.0000	0.0002
rs6687430	1	10633245	A	G	0.5434	0.0119	0.0019	-0.0001	0.0002
rs67224956	1	118154575	C	T	0.1703	0.0269	0.0025	0.0003	0.0002
rs701905	1	212553111	C	G	0.3523	0.0217	0.0020	0.0002	0.0002
rs72675573	1	56636881	T	C	0.3665	0.0160	0.0019	-0.0001	0.0002
rs74765249	1	31194784	T	C	0.2815	0.0128	0.0021	-0.0001	0.0002
rs7516138	1	9711642	G	A	0.3911	-0.0226	0.0019	-0.0002	0.0002
rs7522307	1	207998783	C	G	0.1241	-0.0233	0.0029	0.0003	0.0002
rs7524046	1	9307033	A	G	0.2513	0.0147	0.0022	0.0001	0.0002
rs7532966	1	89955188	C	T	0.4994	0.0168	0.0019	0.0001	0.0002
rs80036648	1	93912771	G	A	0.0167	0.1549	0.0076	-0.0007	0.0007
rs10164853	2	158481992	G	A	0.0771	0.0194	0.0035	-0.0002	0.0003
rs10197805	2	225750753	C	T	0.1566	-0.0416	0.0026	0.0000	0.0002
rs10201197	2	145820213	C	T	0.3329	0.0120	0.0020	-0.0002	0.0002
rs10202404	2	103177414	T	C	0.5643	-0.0122	0.0019	-0.0001	0.0002
rs1027272	2	143797072	T	C	0.1274	-0.0193	0.0028	-0.0005	0.0002
rs1031091	2	24680948	G	A	0.3362	-0.0109	0.0020	0.0000	0.0002
rs1047891	2	211540507	A	C	0.3150	-0.0118	0.0020	0.0002	0.0002
rs11680095	2	181825956	T	C	0.5917	0.0224	0.0019	0.0003	0.0002
rs11680709	2	220050707	A	G	0.5934	0.0147	0.0019	0.0000	0.0002
rs12468071	2	188767101	C	T	0.2624	0.0118	0.0021	-0.0002	0.0002
rs13007974	2	227283285	T	C	0.3736	0.0136	0.0019	0.0002	0.0002
rs13013794	2	102286492	T	G	0.4349	0.0117	0.0019	-0.0001	0.0002
rs13026184	2	64930786	G	C	0.2421	0.0182	0.0022	0.0000	0.0002
rs13032786	2	148803672	G	C	0.2999	0.0203	0.0020	-0.0003	0.0002
rs1430055	2	16680789	A	G	0.7027	-0.0118	0.0021	-0.0004	0.0002
rs150449635	2	111752151	C	T	0.0225	0.1402	0.0067	0.0001	0.0006
rs155109	2	182352679	G	A	0.3207	-0.0211	0.0020	0.0001	0.0002
rs155127	2	182305701	C	G	0.3483	0.0596	0.0020	0.0002	0.0002
rs17860428	2	202151400	A	G	0.1795	-0.0156	0.0024	0.0000	0.0002
rs1863219	2	160418171	G	C	0.5123	0.0184	0.0019	0.0000	0.0002
rs2278784	2	168997097	G	A	0.2773	-0.0118	0.0021	-0.0002	0.0002
rs2280244	2	68979302	A	G	0.3044	0.0119	0.0020	-0.0001	0.0002
rs2368280	2	183046558	G	A	0.2040	0.0128	0.0023	0.0003	0.0002
rs28498283	2	43360065	T	A	0.2576	0.0211	0.0022	0.0001	0.0002
rs290773	2	102125930	A	G	0.3450	-0.0200	0.0020	-0.0002	0.0002
rs30102	2	237817538	C	G	0.3170	-0.0130	0.0020	-0.0002	0.0002
rs3111414	2	8443859	G	C	0.7944	0.0174	0.0023	-0.0002	0.0002
rs34236350	2	241568326	T	C	0.1825	-0.0322	0.0025	-0.0003	0.0002
rs3789062	2	111917317	T	C	0.2454	-0.0201	0.0022	0.0000	0.0002
rs4470337	2	228293381	G	A	0.5961	-0.0119	0.0019	-0.0001	0.0002
rs4525653	2	181793804	A	G	0.6607	0.0120	0.0020	0.0001	0.0002
rs4669869	2	12898460	C	T	0.4451	0.0204	0.0019	-0.0001	0.0002
rs4907230	2	96855241	A	G	0.3235	0.0156	0.0020	0.0001	0.0002
rs5022713	2	162924483	G	C	0.3678	0.0123	0.0019	0.0001	0.0002
rs62176784	2	169744551	G	A	0.3195	0.0226	0.0020	-0.0003	0.0002
rs6434817	2	196914283	G	A	0.7181	0.0116	0.0021	-0.0001	0.0002
rs647137	2	31463850	A	G	0.6975	0.0205	0.0020	0.0000	0.0002
rs6545873	2	61733012	C	T	0.6105	-0.0153	0.0019	0.0001	0.0002
rs6706095	2	46075677	G	T	0.7600	0.0193	0.0022	0.0001	0.0002
rs6736362	2	219115108	T	C	0.5591	0.0222	0.0019	0.0000	0.0002
rs6753534	2	27752871	T	C	0.5622	0.0129	0.0019	0.0000	0.0002
rs72836307	2	111776154	T	C	0.1564	-0.0409	0.0026	0.0001	0.0002
rs73955669	2	128656804	A	G	0.1974	0.0138	0.0024	0.0003	0.0002
rs75475627	2	54787592	G	C	0.0767	0.0241	0.0036	0.0000	0.0003

rs7569084	2	65656969	T	C	0.5838	0.0276	0.0019	0.0000	0.0002
rs7572278	2	8563029	A	T	0.2068	0.0217	0.0024	0.0001	0.0002
rs7574456	2	136890059	T	C	0.7400	-0.0403	0.0021	0.0000	0.0002
rs7579497	2	71677946	A	G	0.1856	-0.0137	0.0024	0.0000	0.0002
rs7593080	2	86145787	T	C	0.5741	0.0107	0.0019	0.0005	0.0002
rs7608128	2	137007943	T	G	0.1812	-0.0195	0.0024	-0.0005	0.0002
rs78218855	2	43556575	T	C	0.0684	0.0220	0.0037	0.0003	0.0003
rs78655702	2	182303729	T	C	0.0498	-0.0590	0.0043	-0.0001	0.0004
rs8207	2	170493863	G	A	0.2667	0.0135	0.0021	0.0000	0.0002
rs9247	2	234113301	T	C	0.1903	0.0168	0.0025	0.0001	0.0002
rs10804681	3	141660675	T	A	0.8433	-0.0184	0.0026	-0.0001	0.0002
rs10935473	3	98416900	T	G	0.4415	0.0250	0.0019	-0.0001	0.0002
rs11713164	3	37892775	T	C	0.1621	0.0164	0.0025	0.0002	0.0002
rs11713343	3	128185399	A	G	0.1966	-0.0263	0.0024	-0.0001	0.0002
rs12485444	3	188135783	A	T	0.5438	-0.0235	0.0019	-0.0001	0.0002
rs13081337	3	40972152	A	G	0.1548	-0.0141	0.0026	0.0000	0.0002
rs13094390	3	71817778	G	A	0.1160	-0.0208	0.0029	0.0003	0.0003
rs1466684	3	151046308	A	G	0.8240	0.0155	0.0024	-0.0003	0.0002
rs16831132	3	159923303	T	C	0.3974	-0.0136	0.0019	-0.0001	0.0002
rs17078348	3	45847241	G	A	0.0907	-0.0267	0.0033	0.0004	0.0003
rs17295246	3	123105721	A	G	0.2248	-0.0256	0.0022	0.0001	0.0002
rs1822534	3	12266804	G	A	0.3940	-0.0310	0.0019	0.0000	0.0002
rs2018092	3	185903842	C	T	0.6150	-0.0115	0.0019	-0.0001	0.0002
rs2213290	3	46406367	T	C	0.4078	0.0356	0.0019	0.0000	0.0002
rs2245626	3	128287687	G	A	0.6764	0.0747	0.0020	0.0004	0.0002
rs231988	3	172276979	C	T	0.8682	0.0258	0.0029	0.0003	0.0002
rs2371108	3	27757018	T	G	0.3883	0.0106	0.0019	0.0000	0.0002
rs35902169	3	119926380	T	A	0.3319	0.0134	0.0020	-0.0002	0.0002
rs3732378	3	39307162	A	G	0.1727	0.0545	0.0025	0.0005	0.0002
rs4678272	3	136610630	T	G	0.6895	0.0174	0.0020	0.0001	0.0002
rs4683345	3	42886642	A	G	0.3855	-0.0578	0.0019	0.0001	0.0002
rs62256003	3	53283952	T	A	0.2110	-0.0153	0.0023	0.0001	0.0002
rs6800122	3	141249398	T	C	0.3997	-0.0218	0.0019	-0.0002	0.0002
rs68094955	3	128131170	T	C	0.0496	0.0363	0.0045	-0.0005	0.0004
rs683194	3	107299698	G	A	0.3010	-0.0152	0.0020	0.0001	0.0002
rs7426833	3	46728904	A	C	0.3073	-0.0172	0.0021	-0.0002	0.0002
rs7626444	3	196504902	C	G	0.4214	-0.0287	0.0019	0.0000	0.0002
rs7633965	3	168864643	C	A	0.9293	0.0250	0.0037	0.0000	0.0003
rs839228	3	58038605	T	C	0.3720	-0.0117	0.0019	0.0002	0.0002
rs869785	3	24347800	C	T	0.6696	-0.0172	0.0020	-0.0002	0.0002
rs922857	3	128741071	C	G	0.1063	0.0212	0.0033	0.0004	0.0003
rs9809116	3	72397279	G	A	0.4088	0.0174	0.0019	0.0001	0.0002
rs9846508	3	142815523	G	A	0.3891	0.0113	0.0019	0.0001	0.0002
rs10518329	4	120401835	G	A	0.3307	-0.0137	0.0020	0.0002	0.0002
rs113172748	4	79639615	G	A	0.6710	0.0157	0.0020	0.0000	0.0002
rs11723621	4	72615362	G	A	0.2908	-0.0132	0.0021	0.0000	0.0002
rs141936164	4	103401723	G	A	0.3508	-0.0187	0.0020	-0.0001	0.0002
rs1425553	4	185396920	A	G	0.5786	-0.0111	0.0019	-0.0002	0.0002
rs144317085	4	105806108	T	A	0.0342	0.0618	0.0052	-0.0003	0.0005
rs17005891	4	83547862	A	G	0.1844	-0.0510	0.0024	0.0000	0.0002
rs2059489	4	153002784	A	G	0.4425	-0.0135	0.0019	-0.0002	0.0002
rs2174326	4	90223611	G	A	0.4529	0.0108	0.0019	0.0002	0.0002
rs2609261	4	89835485	G	A	0.7864	0.0137	0.0023	0.0002	0.0002
rs2711981	4	39039258	T	C	0.6631	-0.0216	0.0020	-0.0001	0.0002
rs2714901	4	148247482	C	T	0.8067	-0.0146	0.0024	-0.0001	0.0002
rs28723530	4	106426718	A	C	0.1346	-0.0153	0.0028	-0.0003	0.0002
rs4240356	4	145042250	G	C	0.5505	-0.0235	0.0019	-0.0002	0.0002
rs4558856	4	154389786	G	C	0.3455	0.0128	0.0020	0.0004	0.0002

rs4566648	4	84159219	G	T	0.3420	-0.0254	0.0020	0.0000	0.0002
rs56058420	4	185237116	G	A	0.3562	0.0117	0.0020	-0.0001	0.0002
rs6856799	4	38081983	C	T	0.3150	-0.0111	0.0020	0.0001	0.0002
rs723585	4	55503194	G	A	0.4834	-0.0165	0.0019	0.0002	0.0002
rs72720206	4	146105777	A	G	0.3269	-0.0162	0.0020	0.0001	0.0002
rs73809166	4	36305937	C	T	0.0231	0.0465	0.0064	-0.0009	0.0006
rs76424165	4	157727919	T	C	0.0842	-0.0250	0.0034	-0.0001	0.0003
rs871134	4	7044380	T	C	0.5711	0.0383	0.0019	-0.0003	0.0002
rs929446	4	110883344	T	C	0.4054	-0.0182	0.0019	-0.0002	0.0002
rs9637714	4	57731446	C	T	0.2890	-0.0163	0.0021	-0.0003	0.0002
rs9992013	4	2259463	G	C	0.1365	0.0224	0.0028	0.0008	0.0002
rs10478058	5	110962584	G	A	0.1970	0.0148	0.0024	0.0000	0.0002
rs11242109	5	131677047	T	G	0.4782	-0.0225	0.0019	-0.0004	0.0002
rs116619972	5	32214314	A	G	0.0265	-0.0403	0.0059	-0.0001	0.0005
rs11954859	5	60624958	A	C	0.4985	0.0112	0.0019	0.0002	0.0002
rs11957478	5	159249469	G	T	0.0518	0.0247	0.0042	0.0001	0.0004
rs12332674	5	132322852	G	T	0.1524	-0.0165	0.0026	-0.0001	0.0002
rs13154298	5	156754659	T	C	0.1198	-0.0166	0.0029	-0.0002	0.0003
rs13167280	5	1280477	A	G	0.1281	0.0174	0.0030	0.0005	0.0003
rs17656204	5	149501803	T	C	0.3220	-0.0542	0.0020	-0.0001	0.0002
rs190982	5	88223420	A	G	0.6027	-0.0124	0.0019	-0.0002	0.0002
rs2338021	5	71749094	C	T	0.8797	-0.0317	0.0029	-0.0003	0.0003
rs2548257	5	100162317	A	C	0.6530	0.0129	0.0020	-0.0002	0.0002
rs28722705	5	55453942	T	A	0.1490	0.0343	0.0026	0.0003	0.0002
rs31196	5	158300798	A	C	0.5723	-0.0119	0.0019	-0.0001	0.0002
rs31243	5	75594360	G	A	0.0963	-0.0226	0.0032	0.0001	0.0003
rs329125	5	133871101	A	C	0.2071	-0.0166	0.0023	0.0001	0.0002
rs360017	5	173207353	G	A	0.7752	-0.0245	0.0023	-0.0003	0.0002
rs445611	5	35396119	G	A	0.8447	0.0160	0.0026	0.0002	0.0002
rs4865956	5	54882505	A	T	0.6963	-0.0189	0.0020	-0.0003	0.0002
rs60689150	5	90314241	A	G	0.0285	0.0310	0.0057	0.0001	0.0005
rs6579771	5	149482262	T	C	0.2686	0.0336	0.0021	0.0000	0.0002
rs6865582	5	78201833	G	C	0.4611	-0.0110	0.0019	0.0001	0.0002
rs6869021	5	111039017	T	C	0.3133	-0.0169	0.0020	0.0000	0.0002
rs6883116	5	179230321	C	T	0.4173	-0.0180	0.0019	-0.0001	0.0002
rs72800905	5	140004909	G	C	0.2228	0.0165	0.0022	-0.0001	0.0002
rs74735005	5	57202727	C	T	0.2065	-0.0170	0.0023	-0.0001	0.0002
rs7704551	5	123883857	T	C	0.2825	0.0144	0.0021	-0.0001	0.0002
rs80027325	5	150613352	C	T	0.0348	-0.0297	0.0051	0.0005	0.0004
rs964752	5	17399899	G	C	0.5399	-0.0124	0.0019	0.0001	0.0002
rs1008251	6	16727460	C	T	0.5098	-0.0167	0.0019	0.0001	0.0002
rs10948314	6	46911131	G	A	0.1029	-0.0336	0.0031	0.0003	0.0003
rs115202835	6	45533239	A	G	0.0677	0.0272	0.0038	0.0003	0.0003
rs12212674	6	22087074	A	T	0.5672	0.0147	0.0019	0.0000	0.0002
rs12530071	6	2886067	C	T	0.2246	-0.0147	0.0022	0.0002	0.0002
rs1283945	6	44572306	C	T	0.9756	-0.0347	0.0061	0.0013	0.0005
rs13207265	6	163787058	C	A	0.3613	-0.0113	0.0020	0.0000	0.0002
rs13220522	6	26316295	A	G	0.0997	-0.0505	0.0031	-0.0005	0.0003
rs138437628	6	144362698	C	T	0.0959	-0.0223	0.0032	-0.0001	0.0003
rs149110519	6	144385777	T	C	0.0360	0.0746	0.0051	0.0001	0.0004
rs1970364	6	113969527	T	C	0.2648	0.0193	0.0021	0.0003	0.0002
rs1998044	6	161097865	A	G	0.1500	0.0156	0.0026	0.0001	0.0002
rs2239707	6	31525319	T	C	0.6797	-0.0330	0.0020	0.0000	0.0002
rs2273215	6	170586082	A	G	0.4582	-0.0181	0.0020	-0.0003	0.0002
rs2614263	6	135780717	C	A	0.5460	0.0180	0.0019	0.0003	0.0002
rs2797670	6	137571613	A	G	0.6473	-0.0127	0.0020	0.0000	0.0002
rs2817441	6	156938699	T	C	0.2793	-0.0217	0.0021	0.0002	0.0002
rs3012415	6	170489464	T	C	0.8228	0.0203	0.0025	0.0002	0.0002

rs35880697	6	32433759	T	C	0.1552	0.0390	0.0027	0.0005	0.0002
rs36057735	6	31319923	G	C	0.1960	-0.0725	0.0024	-0.0004	0.0002
rs3761986	6	6680510	C	T	0.3943	-0.0121	0.0020	0.0000	0.0002
rs56007794	6	41990827	T	A	0.2443	0.0391	0.0022	-0.0001	0.0002
rs56217718	6	33096776	T	C	0.0704	0.0218	0.0036	-0.0003	0.0003
rs644492	6	53444319	G	A	0.1959	-0.0201	0.0024	0.0000	0.0002
rs67866855	6	20839501	C	G	0.0816	-0.0194	0.0034	-0.0001	0.0003
rs6902600	6	135035892	G	A	0.2065	0.0219	0.0023	-0.0001	0.0002
rs6912539	6	137108757	G	A	0.6233	-0.0158	0.0019	-0.0002	0.0002
rs6917549	6	113386660	G	A	0.1971	-0.0179	0.0024	0.0003	0.0002
rs6918882	6	21818078	T	C	0.1544	0.0170	0.0026	0.0001	0.0002
rs707793	6	10520009	C	T	0.5313	-0.0303	0.0019	0.0000	0.0002
rs73003601	6	152943434	G	A	0.1882	0.0154	0.0024	-0.0004	0.0002
rs7776054	6	135418916	G	A	0.2608	-0.0379	0.0021	0.0001	0.0002
rs79894332	6	44591721	A	G	0.1005	-0.0482	0.0031	0.0000	0.0003
rs915125	6	82463376	T	C	0.2822	0.0338	0.0021	0.0001	0.0002
rs9277946	6	33194717	T	C	0.1378	0.0229	0.0027	0.0002	0.0002
rs9358531	6	22309630	G	T	0.4334	0.0121	0.0019	-0.0001	0.0002
rs9375150	6	122887555	G	T	0.4468	-0.0184	0.0019	0.0000	0.0002
rs9379077	6	7167170	G	A	0.2009	0.0286	0.0023	-0.0002	0.0002
rs9451139	6	89777631	T	A	0.3197	-0.0139	0.0020	-0.0001	0.0002
rs9479025	6	151698748	T	A	0.5185	0.0142	0.0019	0.0001	0.0002
rs9480737	6	107442277	G	A	0.3211	-0.0217	0.0020	0.0003	0.0002
rs10238435	7	28724491	T	C	0.2397	-0.0236	0.0023	-0.0001	0.0002
rs111363146	7	44801682	C	T	0.1358	0.0222	0.0028	0.0000	0.0002
rs11772895	7	143081942	C	G	0.2768	0.0206	0.0021	0.0004	0.0002
rs11974139	7	149160886	C	T	0.2846	-0.0138	0.0021	0.0001	0.0002
rs13229868	7	6881302	A	G	0.1973	0.0156	0.0026	0.0000	0.0002
rs13238198	7	49837504	T	C	0.1076	-0.0216	0.0030	-0.0003	0.0003
rs146039611	7	92323422	A	G	0.0194	0.0714	0.0070	0.0000	0.0006
rs147341073	7	92381696	G	A	0.0278	-0.0747	0.0058	0.0001	0.0005
rs149007767	7	50370254	T	C	0.1621	-0.0722	0.0027	-0.0001	0.0002
rs17156536	7	28279488	A	C	0.1594	0.0247	0.0026	-0.0001	0.0002
rs17700436	7	17167825	T	C	0.0574	-0.0285	0.0040	0.0002	0.0004
rs178405	7	73513045	T	G	0.7840	-0.0149	0.0023	0.0002	0.0002
rs2279860	7	138721021	G	A	0.5122	-0.0115	0.0019	-0.0003	0.0002
rs2282986	7	92299545	C	T	0.0184	-0.0523	0.0070	-0.0006	0.0006
rs2885735	7	949115	G	A	0.8234	0.0143	0.0026	0.0001	0.0002
rs342294	7	106372622	C	T	0.4555	-0.0110	0.0019	0.0002	0.0002
rs3731332	7	92300568	T	C	0.0229	-0.1080	0.0064	0.0005	0.0006
rs41430449	7	50798525	G	C	0.0664	-0.0300	0.0038	0.0000	0.0003
rs4385425	7	50307334	G	A	0.3239	-0.0601	0.0020	-0.0001	0.0002
rs4534048	7	21944185	G	A	0.2920	0.0124	0.0021	-0.0002	0.0002
rs4730221	7	106809649	C	A	0.5451	0.0124	0.0019	0.0001	0.0002
rs61753095	7	2583328	T	C	0.2095	0.0155	0.0023	-0.0001	0.0002
rs62454420	7	27191804	G	A	0.0702	-0.0349	0.0037	0.0001	0.0003
rs62470670	7	114651694	G	A	0.0925	-0.0233	0.0032	0.0003	0.0003
rs62489376	7	129583934	C	T	0.1676	0.0177	0.0025	-0.0002	0.0002
rs6465661	7	97885213	T	C	0.4916	-0.0163	0.0019	-0.0003	0.0002
rs6585	7	134850725	A	G	0.4948	-0.0106	0.0019	0.0001	0.0002
rs6796	7	6502367	C	T	0.2743	0.0455	0.0021	0.0002	0.0002
rs6950469	7	137611873	C	G	0.4452	-0.0130	0.0019	0.0001	0.0002
rs73142138	7	65347962	G	A	0.3995	0.0128	0.0019	0.0001	0.0002
rs7785014	7	137881690	C	T	0.6708	-0.0253	0.0020	0.0001	0.0002
rs7792525	7	99972122	G	A	0.1859	0.0189	0.0024	0.0003	0.0002
rs7803075	7	130742066	G	A	0.7353	-0.0144	0.0021	0.0000	0.0002
rs7805504	7	73042585	C	T	0.2077	0.0140	0.0023	0.0002	0.0002
rs798563	7	2757867	C	A	0.2971	0.0176	0.0021	0.0000	0.0002

rs834601	7	47445812	C	T	0.4930	-0.0113	0.0019	-0.0001	0.0002
rs855673	7	149464433	C	T	0.1650	-0.0152	0.0026	-0.0001	0.0002
rs10099546	8	144638734	G	A	0.1433	-0.0203	0.0028	-0.0002	0.0002
rs10101101	8	130691767	A	G	0.1388	-0.0412	0.0028	-0.0003	0.0002
rs117182261	8	43508916	A	G	0.0264	-0.0536	0.0066	-0.0002	0.0005
rs12334935	8	126617990	A	G	0.4709	-0.0150	0.0019	0.0000	0.0002
rs12542907	8	68813188	G	C	0.3989	-0.0242	0.0019	0.0001	0.0002
rs13248936	8	30316162	G	A	0.4983	0.0124	0.0019	0.0000	0.0002
rs13267464	8	6906925	T	C	0.3994	-0.0205	0.0019	0.0001	0.0002
rs13271228	8	116597409	G	T	0.5671	-0.0306	0.0019	0.0001	0.0002
rs145718079	8	48641192	A	G	0.0102	-0.0802	0.0099	0.0001	0.0009
rs16939607	8	79013333	A	G	0.1460	-0.0252	0.0027	0.0001	0.0002
rs1863651	8	82066998	T	A	0.6994	-0.0167	0.0021	-0.0003	0.0002
rs1954735	8	108239325	C	T	0.1684	0.0208	0.0025	0.0002	0.0002
rs2466008	8	56937347	C	G	0.2183	0.0184	0.0023	0.0001	0.0002
rs2737229	8	116648565	C	A	0.3008	-0.0119	0.0020	-0.0003	0.0002
rs2978889	8	6697316	A	G	0.5039	-0.0191	0.0019	-0.0001	0.0002
rs34935896	8	39117823	T	G	0.0503	-0.0237	0.0043	-0.0001	0.0004
rs3808609	8	59465377	C	G	0.3180	0.0169	0.0020	0.0000	0.0002
rs4397371	8	130701417	T	C	0.7340	-0.0184	0.0021	-0.0001	0.0002
rs45577137	8	48651633	G	A	0.0439	-0.0646	0.0050	-0.0002	0.0004
rs4871844	8	22879734	C	T	0.3431	0.0155	0.0020	0.0000	0.0002
rs62501136	8	23089429	A	G	0.2214	0.0205	0.0023	-0.0001	0.0002
rs62502392	8	27190945	A	G	0.1431	-0.0179	0.0027	0.0000	0.0002
rs72673751	8	106578940	C	T	0.1900	-0.0166	0.0024	0.0003	0.0002
rs7462003	8	43510694	A	C	0.9581	0.0324	0.0048	-0.0009	0.0004
rs74915527	8	48806383	T	C	0.0975	0.0185	0.0032	0.0000	0.0003
rs75910605	8	74721125	C	T	0.0254	-0.0384	0.0060	0.0000	0.0005
rs7824937	8	61392724	G	A	0.3686	0.0177	0.0019	0.0001	0.0002
rs7826487	8	6880925	G	A	0.1158	-0.0410	0.0030	-0.0001	0.0003
rs7836456	8	11829175	G	T	0.5144	-0.0150	0.0020	0.0000	0.0002
rs7836786	8	121006826	A	G	0.8150	-0.0132	0.0024	-0.0001	0.0002
rs7843207	8	130570063	A	C	0.5056	-0.0692	0.0019	0.0001	0.0002
rs837227	8	130964654	T	C	0.7448	-0.0147	0.0022	0.0003	0.0002
rs1008158	9	113828811	G	A	0.3427	-0.0674	0.0020	0.0002	0.0002
rs10780209	9	91472127	A	G	0.4794	0.0454	0.0019	0.0000	0.0002
rs10814193	9	35018686	C	A	0.7156	0.0147	0.0021	-0.0001	0.0002
rs10817147	9	113864075	T	C	0.3765	-0.0589	0.0019	0.0001	0.0002
rs10987787	9	130729153	G	C	0.7680	-0.0197	0.0022	-0.0001	0.0002
rs11557154	9	34107505	T	C	0.1276	0.0320	0.0028	-0.0002	0.0002
rs12376511	9	22142756	C	T	0.1639	-0.0486	0.0025	0.0002	0.0002
rs13292863	9	113316712	G	C	0.1548	-0.0161	0.0026	0.0001	0.0002
rs13293713	9	114353573	A	G	0.0806	0.0525	0.0035	0.0004	0.0003
rs2236288	9	35749845	C	G	0.2288	0.0135	0.0022	0.0004	0.0002
rs2253843	9	130313865	G	A	0.2783	-0.0131	0.0021	0.0000	0.0002
rs2773810	9	135873066	G	A	0.3363	0.0132	0.0020	0.0003	0.0002
rs290243	9	93571337	A	G	0.2145	0.0143	0.0023	0.0000	0.0002
rs3731211	9	21986847	A	T	0.7198	0.0284	0.0021	-0.0002	0.0002
rs4838147	9	127034352	G	A	0.1540	0.0318	0.0026	-0.0002	0.0002
rs676996	9	136146077	G	T	0.3202	-0.0438	0.0020	0.0002	0.0002
rs77155602	9	114153208	T	C	0.1796	0.0292	0.0024	-0.0001	0.0002
rs7861055	9	38197033	G	A	0.4876	-0.0141	0.0019	-0.0002	0.0002
rs79446921	9	91652798	A	G	0.0538	-0.0229	0.0041	-0.0001	0.0004
rs9410425	9	91562311	A	G	0.3246	-0.0300	0.0020	0.0000	0.0002
rs1043009	10	3819714	T	C	0.3770	-0.0133	0.0019	0.0000	0.0002
rs10795595	10	5895923	T	A	0.4253	0.0196	0.0019	0.0001	0.0002
rs10828725	10	25218243	T	G	0.3678	-0.0473	0.0020	0.0003	0.0002
rs10997925	10	69829643	C	T	0.0645	-0.0235	0.0039	0.0002	0.0003

rs11000009	10	73569749	T	C	0.1160	0.0165	0.0029	0.0003	0.0003
rs111456533	10	126439381	A	G	0.1635	-0.0143	0.0025	-0.0002	0.0002
rs11189154	10	99108922	A	G	0.2557	0.0505	0.0021	0.0002	0.0002
rs11190141	10	101292390	T	C	0.3720	-0.0437	0.0019	-0.0003	0.0002
rs11191206	10	103832689	C	G	0.4532	0.0111	0.0019	0.0000	0.0002
rs1180658	10	61645945	A	C	0.6339	-0.0125	0.0019	0.0001	0.0002
rs1270799	10	21907423	G	T	0.3039	0.0129	0.0021	-0.0002	0.0002
rs17011726	10	50264204	G	C	0.2333	-0.0228	0.0022	-0.0003	0.0002
rs1749824	10	80923862	A	C	0.4325	-0.0119	0.0020	0.0001	0.0002
rs1781799	10	79697211	T	C	0.6127	0.0112	0.0019	0.0000	0.0002
rs224111	10	64552010	A	G	0.3902	-0.0209	0.0019	-0.0001	0.0002
rs2646421	10	8467155	C	G	0.3778	-0.0261	0.0019	0.0000	0.0002
rs475616	10	30496905	G	A	0.6563	0.0157	0.0020	0.0001	0.0002
rs7094871	10	114712154	G	C	0.5426	-0.0142	0.0019	0.0004	0.0002
rs7097656	10	82250831	C	T	0.7948	0.0202	0.0023	0.0001	0.0002
rs72790862	10	44880260	C	T	0.3071	-0.0203	0.0020	-0.0001	0.0002
rs72828247	10	111750960	T	G	0.1348	-0.0191	0.0028	0.0004	0.0002
rs74364334	10	89878334	A	G	0.0498	0.0348	0.0044	-0.0007	0.0004
rs7475853	10	104673097	A	G	0.0798	-0.0280	0.0035	0.0003	0.0003
rs7918233	10	3919071	A	G	0.4888	0.0120	0.0019	-0.0001	0.0002
rs7919533	10	73525525	C	T	0.4879	0.0204	0.0019	-0.0001	0.0002
rs9787670	10	96010237	A	G	0.2980	-0.0135	0.0021	0.0001	0.0002
rs10796828	11	69490346	G	T	0.6350	-0.0180	0.0020	0.0002	0.0002
rs10831507	11	96060068	C	A	0.3897	0.0108	0.0019	0.0001	0.0002
rs10892342	11	119135814	C	T	0.2761	-0.0181	0.0021	0.0000	0.0002
rs10892891	11	122608411	A	G	0.3834	-0.0142	0.0019	-0.0001	0.0002
rs11235689	11	72949747	T	C	0.4139	-0.0161	0.0020	0.0003	0.0002
rs11246065	11	324170	G	A	0.3391	-0.0221	0.0021	0.0001	0.0002
rs11602323	11	122519281	G	T	0.1231	0.0652	0.0029	0.0001	0.0002
rs12575164	11	128349430	C	T	0.2196	-0.0276	0.0023	-0.0003	0.0002
rs1885525	11	33904180	A	G	0.5060	0.0129	0.0019	0.0003	0.0002
rs2262541	11	54842280	C	T	0.9644	0.0295	0.0051	0.0003	0.0004
rs2294081	11	193863	C	T	0.4808	0.0146	0.0020	0.0000	0.0002
rs2606724	11	113957880	A	G	0.4510	-0.0138	0.0019	0.0003	0.0002
rs2957873	11	47249294	A	G	0.8085	-0.0211	0.0024	-0.0004	0.0002
rs531831	11	114071814	T	C	0.2507	-0.0128	0.0022	0.0001	0.0002
rs573790	11	59855385	C	T	0.6137	-0.0161	0.0019	0.0001	0.0002
rs597136	11	128545217	T	C	0.1902	0.0260	0.0024	-0.0003	0.0002
rs622614	11	65663239	C	T	0.8000	0.0193	0.0024	0.0003	0.0002
rs626816	11	128619775	A	G	0.4933	-0.0110	0.0019	0.0001	0.0002
rs6591362	11	68746062	C	A	0.3186	-0.0113	0.0020	0.0002	0.0002
rs6591578	11	60158649	A	G	0.6336	0.0219	0.0019	-0.0001	0.0002
rs662333	11	121578945	A	G	0.7795	-0.0138	0.0023	-0.0001	0.0002
rs7120300	11	8823493	T	C	0.7349	-0.0172	0.0021	0.0003	0.0002
rs72966841	11	88070110	T	A	0.0936	-0.0267	0.0032	0.0000	0.0003
rs907612	11	1874221	T	C	0.3804	-0.0331	0.0020	0.0000	0.0002
rs964184	11	116648917	C	G	0.8659	0.0198	0.0027	0.0002	0.0002
rs9734613	11	46249863	G	A	0.9638	0.0288	0.0051	0.0005	0.0004
rs9783374	11	2340619	C	T	0.8490	0.0209	0.0027	0.0002	0.0002
rs10845585	12	12749625	G	A	0.5503	-0.0133	0.0019	0.0000	0.0002
rs10849020	12	4332009	G	C	0.2102	-0.0241	0.0023	-0.0001	0.0002
rs10849448	12	6493351	G	A	0.7533	-0.0501	0.0022	-0.0001	0.0002
rs10860738	12	101867997	A	G	0.6696	0.0128	0.0020	0.0000	0.0002
rs11104881	12	88843474	C	T	0.7021	-0.0156	0.0021	-0.0001	0.0002
rs11111707	12	104057384	G	C	0.0881	-0.0203	0.0033	0.0003	0.0003
rs11170652	12	54099076	A	G	0.1947	-0.0181	0.0024	0.0005	0.0002
rs11614523	12	1075536	C	T	0.4363	0.0137	0.0020	0.0000	0.0002
rs12306790	12	89864047	T	C	0.7146	0.0191	0.0021	0.0002	0.0002

rs12321936	12	108726546	A	G	0.0789	0.0216	0.0035	0.0000	0.0003
rs1800973	12	69744014	A	C	0.0619	0.1134	0.0039	0.0010	0.0003
rs2651369	12	32552769	G	C	0.3256	0.0141	0.0020	0.0002	0.0002
rs2734442	12	10600368	A	G	0.8755	-0.0420	0.0028	0.0002	0.0002
rs3184504	12	111884608	C	T	0.5174	-0.0471	0.0019	0.0002	0.0002
rs34038797	12	740009	G	C	0.4812	-0.0194	0.0019	0.0001	0.0002
rs35979828	12	54685880	T	C	0.0708	-0.0461	0.0037	0.0000	0.0003
rs3860253	12	80280844	C	G	0.9262	-0.0211	0.0036	-0.0002	0.0003
rs4082413	12	30783184	G	C	0.5122	0.0154	0.0019	0.0002	0.0002
rs4763944	12	13357972	A	G	0.2044	0.0131	0.0023	0.0002	0.0002
rs61736007	12	57618619	A	G	0.2685	-0.0140	0.0021	0.0003	0.0002
rs61931988	12	124863847	C	T	0.5582	0.0148	0.0020	0.0001	0.0002
rs6538697	12	96403610	C	T	0.0776	0.0194	0.0035	0.0005	0.0003
rs706819	12	52315923	C	T	0.7383	0.0184	0.0022	0.0001	0.0002
rs73069021	12	21399955	A	G	0.1605	-0.0157	0.0025	-0.0002	0.0002
rs7308348	12	122225420	C	T	0.1821	0.0154	0.0025	0.0001	0.0002
rs7314538	12	110050110	G	A	0.4428	0.0155	0.0019	0.0000	0.0002
rs73201961	12	116830960	C	A	0.0892	0.0272	0.0033	0.0000	0.0003
rs795473	12	118607737	G	A	0.4283	-0.0107	0.0019	0.0001	0.0002
rs1146932	13	78403687	C	T	0.7876	-0.0142	0.0023	-0.0001	0.0002
rs12874404	13	108993494	G	A	0.0555	-0.0645	0.0041	0.0002	0.0004
rs138028125	13	28712689	G	C	0.0348	0.1354	0.0056	-0.0001	0.0005
rs17086239	13	28611186	C	G	0.0412	-0.0686	0.0048	0.0008	0.0004
rs188175496	13	28595940	T	C	0.0244	-0.0929	0.0067	-0.0001	0.0006
rs1892548	13	41002641	C	T	0.6548	-0.0628	0.0020	0.0001	0.0002
rs2183246	13	114901188	T	C	0.6828	-0.0138	0.0021	-0.0001	0.0002
rs2504209	13	28546766	G	A	0.8111	0.0184	0.0024	0.0002	0.0002
rs58814158	13	110820255	G	T	0.1574	-0.0235	0.0026	-0.0003	0.0002
rs60699901	13	92009193	C	T	0.1181	0.0215	0.0029	0.0000	0.0003
rs61971980	13	108899416	A	G	0.1726	-0.0182	0.0025	0.0001	0.0002
rs73217470	13	72513420	G	A	0.0467	0.0648	0.0045	0.0002	0.0004
rs76428106	13	28604007	C	T	0.0134	0.5355	0.0086	-0.0006	0.0007
rs77733744	13	52344004	A	G	0.1719	-0.0140	0.0025	-0.0002	0.0002
rs7996207	13	50122681	A	G	0.6935	0.0205	0.0020	0.0002	0.0002
rs9532580	13	41244260	C	T	0.2637	-0.0281	0.0021	-0.0002	0.0002
rs9555596	13	110011839	C	T	0.3833	0.0229	0.0019	-0.0001	0.0002
rs9568031	13	48897520	T	C	0.7059	-0.0121	0.0021	0.0002	0.0002
rs9573092	13	73627275	G	A	0.3492	-0.0109	0.0020	0.0006	0.0002
rs1052484	14	25281444	C	G	0.2158	0.0139	0.0023	-0.0002	0.0002
rs12147629	14	75307588	G	A	0.3116	0.0166	0.0020	-0.0001	0.0002
rs2038700	14	25461989	C	T	0.3942	0.0400	0.0019	0.0000	0.0002
rs2063996	14	75820645	C	T	0.6259	-0.0113	0.0019	-0.0002	0.0002
rs2239630	14	23589349	G	A	0.5560	0.0488	0.0019	0.0000	0.0002
rs4905043	14	93550009	A	G	0.3995	0.0187	0.0020	0.0000	0.0002
rs4983387	14	105268228	A	G	0.8991	0.0194	0.0032	-0.0002	0.0003
rs61985545	14	60040825	A	G	0.4952	-0.0115	0.0019	-0.0001	0.0002
rs7148739	14	35319833	T	C	0.0969	0.0304	0.0032	-0.0002	0.0003
rs754388	14	93115410	C	G	0.8109	0.0303	0.0024	0.0001	0.0002
rs77406312	14	36050217	C	T	0.0872	-0.0237	0.0034	0.0001	0.0003
rs8016326	14	103846716	A	G	0.7338	0.0611	0.0021	0.0000	0.0002
rs1002774	15	42261781	A	G	0.1117	-0.0540	0.0030	-0.0002	0.0003
rs1081230	15	80095646	G	A	0.5772	-0.0197	0.0019	-0.0001	0.0002
rs117677615	15	43105549	A	G	0.0846	0.0198	0.0034	0.0005	0.0003
rs11857609	15	66095270	C	T	0.7598	0.0233	0.0022	0.0003	0.0002
rs12595562	15	50974453	C	T	0.2141	-0.0178	0.0023	0.0001	0.0002
rs2061822	15	86122779	C	T	0.6741	-0.0136	0.0020	-0.0002	0.0002
rs2733095	15	80250863	G	A	0.7609	0.0694	0.0022	0.0001	0.0002
rs4577036	15	80298484	T	G	0.0755	0.0241	0.0036	0.0003	0.0003

rs62011334	15	63837491	G	A	0.3552	0.0119	0.0020	-0.0001	0.0002
rs62018159	15	40397421	A	G	0.2091	0.0150	0.0023	0.0000	0.0002
rs6493575	15	53013475	T	C	0.3957	-0.0122	0.0019	0.0001	0.0002
rs6938	15	75136261	G	C	0.6934	-0.0198	0.0021	0.0000	0.0002
rs7180079	15	64629873	G	A	0.8769	0.0594	0.0029	0.0004	0.0003
rs73467599	15	99088453	C	T	0.1995	0.0185	0.0024	-0.0001	0.0002
rs11508026	16	56999328	T	C	0.4313	-0.0119	0.0019	-0.0002	0.0002
rs11644125	16	57058974	T	C	0.5979	-0.0194	0.0019	0.0002	0.0002
rs12325238	16	10981518	A	C	0.2654	0.0174	0.0021	0.0002	0.0002
rs12935169	16	67010113	T	C	0.0165	-0.0616	0.0077	-0.0006	0.0007
rs1967309	16	4065583	G	A	0.6031	-0.0221	0.0019	-0.0003	0.0002
rs205422	16	28076858	C	T	0.6974	0.0128	0.0021	0.0002	0.0002
rs2885363	16	86009261	C	G	0.0792	0.1166	0.0035	-0.0003	0.0003
rs36026517	16	74597758	C	G	0.1987	-0.0167	0.0023	0.0002	0.0002
rs366078	16	85964563	C	T	0.1576	0.1070	0.0026	-0.0003	0.0002
rs430634	16	84556707	T	C	0.2086	0.0191	0.0023	-0.0001	0.0002
rs68131969	16	85883140	A	G	0.0210	0.0563	0.0067	-0.0013	0.0006
rs7185007	16	30927509	T	C	0.2402	-0.0167	0.0022	0.0002	0.0002
rs7191820	16	79363814	G	A	0.6738	0.0114	0.0020	-0.0001	0.0002
rs7196129	16	30471109	C	T	0.5303	0.0250	0.0019	-0.0001	0.0002
rs7204799	16	11004549	G	C	0.9240	0.0209	0.0036	0.0001	0.0003
rs76209847	16	85967249	T	C	0.0371	-0.0746	0.0051	-0.0001	0.0004
rs79252394	16	86021968	T	G	0.0302	-0.0427	0.0056	0.0008	0.0005
rs8049116	16	4150530	T	C	0.1183	0.0230	0.0029	-0.0001	0.0003
rs9937847	16	85917551	C	T	0.0708	-0.1123	0.0037	0.0005	0.0003
rs11655888	17	79389411	T	C	0.1801	-0.0141	0.0025	0.0003	0.0002
rs11656162	17	56404625	C	G	0.0337	0.0351	0.0054	-0.0001	0.0005
rs118083884	17	16522922	A	G	0.0167	-0.0965	0.0085	-0.0005	0.0007
rs12936934	17	7500765	T	C	0.1975	0.0177	0.0024	-0.0002	0.0002
rs12941356	17	17716531	G	A	0.5885	-0.0174	0.0020	0.0001	0.0002
rs16960644	17	19953617	C	T	0.1889	0.0134	0.0024	0.0000	0.0002
rs16978176	17	72761242	C	T	0.4653	-0.0254	0.0019	0.0001	0.0002
rs190591650	17	80738558	G	T	0.0268	-0.0371	0.0063	0.0001	0.0005
rs2084312	17	72695211	T	C	0.8003	0.0377	0.0024	0.0001	0.0002
rs2302774	17	38183090	T	G	0.3825	-0.0242	0.0019	-0.0001	0.0002
rs236513	17	68169458	C	T	0.1180	-0.0202	0.0029	0.0007	0.0003
rs2521879	17	62742678	G	A	0.8040	-0.0130	0.0024	0.0002	0.0002
rs2665405	17	57875292	A	G	0.5483	0.0384	0.0019	0.0000	0.0002
rs2729450	17	28088459	C	T	0.5242	-0.0306	0.0019	-0.0003	0.0002
rs2740354	17	632905	C	T	0.6386	0.0113	0.0020	0.0001	0.0002
rs34097845	17	56413635	T	C	0.0560	-0.1006	0.0042	-0.0001	0.0003
rs3809857	17	44848314	T	G	0.3260	-0.0156	0.0020	0.0000	0.0002
rs4789078	17	72554661	G	A	0.3637	0.0134	0.0020	0.0003	0.0002
rs4792849	17	43395178	G	A	0.7418	-0.0216	0.0021	-0.0001	0.0002
rs56013073	17	38357705	C	T	0.1461	0.0239	0.0027	0.0000	0.0002
rs62057782	17	25831128	A	G	0.1195	-0.0193	0.0029	-0.0006	0.0003
rs72835478	17	47369481	T	C	0.0261	0.0366	0.0059	0.0002	0.0005
rs745570	17	77781725	G	A	0.5136	0.0141	0.0019	0.0000	0.0002
rs7501986	17	8144554	T	C	0.4483	0.0108	0.0019	-0.0002	0.0002
rs77569276	17	32534568	G	C	0.1816	0.0190	0.0024	0.0001	0.0002
rs8066044	17	1367352	G	A	0.7320	0.0146	0.0022	0.0000	0.0002
rs8081327	17	40274200	A	G	0.2265	0.0214	0.0022	-0.0001	0.0002
rs865483	17	35851177	C	A	0.6429	-0.0258	0.0020	0.0000	0.0002
rs869717	17	27423115	G	A	0.1740	0.0188	0.0025	0.0001	0.0002
rs9915112	17	2007826	G	A	0.2031	-0.0266	0.0023	0.0003	0.0002
rs10460159	18	60996360	C	T	0.4290	-0.0158	0.0019	-0.0001	0.0002
rs12327253	18	20719164	G	A	0.2936	0.0178	0.0021	-0.0002	0.0002
rs141390096	18	60911544	C	G	0.0371	-0.0332	0.0050	0.0002	0.0004

rs17758695	18	60920854	T	C	0.0288	-0.1093	0.0058	0.0000	0.0005
rs1790576	18	67518843	C	A	0.5340	-0.0126	0.0019	-0.0002	0.0002
rs2007483	18	77476131	A	T	0.3823	-0.0237	0.0019	0.0001	0.0002
rs2013605	18	19726151	T	G	0.1024	0.0182	0.0031	-0.0002	0.0003
rs238136	18	3448755	G	A	0.2500	-0.0150	0.0022	0.0000	0.0002
rs2846573	18	841779	T	C	0.3518	-0.0108	0.0020	0.0000	0.0002
rs3177609	18	74071078	C	T	0.0776	0.0312	0.0035	-0.0007	0.0003
rs613872	18	53210302	T	G	0.8274	0.0173	0.0025	0.0000	0.0002
rs62100531	18	33112001	C	G	0.1442	-0.0169	0.0027	0.0005	0.0002
rs718515	18	43856297	A	G	0.5522	-0.0199	0.0019	0.0001	0.0002
rs745822	18	48142822	G	T	0.3757	0.0164	0.0019	-0.0001	0.0002
rs75763843	18	42062380	C	A	0.1292	0.0191	0.0028	0.0000	0.0002
rs954954	18	60902328	C	A	0.1052	-0.0290	0.0031	-0.0001	0.0003
rs9963693	18	45599257	C	T	0.2606	0.0133	0.0021	-0.0001	0.0002
rs1003393	19	13212214	T	C	0.0166	0.0778	0.0079	-0.0001	0.0007
rs11086102	19	18398628	C	G	0.6329	0.0314	0.0020	0.0000	0.0002
rs11669910	19	45741333	T	A	0.2584	-0.0665	0.0021	0.0000	0.0002
rs117758012	19	12957650	T	C	0.0593	-0.0353	0.0041	0.0000	0.0003
rs12459419	19	51728477	T	C	0.3220	-0.0193	0.0020	0.0003	0.0002
rs12461422	19	3167542	T	C	0.2830	-0.0160	0.0022	0.0003	0.0002
rs141801008	19	10374952	T	C	0.0096	-0.0886	0.0102	0.0000	0.0009
rs146989801	19	45630360	G	C	0.0167	0.0495	0.0081	-0.0008	0.0007
rs28540338	19	53400168	C	T	0.5477	0.0120	0.0019	0.0001	0.0002
rs2927455	19	45267258	C	T	0.0626	0.0252	0.0041	0.0002	0.0003
rs338585	19	41711815	A	G	0.4407	-0.0113	0.0019	-0.0003	0.0002
rs34057576	19	52162832	T	C	0.3883	-0.0159	0.0019	0.0002	0.0002
rs3803904	19	55699646	T	C	0.1341	0.0172	0.0029	-0.0004	0.0002
rs3859570	19	18510925	C	T	0.4248	-0.0278	0.0020	0.0001	0.0002
rs413141	19	6675989	G	A	0.8625	0.0430	0.0028	-0.0001	0.0002
rs4632248	19	54324995	T	G	0.2111	-0.0652	0.0023	0.0001	0.0002
rs4807440	19	1026477	T	G	0.6375	0.0251	0.0021	0.0003	0.0002
rs55697131	19	36751636	G	A	0.2090	-0.0172	0.0023	-0.0001	0.0002
rs56192531	19	3258148	A	G	0.1241	-0.0244	0.0029	0.0001	0.0002
rs56344893	19	44282529	A	C	0.3730	-0.0443	0.0020	0.0001	0.0002
rs571497	19	7827830	A	G	0.1546	-0.0282	0.0026	-0.0004	0.0002
rs67178614	19	1658966	G	C	0.2395	-0.0130	0.0023	-0.0004	0.0002
rs7249692	19	19670688	C	T	0.6707	-0.0172	0.0020	0.0001	0.0002
rs736926	19	801381	T	C	0.1394	-0.0184	0.0028	-0.0003	0.0002
rs74259566	19	42735177	G	C	0.1005	0.0287	0.0031	0.0000	0.0003
rs78371511	19	38924996	C	T	0.0309	0.0342	0.0055	0.0002	0.0005
rs78512588	19	13094218	A	C	0.0294	-0.0341	0.0058	0.0006	0.0005
rs79407714	19	33745290	A	G	0.0300	-0.0644	0.0058	-0.0007	0.0005
rs8104986	19	33761313	G	A	0.8334	0.0171	0.0026	0.0002	0.0002
rs12480462	20	31065178	T	C	0.3160	-0.0310	0.0020	-0.0001	0.0002
rs12480732	20	31191015	T	C	0.2578	0.0543	0.0022	0.0001	0.0002
rs17196752	20	48887268	T	C	0.1899	-0.0725	0.0024	0.0004	0.0002
rs2143607	20	42838712	G	C	0.4335	0.0223	0.0019	0.0000	0.0002
rs2295481	20	61574727	T	C	0.2074	-0.0153	0.0023	-0.0002	0.0002
rs235529	20	3597171	G	A	0.7236	-0.0146	0.0021	0.0001	0.0002
rs34600126	20	1597433	T	G	0.2177	0.0224	0.0023	0.0000	0.0002
rs3790163	20	10647951	G	A	0.7927	-0.0147	0.0024	0.0004	0.0002
rs4812447	20	39272620	G	A	0.4404	0.0221	0.0019	0.0000	0.0002
rs4813619	20	2815715	T	G	0.5108	0.0165	0.0019	0.0002	0.0002
rs6020624	20	49214156	G	A	0.7045	-0.0187	0.0021	-0.0001	0.0002
rs6044081	20	16513342	G	A	0.7581	0.0123	0.0022	0.0000	0.0002
rs6045733	20	1950858	A	G	0.3504	-0.0114	0.0020	0.0000	0.0002
rs6055955	20	8604181	T	C	0.5085	-0.0290	0.0019	0.0000	0.0002
rs6120950	20	30225070	T	C	0.2028	-0.0194	0.0023	0.0000	0.0002

rs6122884	20	48789388	G	A	0.3304	0.0216	0.0020	-0.0001	0.0002
rs62191845	20	11311923	T	C	0.1135	0.0192	0.0030	0.0001	0.0003
rs6512627	20	48967591	A	G	0.1936	-0.0474	0.0024	0.0001	0.0002
rs927377	20	38639838	T	C	0.6174	0.0106	0.0019	0.0002	0.0002
rs1997577	21	16371102	T	A	0.1550	-0.0296	0.0026	-0.0001	0.0002
rs2836220	21	39600499	C	A	0.3385	-0.0114	0.0020	-0.0001	0.0002
rs35068491	21	36238307	C	T	0.2092	0.0146	0.0024	0.0004	0.0002
rs57221391	21	16583936	G	T	0.0714	-0.0223	0.0037	-0.0002	0.0003
rs73203055	21	36407530	G	C	0.0720	-0.0261	0.0037	0.0003	0.0003
rs9981640	21	43431830	C	T	0.2794	-0.0127	0.0021	0.0000	0.0002
rs138632530	22	17676901	T	C	0.1094	0.0248	0.0032	0.0007	0.0003
rs139271	22	39487595	C	T	0.4422	-0.0153	0.0019	0.0000	0.0002
rs140763648	22	50767351	G	A	0.4124	-0.0117	0.0020	-0.0001	0.0002
rs181406	22	18233525	C	G	0.6593	0.0120	0.0021	-0.0001	0.0002
rs192498589	22	17516047	G	A	0.0170	0.0696	0.0081	0.0003	0.0007
rs2015580	22	32326021	T	C	0.3119	-0.0174	0.0021	0.0000	0.0002
rs2019180	22	17712142	A	G	0.0932	-0.0367	0.0034	-0.0001	0.0003
rs2092274	22	42123737	G	A	0.8093	0.0134	0.0024	0.0001	0.0002
rs34505104	22	24624609	G	A	0.3053	-0.0331	0.0021	0.0000	0.0002
rs41409548	22	17579495	A	G	0.0338	-0.0965	0.0057	-0.0006	0.0005
rs45462093	22	28187396	G	A	0.2294	0.0130	0.0023	0.0000	0.0002
rs47341	22	43560763	T	C	0.3960	0.0311	0.0020	0.0000	0.0002
rs55740286	22	40798227	G	T	0.0531	-0.0238	0.0042	0.0004	0.0004
rs5748937	22	17675324	T	C	0.0549	-0.0693	0.0046	-0.0003	0.0004
rs9616327	22	50010891	A	G	0.6212	0.0185	0.0019	0.0003	0.0002
rs9625746	22	29637658	C	G	0.4127	-0.0213	0.0019	0.0001	0.0002
