# Supplementary material for: Blood cell traits and risk of glaucoma: A two-sample mendelian randomization study
Source: Front Genet. 2023 Apr 12;14:1142773. doi: 10.3389/fgene.2023.1142773 (PMC10130872; doi:10.3389/fgene.2023.1142773)
Supplement: Supplementary file 1 [file DataSheet1.ZIP › eTable 3. Lymphocyte cell count exposure SNPs and their association with glaucoma.pdf]

eTable 3. Lymphocyte cell count exposure SNPs and their association with glaucoma.
Chr = chromosome; POS = position ; EA = effect allele; NEA = non-effect allele; EAF = effect allele frequency; SE = standard error.

SNP	Chr	POS	EA	NEA	EAF	Lymphocyte Cell Count		Glaucoma	
						Beta	SE	Beta	SE
rs10494783	1	198663661	A	G	0.0518	-0.0541	0.0043	-0.0003	0.0004
rs10875371	1	101762529	T	C	0.4178	-0.0208	0.0019	0.0002	0.0002
rs11247712	1	28229681	T	C	0.3689	-0.0199	0.0020	0.0000	0.0002
rs113466616	1	26726127	A	G	0.3331	0.0121	0.0020	-0.0001	0.0002
rs116505298	1	92841991	T	C	0.0356	0.0354	0.0051	-0.0006	0.0004
rs11800059	1	150651175	T	C	0.5100	-0.0256	0.0019	0.0002	0.0002
rs12021809	1	225941782	A	G	0.1098	-0.0263	0.0030	0.0005	0.0003
rs12038019	1	161685800	C	G	0.2680	-0.0165	0.0021	-0.0003	0.0002
rs12045893	1	158530416	T	C	0.2481	0.0268	0.0022	0.0002	0.0002
rs12059564	1	159000779	T	C	0.1503	0.0163	0.0026	0.0004	0.0002
rs12070594	1	51136876	A	C	0.0899	0.0214	0.0033	-0.0003	0.0003
rs12088882	1	101224620	T	C	0.4085	0.0340	0.0019	0.0002	0.0002
rs12124035	1	207080628	A	T	0.4559	-0.0117	0.0019	-0.0002	0.0002
rs12127284	1	65431599	T	C	0.7101	-0.0168	0.0021	0.0002	0.0002
rs12133917	1	101013188	A	C	0.4357	0.0156	0.0019	-0.0001	0.0002
rs12142033	1	39542129	A	G	0.3328	-0.0130	0.0020	-0.0001	0.0002
rs12745411	1	214405704	T	G	0.6001	0.0166	0.0019	0.0000	0.0002
rs1320562	1	90174762	C	G	0.7585	0.0127	0.0022	-0.0001	0.0002
rs144520705	1	101801341	A	C	0.0076	0.0667	0.0115	0.0007	0.0010
rs1493694	1	120526982	T	C	0.1079	0.0200	0.0030	0.0002	0.0003
rs17534202	1	203281175	C	G	0.5319	-0.0113	0.0019	0.0001	0.0002
rs1933295	1	62107021	G	A	0.7769	-0.0138	0.0023	0.0002	0.0002
rs1967115	1	212375359	A	G	0.6294	0.0168	0.0020	0.0001	0.0002
rs197433	1	112268666	C	T	0.8341	-0.0144	0.0026	0.0002	0.0002
rs214342	1	19681393	T	C	0.2290	0.0131	0.0023	0.0000	0.0002
rs2228445	1	203667409	C	T	0.9003	-0.0365	0.0032	-0.0001	0.0003
rs2275902	1	79357360	C	G	0.2976	-0.0161	0.0021	-0.0001	0.0002
rs2282718	1	25241056	A	G	0.3721	-0.0176	0.0020	-0.0004	0.0002
rs2476601	1	114377568	G	A	0.8997	0.0544	0.0031	0.0001	0.0003
rs2489279	1	182144119	G	C	0.3337	0.0176	0.0020	-0.0001	0.0002
rs2755253	1	67470843	T	C	0.7090	-0.0309	0.0021	-0.0002	0.0002
rs284278	1	10790536	G	A	0.6557	0.0129	0.0020	0.0000	0.0002
rs343757	1	111348951	G	A	0.2466	0.0149	0.0022	-0.0002	0.0002
rs4319365	1	56907720	A	G	0.5449	-0.0193	0.0019	0.0001	0.0002
rs4619033	1	204269242	G	A	0.2679	-0.0190	0.0021	-0.0002	0.0002
rs4660128	1	236087992	C	T	0.8970	-0.0248	0.0031	0.0003	0.0003
rs4925547	1	247612435	T	A	0.4024	0.0120	0.0019	0.0000	0.0002
rs494091	1	160791892	C	T	0.4256	0.0271	0.0019	-0.0001	0.0002
rs533483	1	234765256	A	G	0.2441	-0.0143	0.0022	0.0003	0.0002
rs59508186	1	66107881	A	C	0.3879	-0.0174	0.0019	0.0000	0.0002
rs6025	1	169519049	C	T	0.9767	-0.0369	0.0063	-0.0001	0.0005
rs60633358	1	91500508	C	T	0.1318	-0.0217	0.0028	-0.0001	0.0002
rs61778219	1	24235932	T	C	0.1409	0.0233	0.0027	-0.0002	0.0002
rs6692729	1	227018919	T	C	0.4738	-0.0117	0.0021	0.0001	0.0002
rs71519213	1	161572330	G	C	0.5650	0.0132	0.0020	0.0002	0.0002
rs716723	1	156984942	T	A	0.1343	-0.0170	0.0028	0.0002	0.0002
rs72634819	1	1596500	T	C	0.2636	-0.0143	0.0023	0.0003	0.0002
rs72740550	1	197342380	A	G	0.2221	-0.0187	0.0023	0.0003	0.0002
rs74227709	1	247722588	A	G	0.0715	-0.0289	0.0037	0.0000	0.0003
rs7527389	1	8556545	G	A	0.6306	-0.0193	0.0020	0.0000	0.0002
rs7529794	1	40403370	T	G	0.2624	0.0161	0.0022	0.0000	0.0002
rs753612	1	19978895	G	A	0.6119	0.0141	0.0019	0.0001	0.0002
rs7554936	1	151122489	T	C	0.6578	0.0110	0.0020	-0.0001	0.0002

rs75865061	1	221047603	A	G	0.1205	-0.0193	0.0029	0.0000	0.0003
rs77506398	1	45822980	C	T	0.0906	0.0200	0.0033	0.0003	0.0003
rs77954449	1	10242292	G	A	0.0283	-0.0368	0.0057	0.0003	0.0005
rs9429767	1	110496087	A	G	0.1974	0.0176	0.0024	-0.0001	0.0002
rs9662343	1	23574051	C	G	0.7372	-0.0150	0.0022	-0.0002	0.0002
rs10174056	2	162972370	A	T	0.3844	0.0140	0.0019	0.0000	0.0002
rs10179385	2	153288972	C	T	0.1568	-0.0191	0.0026	-0.0002	0.0002
rs10179705	2	188315234	A	G	0.2944	-0.0213	0.0021	-0.0001	0.0002
rs10203838	2	213935900	C	T	0.4173	0.0111	0.0019	0.0000	0.0002
rs10206961	2	85814984	T	C	0.4182	-0.0106	0.0019	-0.0002	0.0002
rs1047891	2	211540507	A	C	0.3151	-0.0153	0.0020	0.0002	0.0002
rs10865331	2	62551472	G	A	0.6250	0.0132	0.0020	-0.0002	0.0002
rs10932018	2	204627407	A	G	0.7297	-0.0146	0.0021	-0.0001	0.0002
rs112276394	2	112217228	T	C	0.0351	-0.0548	0.0055	-0.0002	0.0004
rs113542380	2	43464818	A	G	0.0749	-0.0675	0.0036	-0.0001	0.0003
rs11676298	2	227291731	G	C	0.1907	0.0270	0.0024	0.0002	0.0002
rs11684463	2	182077535	T	A	0.3275	-0.0473	0.0020	0.0001	0.0002
rs11688289	2	40698811	C	T	0.1893	-0.0148	0.0024	-0.0001	0.0002
rs1177269	2	61287445	T	C	0.5650	-0.0137	0.0020	0.0002	0.0002
rs11897252	2	216250263	T	C	0.2335	-0.0139	0.0022	0.0000	0.0002
rs1260326	2	27730940	C	T	0.6021	-0.0240	0.0019	0.0000	0.0002
rs12623165	2	137039910	T	C	0.8774	-0.0216	0.0029	-0.0001	0.0003
rs12692735	2	165504565	T	G	0.3611	-0.0125	0.0020	0.0000	0.0002
rs13018466	2	11317226	C	T	0.5614	0.0130	0.0019	0.0004	0.0002
rs13020851	2	182434184	G	A	0.3729	-0.0119	0.0020	0.0000	0.0002
rs143025457	2	68577926	A	T	0.0361	-0.0350	0.0054	-0.0005	0.0005
rs144762127	2	112294894	T	C	0.0278	-0.0493	0.0062	0.0001	0.0005
rs1448318	2	223982434	A	G	0.6149	0.0178	0.0019	0.0001	0.0002
rs148601577	2	143847602	G	A	0.0196	-0.0488	0.0070	-0.0009	0.0006
rs1533298	2	111616304	T	C	0.7153	0.0310	0.0021	-0.0001	0.0002
rs2113812	2	12951779	G	A	0.4910	0.0165	0.0019	-0.0001	0.0002
rs2304518	2	128645618	A	G	0.1901	0.0139	0.0024	0.0003	0.0002
rs2345626	2	46067440	C	T	0.7621	0.0136	0.0022	0.0001	0.0002
rs2353701	2	170570687	C	T	0.4396	0.0115	0.0019	0.0001	0.0002
rs2542573	2	54046646	C	T	0.3310	-0.0148	0.0020	0.0001	0.0002
rs34030812	2	144248905	C	T	0.3674	-0.0182	0.0020	-0.0003	0.0002
rs34562738	2	102376755	A	G	0.7719	0.0128	0.0022	0.0001	0.0002
rs413028	2	225904282	G	C	0.5143	0.0151	0.0019	0.0000	0.0002
rs4407213	2	25541636	C	G	0.0316	0.0425	0.0055	-0.0002	0.0005
rs4671238	2	55324905	T	C	0.5603	-0.0136	0.0019	-0.0002	0.0002
rs4849169	2	113953657	C	A	0.4822	0.0209	0.0019	0.0003	0.0002
rs4907236	2	97187605	T	C	0.2832	0.0115	0.0021	-0.0001	0.0002
rs4972950	2	231221252	A	C	0.1076	0.0216	0.0030	0.0002	0.0003
rs531864	2	169703660	T	C	0.3500	-0.0129	0.0020	0.0001	0.0002
rs55982282	2	24188128	A	G	0.1353	0.0616	0.0028	0.0002	0.0002
rs56657871	2	231521475	A	G	0.2144	0.0153	0.0024	-0.0004	0.0002
rs59153528	2	16058094	G	C	0.2116	-0.0141	0.0024	0.0001	0.0002
rs62160910	2	136919140	C	G	0.0578	-0.0243	0.0041	-0.0001	0.0004
rs62180255	2	182188444	A	T	0.4410	0.0342	0.0019	0.0003	0.0002
rs6546881	2	74187420	T	C	0.6572	0.0127	0.0020	0.0003	0.0002
rs6721663	2	7615061	A	G	0.1587	-0.0261	0.0026	-0.0002	0.0002
rs6742799	2	161317460	C	A	0.1998	-0.0392	0.0024	-0.0005	0.0002
rs6743068	2	202153920	G	A	0.7297	0.0314	0.0021	0.0001	0.0002
rs6755786	2	103048103	T	C	0.7734	0.0266	0.0023	0.0002	0.0002
rs6755895	2	232579795	C	T	0.2310	-0.0234	0.0023	0.0002	0.0002
rs71413380	2	112454561	T	C	0.4137	-0.0118	0.0019	-0.0001	0.0002
rs71420836	2	129060871	T	C	0.0971	0.0302	0.0033	0.0000	0.0003
rs73961715	2	143803847	C	T	0.1277	-0.0490	0.0028	-0.0005	0.0002

rs7572278	2	8563029	A	T	0.2068	0.0204	0.0024	0.0001	0.0002
rs7574865	2	191964633	G	T	0.7770	0.0133	0.0023	0.0002	0.0002
rs7578047	2	68579931	G	A	0.1323	0.0162	0.0029	0.0001	0.0002
rs7579497	2	71677946	A	G	0.1856	-0.0137	0.0024	0.0000	0.0002
rs7596846	2	220050527	T	G	0.9206	-0.0212	0.0035	0.0001	0.0003
rs77713896	2	120680218	C	T	0.0613	0.0346	0.0039	0.0002	0.0003
rs880114	2	234246458	C	T	0.5024	-0.0163	0.0019	0.0001	0.0002
rs113267846	3	121820906	T	C	0.0601	-0.0253	0.0040	-0.0009	0.0003
rs11505019	3	188094327	G	T	0.4109	-0.0134	0.0019	0.0002	0.0002
rs11707962	3	18760094	C	T	0.1763	0.0341	0.0025	0.0001	0.0002
rs11715679	3	107532069	G	A	0.2318	0.0124	0.0022	0.0001	0.0002
rs11717285	3	142891007	T	C	0.2624	0.0142	0.0021	0.0000	0.0002
rs11928508	3	182739738	G	C	0.7113	-0.0137	0.0021	-0.0001	0.0002
rs12330493	3	27791614	G	T	0.3649	0.0193	0.0017	0.0001	0.0002
rs12636926	3	141660742	C	T	0.7296	-0.0136	0.0021	0.0001	0.0002
rs13065854	3	24234232	T	A	0.1908	0.0140	0.0021	0.0000	0.0002
rs13068211	3	14146712	A	G	0.0602	0.0223	0.0040	0.0005	0.0004
rs1354034	3	56849749	C	T	0.6002	0.0227	0.0019	0.0003	0.0002
rs140763487	3	112686528	T	C	0.0143	-0.0489	0.0080	-0.0003	0.0007
rs17776120	3	186732679	A	C	0.3839	-0.0118	0.0019	0.0001	0.0002
rs1822534	3	12266804	G	A	0.3940	-0.0344	0.0019	0.0000	0.0002
rs1991431	3	141133450	A	G	0.4405	0.0238	0.0019	-0.0001	0.0002
rs2089979	3	196501413	G	A	0.4144	-0.0345	0.0019	0.0000	0.0002
rs2100139	3	108504265	C	T	0.6584	0.0152	0.0020	0.0003	0.0002
rs2245278	3	129286635	A	G	0.2860	-0.0136	0.0021	0.0000	0.0002
rs2291900	3	170825920	A	G	0.2905	0.0169	0.0021	-0.0002	0.0002
rs2713602	3	128203035	G	A	0.5826	0.0161	0.0019	0.0000	0.0002
rs34850939	3	47063910	A	G	0.3461	0.0481	0.0021	0.0001	0.0002
rs35592432	3	71355240	C	G	0.0273	0.1085	0.0062	0.0008	0.0005
rs3732378	3	39307162	A	G	0.1727	0.0348	0.0025	0.0005	0.0002
rs4148593	3	183649870	G	A	0.3824	0.0107	0.0019	-0.0001	0.0002
rs56850780	3	195926731	T	G	0.0777	-0.0214	0.0036	0.0004	0.0003
rs62262391	3	123105119	T	C	0.2247	-0.0259	0.0023	0.0001	0.0002
rs66815886	3	64703394	T	G	0.2799	-0.0126	0.0021	0.0001	0.0002
rs6774387	3	4749545	T	C	0.4037	-0.0111	0.0019	0.0002	0.0002
rs6809697	3	71414864	A	G	0.1364	-0.0182	0.0028	-0.0003	0.0002
rs73073149	3	194193629	T	A	0.0106	-0.0541	0.0094	0.0015	0.0008
rs73187869	3	119113344	A	G	0.1006	0.0181	0.0031	-0.0002	0.0003
rs74280151	3	28287735	T	C	0.1748	0.0288	0.0022	0.0001	0.0002
rs7617331	3	56624159	C	T	0.5304	0.0160	0.0019	0.0003	0.0002
rs7627511	3	171178037	C	G	0.2190	-0.0170	0.0024	-0.0001	0.0002
rs76332743	3	187705133	C	A	0.0811	-0.0249	0.0035	0.0008	0.0003
rs76830965	3	159637678	A	C	0.1167	-0.0208	0.0030	-0.0001	0.0003
rs9835571	3	136523475	G	T	0.6898	0.0220	0.0020	0.0001	0.0002
rs9864216	3	151007262	A	G	0.7764	0.0237	0.0023	-0.0002	0.0002
rs9865811	3	63994505	A	G	0.3017	0.0121	0.0021	-0.0004	0.0002
rs10003447	4	148447379	T	C	0.2543	0.0138	0.0023	0.0000	0.0002
rs10006495	4	120263315	G	T	0.3584	-0.0111	0.0020	0.0001	0.0002
rs10027415	4	80898027	T	C	0.5295	-0.0163	0.0014	-0.0002	0.0002
rs12640189	4	6893181	T	C	0.1758	0.0164	0.0026	-0.0002	0.0002
rs13105987	4	38602467	A	G	0.2742	0.0457	0.0021	-0.0002	0.0002
rs13135381	4	102743353	G	A	0.3143	0.0118	0.0020	0.0000	0.0002
rs1344603	4	38109847	C	T	0.6757	0.0293	0.0020	0.0003	0.0002
rs1431545	4	90220659	T	C	0.6533	0.0211	0.0020	0.0002	0.0002
rs17005891	4	83547862	A	G	0.1844	-0.0243	0.0018	0.0000	0.0002
rs17581460	4	38456404	C	T	0.0197	-0.0404	0.0069	0.0006	0.0006
rs17703261	4	88058337	T	A	0.1886	0.0096	0.0017	-0.0002	0.0002
rs2298850	4	72614267	C	G	0.2782	-0.0142	0.0021	-0.0001	0.0002

rs309375	4	123681156	A	C	0.5747	0.0192	0.0020	0.0000	0.0002
rs4696314	4	153005716	C	T	0.5151	0.0142	0.0019	-0.0002	0.0002
rs4834240	4	113116179	T	C	0.3032	-0.0221	0.0021	0.0000	0.0002
rs5026470	4	103554722	G	A	0.5173	0.0369	0.0019	0.0000	0.0002
rs58408429	4	57769824	C	T	0.1861	-0.0236	0.0024	-0.0004	0.0002
rs62334097	4	169301245	G	A	0.1125	-0.0188	0.0030	0.0004	0.0003
rs6531629	4	38358752	C	T	0.7033	0.0645	0.0021	0.0000	0.0002
rs6554158	4	55066077	A	G	0.2312	0.0142	0.0022	-0.0004	0.0002
rs6850557	4	110911015	A	G	0.3160	-0.0198	0.0020	-0.0003	0.0002
rs73142294	4	40301624	T	C	0.2476	0.0152	0.0022	0.0000	0.0002
rs74408817	4	157801752	A	C	0.0808	-0.0234	0.0035	-0.0002	0.0003
rs7661349	4	106066982	C	T	0.6368	0.0252	0.0020	-0.0001	0.0002
rs7672879	4	122811172	A	T	0.3528	0.0135	0.0020	-0.0003	0.0002
rs7696969	4	143326714	G	T	0.6199	-0.0125	0.0020	0.0000	0.0002
rs10076701	5	131596419	C	T	0.5572	-0.0228	0.0019	-0.0003	0.0002
rs11134475	5	156399950	G	A	0.6358	-0.0142	0.0020	-0.0001	0.0002
rs114378220	5	110566360	T	C	0.0736	-0.0380	0.0038	-0.0008	0.0003
rs11567701	5	35859863	T	G	0.2616	0.0544	0.0021	-0.0002	0.0002
rs11742873	5	179244302	T	C	0.8374	-0.0166	0.0026	-0.0004	0.0002
rs12656316	5	126191707	C	A	0.4277	-0.0115	0.0019	0.0000	0.0002
rs1443693	5	112464953	C	T	0.6800	-0.0114	0.0020	0.0000	0.0002
rs17055818	5	157969475	C	T	0.2523	-0.0297	0.0022	-0.0001	0.0002
rs17728338	5	150478318	A	G	0.0552	-0.0283	0.0043	-0.0001	0.0004
rs1862191	5	52328400	T	C	0.3195	0.0117	0.0020	-0.0002	0.0002
rs1862362	5	150530679	G	A	0.4423	-0.0175	0.0019	-0.0001	0.0002
rs1864054	5	67300771	G	C	0.1094	0.0194	0.0031	-0.0001	0.0003
rs247557	5	88710112	G	T	0.5242	-0.0110	0.0019	0.0000	0.0002
rs251398	5	67509801	C	T	0.4469	-0.0173	0.0019	-0.0001	0.0002
rs2548256	5	100148462	T	G	0.6201	0.0140	0.0019	-0.0002	0.0002
rs2548499	5	52072544	G	A	0.2082	0.0190	0.0023	0.0001	0.0002
rs28710104	5	54879845	G	A	0.6833	-0.0143	0.0020	-0.0002	0.0002
rs2910580	5	57527886	A	T	0.6591	-0.0139	0.0020	0.0000	0.0002
rs304154	5	88120416	C	T	0.3080	0.0120	0.0020	0.0001	0.0002
rs360017	5	173207353	G	A	0.7753	-0.0211	0.0023	-0.0003	0.0002
rs3749748	5	127350549	T	C	0.2467	-0.0260	0.0022	0.0001	0.0002
rs3992621	5	118530823	C	T	0.5356	-0.0104	0.0019	-0.0002	0.0002
rs419470	5	16625591	G	A	0.7166	0.0150	0.0021	-0.0002	0.0002
rs4406174	5	1519079	T	C	0.1012	0.0184	0.0033	0.0001	0.0003
rs464609	5	34654477	A	G	0.5443	0.0118	0.0019	-0.0003	0.0002
rs4866971	5	43151537	T	C	0.7064	0.0115	0.0021	0.0000	0.0002
rs4976642	5	176673068	A	T	0.8314	0.0244	0.0025	0.0001	0.0002
rs55977949	5	158375669	A	G	0.0976	-0.0305	0.0032	-0.0003	0.0003
rs58265751	5	124377841	C	T	0.5208	-0.0122	0.0019	-0.0002	0.0002
rs60580948	5	148340270	G	A	0.1401	-0.0178	0.0028	0.0002	0.0002
rs678393	5	72418309	G	T	0.2617	-0.0154	0.0022	0.0000	0.0002
rs6882776	5	172664163	A	G	0.2842	0.0131	0.0021	0.0004	0.0002
rs73239280	5	118695579	A	G	0.2033	-0.0315	0.0023	-0.0003	0.0002
rs7723153	5	147877724	A	G	0.4195	-0.0123	0.0019	0.0002	0.0002
rs7736615	5	43570686	G	C	0.5080	0.0132	0.0019	-0.0001	0.0002
rs79237520	5	134712566	T	C	0.0228	0.0854	0.0064	0.0000	0.0005
rs819591	5	72843780	C	T	0.2287	-0.0137	0.0023	0.0000	0.0002
rs1050979	6	410417	G	A	0.5252	0.0245	0.0019	0.0003	0.0002
rs1132352	6	24806070	C	A	0.5389	-0.0240	0.0019	0.0001	0.0002
rs12214623	6	113387068	T	C	0.2011	-0.0188	0.0024	0.0003	0.0002
rs1226013	6	10539289	G	T	0.5512	-0.0117	0.0019	0.0000	0.0002
rs12526696	6	108053364	A	G	0.1606	0.0374	0.0027	0.0000	0.0002
rs1297770	6	7158225	T	G	0.1892	0.0238	0.0024	0.0000	0.0002
rs13199289	6	44830222	C	T	0.4696	0.0133	0.0019	-0.0002	0.0002

rs13199519	6	109709175	A	G	0.2985	-0.0141	0.0021	0.0001	0.0002
rs13203475	6	149568231	A	G	0.3566	0.0113	0.0020	-0.0002	0.0002
rs1341235	6	22268222	C	T	0.5261	0.0151	0.0019	-0.0002	0.0002
rs1343658	6	127263275	G	A	0.4598	-0.0122	0.0019	0.0003	0.0002
rs137928493	6	30948693	A	C	0.0131	0.0697	0.0083	-0.0001	0.0008
rs142761146	6	138004508	A	T	0.1821	0.0153	0.0025	0.0001	0.0002
rs17119	6	14719496	A	G	0.8031	-0.0172	0.0024	0.0002	0.0002
rs17710008	6	153043035	A	G	0.1817	0.0206	0.0025	-0.0004	0.0002
rs183120114	6	31352678	G	A	0.0160	-0.0894	0.0076	0.0002	0.0006
rs2273215	6	170586082	A	G	0.4582	-0.0150	0.0020	-0.0003	0.0002
rs2294861	6	53136516	T	C	0.2941	0.0190	0.0021	0.0001	0.0002
rs2523978	6	30083708	T	C	0.1263	-0.0864	0.0029	-0.0007	0.0002
rs3012415	6	170489464	T	C	0.8229	0.0143	0.0026	0.0002	0.0002
rs318493	6	2904791	G	A	0.2713	0.0129	0.0022	-0.0001	0.0002
rs34451538	6	31487454	A	G	0.0295	-0.0770	0.0056	-0.0005	0.0005
rs4711790	6	44573822	T	C	0.3402	-0.0173	0.0020	-0.0002	0.0002
rs4714431	6	41168337	C	A	0.3711	-0.0108	0.0020	-0.0006	0.0002
rs4869745	6	151908076	T	C	0.2894	0.0126	0.0021	0.0002	0.0002
rs62428835	6	143263836	T	G	0.1631	0.0148	0.0026	0.0003	0.0002
rs6569648	6	130349119	T	C	0.7613	0.0187	0.0022	0.0000	0.0002
rs6918542	6	21817932	T	C	0.1546	0.0235	0.0026	0.0001	0.0002
rs6926219	6	122720806	A	G	0.5473	0.0268	0.0019	0.0001	0.0002
rs6936325	6	159432659	A	G	0.0247	-0.0448	0.0061	0.0013	0.0005
rs707927	6	31745518	G	A	0.0272	0.1126	0.0058	0.0001	0.0005
rs72835315	6	231878	C	T	0.0602	0.0351	0.0042	-0.0006	0.0004
rs72843213	6	32299173	T	G	0.0576	0.1202	0.0041	0.0005	0.0003
rs72928038	6	90976768	A	G	0.1769	-0.0333	0.0025	0.0001	0.0002
rs74526869	6	42024634	A	G	0.1254	0.0348	0.0029	0.0003	0.0002
rs77176370	6	25042978	C	G	0.0427	-0.0397	0.0047	-0.0004	0.0004
rs7776054	6	135418916	G	A	0.2608	-0.0401	0.0022	0.0001	0.0002
rs8192587	6	32188713	G	C	0.0231	0.0699	0.0063	0.0005	0.0006
rs9268233	6	32289292	A	G	0.0149	0.0505	0.0078	0.0003	0.0007
rs9328393	6	6902939	A	C	0.6684	0.0234	0.0020	-0.0001	0.0002
rs9396582	6	15326802	G	C	0.5873	0.0160	0.0019	0.0000	0.0002
rs9942489	6	35323709	A	T	0.0375	-0.0694	0.0050	0.0003	0.0004
rs10230506	7	92471518	A	T	0.1609	0.0212	0.0026	0.0002	0.0002
rs10235989	7	40000787	A	G	0.3561	-0.0194	0.0020	-0.0002	0.0002
rs10237524	7	56036024	A	G	0.3302	-0.0192	0.0020	0.0000	0.0002
rs10245472	7	55147478	A	G	0.1766	-0.0159	0.0025	0.0000	0.0002
rs10486847	7	84674997	A	G	0.2885	0.0142	0.0021	0.0001	0.0002
rs11761199	7	128581835	G	A	0.4543	-0.0108	0.0019	0.0001	0.0002
rs117960080	7	100273085	C	T	0.0354	0.0369	0.0052	0.0007	0.0005
rs12534473	7	106374548	G	T	0.4768	0.0105	0.0019	-0.0002	0.0002
rs149984502	7	50539036	T	C	0.0186	0.0479	0.0071	-0.0007	0.0006
rs1637366	7	74360784	G	C	0.2894	0.0142	0.0022	0.0000	0.0002
rs17138740	7	18224597	G	A	0.2145	-0.0135	0.0023	-0.0001	0.0002
rs2282867	7	14015318	A	T	0.7167	-0.0196	0.0021	-0.0001	0.0002
rs2347784	7	6524843	G	C	0.2682	0.0281	0.0022	0.0002	0.0002
rs2710804	7	36084529	C	T	0.3761	0.0138	0.0020	0.0004	0.0002
rs2727487	7	129513820	C	A	0.6292	-0.0126	0.0020	0.0001	0.0002
rs2908425	7	5569327	A	G	0.3322	0.0142	0.0020	-0.0006	0.0002
rs35114152	7	150294476	G	A	0.2256	0.0247	0.0023	-0.0002	0.0002
rs35121828	7	23634985	A	G	0.2590	0.0131	0.0022	0.0001	0.0002
rs35345753	7	22740513	G	C	0.2071	-0.0265	0.0023	-0.0004	0.0002
rs3735485	7	45009341	G	A	0.8448	0.0444	0.0026	0.0001	0.0002
rs3735609	7	107383744	G	C	0.2477	-0.0147	0.0022	-0.0001	0.0002
rs3812316	7	73020337	G	C	0.1275	-0.0167	0.0028	0.0003	0.0002
rs4718976	7	70113409	C	T	0.6945	-0.0179	0.0021	0.0002	0.0002

rs4722008	7	2768897	T	C	0.4219	-0.0132	0.0019	0.0000	0.0002
rs4722840	7	985201	G	A	0.4329	-0.0141	0.0020	-0.0001	0.0002
rs62485905	7	138741586	T	C	0.5515	-0.0177	0.0019	-0.0001	0.0002
rs62621812	7	127015083	A	G	0.0207	0.0560	0.0068	-0.0006	0.0006
rs6462758	7	37386272	T	C	0.5194	-0.0122	0.0019	0.0000	0.0002
rs6953822	7	134576441	C	T	0.8312	0.0139	0.0025	-0.0001	0.0002
rs6955702	7	3157702	G	A	0.5221	-0.0223	0.0019	0.0001	0.0002
rs6967605	7	156636308	G	C	0.4266	-0.0128	0.0019	-0.0002	0.0002
rs73165514	7	150527651	A	G	0.0864	0.0209	0.0034	0.0001	0.0003
rs75049211	7	115849440	T	C	0.0398	-0.0307	0.0049	0.0000	0.0004
rs7780328	7	99256844	G	A	0.0671	0.0373	0.0038	0.0000	0.0003
rs7790229	7	97919338	A	G	0.4930	0.0121	0.0019	-0.0003	0.0002
rs7802739	7	130749033	T	C	0.7930	-0.0222	0.0023	0.0001	0.0002
rs9648346	7	28160113	G	C	0.2032	-0.0244	0.0023	-0.0006	0.0002
rs9920	7	116200092	C	T	0.1047	0.0294	0.0031	0.0004	0.0003
rs10107362	8	130986984	G	C	0.3606	0.0128	0.0020	0.0002	0.0002
rs10109370	8	72909349	A	C	0.2385	-0.0189	0.0022	0.0001	0.0002
rs10503147	8	421777	T	C	0.0889	0.0207	0.0034	0.0000	0.0003
rs10956401	8	129002419	A	G	0.3451	-0.0253	0.0020	-0.0001	0.0002
rs113142693	8	30242076	C	T	0.2475	-0.0171	0.0022	-0.0001	0.0002
rs117934175	8	79069520	A	G	0.0405	-0.0446	0.0049	0.0003	0.0004
rs12542907	8	68813188	G	C	0.3989	-0.0111	0.0019	0.0001	0.0002
rs1264593	8	102471269	T	G	0.4949	0.0146	0.0019	0.0001	0.0002
rs13262073	8	56760360	G	C	0.6876	-0.0119	0.0021	0.0002	0.0002
rs1384804	8	79572222	A	C	0.7481	-0.0502	0.0022	0.0001	0.0002
rs1533065	8	22785768	T	C	0.3452	0.0167	0.0020	0.0000	0.0002
rs17180155	8	105807849	C	T	0.3940	-0.0111	0.0019	0.0000	0.0002
rs2055101	8	119886923	T	C	0.5272	-0.0134	0.0019	0.0001	0.0002
rs2322599	8	27211910	A	G	0.3658	-0.0115	0.0020	-0.0001	0.0002
rs2453628	8	101139413	A	G	0.5258	0.0108	0.0019	0.0001	0.0002
rs28588745	8	10647044	T	A	0.2057	0.0225	0.0023	-0.0001	0.0002
rs2980821	8	40028306	A	C	0.4251	-0.0132	0.0019	-0.0001	0.0002
rs2980888	8	126507308	C	T	0.6971	-0.0153	0.0021	0.0003	0.0002
rs4737010	8	41630447	A	G	0.2289	0.0268	0.0023	0.0002	0.0002
rs56094005	8	21769432	G	A	0.0429	-0.0401	0.0047	-0.0001	0.0004
rs6474359	8	41549194	C	T	0.0377	-0.0514	0.0050	-0.0003	0.0004
rs6586777	8	18702471	A	C	0.4378	-0.0128	0.0019	0.0002	0.0002
rs7007986	8	142217115	A	G	0.6012	-0.0220	0.0020	0.0000	0.0002
rs71518536	8	11647389	T	C	0.1181	0.0182	0.0030	0.0001	0.0003
rs72637694	8	49288118	A	G	0.1881	-0.0140	0.0024	0.0003	0.0002
rs75653581	8	78363128	T	C	0.0127	0.0611	0.0089	-0.0003	0.0007
rs7839516	8	61386533	C	T	0.3694	0.0155	0.0020	0.0001	0.0002
rs881492	8	101484638	G	C	0.4675	-0.0190	0.0019	-0.0002	0.0002
rs1002607	9	113858933	C	T	0.5929	-0.0130	0.0019	0.0003	0.0002
rs10814138	9	34723035	A	C	0.3139	-0.0137	0.0021	0.0001	0.0002
rs10973700	9	38196117	C	G	0.4872	-0.0190	0.0019	-0.0002	0.0002
rs10985915	9	126018715	G	A	0.1106	-0.0226	0.0030	0.0001	0.0003
rs10986338	9	127191232	A	G	0.6498	-0.0116	0.0020	0.0001	0.0002
rs11541908	9	35705759	A	G	0.2772	0.0204	0.0021	0.0005	0.0002
rs12336678	9	21785894	T	C	0.0993	-0.0217	0.0032	0.0002	0.0003
rs12379227	9	92173732	G	A	0.2088	-0.0137	0.0024	0.0002	0.0002
rs1971429	9	33776182	A	C	0.7290	0.0128	0.0021	-0.0001	0.0002
rs2065500	9	22145694	G	A	0.1598	-0.0480	0.0026	0.0001	0.0002
rs2313532	9	133635321	A	G	0.4008	-0.0143	0.0019	0.0002	0.0002
rs2807303	9	82187095	A	G	0.3462	0.0216	0.0020	0.0000	0.0002
rs3731211	9	21986847	A	T	0.7198	0.0436	0.0021	-0.0002	0.0002
rs3761846	9	123689597	T	C	0.5695	0.0205	0.0019	0.0005	0.0002
rs4743469	9	98788301	A	G	0.0367	-0.0325	0.0050	-0.0005	0.0004

rs4880192	9	139927062	G	A	0.7208	0.0294	0.0022	0.0003	0.0002
rs501461	9	4039727	T	G	0.5997	-0.0177	0.0019	-0.0001	0.0002
rs616154	9	136150466	T	C	0.5145	-0.0104	0.0019	0.0001	0.0002
rs61750929	9	91495135	T	C	0.0563	-0.0703	0.0041	0.0004	0.0004
rs62565782	9	86600887	A	G	0.1309	-0.0216	0.0029	-0.0002	0.0002
rs6474820	9	14142907	C	T	0.1696	0.0140	0.0025	0.0004	0.0002
rs6475476	9	20802452	G	A	0.3731	0.0108	0.0020	0.0000	0.0002
rs7855586	9	112678121	G	A	0.2901	0.0149	0.0021	-0.0001	0.0002
rs7870106	9	99210584	A	G	0.2290	-0.0132	0.0023	-0.0003	0.0002
rs10748526	10	82273079	C	T	0.7935	0.0152	0.0023	0.0001	0.0002
rs10762264	10	70976833	A	G	0.6807	-0.0204	0.0020	0.0000	0.0002
rs10794175	10	126358073	T	G	0.4160	-0.0213	0.0020	0.0001	0.0002
rs10828725	10	25218243	T	G	0.3661	-0.0321	0.0020	0.0003	0.0002
rs10995312	10	64566572	C	T	0.1263	0.0261	0.0029	0.0002	0.0002
rs11189058	10	99001258	A	G	0.6604	0.0123	0.0020	0.0000	0.0002
rs11574852	10	104161475	C	A	0.0375	-0.0286	0.0050	0.0003	0.0004
rs12247907	10	65317045	C	G	0.4851	0.0141	0.0019	0.0000	0.0002
rs1227067	10	73487017	C	T	0.3275	0.0122	0.0021	0.0000	0.0002
rs1399180	10	8098719	C	T	0.8290	0.0258	0.0025	0.0000	0.0002
rs1923403	10	4160744	G	A	0.9221	0.0200	0.0035	-0.0012	0.0003
rs2226260	10	5159597	C	T	0.8244	0.0144	0.0025	0.0001	0.0002
rs2249989	10	73105421	G	C	0.7492	-0.0226	0.0022	0.0002	0.0002
rs2462017	10	30486133	A	G	0.6523	0.0140	0.0020	0.0000	0.0002
rs2992263	10	26741207	A	G	0.6100	-0.0203	0.0019	0.0003	0.0002
rs3118469	10	6101129	T	A	0.3000	0.0292	0.0021	0.0002	0.0002
rs34487722	10	116008888	C	T	0.1592	0.0215	0.0026	0.0001	0.0002
rs35001362	10	14958758	A	C	0.2358	-0.0151	0.0022	0.0002	0.0002
rs41317270	10	98369755	T	C	0.1773	-0.0185	0.0025	0.0003	0.0002
rs4948492	10	63719739	T	C	0.6617	-0.0219	0.0020	0.0000	0.0002
rs4948822	10	44513645	G	T	0.2316	0.0127	0.0022	0.0000	0.0002
rs55851824	10	94515006	A	G	0.1619	0.0201	0.0026	0.0000	0.0002
rs58667319	10	96524395	T	C	0.1230	-0.0232	0.0029	-0.0002	0.0002
rs603424	10	102075479	A	G	0.1745	0.0205	0.0025	0.0001	0.0002
rs61850684	10	50252936	A	G	0.2335	-0.0325	0.0022	-0.0003	0.0002
rs7071131	10	121026712	G	A	0.4951	-0.0160	0.0020	0.0002	0.0002
rs7096430	10	92764460	G	A	0.5740	-0.0108	0.0019	-0.0002	0.0002
rs748113	10	73508791	C	T	0.4363	-0.0343	0.0020	0.0000	0.0002
rs78051198	10	44887215	G	T	0.0924	-0.0274	0.0033	-0.0002	0.0003
rs7893800	10	27125289	A	G	0.3898	0.0107	0.0019	-0.0001	0.0002
rs10047467	11	101761011	G	A	0.0985	0.0198	0.0032	-0.0005	0.0003
rs1017875	11	47999218	T	C	0.2017	-0.0361	0.0024	0.0001	0.0002
rs10838634	11	46929954	G	A	0.9015	-0.0261	0.0032	-0.0001	0.0003
rs11022177	11	12133478	G	C	0.3109	-0.0121	0.0021	0.0003	0.0002
rs11033388	11	36074218	T	A	0.4188	0.0196	0.0019	0.0002	0.0002
rs1111949	11	121590410	T	G	0.6882	-0.0113	0.0021	-0.0004	0.0002
rs11218725	11	122509237	G	A	0.3765	0.0330	0.0020	-0.0001	0.0002
rs11218767	11	122583862	G	A	0.8536	0.0174	0.0027	0.0001	0.0002
rs11224302	11	100456604	T	C	0.0988	-0.0383	0.0032	0.0006	0.0003
rs11230661	11	55451313	A	G	0.1564	-0.0259	0.0028	-0.0001	0.0002
rs11246147	11	396701	T	C	0.5494	0.0114	0.0020	0.0000	0.0002
rs1788973	11	70123737	C	T	0.3498	0.0135	0.0020	0.0001	0.0002
rs1893033	11	119089055	T	C	0.0746	-0.0282	0.0036	-0.0002	0.0003
rs189426461	11	108326327	A	G	0.0136	0.0671	0.0083	-0.0004	0.0007
rs2294081	11	193863	C	T	0.4807	0.0156	0.0020	0.0000	0.0002
rs2298559	11	60274640	C	T	0.4254	0.0154	0.0019	0.0000	0.0002
rs238914	11	113984109	A	C	0.3943	0.0123	0.0019	-0.0001	0.0002
rs2403246	11	18101078	G	C	0.4145	0.0120	0.0019	0.0001	0.0002
rs34662054	11	68908815	C	G	0.2991	0.0143	0.0021	0.0002	0.0002

rs36020612	11	123344435	T	C	0.1943	0.0207	0.0024	-0.0002	0.0002
rs520734	11	128100120	A	G	0.2897	0.0118	0.0021	0.0000	0.0002
rs61867141	11	1874892	A	G	0.1949	-0.0171	0.0025	0.0000	0.0002
rs6592656	11	76368054	G	C	0.5228	0.0107	0.0019	-0.0001	0.0002
rs7127911	11	128492549	T	G	0.2664	0.0241	0.0021	0.0002	0.0002
rs74679312	11	62194434	G	A	0.0567	0.0313	0.0043	-0.0005	0.0004
rs77876222	11	67133248	T	C	0.0196	0.0447	0.0071	-0.0010	0.0006
rs79805393	11	128412297	C	T	0.1730	0.0170	0.0025	-0.0002	0.0002
rs934177	11	44612714	G	C	0.4368	-0.0142	0.0019	0.0000	0.0002
rs949349	11	86297189	C	T	0.2711	0.0142	0.0021	0.0006	0.0002
rs10466905	12	6502832	A	G	0.1900	0.0620	0.0025	0.0000	0.0002
rs10846577	12	124400261	C	T	0.4421	-0.0141	0.0019	0.0000	0.0002
rs11045171	12	20470199	G	A	0.1981	-0.0155	0.0024	0.0001	0.0002
rs11168249	12	48208368	C	T	0.4613	-0.0195	0.0019	-0.0002	0.0002
rs1118866	12	68520751	T	C	0.7035	0.0148	0.0021	0.0000	0.0002
rs1131017	12	56435929	G	C	0.5727	0.0136	0.0019	-0.0001	0.0002
rs115647629	12	111811903	A	G	0.0265	-0.0639	0.0063	0.0001	0.0005
rs12299943	12	96611648	G	A	0.3019	0.0124	0.0021	-0.0001	0.0002
rs1265564	12	111708458	C	A	0.4432	0.0716	0.0019	-0.0002	0.0002
rs12811832	12	48376213	A	G	0.3539	-0.0162	0.0020	0.0001	0.0002
rs1371076	12	90561450	C	T	0.7404	0.0149	0.0022	0.0002	0.0002
rs147700878	12	32635390	A	G	0.0438	0.0259	0.0046	0.0004	0.0004
rs17041439	12	101873240	C	A	0.0565	0.0409	0.0041	0.0000	0.0004
rs2137434	12	122153496	A	G	0.3804	0.0114	0.0020	0.0001	0.0002
rs2269899	12	113381956	T	C	0.6440	-0.0135	0.0020	0.0000	0.0002
rs28533432	12	123873242	T	C	0.6996	-0.0251	0.0021	0.0000	0.0002
rs34038797	12	740009	G	C	0.4812	-0.0193	0.0020	0.0001	0.0002
rs34327	12	12873748	C	T	0.5231	-0.0259	0.0019	0.0001	0.0002
rs3741494	12	133464500	T	C	0.0954	-0.0215	0.0034	0.0004	0.0003
rs4149577	12	6447522	A	G	0.5170	-0.0233	0.0019	0.0001	0.0002
rs4760278	12	57771153	A	C	0.2227	-0.0140	0.0023	0.0001	0.0002
rs4764803	12	101898230	G	C	0.6170	0.0142	0.0020	0.0000	0.0002
rs550349	12	121236160	C	A	0.7185	-0.0131	0.0021	0.0000	0.0002
rs6487543	12	26438189	A	G	0.7710	0.0197	0.0023	0.0003	0.0002
rs6490291	12	112177775	A	T	0.9641	-0.0556	0.0058	0.0002	0.0005
rs67516712	12	124201524	A	G	0.4225	0.0149	0.0019	-0.0001	0.0002
rs7138945	12	50539419	G	T	0.3635	0.0123	0.0020	0.0003	0.0002
rs7308380	12	89851232	C	A	0.7505	0.0160	0.0022	0.0002	0.0002
rs73105786	12	49138958	A	G	0.3194	-0.0152	0.0020	0.0002	0.0002
rs7488780	12	20579392	C	G	0.2045	-0.0135	0.0024	-0.0001	0.0002
rs76362970	12	96193112	G	A	0.1921	-0.0134	0.0024	-0.0002	0.0002
rs7955734	12	4333159	G	C	0.2101	-0.0162	0.0023	-0.0001	0.0002
rs7967230	12	12541869	A	G	0.5137	0.0133	0.0019	0.0001	0.0002
rs937283	12	69202164	G	A	0.4052	0.0106	0.0019	0.0001	0.0002
rs12586002	13	100046106	G	T	0.3528	-0.0137	0.0020	-0.0001	0.0002
rs1320472	13	114843162	C	T	0.4612	-0.0137	0.0019	0.0000	0.0002
rs146851424	13	50377910	C	A	0.0220	-0.0519	0.0065	-0.0002	0.0006
rs1523178	13	50652456	C	A	0.2211	-0.0257	0.0023	0.0001	0.0002
rs198612	13	48976855	G	A	0.2718	0.0150	0.0021	0.0000	0.0002
rs2847468	13	110928439	T	C	0.2637	-0.0119	0.0022	-0.0001	0.0002
rs3812849	13	74701736	C	A	0.2662	0.0435	0.0021	0.0001	0.0002
rs67483792	13	72503638	T	C	0.0429	-0.0354	0.0047	0.0001	0.0004
rs76428106	13	28604007	C	T	0.0134	0.0628	0.0087	-0.0006	0.0007
rs797496	13	51270494	T	G	0.0917	0.0194	0.0033	0.0002	0.0003
rs9525619	13	42992135	T	C	0.5343	-0.0203	0.0019	0.0002	0.0002
rs9532679	13	41522338	C	A	0.1490	-0.0323	0.0027	-0.0001	0.0002
rs9534338	13	46707137	C	T	0.4599	0.0151	0.0019	0.0000	0.0002
rs9590390	13	114920867	A	G	0.2919	0.0301	0.0021	0.0002	0.0002

rs9592965	13	74615925	C	A	0.3270	-0.0144	0.0020	0.0001	0.0002
rs10145277	14	99793449	A	T	0.6247	-0.0149	0.0020	0.0000	0.0002
rs11160706	14	103361736	A	G	0.2222	0.0305	0.0023	-0.0001	0.0002
rs112992671	14	50412380	A	G	0.0459	-0.0267	0.0046	-0.0003	0.0004
rs11499034	14	81972441	C	T	0.0143	-0.0518	0.0080	-0.0003	0.0007
rs1204997	14	72183309	C	T	0.5559	0.0105	0.0019	0.0001	0.0002
rs175714	14	75981856	C	T	0.5775	0.0261	0.0019	-0.0001	0.0002
rs1966865	14	64975861	A	G	0.3173	0.0271	0.0020	0.0000	0.0002
rs2057340	14	35848774	G	A	0.6534	0.0138	0.0020	0.0001	0.0002
rs28688110	14	93511785	T	A	0.1106	0.0207	0.0031	0.0003	0.0003
rs45528934	14	23793305	T	C	0.1618	-0.0205	0.0026	0.0001	0.0002
rs4902750	14	70186440	A	G	0.1587	-0.0144	0.0026	0.0001	0.0002
rs55867915	14	69186660	T	A	0.0762	-0.0303	0.0036	-0.0003	0.0003
rs696	14	35871093	T	C	0.3648	0.0254	0.0020	0.0001	0.0002
rs8004780	14	103853360	C	A	0.3664	0.0183	0.0020	-0.0003	0.0002
rs1075619	15	83781474	C	T	0.2214	0.0171	0.0023	-0.0004	0.0002
rs1382538	15	86165896	C	G	0.2608	0.0119	0.0022	-0.0005	0.0002
rs139974673	15	44027885	C	T	0.0260	0.0541	0.0060	0.0006	0.0005
rs149453951	15	69583434	T	C	0.0236	0.0486	0.0067	-0.0005	0.0006
rs187856913	15	90555181	A	G	0.0388	0.0482	0.0050	0.0008	0.0004
rs2412544	15	40949526	T	C	0.3936	-0.0162	0.0019	0.0001	0.0002
rs2439408	15	66925163	C	G	0.5611	-0.0125	0.0019	0.0000	0.0002
rs28539372	15	91021412	A	T	0.3191	0.0381	0.0020	0.0002	0.0002
rs34025077	15	70033578	G	A	0.1014	-0.0328	0.0031	-0.0004	0.0003
rs3926279	15	81599443	G	A	0.0910	0.0305	0.0033	-0.0003	0.0003
rs62019188	15	90645265	A	G	0.5737	-0.0130	0.0019	-0.0002	0.0002
rs631864	15	70376441	C	T	0.4717	0.0139	0.0020	0.0001	0.0002
rs6938	15	75136261	G	C	0.6935	-0.0142	0.0021	0.0000	0.0002
rs7161799	15	58770523	T	C	0.0762	0.0341	0.0036	-0.0004	0.0003
rs7182177	15	48646554	G	T	0.1623	-0.0190	0.0026	0.0001	0.0002
rs78099598	15	50991569	G	T	0.1963	-0.0186	0.0024	0.0001	0.0002
rs117556162	16	67680806	A	G	0.0576	0.0445	0.0041	-0.0003	0.0003
rs12598978	16	30482540	G	T	0.5196	0.0551	0.0019	0.0000	0.0002
rs145719494	16	24044921	G	A	0.0588	-0.0309	0.0041	0.0004	0.0003
rs1677490	16	9028541	C	G	0.7912	0.0159	0.0023	0.0002	0.0002
rs17229044	16	11062936	T	C	0.2101	-0.0192	0.0023	0.0000	0.0002
rs247826	16	84582965	T	C	0.2207	0.0387	0.0023	-0.0002	0.0002
rs252491	16	29158794	G	A	0.5144	0.0144	0.0019	0.0001	0.0002
rs2738499	16	78568416	G	A	0.7934	0.0163	0.0023	0.0002	0.0002
rs28853644	16	30801027	T	C	0.2713	-0.0243	0.0021	0.0002	0.0002
rs34140544	16	4105786	C	G	0.6485	0.0170	0.0020	0.0001	0.0002
rs3810818	16	4432029	C	A	0.7734	-0.0182	0.0024	0.0001	0.0002
rs6499144	16	67890388	C	T	0.0510	-0.0296	0.0043	0.0003	0.0004
rs7192652	16	57075180	G	A	0.4806	0.0222	0.0019	-0.0001	0.0002
rs78487935	16	3651579	G	A	0.0777	-0.0236	0.0036	0.0002	0.0003
rs8052370	16	88837298	T	C	0.6095	-0.0210	0.0020	0.0003	0.0002
rs8060375	16	88507538	T	C	0.3213	0.0133	0.0021	0.0001	0.0002
rs9937837	16	31298939	G	T	0.2697	-0.0151	0.0021	0.0000	0.0002
rs9939124	16	75185764	T	C	0.3070	-0.0167	0.0021	0.0003	0.0002
rs10445308	17	37938047	T	C	0.4791	-0.0300	0.0019	-0.0001	0.0002
rs113513990	17	2190390	C	T	0.0971	-0.0377	0.0032	-0.0001	0.0003
rs11652705	17	41761741	G	A	0.2505	-0.0126	0.0022	0.0000	0.0002
rs11652760	17	16786819	G	T	0.1002	-0.0210	0.0032	0.0002	0.0003
rs117499775	17	44078618	C	T	0.0404	-0.0379	0.0051	0.0004	0.0004
rs11869827	17	62021374	T	C	0.3267	0.0125	0.0021	0.0000	0.0002
rs1215	17	7163350	G	A	0.1442	-0.0228	0.0027	-0.0001	0.0002
rs1292069	17	57928290	C	T	0.4496	-0.0225	0.0019	0.0000	0.0002
rs13306780	17	42329004	C	A	0.6973	0.0179	0.0021	0.0003	0.0002

rs1491765	17	73028923	A	G	0.2932	-0.0173	0.0021	-0.0001	0.0002
rs2297508	17	17715317	G	C	0.6476	-0.0224	0.0021	0.0000	0.0002
rs2453582	17	19439066	T	C	0.3913	0.0177	0.0020	-0.0003	0.0002
rs2665960	17	74024711	G	A	0.6686	0.0122	0.0021	0.0001	0.0002
rs35186877	17	4625892	A	G	0.2138	0.0319	0.0023	0.0002	0.0002
rs3809790	17	27955540	T	C	0.4765	-0.0165	0.0019	-0.0002	0.0002
rs4789294	17	74419177	G	A	0.2194	0.0265	0.0023	-0.0003	0.0002
rs59241596	17	81012062	T	C	0.2565	-0.0139	0.0022	0.0002	0.0002
rs62070638	17	29137678	G	A	0.1750	0.0150	0.0025	-0.0004	0.0002
rs62091998	17	2881625	G	A	0.3075	-0.0223	0.0020	0.0001	0.0002
rs6502555	17	2729652	C	T	0.2695	0.0289	0.0023	-0.0001	0.0002
rs72809985	17	8983686	A	G	0.0616	0.0223	0.0039	-0.0002	0.0003
rs7503050	17	66048462	T	G	0.2134	0.0129	0.0023	0.0004	0.0002
rs763333	17	65087308	C	G	0.4806	-0.0124	0.0019	-0.0001	0.0002
rs8069861	17	55010348	A	G	0.1589	-0.0156	0.0026	0.0000	0.0002
rs8070966	17	27196748	C	T	0.8417	-0.0158	0.0026	-0.0007	0.0002
rs8075090	17	4969108	C	T	0.5237	0.0210	0.0019	0.0002	0.0002
rs853198	17	35849342	C	A	0.6396	-0.0204	0.0020	0.0000	0.0002
rs867596	17	76791217	A	G	0.5330	-0.0106	0.0019	0.0000	0.0002
rs9906320	17	72690829	A	G	0.7751	0.0246	0.0023	0.0001	0.0002
rs111626441	18	77250669	A	G	0.0966	0.0278	0.0033	0.0003	0.0003
rs1456059	18	48954037	G	A	0.7663	0.0129	0.0022	-0.0002	0.0002
rs3851820	18	20461322	T	A	0.7450	-0.0176	0.0022	0.0002	0.0002
rs4940572	18	60826343	A	G	0.1365	0.0233	0.0028	0.0001	0.0002
rs4987855	18	60793549	T	C	0.0963	-0.0241	0.0032	-0.0004	0.0003
rs559406	18	12857002	T	G	0.5481	0.0126	0.0019	0.0002	0.0002
rs9676181	18	74769175	A	T	0.5739	0.0118	0.0019	0.0001	0.0002
rs10411704	19	35800662	T	G	0.7910	-0.0129	0.0024	-0.0001	0.0002
rs1077667	19	6668972	T	C	0.2096	-0.0164	0.0023	0.0002	0.0002
rs11084096	19	52128795	A	G	0.2962	-0.0184	0.0021	-0.0003	0.0002
rs12463256	19	297217	A	G	0.2748	0.0146	0.0022	-0.0001	0.0002
rs12981980	19	57050648	A	G	0.2667	-0.0143	0.0021	-0.0001	0.0002
rs138243594	19	16495586	A	G	0.0127	-0.0758	0.0093	-0.0013	0.0008
rs148294866	19	16523646	A	G	0.0087	0.1530	0.0114	0.0001	0.0010
rs148962758	19	16347015	T	C	0.0137	0.0847	0.0085	-0.0005	0.0007
rs157584	19	45396899	C	T	0.4696	-0.0128	0.0019	-0.0004	0.0002
rs2070745	19	52249947	G	C	0.3668	-0.0165	0.0020	-0.0001	0.0002
rs2075022	19	11136315	T	C	0.3512	0.0109	0.0020	-0.0002	0.0002
rs36084354	19	1079959	A	G	0.0917	-0.0607	0.0034	0.0003	0.0003
rs3810276	19	50934939	A	G	0.4046	-0.0118	0.0020	0.0002	0.0002
rs4530278	19	33752994	T	G	0.5982	0.0171	0.0020	0.0005	0.0002
rs4805881	19	33896432	C	A	0.6653	-0.0150	0.0020	0.0000	0.0002
rs5498	19	10395683	G	A	0.4257	-0.0436	0.0020	0.0000	0.0002
rs56179616	19	47695751	T	G	0.2587	0.0312	0.0022	-0.0001	0.0002
rs57398315	19	13900981	A	G	0.2000	0.0317	0.0025	-0.0001	0.0002
rs61387190	19	44260929	T	C	0.1574	0.0316	0.0026	-0.0002	0.0002
rs62111672	19	7415064	A	G	0.0419	0.0629	0.0054	0.0002	0.0004
rs62654154	19	6587264	T	A	0.2887	0.0136	0.0021	-0.0002	0.0002
rs630586	19	46922916	C	A	0.5392	0.0116	0.0019	-0.0004	0.0002
rs68175985	19	13109763	A	G	0.1614	0.0144	0.0026	0.0001	0.0002
rs7252565	19	1192769	A	G	0.7876	0.0276	0.0025	0.0000	0.0002
rs75018496	19	40785683	G	C	0.0760	0.0245	0.0036	0.0004	0.0003
rs79129871	19	16218062	T	C	0.2642	0.0126	0.0023	0.0001	0.0002
rs8101619	19	16410530	T	C	0.4239	-0.0611	0.0019	-0.0001	0.0002
rs897791	19	16490859	G	A	0.9668	-0.0965	0.0055	0.0004	0.0005
rs151332	20	57552121	G	A	0.9285	-0.0237	0.0037	0.0002	0.0003
rs17093026	20	34318911	T	C	0.1015	-0.0219	0.0031	0.0000	0.0003
rs1997797	20	31387954	G	C	0.4457	0.0170	0.0019	-0.0002	0.0002

rs2281559	20	25252161	T	C	0.1804	0.0148	0.0025	-0.0004	0.0002
rs259981	20	57748369	A	T	0.3604	-0.0271	0.0020	0.0002	0.0002
rs4411786	20	1930897	C	T	0.2653	-0.0451	0.0021	-0.0002	0.0002
rs6015470	20	57867952	C	T	0.5379	-0.0123	0.0019	0.0000	0.0002
rs6020560	20	49119419	C	T	0.4702	0.0188	0.0019	0.0000	0.0002
rs6031444	20	42815738	T	G	0.5608	-0.0148	0.0019	-0.0001	0.0002
rs6055955	20	8604181	T	C	0.5085	-0.0271	0.0019	0.0000	0.0002
rs6063965	20	52191200	A	G	0.1137	0.0227	0.0030	0.0005	0.0003
rs6065673	20	42539362	A	G	0.2703	-0.0134	0.0021	0.0001	0.0002
rs6072080	20	39260927	T	C	0.5853	0.0198	0.0019	0.0001	0.0002
rs611847	20	3684022	G	A	0.6355	0.0146	0.0020	0.0005	0.0002
rs62229760	20	50068744	G	A	0.5039	0.0120	0.0019	-0.0002	0.0002
rs817325	20	62595169	G	A	0.5896	0.0118	0.0020	0.0002	0.0002
rs113382956	21	37750829	C	T	0.1437	0.0172	0.0027	0.0001	0.0002
rs1297265	21	16817051	G	A	0.4431	0.0118	0.0019	0.0000	0.0002
rs150797	21	43465831	G	C	0.4474	-0.0136	0.0019	0.0002	0.0002
rs1893592	21	43855067	C	A	0.2960	0.0147	0.0021	-0.0001	0.0002
rs721131	21	47957767	C	T	0.3196	-0.0189	0.0020	0.0000	0.0002
rs165699	22	21178464	A	G	0.4500	0.0110	0.0019	-0.0001	0.0002
rs16986308	22	28717032	A	G	0.1162	-0.0323	0.0030	0.0004	0.0003
rs1807669	22	42122926	T	C	0.7935	-0.0271	0.0023	0.0001	0.0002
rs2238792	22	19983213	T	C	0.2865	0.0218	0.0022	-0.0001	0.0002
rs5759607	22	23497998	A	G	0.2597	-0.0132	0.0022	-0.0001	0.0002
rs5998509	22	21917479	T	C	0.1883	-0.0409	0.0025	-0.0001	0.0002
rs6001858	22	40672078	G	A	0.7356	-0.0153	0.0022	-0.0003	0.0002
rs62237815	22	31993652	G	T	0.0787	-0.0228	0.0035	-0.0006	0.0003
rs62241216	22	50744821	G	A	0.4925	-0.0124	0.0020	-0.0002	0.0002
rs713909	22	39532420	C	G	0.4328	-0.0286	0.0019	0.0000	0.0002
rs714027	22	30577771	G	A	0.5521	-0.0349	0.0019	-0.0004	0.0002
rs9306336	22	39873470	T	A	0.4898	0.0112	0.0020	0.0001	0.0002
