# Supplementary material for: Blood cell traits and risk of glaucoma: A two-sample mendelian randomization study
Source: Front Genet. 2023 Apr 12;14:1142773. doi: 10.3389/fgene.2023.1142773 (PMC10130872; doi:10.3389/fgene.2023.1142773)
Supplement: Supplementary file 1 [file DataSheet1.ZIP › eTable 2. Eosinophil cell count exposure SNPs and their association with glaucoma.pdf]

eTable 2. Eosinophil cell count exposure SNPs and their association with glaucoma.
Chr = chromosome; POS = position ; EA = effect allele; NEA = non-effect allele; EAF = effect allele frequency; SE = standard error.

SNP	Chr	POS	EA	NEA	EAF	Eosinophil Cell Count		Glaucoma	
						Beta	SE	Beta	SE
rs1004870	1	42370787	T	C	0.589	0.0262	0.002014	-0.0004	0.0002
rs1036332	1	199012478	C	A	0.7374	-0.0327	0.002257	0.0004	0.0002
rs10782957	1	93763896	T	C	0.6216	0.0225	0.002042	0.0001	0.0002
rs10916508	1	229659791	C	T	0.2298	-0.0163	0.002375	0.0003	0.0002
rs1099448	1	173231898	T	C	0.4664	0.0177	0.001998	0.0004	0.0002
rs111759324	1	101652522	T	C	0.1108	-0.043	0.00316	0.0002	0.0003
rs11204682	1	150595537	T	G	0.223	-0.0413	0.002388	0	0.0002
rs11264296	1	154994750	C	T	0.4206	0.0124	0.002019	0.0001	0.0002
rs113105190	1	28614481	C	T	0.0681	-0.0331	0.003942	-0.0002	0.0003
rs11578794	1	108401237	T	A	0.7281	-0.0135	0.002238	0.0001	0.0002
rs11583398	1	206968955	A	C	0.1409	-0.0204	0.002867	-0.0003	0.0002
rs1214598	1	167426424	A	G	0.3841	-0.0181	0.002037	-0.0001	0.0002
rs12408934	1	65423447	A	G	0.1026	-0.0413	0.00328	-0.0002	0.0003
rs12735509	1	198099838	T	C	0.083	0.0214	0.003584	0.0002	0.0003
rs12747584	1	101106044	T	C	0.0986	-0.0197	0.003314	0.0001	0.0003
rs1289273	1	226909233	G	A	0.5086	0.0159	0.002132	0	0.0002
rs1414517	1	221092838	G	C	0.1873	-0.025	0.002552	0.0002	0.0002
rs146078144	1	150889608	A	G	0.0096	0.0859	0.010438	0.0014	0.0009
rs1503854	1	160599010	G	A	0.3871	0.0149	0.002064	0.0001	0.0002
rs159963	1	8504421	A	C	0.582	0.0193	0.002017	0.0001	0.0002
rs17668272	1	198623842	T	G	0.1178	-0.0417	0.003072	-0.0004	0.0003
rs17849501	1	183542323	T	C	0.052	-0.0268	0.004469	-0.0001	0.0004
rs1848435	1	92043073	A	G	0.1684	0.0152	0.002666	0	0.0002
rs2502995	1	24199290	C	T	0.5705	0.0219	0.002002	0.0001	0.0002
rs2767327	1	117376689	G	A	0.894	-0.0186	0.003231	0	0.0003
rs3218148	1	23851787	A	G	0.5398	-0.0177	0.001991	-0.0004	0.0002
rs34448954	1	161168189	T	C	0.1064	-0.0253	0.003211	-0.0001	0.0003
rs34631302	1	28208327	G	A	0.6449	-0.0119	0.002072	-0.0002	0.0002
rs35249183	1	12099345	G	A	0.0998	0.04	0.003364	0	0.0003
rs41299003	1	206656165	G	A	0.2359	0.0146	0.002332	0.0004	0.0002
rs41313381	1	79411968	A	C	0.0307	0.0501	0.005645	-0.0001	0.0005
rs4149909	1	242023898	G	A	0.0326	-0.0334	0.005569	0.0002	0.0005
rs4310436	1	39489221	A	G	0.1196	0.0188	0.003065	0.0001	0.0003
rs4652560	1	181057188	T	A	0.6178	-0.0154	0.002044	-0.0002	0.0002
rs4653102	1	33830413	C	T	0.5466	-0.0163	0.001985	-0.0001	0.0002
rs4870	1	2488153	G	A	0.4816	-0.0144	0.001987	0.0002	0.0002
rs4908835	1	9357460	C	T	0.1614	0.0192	0.002719	-0.0002	0.0002
rs556063	1	111318291	G	A	0.5735	0.0128	0.002003	0.0001	0.0002
rs6540985	1	9708688	G	A	0.3193	-0.0196	0.002141	-0.0002	0.0002
rs6672031	1	172796505	G	C	0.4863	0.0201	0.002015	0.0001	0.0002
rs6684992	1	87752000	T	A	0.1195	0.0377	0.003052	-0.0001	0.0003
rs6691839	1	26559306	T	G	0.7655	-0.0132	0.002336	0.0002	0.0002
rs6696908	1	212907866	C	T	0.6482	0.0118	0.002071	0	0.0002
rs72736909	1	220999969	T	C	0.0663	0.025	0.004031	-0.0005	0.0003
rs77625297	1	65362766	C	G	0.0353	-0.0459	0.005607	-0.0001	0.0005
rs100461	2	38265978	C	T	0.5425	-0.0122	0.00198	0	0.0002
rs10165678	2	30446568	A	G	0.7575	-0.0147	0.002302	0.0002	0.0002
rs10174238	2	191973034	A	G	0.7656	0.0133	0.002402	0.0002	0.0002
rs10195713	2	158520905	T	C	0.8644	0.0249	0.002947	-0.0002	0.0002
rs1047891	2	211540507	A	C	0.3155	0.017	0.002127	0.0002	0.0002
rs1057258	2	234115629	T	C	0.1781	-0.0321	0.002589	0	0.0002
rs10930337	2	169695031	T	C	0.2848	0.0147	0.002192	-0.0003	0.0002
rs113542380	2	43464818	A	G	0.0751	-0.0269	0.003797	-0.0001	0.0003

rs11695281	2	28686568	T	C	0.5144	-0.023	0.00198	-0.0001	0.0002
rs11886928	2	74258529	G	T	0.3085	-0.0144	0.00214	0.0001	0.0002
rs11895277	2	72077960	T	C	0.8837	-0.0182	0.003081	0.0001	0.0003
rs11895564	2	173339808	A	G	0.3046	-0.0123	0.002148	0.0001	0.0002
rs12470046	2	28873870	C	T	0.3185	-0.0123	0.00212	0	0.0002
rs12472034	2	136828273	G	C	0.323	-0.0123	0.002123	0.0001	0.0002
rs1257192	2	134991024	G	A	0.8121	0.0146	0.002535	-0.0002	0.0002
rs13411829	2	106466101	C	G	0.3771	0.013	0.002045	0.0001	0.0002
rs1377454	2	148937370	T	G	0.4406	-0.0142	0.002	0.0002	0.0002
rs1427499	2	145400317	G	A	0.7103	0.0187	0.002183	-0.0003	0.0002
rs144569746	2	111908567	T	C	0.1022	0.059	0.003282	0.0002	0.0003
rs1448187	2	112220359	C	T	0.6999	0.0164	0.0024	0.0002	0.0002
rs1468445	2	242395554	G	A	0.1281	-0.0318	0.002958	-0.0001	0.0002
rs1519602	2	197027792	G	T	0.6478	0.0127	0.002069	0.0001	0.0002
rs17682575	2	43152304	T	C	0.2013	-0.0176	0.002487	0.0002	0.0002
rs17696274	2	102963227	G	C	0.0375	0.0478	0.005221	-0.0004	0.0004
rs2160783	2	71283467	G	A	0.3408	-0.0159	0.002089	0	0.0002
rs2579506	2	97207321	A	G	0.3862	0.0475	0.002032	-0.0001	0.0002
rs2713548	2	227156662	T	C	0.6326	0.0125	0.002046	-0.0001	0.0002
rs34290285	2	242698640	A	G	0.2571	-0.0459	0.002267	0.0001	0.0002
rs346835	2	8438693	T	C	0.3283	-0.0267	0.002104	-0.0004	0.0002
rs35409523	2	213908457	A	G	0.0759	0.0501	0.003754	0.0002	0.0003
rs4675190	2	228320192	T	C	0.5911	-0.0117	0.002017	-0.0001	0.0002
rs4849903	2	112376294	T	C	0.6331	0.0211	0.002058	0.0003	0.0002
rs55686954	2	204586515	A	G	0.0446	-0.0288	0.004819	0	0.0004
rs62105489	2	8757326	T	C	0.0529	-0.0302	0.004452	-0.0009	0.0004
rs6731125	2	182308836	C	T	0.5648	0.0172	0.002002	0.0003	0.0002
rs6750754	2	213830187	G	T	0.2642	-0.064	0.002243	0	0.0002
rs699664	2	85780536	T	C	0.3296	-0.0157	0.002102	-0.0001	0.0002
rs72998585	2	102858490	T	A	0.1345	-0.0908	0.002922	0	0.0002
rs7423615	2	231116874	T	C	0.1873	0.0232	0.002529	0.0003	0.0002
rs75535961	2	204603769	A	G	0.0373	-0.0472	0.005222	0.0003	0.0004
rs7561544	2	46076545	C	T	0.7588	0.0127	0.002312	0.0001	0.0002
rs7569084	2	65656969	T	C	0.5843	0.0187	0.002006	0	0.0002
rs7590808	2	238859223	G	C	0.371	-0.012	0.002065	0	0.0002
rs75960776	2	242804707	C	T	0.1614	0.017	0.002713	0.0001	0.0002
rs7602992	2	54795929	G	A	0.1978	0.0155	0.002481	-0.0002	0.0002
rs76908370	2	163207971	G	A	0.0363	-0.0323	0.005559	-0.0003	0.0005
rs778756	2	61781994	G	A	0.5797	-0.016	0.002001	0.0002	0.0002
rs78694312	2	103080819	C	T	0.0213	0.0491	0.006915	-0.0006	0.0006
rs79716587	2	143886819	A	G	0.1265	-0.0245	0.003003	-0.0004	0.0002
rs80066203	2	26220710	T	C	0.0679	-0.0241	0.003931	-0.0004	0.0003
rs925966	2	136806959	G	C	0.7595	-0.0183	0.002373	0.0004	0.0002
rs112255429	3	127786822	A	G	0.0166	-0.0462	0.007975	0.0001	0.0007
rs1131199	3	112059768	G	C	0.5295	-0.0247	0.001979	0.0001	0.0002
rs11465898	3	10255873	G	A	0.054	0.0413	0.004405	0.0005	0.0004
rs12487980	3	71760041	A	C	0.6338	0.0125	0.002073	-0.0003	0.0002
rs12629111	3	40285351	C	T	0.388	-0.0116	0.002031	0	0.0002
rs13064662	3	18687199	A	G	0.1326	-0.0161	0.002914	-0.0003	0.0002
rs1353286	3	27772014	G	T	0.453	0.0202	0.00199	0	0.0002
rs1516527	3	148609704	C	T	0.9511	-0.0323	0.004596	0.0005	0.0004
rs1546079	3	46150119	T	C	0.5114	-0.0112	0.002002	0.0002	0.0002
rs1672753	3	3221430	T	C	0.811	-0.0175	0.002545	0.0001	0.0002
rs191413135	3	49745681	A	G	0.0122	0.0518	0.009293	-0.0007	0.0008
rs2089979	3	196501413	G	A	0.4159	-0.0149	0.002013	0	0.0002
rs2228467	3	42906116	C	T	0.0616	0.0623	0.004111	0.0001	0.0003
rs2399441	3	112587012	C	T	0.3506	-0.0189	0.002069	0.0003	0.0002
rs2920505	3	12335081	A	G	0.6018	-0.0296	0.002019	-0.0003	0.0002

rs2942062	3	112305130	G	A	0.9374	-0.0227	0.004079	-0.0004	0.0003
rs322692	3	25348620	A	G	0.3451	0.0161	0.002091	-0.0001	0.0002
rs3804590	3	121978073	T	G	0.3172	0.018	0.002139	-0.0004	0.0002
rs3950296	3	169493283	G	C	0.2444	0.0236	0.002297	-0.0003	0.0002
rs4074672	3	183730295	T	C	0.3693	0.0145	0.00205	-0.0001	0.0002
rs4618204	3	101281534	C	T	0.4438	0.0182	0.002001	0.0001	0.0002
rs4680250	3	150941492	G	C	0.693	-0.0157	0.002175	0.0004	0.0002
rs4686811	3	186617522	C	G	0.5686	-0.0113	0.002018	0.0002	0.0002
rs57584583	3	105917638	G	A	0.2581	0.0124	0.00226	0.0003	0.0002
rs60123882	3	51689306	G	A	0.2061	-0.0273	0.002542	0.0003	0.0002
rs6769504	3	16909336	G	A	0.1495	-0.0193	0.00277	-0.0001	0.0002
rs6777420	3	142002869	T	C	0.1316	-0.02	0.002936	0.0001	0.0002
rs6787336	3	3153194	A	G	0.2875	0.0363	0.002238	0	0.0002
rs6793191	3	3142503	A	C	0.478	-0.012	0.001996	0.0002	0.0002
rs73176183	3	171503934	C	T	0.1257	-0.0196	0.002988	0.0004	0.0002
rs73203442	3	128265298	T	C	0.1222	-0.0957	0.003032	0.0003	0.0003
rs7636495	3	196367936	A	G	0.1808	-0.024	0.002591	0	0.0002
rs7646283	3	33046480	T	C	0.3677	0.026	0.002063	-0.0003	0.0002
rs7646695	3	72172181	T	C	0.1797	0.0176	0.002617	-0.0002	0.0002
rs7649812	3	98449191	G	C	0.2428	0.016	0.002304	0.0001	0.0002
rs76830965	3	159637678	A	C	0.1176	-0.0234	0.003083	-0.0001	0.0003
rs789862	3	194404522	G	A	0.4033	-0.0146	0.002018	0.0002	0.0002
rs79171715	3	128776664	C	A	0.0257	-0.0526	0.006317	-0.0001	0.0005
rs9815874	3	188441161	T	C	0.2995	0.0284	0.002157	0	0.0002
rs9835307	3	48857805	C	T	0.6583	0.0263	0.002187	-0.0002	0.0002
rs9837045	3	3069773	A	G	0.4554	0.0113	0.001984	0	0.0002
rs9838419	3	141215409	G	C	0.3271	0.0127	0.002123	-0.0001	0.0002
rs9840310	3	71452860	G	A	0.7346	-0.0127	0.002239	0	0.0002
rs9880192	3	128297569	C	G	0.4113	0.0421	0.002026	-0.0005	0.0002
rs113473633	4	103449131	G	A	0.0262	-0.0632	0.006522	0	0.0005
rs11931711	4	153291513	T	C	0.2824	0.0193	0.002213	0.0003	0.0002
rs13105682	4	102702364	G	T	0.0597	-0.0266	0.00433	0.0004	0.0004
rs13120371	4	139092719	G	A	0.3272	0.0155	0.002118	-0.0001	0.0002
rs13138355	4	83545976	T	C	0.1855	-0.0548	0.002551	0	0.0002
rs13139941	4	7071741	G	A	0.8029	-0.0138	0.002491	-0.0004	0.0002
rs1365623	4	175486022	T	C	0.3725	0.0116	0.00206	-0.0001	0.0002
rs1479918	4	123351431	T	A	0.2598	-0.0188	0.002261	0	0.0002
rs1828803	4	2689449	A	C	0.3924	0.0131	0.002033	-0.0001	0.0002
rs2566133	4	39022515	C	T	0.5836	-0.0144	0.002015	-0.0001	0.0002
rs62308111	4	57738491	T	G	0.2215	0.0154	0.0024	-0.0001	0.0002
rs73232881	4	38664131	C	T	0.2132	0.0674	0.002418	0.0002	0.0002
rs7441808	4	26090375	G	A	0.3012	0.0139	0.002161	0.0002	0.0002
rs746550	4	123619689	T	C	0.2359	0.023	0.002342	-0.0002	0.0002
rs7687708	4	6906076	G	T	0.22	-0.0147	0.002434	0.0005	0.0002
rs76981581	4	38416046	G	C	0.0479	-0.0261	0.004658	0.0002	0.0004
rs10059018	5	110501604	T	G	0.2008	-0.0234	0.00247	-0.0001	0.0002
rs10062687	5	10624866	G	T	0.2329	0.0241	0.002349	-0.0001	0.0002
rs10472984	5	35843832	G	C	0.3399	-0.035	0.00209	0.0001	0.0002
rs13158276	5	156664205	G	A	0.262	0.0138	0.002255	0.0002	0.0002
rs139640694	5	109938754	G	A	0.0929	0.0249	0.003415	-0.0004	0.0003
rs16903574	5	14610309	G	C	0.0778	0.0285	0.003838	0.0002	0.0003
rs17516457	5	131590387	C	T	0.417	-0.0639	0.002008	-0.0004	0.0002
rs2237060	5	131970885	G	T	0.4357	-0.0181	0.002039	-0.0002	0.0002
rs2568928	5	127487603	G	A	0.7568	0.0152	0.002308	0	0.0002
rs2961914	5	159868958	A	C	0.2004	-0.019	0.002474	0.0002	0.0002
rs34495	5	98265807	T	G	0.3042	-0.0198	0.002155	-0.0001	0.0002
rs36115365	5	1313242	C	G	0.2055	0.0165	0.002463	0.0002	0.0002
rs4703589	5	72097351	C	T	0.5327	0.0145	0.001983	-0.0002	0.0002

rs4703730	5	76549688	T	C	0.517	-0.016	0.001984	-0.0001	0.0002
rs56330463	5	148200011	C	T	0.5533	0.0395	0.002008	-0.0003	0.0002
rs5742913	5	133451683	A	C	0.1112	0.0268	0.003157	-0.0003	0.0003
rs60905948	5	142459812	T	G	0.7091	0.0162	0.002291	0.0001	0.0002
rs62385501	5	171950231	A	T	0.3081	-0.0144	0.002142	-0.0003	0.0002
rs6556313	5	176792491	G	A	0.3323	0.0195	0.002102	0.0003	0.0002
rs6874023	5	86383968	A	T	0.7569	0.0139	0.002306	0.0003	0.0002
rs73272842	5	150453888	A	G	0.1228	-0.0254	0.003019	-0.0003	0.0003
rs7700687	5	141491985	T	C	0.6172	0.04	0.002035	-0.0005	0.0002
rs79881201	5	110427795	T	C	0.3598	0.0403	0.002063	0.0002	0.0002
rs9313497	5	169691627	C	T	0.0678	-0.0303	0.003971	0	0.0003
rs113496608	6	138161666	A	G	0.0303	-0.0448	0.005802	0.0005	0.0005
rs12208103	6	107442431	T	C	0.3784	-0.0307	0.002069	-0.0001	0.0002
rs1569699	6	20679310	G	T	0.3099	0.0125	0.002142	0	0.0002
rs1624064	6	26378681	C	T	0.4206	0.0152	0.002018	0	0.0002
rs1840169	6	52296461	C	G	0.4567	-0.0121	0.001986	0.0001	0.0002
rs2025489	6	87814959	G	A	0.526	0.0158	0.00199	0.0004	0.0002
rs2356817	6	109642675	A	G	0.4788	0.0143	0.002001	0.0002	0.0002
rs2788211	6	697500	C	T	0.8173	0.016	0.002573	0.0001	0.0002
rs28383314	6	32587213	C	T	0.6241	0.0616	0.002048	0.0002	0.0002
rs28449420	6	33045530	C	T	0.0384	-0.0335	0.005197	-0.0004	0.0004
rs2894401	6	35408959	G	A	0.7053	-0.0205	0.002187	-0.0001	0.0002
rs3093023	6	167534290	A	G	0.4338	0.0127	0.001998	0.0002	0.0002
rs3757114	6	559576	C	A	0.4742	-0.0252	0.001987	0.0001	0.0002
rs3846855	6	33555877	A	G	0.1829	0.0282	0.002567	-0.0003	0.0002
rs391875	6	149795136	A	G	0.4038	0.0131	0.002021	0.0001	0.0002
rs547211157	6	130341903	C	G	0.014	0.0499	0.008857	0.001	0.0007
rs61612642	6	42197707	T	C	0.165	0.0375	0.002694	0	0.0002
rs62395833	6	31807652	C	G	0.0431	0.0368	0.004882	-0.0002	0.0004
rs62408224	6	90955995	G	A	0.3504	-0.0422	0.002073	0	0.0002
rs62420764	6	106799970	C	T	0.1404	0.019	0.002874	-0.0006	0.0002
rs6904506	6	245096	C	T	0.0885	-0.0524	0.003544	-0.0004	0.0003
rs6924350	6	31343632	C	A	0.1845	0.05	0.002537	-0.0001	0.0002
rs6924387	6	137082948	G	A	0.4102	0.0167	0.002027	0	0.0002
rs6930635	6	395634	C	T	0.0785	-0.0377	0.003739	0.0002	0.0003
rs72992130	6	144441671	T	C	0.0456	0.0346	0.004829	-0.0001	0.0004
rs73428834	6	42255274	T	C	0.0748	0.0298	0.003774	-0.0004	0.0003
rs7382061	6	30047965	C	T	0.5907	-0.0546	0.002014	-0.0005	0.0002
rs7759516	6	151838245	C	T	0.1822	-0.0161	0.002572	-0.0002	0.0002
rs783646	6	117286220	C	G	0.8167	-0.0188	0.002566	0	0.0002
rs79701703	6	135178202	T	C	0.0307	0.041	0.00575	0.0002	0.0005
rs9389268	6	135419631	G	A	0.2561	-0.0454	0.002292	0.0001	0.0002
rs9390030	6	143224510	T	C	0.0945	0.0248	0.003397	0.0001	0.0003
rs9392525	6	435677	C	T	0.5755	0.0132	0.002024	0.0003	0.0002
rs9395112	6	45682806	G	A	0.1559	0.0199	0.002742	0.0002	0.0002
rs9654603	6	150370053	A	G	0.1595	-0.0159	0.002712	0.0001	0.0002
rs10230696	7	106432360	T	A	0.825	0.0149	0.002634	0.0003	0.0002
rs1037674	7	50217850	T	G	0.2923	0.0132	0.002188	-0.0003	0.0002
rs111246112	7	3055099	T	A	0.0594	-0.0258	0.004222	-0.0002	0.0003
rs12154498	7	92223518	C	A	0.8544	-0.0325	0.002817	0	0.0002
rs12530946	7	148887942	G	A	0.6133	0.0428	0.002044	0.0003	0.0002
rs12531540	7	28162674	T	C	0.4952	-0.0122	0.001982	0.0004	0.0002
rs12540285	7	150312474	G	A	0.2206	0.0146	0.002396	-0.0002	0.0002
rs12705849	7	112782556	A	G	0.4069	-0.0206	0.002021	0.0001	0.0002
rs13226583	7	75454152	T	A	0.1157	-0.0619	0.0031	-0.0003	0.0003
rs150640087	7	50444152	T	G	0.0162	0.0995	0.008004	-0.0005	0.0007
rs1880345	7	101372755	A	G	0.266	0.0135	0.002248	-0.0002	0.0002
rs3757569	7	44889508	A	G	0.4335	-0.012	0.001999	-0.0002	0.0002

rs3823536	7	128579666	A	G	0.4672	-0.0178	0.001986	0.0002	0.0002
rs4721559	7	17007940	T	C	0.2781	-0.0174	0.002234	-0.0002	0.0002
rs4722171	7	22785717	G	A	0.5921	-0.0273	0.002019	0	0.0002
rs55879743	7	75470364	T	C	0.0664	0.0777	0.004051	-0.0002	0.0003
rs56179563	7	129685597	A	G	0.3891	0.0179	0.002055	-0.0002	0.0002
rs56195338	7	8107922	A	G	0.0577	-0.0301	0.004266	0	0.0004
rs57834782	7	20502828	A	T	0.2448	-0.0583	0.002306	-0.0001	0.0002
rs60600003	7	37382465	G	T	0.1003	0.0429	0.003317	0	0.0003
rs62473720	7	77254547	G	A	0.3171	0.0142	0.002136	0	0.0002
rs6956283	7	98756597	T	C	0.7328	-0.0157	0.002237	0	0.0002
rs6959832	7	135289854	A	G	0.5069	-0.0162	0.001983	0	0.0002
rs6971710	7	3140173	A	G	0.201	0.0283	0.002475	0.0001	0.0002
rs6979947	7	157005863	G	A	0.2664	0.0132	0.002242	0.0001	0.0002
rs73069541	7	26904928	G	A	0.2338	-0.0154	0.00234	-0.0001	0.0002
rs73118830	7	50764948	C	T	0.0855	-0.0294	0.003538	0	0.0003
rs73187850	7	101867824	T	A	0.2783	0.0185	0.002218	0.0003	0.0002
rs7778729	7	45089726	C	T	0.2755	0.0185	0.002219	-0.0002	0.0002
rs7789337	7	138788795	C	G	0.2179	0.0185	0.002402	-0.0002	0.0002
rs7797428	7	124886883	T	C	0.4692	0.0124	0.001999	-0.0001	0.0002
rs8	7	92408329	T	C	0.2043	0.0219	0.002545	0	0.0002
rs1000264	8	134110278	G	A	0.4802	0.0121	0.001986	0	0.0002
rs10100356	8	130626164	A	G	0.2272	-0.0134	0.002387	-0.0003	0.0002
rs117182261	8	43508916	A	G	0.0264	-0.0496	0.006701	-0.0002	0.0005
rs11786536	8	129000416	A	G	0.1649	-0.0267	0.002705	-0.0001	0.0002
rs117961539	8	144989466	A	G	0.0396	-0.031	0.0052	-0.0003	0.0004
rs12545733	8	22957003	C	T	0.7034	-0.0307	0.002188	-0.0001	0.0002
rs12681644	8	119972974	T	C	0.2119	-0.0194	0.002427	-0.0004	0.0002
rs13251643	8	126240768	T	A	0.0444	0.0304	0.004844	0.001	0.0004
rs28846070	8	43636212	G	T	0.9746	0.0346	0.006327	-0.0007	0.0005
rs2887502	8	79756591	T	C	0.5902	-0.0136	0.002024	-0.0001	0.0002
rs295	8	19816238	C	A	0.2351	0.0159	0.002337	0.0001	0.0002
rs2979489	8	30280833	A	G	0.7417	0.0138	0.002266	0	0.0002
rs34173062	8	145158607	A	G	0.073	0.0546	0.004091	-0.0003	0.0003
rs3829054	8	61766431	T	C	0.6779	-0.0141	0.002124	-0.0001	0.0002
rs4236746	8	130699861	G	A	0.9751	0.0569	0.006399	0.0005	0.0005
rs4240624	8	9184231	A	G	0.909	-0.0204	0.003453	-0.0001	0.0003
rs45577137	8	48651633	G	A	0.0452	-0.0598	0.005157	-0.0002	0.0004
rs4870977	8	127526842	C	G	0.8709	-0.0188	0.002958	0	0.0002
rs574183	8	95973816	G	A	0.389	-0.0138	0.002036	-0.0003	0.0002
rs62539154	8	48677930	A	G	0.0973	0.0275	0.003402	-0.0001	0.0003
rs6986109	8	81286298	T	G	0.7043	-0.0165	0.002181	-0.0004	0.0002
rs6989099	8	66898262	C	T	0.3173	-0.0178	0.002133	-0.0002	0.0002
rs6999452	8	106397116	A	G	0.5177	0.0116	0.001991	0	0.0002
rs7839946	8	22901943	C	T	0.057	0.0243	0.004327	-0.0001	0.0004
rs7840212	8	130599247	T	C	0.337	-0.0402	0.00211	0.0001	0.0002
rs7846314	8	61650831	T	A	0.187	-0.0329	0.002543	-0.0001	0.0002
rs9969668	8	135731578	A	G	0.1546	-0.0185	0.002756	-0.0003	0.0002
rs114741563	9	135869211	C	T	0.0075	0.1196	0.011508	0.0009	0.001
rs143491704	9	135345666	G	C	0.0358	-0.0313	0.005517	0.0001	0.0005
rs1547258	9	6523056	C	T	0.7077	-0.0195	0.002215	-0.0001	0.0002
rs295273	9	86466968	A	G	0.2556	0.0206	0.002274	-0.0002	0.0002
rs33982662	9	139324737	A	C	0.2853	0.0204	0.002192	0.0003	0.0002
rs3731211	9	21986847	A	T	0.7203	0.0175	0.002213	-0.0002	0.0002
rs4142528	9	6172296	A	T	0.6722	-0.0749	0.00212	0.0003	0.0002
rs460631	9	4851440	G	A	0.8849	0.0257	0.003115	0	0.0003
rs4644350	9	16882916	G	C	0.6238	-0.0151	0.002058	0	0.0002
rs4743150	9	100740124	T	C	0.2197	0.0144	0.002391	0.0003	0.0002
rs496475	9	113638236	G	T	0.387	0.035	0.002033	0	0.0002

rs6479336	9	94110288	A	T	0.185	-0.0292	0.002556	0.0001	0.0002
rs7026022	9	102562216	C	A	0.3778	0.0121	0.002045	0	0.0002
rs72766638	9	136931778	A	C	0.1643	-0.0243	0.002673	0.0003	0.0002
rs74612091	9	135877278	A	T	0.0631	0.061	0.004113	0.0001	0.0003
rs7868130	9	4998401	T	C	0.2588	0.0324	0.002269	0	0.0002
rs884634	9	34861298	C	T	0.415	0.0124	0.002012	0.0001	0.0002
rs911603	9	117697584	A	C	0.4036	-0.0247	0.002043	0.0005	0.0002
rs10995240	10	64388631	C	G	0.3683	-0.0438	0.002049	-0.0001	0.0002
rs11010084	10	35360620	G	A	0.4031	0.0158	0.002032	-0.0001	0.0002
rs11255507	10	8109615	G	T	0.1782	0.0202	0.00261	-0.0001	0.0002
rs1250567	10	81046265	C	T	0.4514	-0.011	0.002002	-0.0001	0.0002
rs12762934	10	104359884	T	C	0.3225	0.0147	0.002119	0.0001	0.0002
rs1539174	10	974870	G	C	0.2374	0.038	0.002332	-0.0001	0.0002
rs16918741	10	65410353	C	G	0.0266	0.0345	0.006235	0.0001	0.0005
rs17482472	10	44859618	A	G	0.0989	-0.0315	0.003346	-0.0001	0.0003
rs2419313	10	111750851	A	G	0.1425	-0.0207	0.002869	0.0004	0.0002
rs2497318	10	94432000	T	C	0.4426	-0.0299	0.00202	0.0001	0.0002
rs2505521	10	43804617	T	C	0.8292	-0.0152	0.002675	-0.0002	0.0002
rs2807740	10	28784483	T	C	0.7697	0.0353	0.002358	-0.0001	0.0002
rs290897	10	8932980	T	C	0.9725	0.0437	0.006135	0.001	0.0005
rs2992333	10	26727454	A	G	0.5987	-0.0342	0.002028	0.0002	0.0002
rs3747869	10	73520632	C	A	0.9008	0.0198	0.003316	-0.0003	0.0003
rs4268448	10	99105320	A	G	0.2552	0.0139	0.002275	0.0001	0.0002
rs4746153	10	75598282	C	G	0.1854	0.0157	0.002552	0	0.0002
rs495149	10	89795523	T	C	0.1642	0.0243	0.002674	0	0.0002
rs7072793	10	6106266	C	T	0.4119	0.0176	0.002018	0.0003	0.0002
rs7078237	10	70774039	T	A	0.2937	0.0123	0.00219	-0.0001	0.0002
rs7080536	10	115348046	A	G	0.0432	-0.0437	0.004952	-0.0003	0.0004
rs7098294	10	8575248	C	T	0.4685	-0.0228	0.001986	0.0001	0.0002
rs71508968	10	65253869	A	G	0.0258	0.0362	0.006364	0.0005	0.0005
rs72834751	10	64559465	T	C	0.0127	-0.0717	0.009325	0	0.0008
rs7897422	10	90849704	C	T	0.1921	0.0208	0.002521	0	0.0002
rs962993	10	9053132	T	C	0.4225	-0.0404	0.002009	0.0004	0.0002
rs1059091	11	309127	G	A	0.3213	0.0338	0.002141	-0.0001	0.0002
rs10893844	11	128185850	C	G	0.5016	0.0246	0.001984	0.0003	0.0002
rs11236813	11	76343427	C	G	0.1019	-0.0303	0.003306	0.0002	0.0003
rs12970	11	117074109	A	G	0.0604	0.0341	0.004168	0	0.0003
rs171270	11	126207745	G	C	0.2872	0.0191	0.002244	0.0003	0.0002
rs174548	11	61571348	G	C	0.3136	-0.0231	0.002137	0.0001	0.0002
rs214080	11	17299762	G	A	0.5799	0.0129	0.002009	0.0001	0.0002
rs2241899	11	57176628	T	C	0.2698	0.0234	0.002243	-0.0001	0.0002
rs34439695	11	33901483	T	C	0.0353	-0.0379	0.005456	-0.0005	0.0005
rs360124	11	9802228	C	G	0.904	-0.0228	0.003372	0	0.0003
rs3824867	11	47468569	G	A	0.7124	0.0168	0.002193	-0.0002	0.0002
rs4409785	11	95311422	C	T	0.1723	0.0164	0.00263	-0.0001	0.0002
rs473739	11	64147919	G	T	0.3189	-0.0139	0.002128	0.0002	0.0002
rs58833930	11	2325997	T	C	0.113	-0.0234	0.003139	-0.0003	0.0003
rs634534	11	65665256	G	A	0.5425	0.0329	0.001998	0.0001	0.0002
rs637064	11	108140909	T	C	0.5561	0.0243	0.001994	-0.0002	0.0002
rs668248	11	128586262	C	G	0.6155	-0.0165	0.002036	-0.0001	0.0002
rs7123726	11	118694547	C	T	0.2105	0.0169	0.002436	-0.0002	0.0002
rs72844043	11	3098933	A	G	0.4318	-0.0114	0.002011	0.0002	0.0002
rs7936434	11	76293805	C	G	0.4777	0.043	0.001984	0	0.0002
rs7939912	11	69982096	A	G	0.6049	0.0153	0.002039	0	0.0002
rs964184	11	116648917	C	G	0.8666	0.0288	0.002913	0.0002	0.0002
rs9666598	11	325386	G	C	0.8429	-0.0197	0.002778	0	0.0002
rs10745763	12	96811386	T	G	0.4233	0.0135	0.002018	0.0001	0.0002
rs10772567	12	12592995	A	G	0.4855	0.0109	0.001982	0.0001	0.0002

rs10777164	12	89831594	G	A	0.7138	0.0135	0.002195	0.0002	0.0002
rs10777378	12	92518680	A	G	0.5357	-0.0225	0.001989	0.0001	0.0002
rs10876550	12	54712308	A	G	0.5592	0.0138	0.001998	0	0.0002
rs11065822	12	111600134	T	G	0.3544	0.065	0.002136	-0.0001	0.0002
rs115647629	12	111811903	A	G	0.0265	-0.0673	0.006524	0.0001	0.0005
rs11571404	12	1041450	T	C	0.2034	0.0154	0.002485	-0.0002	0.0002
rs12581511	12	46573788	G	C	0.173	-0.0176	0.002631	-0.0005	0.0002
rs12820863	12	4318723	T	C	0.3516	0.0225	0.002142	0.0001	0.0002
rs146730870	12	112332996	A	G	0.0103	-0.0867	0.010334	0.0005	0.0009
rs1471816	12	94145120	A	G	0.5033	0.0109	0.001985	0	0.0002
rs1689510	12	56396768	C	G	0.3377	0.0265	0.002094	0	0.0002
rs1800692	12	6442346	G	A	0.5875	-0.0181	0.002039	0.0001	0.0002
rs1861489	12	68610446	A	T	0.7822	0.0133	0.00242	0	0.0002
rs2228570	12	48272895	G	A	0.6148	0.0122	0.002043	0.0001	0.0002
rs28532037	12	123883406	A	G	0.9065	-0.0297	0.003416	-0.0003	0.0003
rs3024971	12	57493727	G	T	0.1072	-0.0402	0.003209	0.0004	0.0003
rs3759332	12	6491078	C	T	0.3906	-0.0119	0.002064	0.0001	0.0002
rs4931002	12	32143169	A	C	0.7793	-0.0216	0.002388	0	0.0002
rs6490291	12	112177775	A	T	0.9641	-0.0661	0.005837	0.0002	0.0005
rs708785	12	121228791	A	G	0.5858	-0.0188	0.002006	0	0.0002
rs7299446	12	70611429	A	T	0.5243	-0.0115	0.001981	0.0002	0.0002
rs73135231	12	62647835	G	T	0.1074	-0.0192	0.003211	-0.0006	0.0003
rs73202462	12	110266430	A	G	0.02	0.0568	0.007727	-0.0003	0.0006
rs78691875	12	113070264	A	C	0.0211	-0.0511	0.006932	-0.0009	0.0006
rs7979810	12	47085520	A	C	0.563	-0.0116	0.002002	0.0001	0.0002
rs12100034	13	114956773	A	G	0.3591	-0.017	0.002079	0.0001	0.0002
rs12861824	13	31079184	C	T	0.2754	-0.021	0.002402	0	0.0002
rs17061503	13	41246758	A	G	0.3074	0.0355	0.002159	-0.0001	0.0002
rs201798	13	50954721	A	G	0.6149	0.0202	0.002044	-0.0003	0.0002
rs2182885	13	99855124	A	G	0.6052	0.0263	0.00203	0.0001	0.0002
rs275945	13	109799451	G	A	0.3489	0.0137	0.002083	-0.0001	0.0002
rs34363176	13	100270177	G	C	0.2436	0.0134	0.002326	0.0002	0.0002
rs71429414	13	42945821	A	G	0.1962	-0.0186	0.002496	-0.0003	0.0002
rs7327960	13	74682892	C	T	0.8266	0.0177	0.002629	0.0003	0.0002
rs77929895	13	50072429	C	T	0.0895	-0.0199	0.003489	0.0003	0.0003
rs7986796	13	40351064	T	G	0.6277	0.0243	0.002058	-0.0002	0.0002
rs11159261	14	77921120	C	T	0.5346	-0.0121	0.001992	0	0.0002
rs113859409	14	94422198	A	G	0.0397	-0.0293	0.00537	-0.0002	0.0004
rs11555542	14	94417531	C	T	0.0627	0.0638	0.004108	-0.0004	0.0003
rs11626787	14	103991749	G	A	0.3436	-0.0116	0.002096	0.0004	0.0002
rs117068593	14	93118229	T	C	0.1901	-0.0261	0.002543	-0.0001	0.0002
rs1274955	14	69289312	C	A	0.7796	-0.0189	0.002401	-0.0001	0.0002
rs12882281	14	21431304	C	T	0.2386	-0.0136	0.00235	0.0001	0.0002
rs175705	14	75975648	G	C	0.7185	0.0382	0.002213	-0.0001	0.0002
rs2239633	14	23589057	A	G	0.4835	0.0348	0.001986	0	0.0002
rs35641442	14	75207263	A	G	0.4624	-0.0111	0.00199	0.0003	0.0002
rs3742704	14	88477882	C	A	0.0916	0.0195	0.003441	0.0004	0.0003
rs3742833	14	73349103	A	G	0.7521	0.0161	0.002313	-0.0001	0.0002
rs6573020	14	55871452	T	C	0.4334	0.0206	0.002019	0.0002	0.0002
rs67856193	14	93024616	G	C	0.3084	0.0237	0.002175	0	0.0002
rs7141943	14	25438250	G	A	0.3947	0.0159	0.002038	0	0.0002
rs7158239	14	65520616	A	G	0.3952	0.0156	0.002037	0	0.0002
rs8012643	14	37692864	T	C	0.2864	0.0155	0.002194	-0.0001	0.0002
rs8020739	14	35882492	T	G	0.6438	0.0247	0.002087	0.0002	0.0002
rs941616	14	52621945	T	C	0.6096	-0.0118	0.002034	-0.0002	0.0002
rs942017	14	103018727	A	G	0.3484	-0.0173	0.002113	0.0002	0.0002
rs11071528	15	60701797	C	G	0.8071	0.0143	0.002523	0.0004	0.0002
rs11071559	15	61069988	T	C	0.1305	-0.0222	0.002945	-0.0003	0.0002

rs11637005	15	70381300	G	C	0.0696	0.0363	0.003951	0	0.0003
rs11855724	15	70915774	A	G	0.1814	-0.0144	0.002584	0.0001	0.0002
rs12913266	15	86294109	A	G	0.3909	-0.0179	0.002036	0	0.0002
rs1407588	15	81052947	A	G	0.4301	-0.0113	0.002009	-0.0001	0.0002
rs17293632	15	67442596	T	C	0.2363	0.0296	0.002337	0.0003	0.0002
rs34212866	15	43702964	G	A	0.2207	0.0201	0.002401	-0.0001	0.0002
rs35026629	15	70732013	G	A	0.4176	-0.0148	0.002036	0.0002	0.0002
rs62006172	15	38904527	A	G	0.042	-0.0375	0.004973	0.0005	0.0004
rs62011287	15	63791228	G	A	0.3443	-0.0127	0.002091	0.0001	0.0002
rs6496715	15	91177563	C	T	0.6124	0.0152	0.002083	0.0001	0.0002
rs7161799	15	58770523	T	C	0.0755	0.0246	0.003791	-0.0004	0.0003
rs7173571	15	41712807	C	T	0.5266	0.014	0.001993	0.0001	0.0002
rs7257	15	80191343	A	G	0.5672	0.0336	0.002002	-0.0001	0.0002
rs74781061	15	74888196	G	A	0.15	0.0159	0.002786	0.0001	0.0002
rs76246800	15	67364356	G	A	0.0286	-0.0333	0.005976	0.0002	0.0005
rs8026803	15	80260014	C	T	0.2609	-0.0313	0.002259	0.0001	0.0002
rs1039341	16	48574869	T	C	0.3109	-0.0239	0.002145	-0.0002	0.0002
rs11077339	16	3516888	C	T	0.0457	0.0297	0.004765	-0.0002	0.0004
rs11642836	16	79753625	A	G	0.3768	-0.0149	0.002052	-0.0001	0.0002
rs1170439	16	68608511	C	T	0.7793	0.0225	0.002403	0.0003	0.0002
rs12935457	16	85599399	G	T	0.5049	-0.0117	0.002047	-0.0001	0.0002
rs13338688	16	67248831	A	G	0.0623	0.0277	0.004106	0.0003	0.0003
rs17175830	16	88558164	A	G	0.2377	0.0322	0.002365	0.0001	0.0002
rs2058811	16	4575162	T	C	0.6905	-0.0127	0.002146	0	0.0002
rs2106450	16	20915247	C	T	0.7426	0.0178	0.002265	0.0003	0.0002
rs2161647	16	57503330	A	C	0.0373	-0.0404	0.005242	-0.0003	0.0004
rs301162	16	85810647	G	A	0.853	0.0261	0.002803	0.0003	0.0002
rs3743553	16	58030053	C	T	0.7706	-0.0145	0.002359	0.0002	0.0002
rs3785356	16	27349168	T	C	0.298	0.0302	0.00218	0.0001	0.0002
rs414723	16	79228250	T	C	0.8555	0.0176	0.00282	0	0.0002
rs4280242	16	2830482	T	C	0.7541	-0.0329	0.002318	-0.0001	0.0002
rs4476175	16	51234174	A	G	0.4595	0.0115	0.001996	0.0001	0.0002
rs61426394	16	9053519	C	G	0.0673	-0.0246	0.003983	0	0.0003
rs6498125	16	10996332	G	C	0.6045	0.0122	0.002061	0.0002	0.0002
rs7188272	16	79651979	G	A	0.7051	-0.0172	0.002178	0.0001	0.0002
rs7192652	16	57075180	G	A	0.4792	-0.0193	0.001996	-0.0001	0.0002
rs725613	16	11169683	G	T	0.356	-0.0474	0.002071	-0.0001	0.0002
rs74331768	16	2161793	A	G	0.0981	0.0195	0.003368	0.0003	0.0003
rs766814	16	11279159	A	G	0.3005	0.026	0.002189	-0.0002	0.0002
rs8061729	16	31283576	C	T	0.2739	-0.0131	0.002288	0	0.0002
rs9939774	16	30068354	T	C	0.4045	-0.0292	0.00202	0.0001	0.0002
rs11077961	17	81012749	G	A	0.3624	0.0137	0.002073	-0.0001	0.0002
rs112036266	17	2834143	T	C	0.1762	0.0168	0.002626	0	0.0002
rs11649845	17	76254193	C	T	0.3015	-0.0187	0.002186	0.0001	0.0002
rs12941068	17	17737857	A	G	0.2859	-0.0139	0.002272	0.0001	0.0002
rs13313564	17	37955555	A	G	0.0345	-0.0693	0.005441	0.0001	0.0004
rs145947882	17	41809207	C	A	0.0263	-0.0599	0.006329	-0.0001	0.0005
rs146346285	17	38897220	T	C	0.0142	0.0594	0.008911	-0.0011	0.0007
rs150497606	17	55466426	A	G	0.0494	-0.0454	0.004614	-0.0002	0.0004
rs180506	17	68274205	A	G	0.7763	-0.023	0.002385	-0.0002	0.0002
rs1838149	17	33819302	A	G	0.4218	-0.0165	0.002055	0.0002	0.0002
rs34210653	17	4535314	A	G	0.0207	-0.1756	0.006973	-0.0003	0.0006
rs3785496	17	56274133	G	A	0.237	0.0154	0.002332	0.0001	0.0002
rs397187	17	8784759	C	T	0.4297	-0.0123	0.002022	0.0001	0.0002
rs55868524	17	7170665	A	G	0.6059	0.0131	0.002031	0.0001	0.0002
rs62061733	17	44018399	G	A	0.2294	-0.0323	0.002364	0.0004	0.0002
rs62078383	17	47042908	C	A	0.4105	-0.0132	0.002029	0.0001	0.0002
rs62085020	17	74471960	A	G	0.3502	-0.0135	0.002099	-0.0001	0.0002

rs62086903	17	66016006	C	T	0.232	0.0304	0.00238	0.0003	0.0002
rs7215391	17	64232022	T	C	0.257	-0.0127	0.002272	-0.0002	0.0002
rs72911377	17	76381463	A	G	0.1484	-0.0161	0.002823	0.0001	0.0002
rs74480102	17	7742601	A	G	0.0428	-0.083	0.00494	0.0004	0.0004
rs8080418	17	26084986	T	C	0.2693	-0.0143	0.002248	-0.0002	0.0002
rs9889262	17	47398070	A	T	0.3641	0.0285	0.002067	-0.0001	0.0002
rs9900933	17	73801154	C	T	0.3443	-0.02	0.002093	0	0.0002
rs1395269	18	61377644	G	T	0.3012	-0.0236	0.002162	-0.0004	0.0002
rs17758695	18	60920854	T	C	0.0296	-0.1221	0.005948	0	0.0005
rs1788105	18	67532292	G	A	0.5276	-0.0157	0.001989	-0.0003	0.0002
rs2292759	18	12884343	G	A	0.6007	0.0155	0.002039	0.0001	0.0002
rs2850542	18	48403560	T	G	0.5563	-0.0132	0.00202	0.0002	0.0002
rs57633475	18	46469650	G	A	0.1217	-0.0247	0.003048	0.0003	0.0003
rs7227000	18	55704834	C	T	0.8869	-0.0172	0.003134	-0.0003	0.0003
rs73963711	18	60874413	T	C	0.2108	0.0221	0.002465	0.0002	0.0002
rs76639817	18	45343508	T	C	0.0137	0.0499	0.008537	-0.0011	0.0007
rs8083368	18	56219590	A	G	0.2301	0.0155	0.002364	0.0001	0.0002
rs954954	18	60902328	C	A	0.1049	-0.0352	0.00326	-0.0001	0.0003
rs9675999	18	20627691	A	G	0.6267	0.0164	0.002057	0.0001	0.0002
rs11084096	19	52128795	A	G	0.2961	-0.0199	0.002173	-0.0003	0.0002
rs118013485	19	33726577	A	G	0.067	-0.0336	0.004068	-0.0001	0.0003
rs12978850	19	17222753	T	C	0.1939	-0.0138	0.002512	0	0.0002
rs145605569	19	45742902	T	C	0.2595	-0.0312	0.002269	0	0.0002
rs1529745	19	19517325	G	C	0.1749	0.0156	0.002619	0	0.0002
rs350836	19	4080516	T	C	0.7099	-0.0172	0.002205	-0.0001	0.0002
rs36084354	19	1079959	A	G	0.0918	-0.0454	0.003441	0.0003	0.0003
rs3786586	19	16495287	G	A	0.1546	0.0319	0.00275	0	0.0002
rs3810115	19	53400760	A	C	0.4757	0.011	0.001998	0.0001	0.0002
rs410867	19	16427111	G	A	0.2167	-0.0594	0.00241	0	0.0002
rs412884	19	40219449	C	T	0.6722	0.0576	0.002109	0	0.0002
rs420458	19	5909780	C	T	0.6803	0.0117	0.00213	0	0.0002
rs45521740	19	2245622	A	G	0.0575	-0.0236	0.004289	-0.0006	0.0004
rs4802399	19	38899999	A	G	0.043	0.0433	0.004908	0.0002	0.0004
rs4804548	19	11041538	G	T	0.2943	0.014	0.002177	-0.0001	0.0002
rs62108948	19	52311527	C	A	0.1302	0.0263	0.002961	0.0001	0.0002
rs62111672	19	7415064	A	G	0.042	0.0405	0.005461	0.0002	0.0004
rs62117160	19	45232161	A	G	0.0453	-0.0476	0.00477	-0.0004	0.0004
rs72980722	19	2710962	A	G	0.205	-0.0137	0.002454	-0.0001	0.0002
rs76793172	19	46359794	T	C	0.0916	-0.0417	0.003436	0.0005	0.0003
rs8108623	19	18408519	A	C	0.6254	0.0178	0.002165	0	0.0002
rs1178016	20	2996497	T	C	0.4954	0.0113	0.001987	0.0001	0.0002
rs13045492	20	49134535	T	A	0.0839	-0.0327	0.003599	0.0001	0.0003
rs2253427	20	1552430	C	T	0.7781	-0.0138	0.002397	-0.0001	0.0002
rs3746420	20	50140627	C	G	0.0612	-0.0318	0.004163	-0.0004	0.0003
rs3790163	20	10647951	G	A	0.7924	-0.0153	0.002521	0.0004	0.0002
rs4811021	20	48907477	G	T	0.5741	0.0112	0.002009	0.0002	0.0002
rs4812447	20	39272620	G	A	0.4402	0.0128	0.002011	0	0.0002
rs6080761	20	17629162	A	G	0.4194	0.0185	0.002014	0.0001	0.0002
rs6103572	20	42657862	C	T	0.7291	-0.0241	0.002237	-0.0001	0.0002
rs6139104	20	390310	T	A	0.0925	-0.0194	0.003441	0.0001	0.0003
rs6141755	20	31163565	T	G	0.236	-0.0168	0.002348	-0.0001	0.0002
rs73322872	20	62694105	T	C	0.2419	0.0146	0.002327	0	0.0002
rs76171326	20	25194611	A	G	0.0155	-0.064	0.00834	0.001	0.0007
rs80054178	20	30294682	C	T	0.0224	-0.0818	0.006746	0.001	0.0006
rs11088236	21	34416187	T	C	0.453	0.0183	0.001998	0.0001	0.0002
rs114152720	21	36434894	A	G	0.0302	-0.0363	0.005897	0.0005	0.0005
rs11701475	21	16931347	C	T	0.2226	0.0149	0.002442	-0.0002	0.0002
rs12483051	21	43850713	G	C	0.1375	-0.0216	0.002903	0.0001	0.0002

rs2223043	21	16438793	G	A	0.307	0.0194	0.002295	-0.0001	0.0002
rs2838330	21	45023876	A	T	0.5538	0.0131	0.001995	0.0003	0.0002
rs28421324	21	36286938	T	A	0.1021	0.0358	0.003291	-0.0004	0.0003
rs2847224	21	45660835	A	C	0.4306	0.0249	0.002027	0.0001	0.0002
rs56117721	21	36398586	A	T	0.0716	-0.1044	0.003874	0.0004	0.0003
rs80109907	21	39850955	A	C	0.0094	0.081	0.010366	0.0017	0.0009
rs9979383	21	36715761	T	C	0.6298	0.0284	0.002067	0	0.0002
rs11702918	22	17594623	T	C	0.1026	-0.0223	0.00328	0.0001	0.0003
rs3218315	22	37529724	A	G	0.3618	-0.0207	0.002096	-0.0003	0.0002
rs34505104	22	24624609	G	A	0.3062	-0.0261	0.00216	0	0.0002
rs4253755	22	46615376	A	G	0.1284	-0.0289	0.002993	-0.0001	0.0002
rs5747308	22	18133500	C	A	0.5045	0.0156	0.001987	0.0001	0.0002
rs5753083	22	30744444	C	G	0.2789	0.0126	0.002214	0.0001	0.0002
rs60175411	22	37311858	A	G	0.1667	-0.0298	0.002681	-0.0001	0.0002
rs73176685	22	41781094	G	C	0.2429	0.0238	0.002321	0	0.0002
rs739427	22	31659101	C	G	0.5117	-0.0225	0.001985	0.0001	0.0002
rs743002	22	41404939	C	T	0.0656	-0.0589	0.00403	0.0003	0.0003
rs75107793	22	50628937	A	G	0.0723	0.0329	0.003882	0.0001	0.0003
