# Supplementary material for: Blood cell traits and risk of glaucoma: A two-sample mendelian randomization study
Source: Front Genet. 2023 Apr 12;14:1142773. doi: 10.3389/fgene.2023.1142773 (PMC10130872; doi:10.3389/fgene.2023.1142773)
Supplement: Supplementary file 1 [file DataSheet1.ZIP › eTable 1. Basophil cell count exposure SNPs and their association with glaucoma..pdf]

eTable 1. Basophil cell count exposure SNPs and their association with glaucoma.
Chr = chromosome; POS = position ; EA = effect allele; NEA = non-effect allele; EAF = effect allele frequency; SE = standard error.

SNP	Chr	POS	EA	NEA	EAF	Basophil Cell Count		Glaucoma	
						Beta	SE	Beta	SE
rs1086893	1	212563114	C	T	0.3432	0.0299	0.0022	0.0002	0.0002
rs10927074	1	236103965	C	T	0.8923	0.0792	0.0033	0.0003	0.0003
rs11121246	1	8917102	G	T	0.5022	0.0127	0.002	0.0001	0.0002
rs11204682	1	150595537	T	G	0.2229	-0.024	0.0025	0	0.0002
rs12075	1	159175354	A	G	0.5795	0.0278	0.0021	-0.0002	0.0002
rs12123922	1	205140436	A	G	0.4406	-0.0278	0.0021	0.0001	0.0002
rs12566174	1	29001893	C	T	0.422	0.0137	0.0021	-0.0001	0.0002
rs142405270	1	161353349	G	A	0.019	0.0507	0.0082	-0.0007	0.0006
rs1537061	1	87739219	C	T	0.0987	-0.0251	0.0034	0	0.0003
rs16856110	1	205631767	G	A	0.2296	0.0167	0.0024	0	0.0002
rs17613339	1	118139540	T	C	0.1284	-0.0168	0.0031	0.0004	0.0002
rs17625587	1	198990494	A	G	0.2636	0.015	0.0023	-0.0004	0.0002
rs3917932	1	36943916	G	C	0.577	-0.012	0.0021	0	0.0002
rs4060971	1	21506930	T	C	0.5511	-0.0207	0.0021	-0.0003	0.0002
rs56043070	1	247719769	A	G	0.0715	-0.026	0.004	0	0.0003
rs588485	1	20916444	A	G	0.3331	-0.0125	0.0022	-0.0002	0.0002
rs6671847	1	161478810	A	G	0.5082	0.0136	0.0021	0	0.0002
rs6701440	1	156249505	G	A	0.5049	0.0119	0.002	0.0004	0.0002
rs724781	1	153336018	G	C	0.2806	-0.0132	0.0023	-0.0001	0.0002
rs7534936	1	22341755	C	T	0.3288	0.0122	0.0021	0.0001	0.0002
rs80194822	1	28109632	G	A	0.0108	0.0608	0.0105	0.0002	0.0008
rs13386606	2	8440572	A	G	0.3581	-0.0119	0.0021	-0.0003	0.0002
rs13419763	2	219134950	T	C	0.5625	0.0162	0.0021	0	0.0002
rs1427499	2	145400317	G	A	0.7103	0.021	0.0023	-0.0003	0.0002
rs1598207	2	148757870	G	A	0.3027	0.0202	0.0022	-0.0003	0.0002
rs2028900	2	85767735	T	C	0.4493	-0.014	0.0021	-0.0002	0.0002
rs2860773	2	65665641	T	G	0.4077	0.0191	0.0021	0.0001	0.0002
rs4602187	2	181834018	T	C	0.6831	0.0141	0.0022	0	0.0002
rs62105478	2	8735869	A	G	0.0517	-0.0317	0.0047	-0.0009	0.0004
rs62160676	2	112167931	C	T	0.2961	-0.0166	0.0023	-0.0002	0.0002
rs6543144	2	103092575	G	A	0.3129	0.0137	0.0022	0.0003	0.0002
rs6734238	2	113841030	G	A	0.4023	0.0121	0.0021	-0.0001	0.0002
rs73987603	2	213844822	A	G	0.1664	0.024	0.0028	0.0001	0.0002
rs7573465	2	182315885	T	G	0.5561	0.0155	0.0021	0.0003	0.0002
rs77785849	2	146580195	A	C	0.0335	0.036	0.0058	0	0.0005
rs79140637	2	65084123	A	G	0.0542	-0.0275	0.0046	0.0003	0.0004
rs11710737	3	107464170	G	A	0.4389	0.0116	0.0021	-0.0001	0.0002
rs12497690	3	27795397	C	A	0.3713	0.0146	0.0021	0.0001	0.0002
rs13089722	3	128306757	A	G	0.1078	-0.088	0.0033	0.0004	0.0003
rs1669340	3	3198380	T	G	0.8392	-0.034	0.0028	-0.0004	0.0002
rs2089979	3	196501413	G	A	0.4157	-0.0139	0.0021	0	0.0002
rs3181077	3	46250652	T	C	0.7185	-0.0371	0.0023	0.0002	0.0002
rs34850939	3	47063910	A	G	0.349	0.0138	0.0022	0.0001	0.0002
rs4324460	3	3120542	G	T	0.2189	0.0242	0.0025	0.0005	0.0002
rs59107033	3	123105643	T	C	0.2248	-0.014	0.0025	0.0001	0.0002
rs6780544	3	187424334	A	G	0.6226	-0.0164	0.0021	0	0.0002
rs74535412	3	128297689	A	G	0.0346	0.0385	0.0057	0.0003	0.0005
rs7611275	3	128206759	G	C	0.0376	-0.03	0.0054	-0.0002	0.0004
rs7613595	3	16908518	C	A	0.2176	-0.0364	0.0025	-0.0001	0.0002
rs9819371	3	141206800	T	C	0.0646	-0.0301	0.0042	0	0.0003
rs10006833	4	9953097	C	T	0.2091	-0.0156	0.0025	-0.0002	0.0002
rs11097787	4	103407342	T	C	0.4026	-0.0145	0.0021	-0.0001	0.0002
rs11568994	4	110897535	A	G	0.3403	-0.0122	0.0022	-0.0004	0.0002

rs56406125	4	87940205	T	G	0.1901	-0.0349	0.0026	0.0001	0.0002
rs6814526	4	83534647	T	C	0.2214	-0.0184	0.0025	0.0001	0.0002
rs7684939	4	55509189	A	G	0.4887	-0.0119	0.002	0.0002	0.0002
rs7694971	4	79629350	G	T	0.6242	0.0182	0.0021	0.0002	0.0002
rs112352373	5	118687625	G	C	0.2032	-0.0139	0.0025	-0.0003	0.0002
rs11741255	5	131811182	A	G	0.4252	-0.0142	0.0021	-0.0004	0.0002
rs13188960	5	35853319	T	G	0.2795	-0.0191	0.0023	-0.0001	0.0002
rs2271352	5	126091428	C	G	0.2256	0.0423	0.0025	-0.0003	0.0002
rs2594836	5	173205318	A	G	0.7223	-0.0178	0.0023	-0.0001	0.0002
rs34500	5	98294885	A	G	0.084	-0.0263	0.0037	-0.0001	0.0003
rs357618	5	150846612	G	A	0.3881	-0.0138	0.0021	-0.0002	0.0002
rs3857286	5	54863190	T	C	0.6951	-0.0123	0.0022	-0.0002	0.0002
rs4912807	5	141519296	C	G	0.6175	0.0129	0.0021	-0.0005	0.0002
rs10806232	6	82679060	T	A	0.5978	-0.0147	0.0021	0.0001	0.0002
rs11756802	6	7170084	A	T	0.0482	-0.0294	0.0049	0.0002	0.0004
rs1205896	6	22341469	A	G	0.5038	0.0242	0.0021	-0.0001	0.0002
rs12212535	6	90808352	T	C	0.3911	-0.0145	0.0022	0	0.0002
rs13204572	6	26189356	C	G	0.1015	-0.0203	0.0034	-0.0005	0.0003
rs139719552	6	130354973	C	T	0.0138	0.0582	0.0093	0.0012	0.0007
rs2429642	6	31486962	A	G	0.163	0.0194	0.0028	0	0.0002
rs2524079	6	31242174	A	G	0.4191	0.0312	0.0021	0.0003	0.0002
rs35045014	6	107432157	A	C	0.4557	-0.0114	0.0021	-0.0001	0.0002
rs377763	6	32199144	A	C	0.2142	-0.0273	0.0025	-0.0004	0.0002
rs4715138	6	13181052	A	G	0.3158	0.0129	0.0022	0.0004	0.0002
rs6927569	6	109621494	C	T	0.523	0.0217	0.0021	0.0002	0.0002
rs915125	6	82463376	T	C	0.2811	-0.0293	0.0023	0.0001	0.0002
rs9274351	6	32632425	A	T	0.1966	0.0197	0.0028	-0.0003	0.0002
rs9376098	6	135499460	A	T	0.3492	0.0232	0.0021	-0.0002	0.0002
rs11768817	7	80223839	G	A	0.0643	0.0255	0.0042	0.0003	0.0003
rs11772895	7	143081942	C	G	0.2778	0.0341	0.0023	0.0004	0.0002
rs1186222	7	75247329	T	C	0.4619	-0.022	0.0021	-0.0002	0.0002
rs149007767	7	50370254	T	C	0.1622	0.0266	0.0029	-0.0001	0.0002
rs182090955	7	92239892	A	G	0.0098	-0.0877	0.0107	-0.0008	0.0009
rs2158799	7	28277107	G	C	0.6096	0.0141	0.0021	0.0001	0.0002
rs2282986	7	92299545	C	T	0.0181	-0.0887	0.0077	-0.0006	0.0006
rs3731332	7	92300568	T	C	0.0222	-0.104	0.0071	0.0005	0.0006
rs42030	7	92236421	A	G	0.0761	0.0302	0.0039	-0.0001	0.0003
rs56179563	7	129685597	A	G	0.3891	0.0146	0.0021	-0.0002	0.0002
rs56388170	7	28724374	T	G	0.293	0.0314	0.0023	0	0.0002
rs6975957	7	100329189	T	C	0.8142	-0.0154	0.0028	-0.0002	0.0002
rs73049252	7	8016602	A	G	0.0585	-0.0372	0.0044	0.0002	0.0003
rs10956401	8	129002419	A	G	0.3433	-0.0153	0.0022	-0.0001	0.0002
rs117182261	8	43508916	A	G	0.0264	-0.0472	0.0069	-0.0002	0.0005
rs13267723	8	130672344	A	G	0.2317	-0.014	0.0024	0.0003	0.0002
rs16923637	8	59567885	T	G	0.286	0.0142	0.0023	0	0.0002
rs2738104	8	6792257	A	G	0.6723	0.0184	0.0022	0.0001	0.0002
rs2977799	8	6696927	G	A	0.5092	0.0275	0.0021	-0.0001	0.0002
rs45577137	8	48651633	G	A	0.0452	-0.0453	0.0053	-0.0002	0.0004
rs4876400	8	119114110	G	A	0.6263	-0.0171	0.0021	0.0001	0.0002
rs55690609	8	6902043	A	G	0.3227	-0.0349	0.0022	0.0001	0.0002
rs56018450	8	38856336	G	A	0.411	0.0126	0.0021	0	0.0002
rs6557615	8	22940647	A	G	0.681	-0.0188	0.0022	-0.0001	0.0002
rs6993770	8	106581528	T	A	0.2871	-0.0182	0.0023	0.0001	0.0002
rs7819602	8	10726842	G	C	0.6129	0.0162	0.0021	-0.0001	0.0002
rs7832357	8	126516197	G	A	0.3423	-0.0154	0.0022	-0.0001	0.0002
rs9297295	8	101382999	T	C	0.3474	0.0118	0.0022	0	0.0002
rs12376511	9	22142756	C	T	0.1629	-0.0207	0.0028	0.0002	0.0002
rs1633768	9	135879138	T	C	0.2753	-0.0166	0.0023	0.0002	0.0002

rs2150052	9	113945067	T	A	0.5055	-0.0133	0.002	0.0003	0.0002
rs2273770	9	77755469	T	C	0.3987	0.0117	0.0021	-0.0002	0.0002
rs2417055	9	130532772	T	C	0.5694	-0.0117	0.0021	0.0001	0.0002
rs4503179	9	21950879	A	G	0.7235	0.0169	0.0023	0	0.0002
rs7044519	9	82233842	G	T	0.1614	0.0167	0.0028	0.0002	0.0002
rs10823305	10	70906916	A	G	0.8289	0.017	0.0027	-0.0001	0.0002
rs10883359	10	101274033	G	A	0.2846	-0.0131	0.0023	-0.0003	0.0002
rs10906375	10	13498371	G	A	0.301	-0.0125	0.0022	0.0002	0.0002
rs11591540	10	26734517	A	G	0.4005	0.0235	0.0021	-0.0002	0.0002
rs1539174	10	974870	G	C	0.2373	0.0464	0.0024	-0.0001	0.0002
rs2077218	10	96071561	A	G	0.7622	0.0159	0.0024	-0.0001	0.0002
rs2998286	10	28780373	C	T	0.7698	0.0209	0.0024	-0.0001	0.0002
rs3071	10	102114463	C	A	0.2983	-0.0164	0.0022	-0.0001	0.0002
rs34377578	10	104336426	C	A	0.2518	0.0134	0.0024	0	0.0002
rs3781452	10	126355129	T	C	0.6321	0.0198	0.0021	-0.0004	0.0002
rs7078507	10	77140864	G	A	0.6092	-0.0161	0.0021	-0.0002	0.0002
rs72786903	10	50376394	T	C	0.2224	-0.0139	0.0025	-0.0004	0.0002
rs748113	10	73508791	C	T	0.4361	-0.0117	0.0021	0	0.0002
rs10734121	11	89656239	A	G	0.8453	-0.0254	0.0028	-0.0004	0.0002
rs10835333	11	3957766	G	A	0.3492	0.0146	0.0022	0	0.0002
rs10893844	11	128185850	C	G	0.5014	-0.0155	0.002	0.0003	0.0002
rs10896064	11	65641033	C	G	0.5325	0.0133	0.0021	0.0001	0.0002
rs149709671	11	74382388	A	G	0.0344	0.0316	0.0058	0.0003	0.0005
rs2568054	11	8876029	G	A	0.5393	0.0116	0.0021	-0.0003	0.0002
rs2606724	11	113957880	A	G	0.453	0.0157	0.0021	0.0003	0.0002
rs6421984	11	305619	C	T	0.5141	0.0184	0.0021	-0.0001	0.0002
rs695113	11	128562098	T	C	0.6963	0.017	0.0022	0.0003	0.0002
rs734095	11	2323198	G	C	0.1133	-0.0276	0.0032	-0.0003	0.0003
rs742631	11	32077472	T	C	0.2837	-0.0166	0.0023	-0.0001	0.0002
rs74472890	11	72946279	C	T	0.0494	0.1026	0.0047	0.0003	0.0004
rs10746147	12	80316758	G	A	0.9262	-0.0224	0.0039	-0.0001	0.0003
rs11064881	12	120146925	A	G	0.0735	-0.0323	0.0039	-0.0003	0.0003
rs146970669	12	27103449	A	G	0.0941	0.0224	0.0035	-0.0002	0.0003
rs17860282	12	51740350	T	C	0.0112	0.1047	0.0099	0.0024	0.0008
rs2118140	12	66698461	G	A	0.5284	0.0183	0.0021	-0.0001	0.0002
rs2286599	12	6499533	A	G	0.1417	0.0232	0.003	-0.0001	0.0002
rs2952110	12	106661316	G	A	0.1689	0.0158	0.0028	-0.0002	0.0002
rs3184504	12	111884608	C	T	0.5186	-0.0282	0.0021	0.0002	0.0002
rs4475963	12	129302255	G	T	0.3702	-0.0135	0.0021	-0.0002	0.0002
rs4763817	12	12481296	T	C	0.7386	0.0135	0.0024	0	0.0002
rs75084335	12	76981753	G	C	0.0692	0.0284	0.0041	0.0005	0.0003
rs3892360	13	41184078	G	C	0.3066	0.0431	0.0059	0	0.0002
rs4941839	13	37025916	A	G	0.7141	-0.0134	0.0023	-0.0001	0.0002
rs76428106	13	28604007	C	T	0.0134	0.0819	0.0094	-0.0006	0.0007
rs11158159	14	57857162	C	G	0.7698	0.0282	0.0026	0	0.0002
rs2239635	14	23588731	C	G	0.7043	0.0344	0.0023	0	0.0002
rs2289511	14	88454910	A	G	0.5823	-0.0164	0.0021	0	0.0002
rs7150069	14	35279515	A	G	0.3621	0.0117	0.0021	0.0001	0.0002
rs72697295	14	93069980	C	G	0.1809	-0.0175	0.0027	-0.0001	0.0002
rs72721631	14	75807762	A	C	0.2371	-0.0193	0.0024	0	0.0002
rs12443468	15	81888088	G	A	0.2259	0.0145	0.0025	0	0.0002
rs2070596	15	50545159	A	T	0.2114	0.0227	0.0025	-0.0001	0.0002
rs2074585	15	91009484	A	G	0.5149	-0.0309	0.0021	0	0.0002
rs2415042	15	69649385	G	C	0.6272	0.0129	0.0021	-0.0002	0.0002
rs28364390	15	42677173	A	T	0.1321	0.0203	0.003	0.0003	0.0002
rs62021606	15	64548734	G	T	0.0506	-0.0343	0.0047	-0.0003	0.0004
rs67175901	15	101748227	T	C	0.1082	0.0261	0.0034	-0.0001	0.0003
rs7496362	15	65758874	G	C	0.3658	-0.0127	0.0021	0	0.0002

rs12447180	16	88517722	C	T	0.3236	0.0235	0.0022	0.0001	0.0002
rs12921873	16	87933057	T	C	0.2109	0.0158	0.0025	0.0002	0.0002
rs12927351	16	74596618	A	C	0.1982	0.0163	0.0026	0.0002	0.0002
rs247833	16	84581684	A	G	0.2489	0.0162	0.0024	-0.0003	0.0002
rs7196129	16	30471109	C	T	0.5294	0.0168	0.0021	-0.0001	0.0002
rs875740	16	16123048	A	C	0.6653	-0.0179	0.0022	-0.0001	0.0002
rs9928015	16	57570561	T	G	0.302	-0.015	0.0022	0.0002	0.0002
rs12453682	17	37770005	T	C	0.6953	-0.0183	0.0022	0	0.0002
rs12601412	17	46208793	T	A	0.2471	0.0131	0.0024	0	0.0002
rs12941811	17	38159335	C	T	0.5782	-0.0464	0.0021	0.0002	0.0002
rs1295927	17	57929535	G	A	0.4478	-0.025	0.0021	0	0.0002
rs34097845	17	56413635	T	C	0.0567	0.0433	0.0046	-0.0001	0.0003
rs7420	17	72765215	T	C	0.5377	-0.0132	0.0021	0.0002	0.0002
rs7503461	17	2883320	T	C	0.3185	-0.0134	0.0022	0.0001	0.0002
rs8178414	17	56345363	T	C	0.0129	0.0739	0.0091	-0.0013	0.0007
rs17758695	18	60920854	T	C	0.0296	-0.1247	0.0061	0	0.0005
rs2959356	18	23590203	G	A	0.2981	0.014	0.0022	-0.0001	0.0002
rs508218	18	51779439	A	G	0.6912	0.0128	0.0022	0.0002	0.0002
rs561102	18	9851388	T	C	0.6549	-0.0141	0.0022	0.0001	0.0002
rs11666033	19	18334805	T	C	0.2891	-0.0132	0.0023	0.0002	0.0002
rs118013485	19	33726577	A	G	0.067	0.0237	0.0042	-0.0001	0.0003
rs12459419	19	51728477	T	C	0.323	-0.0167	0.0022	0.0003	0.0002
rs143273199	19	45744552	C	T	0.26	-0.0188	0.0023	0	0.0002
rs2607278	19	16568197	T	C	0.3061	0.0227	0.0022	0	0.0002
rs2967595	19	8566299	T	C	0.1679	-0.026	0.0028	-0.0003	0.0002
rs34158728	19	38903032	A	G	0.0283	0.0948	0.0062	0.0002	0.0005
rs4760	19	44153100	G	A	0.1556	-0.0195	0.0028	-0.0002	0.0002
rs62111672	19	7415064	A	G	0.0419	0.0349	0.0056	0.0002	0.0004
rs7250849	19	52158316	T	G	0.1072	0.0215	0.0033	0.0005	0.0003
rs73022294	19	33772811	T	C	0.0109	-0.0572	0.0101	-0.0011	0.0008
rs75176215	19	38917533	T	C	0.009	0.1117	0.011	-0.0009	0.0009
rs76427287	19	837190	C	T	0.254	-0.0288	0.0025	0.0002	0.0002
rs778798	19	5839613	C	A	0.7328	-0.0203	0.0023	0	0.0002
rs78744187	19	33754548	T	C	0.0817	-0.1302	0.0038	-0.0004	0.0003
rs8113682	19	19743730	G	T	0.7474	-0.0216	0.0024	0.0001	0.0002
rs1473698	20	8629807	T	C	0.401	-0.0124	0.0021	0	0.0002
rs16989483	20	43538597	C	T	0.2741	-0.0211	0.0023	0	0.0002
rs310631	20	62196253	A	C	0.4119	0.0123	0.0021	0.0001	0.0002
rs4812447	20	39272620	G	A	0.4402	0.0134	0.0021	0	0.0002
rs4911102	20	31179500	T	C	0.2343	-0.0217	0.0024	-0.0001	0.0002
rs6045612	20	1931001	T	C	0.2638	-0.0167	0.0023	-0.0002	0.0002
rs6091176	20	49150510	T	C	0.2611	0.0137	0.0023	-0.0001	0.0002
rs6141781	20	31263342	T	C	0.1471	0.0325	0.0029	-0.0001	0.0002
rs73110111	20	31267270	G	A	0.0268	-0.0374	0.0067	0.0001	0.0005
rs138595256	21	36789420	G	C	0.0256	0.0642	0.0069	0.0008	0.0006
rs2836241	21	39621213	C	T	0.4048	0.0139	0.0021	-0.0001	0.0002
rs34288539	22	24642009	T	C	0.2739	-0.0152	0.0023	0	0.0002
rs34780507	22	41405753	G	A	0.0657	0.0351	0.0042	0.0003	0.0003
rs5750482	22	38117943	C	T	0.6283	-0.0136	0.0021	-0.0003	0.0002
rs5766582	22	45632528	T	C	0.4747	-0.0169	0.0021	-0.0002	0.0002
rs7285377	22	19987202	T	G	0.2842	0.0145	0.0023	-0.0001	0.0002
