# Supplementary material for: Blood cell traits and risk of glaucoma: A two-sample mendelian randomization study
Source: Front Genet. 2023 Apr 12;14:1142773. doi: 10.3389/fgene.2023.1142773 (PMC10130872; doi:10.3389/fgene.2023.1142773)
Supplement: Supplementary file 1 [file DataSheet1.ZIP › eFigure5. Forrest plot of the causal effects of basophil cell count on glaucoma..pdf.pdf]

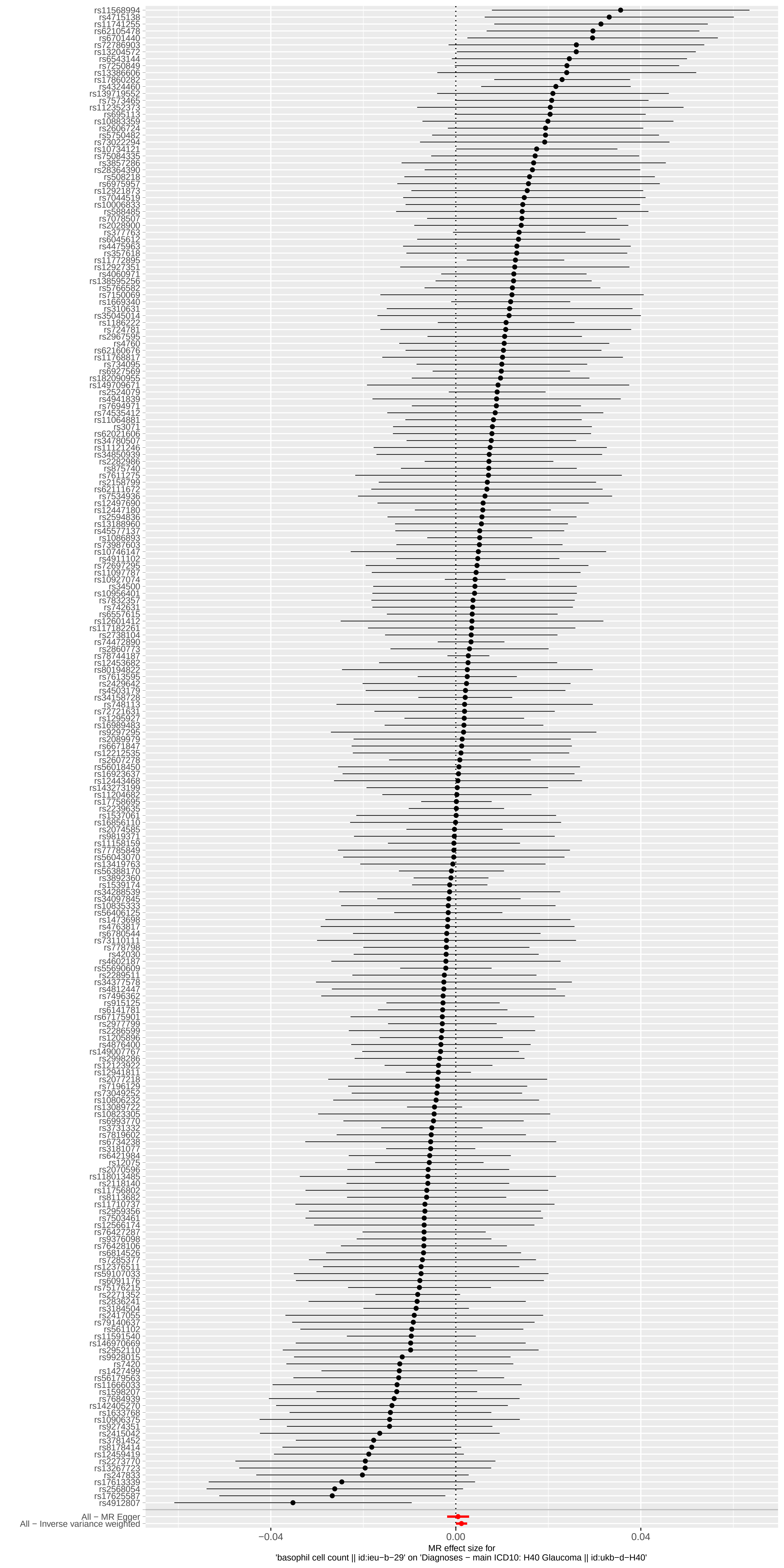
rs11568994
rs4715138
rs11741255
rs62105478
rs6701440
rs72786903
rs13204572
rs6543144
rs7250849
rs13386606
rs17860282
rs4324460
rs139719552
rs7573465
rs112352373
rs695113
rs10883359
rs2606724
rs5750482
rs73022294
rs10734121
rs75084335
rs3857286
rs28364390
rs508218
rs6975957
rs12921873
rs7044519
rs10006833
rs588485
rs7078507
rs2028900
rs377763
rs6045612
rs4475963
rs357618
rs11772895
rs12927351
rs4060971
rs138595256
rs5766582
rs7150069
rs1669340
rs310631
rs35045014
rs1186222
rs724781
rs2967595
rs4760
rs62160676
rs11768817
rs734095
rs6927569
rs182090955
rs149709671
rs2524079
rs4941839
rs7694971
rs74535412
rs11064881
rs3071
rs62021606
rs34780507
rs11121246
rs34850939
rs2282986
rs875740
rs7611275
rs2158799
rs62111672
rs7534936
rs12497690
rs12447180
rs2594836
rs13188960
rs45577137
rs1086893
rs73987603
rs10746147
rs4911102
rs72697295
rs11097787
rs10927074
rs34500
rs10956401
rs7832357
rs742631
rs6557615
rs12601412
rs117182261
rs2738104
rs74472890
rs2860773
rs78744187
rs12453682
rs80194822
rs7613595
rs2429642
rs4503179
rs34158728
rs748113
rs72721631
rs1295927
rs16989483
rs9297295
rs2089979
rs6671847
rs12212535
rs2607278
rs56018450
rs16923637
rs12443468
rs143273199
rs11204682
rs17758695
rs2239635
rs1537061
rs16856110
rs2074585
rs9819371
rs11158159
rs77785849
rs56043070
rs13419763
rs56388170
rs3892360
rs1539174
rs34288539
rs34097845
rs10835333
rs56406125
rs1473698
rs4763817
rs6780544
rs73110111
rs778798
rs42030
rs4602187
rs55690609
rs2289511
rs34377578
rs4812447
rs7496362
rs915125
rs6141781
rs67175901
rs2977799
rs2286599
rs1205896
rs4876400
rs149007767
rs2998286
rs12123922
rs12941811
rs2077218
rs7196129
rs73049252
rs10806232
rs13089722
rs10823305
rs6993770
rs3731332
rs7819602
rs6734238
rs3181077
rs6421984
rs12075
rs2070596
rs118013485
rs2118140
rs11756802
rs8113682
rs11710737
rs2959356
rs7503461
rs12566174
rs76427287
rs9376098
rs76428106
rs6814526
rs7285377
rs12376511
rs59107033
rs6091176
rs75176215
rs2271352
rs2836241
rs3184504
rs2417055
rs79140637
rs561102
rs11591540
rs146970669
rs2952110
rs9928015
rs7420
rs1427499
rs56179563
rs11666033
rs1598207
rs7684939
rs142405270
rs1633768
rs10906375
rs9274351
rs2415042
rs3781452
rs8178414
rs12459419
rs2273770
rs13267723
rs247833
rs17613339
rs2568054
rs17625587
rs4912807
All − MR Egger
All − Inverse variance weighted
−0.04
0.00
0.04
MR effect size for
'basophil cell count || id:ieu−b−29' on 'Diagnoses − main ICD10: H40 Glaucoma || id:ukb−d−H40'
