# Supplementary material for: Blood cell traits and risk of glaucoma: A two-sample mendelian randomization study
Source: Front Genet. 2023 Apr 12;14:1142773. doi: 10.3389/fgene.2023.1142773 (PMC10130872; doi:10.3389/fgene.2023.1142773)
Supplement: Supplementary file 1 [file DataSheet1.ZIP › eFigure7. Forrest plot of the causal effects of plateletcrit on glaucoma..pdf.pdf]

rs13274496
rs73135275
rs12458093
rs34121753
rs4965426
rs2071412
rs2239897
rs587080
rs68116612
rs2070724
rs200713481
rs147635058
rs72654647
rs34912569
rs467317
rs7860776
rs6712091
rs11190133
rs10940473
rs1716182
rs2288419
rs78691875
rs2298993
rs6919440
rs73000929
rs56414520
rs60485593
rs58530613
rs76171326
rs75967349
rs210798
rs1354034
rs6472235
rs4272720
rs1826164
rs6897795
rs945631
rs3135109
rs4709819
rs16858720
rs2894802
rs6556471
rs11071720
rs231353
rs80054178
rs116234817
rs6667939
rs2180369
rs71512947
rs11614212
rs7685731
rs6060976
rs6926219
rs11158588
rs4773860
rs11684299
rs55707100
rs645901
rs12040949
rs7950696
rs2034309
rs725860
rs4420638
rs62027291
rs714543
rs4961345
rs112560164
rs1538970
rs4808579
rs1019068
rs11553699
rs7253820
rs332507
rs150568286
rs8012145
rs4388979
rs10893909
rs75139539
rs9809116
rs218265
rs11841319
rs59865663
rs4686388
rs2836441
rs156355
rs10514301
rs12526480
rs188761458
rs2277339
rs11604127
rs55794721
rs7503168
rs11681227
rs12462978
rs78565404
rs382819
rs114694170
rs73735248
rs113542380
rs4814779
rs3132520
rs855791
rs141721165
rs9605049
rs78719031
rs385893
rs6878680
rs3844535
rs4937333
rs79389047
rs62259323
rs75107793
rs174594
rs11856829
rs9399136
rs2979489
rs7538358
rs2089979
rs11082383
rs12052715
rs113599586
rs4432538
rs2901381
rs1555405
rs17758695
rs182050989
rs34524896
rs1111890
rs487358
rs58434384
rs56043070
rs7705526
rs12943566
rs12884793
rs210142
rs865483
rs10048745
rs1260326
rs56084922
rs2932536
rs45535039
rs12669378
rs564726571
rs296852
rs41785
rs2243423
rs374039502
rs4783187
rs9376060
rs6475611
rs12459847
rs1569419
rs12223381
rs6993770
rs36125859
rs55983424
rs655029
rs79755767
rs11187838
rs532861
rs16979901
rs76840964
rs4587410
rs648103
rs12145626
rs7954567
rs1801689
rs2113814
rs9923283
rs738408
rs11723550
rs13144869
rs10908505
rs11704518
rs1968382
rs11072748
rs34922454
rs10769960
rs17758236
rs2526377
rs7535217
rs3758253
rs62160676
rs7567043
rs2724564
rs114982150
rs1182196
rs11082304
rs35150201
rs4334315
rs9697210
rs1758186
rs457648
rs6696074
rs2518721
rs8050260
rs929579
rs4947490
rs6923682
rs11794772
rs1346272
rs1124980
rs9572786
rs78265569
rs2255531
rs60786079
rs28636836
rs12946564
rs7427439
rs12451471
rs715
rs1539019
rs116052829
rs6860138
rs7034359
rs2748424
rs10004764
rs61025394
rs10116352
rs12266014
rs3865444
rs113825134
rs78399616
rs11235688
rs75612655
rs11617297
rs3804749
rs7829132
All − MR Egger
All − Inverse variance weighted
−0.02
0.00
0.02
MR effect size for
'Plateletcrit || id:ebi−a−GCST004607' on 'Diagnoses − main ICD10: H40 Glaucoma || id:ukb−d−H40'
