# Supplementary material for: Blood cell traits and risk of glaucoma: A two-sample mendelian randomization study
Source: Front Genet. 2023 Apr 12;14:1142773. doi: 10.3389/fgene.2023.1142773 (PMC10130872; doi:10.3389/fgene.2023.1142773)
Supplement: Supplementary file 1 [file DataSheet1.ZIP › eFigure8. Forrest plot of the causal effects of platelet count on glaucoma..pdf.pdf]

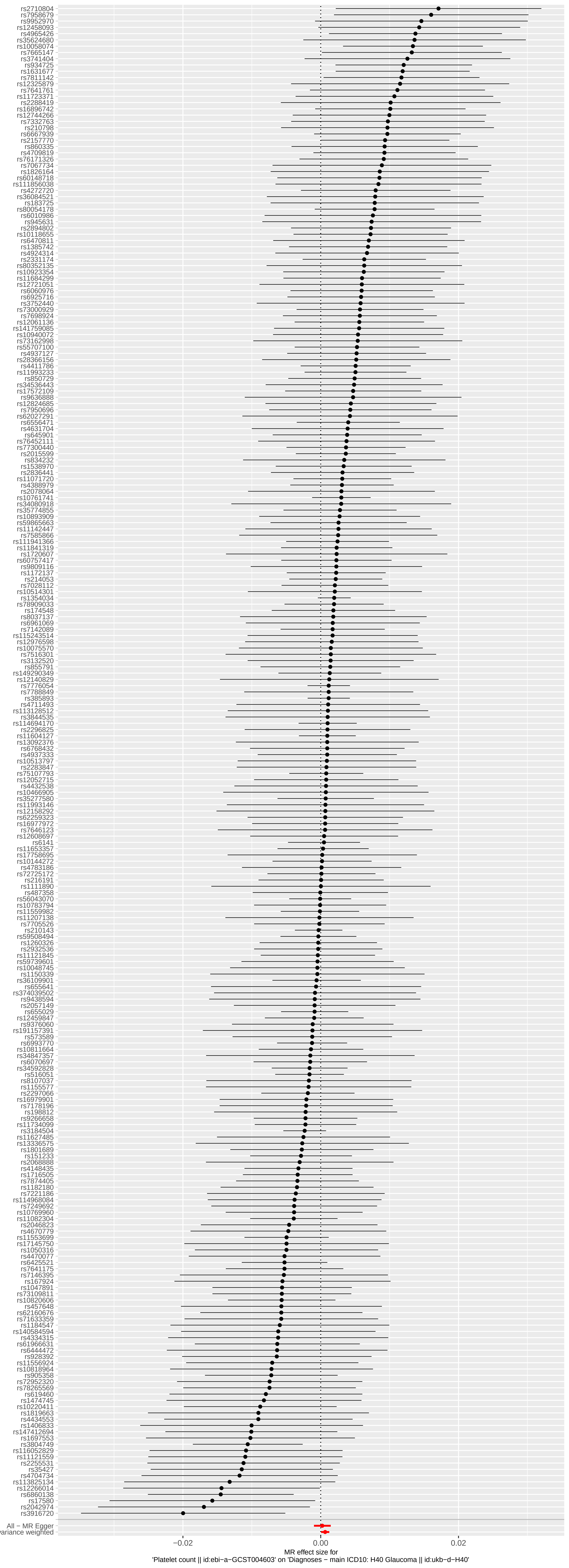
rs2710804
rs7958679
rs9952970
rs12458093
rs4965426
rs35624680
rs10058074
rs7665147
rs3741404
rs934725
rs1631677
rs7811142
rs12325879
rs7641761
rs11723371
rs2288419
rs16896742
rs12744266
rs7332763
rs210798
rs6667939
rs2157770
rs860335
rs4709819
rs76171326
rs7067734
rs1826164
rs60148718
rs111856038
rs4272720
rs36084521
rs183725
rs80054178
rs6010986
rs945631
rs2894802
rs10118655
rs6470811
rs1385742
rs4924314
rs2331174
rs80352135
rs10923354
rs11684299
rs12721051
rs6060976
rs6925716
rs3752440
rs73000929
rs7698924
rs12061136
rs141759085
rs10940072
rs73162998
rs55707100
rs4937127
rs28366156
rs4411786
rs11993233
rs850729
rs34536443
rs17572109
rs9636888
rs12824685
rs7950696
rs62027291
rs6556471
rs4631704
rs645901
rs76452111
rs77300440
rs2015599
rs834232
rs1538970
rs2836441
rs11071720
rs4388979
rs2078064
rs10761741
rs34080918
rs35774855
rs10893909
rs59865663
rs11142447
rs7585866
rs111941366
rs11841319
rs1720607
rs60757417
rs9809116
rs1172137
rs214053
rs7028112
rs10514301
rs1354034
rs78909033
rs174548
rs8037137
rs6961069
rs7142089
rs115243514
rs12976598
rs10075570
rs7516301
rs3132520
rs855791
rs149290349
rs12140829
rs7776054
rs7788849
rs385893
rs4711493
rs113128512
rs3844535
rs114694170
rs2296825
rs11604127
rs13092376
rs6768432
rs4937333
rs10513797
rs2283847
rs75107793
rs12052715
rs4432538
rs10466905
rs35277580
rs11993146
rs12158292
rs62259323
rs16977972
rs7646123
rs12608697
rs6141
rs11653357
rs17758695
rs10144272
rs4783186
rs72725172
rs216191
rs1111890
rs487358
rs56043070
rs10783794
rs11559982
rs11207138
rs7705526
rs210143
rs59508494
rs1260326
rs2932536
rs11121845
rs59739601
rs10048745
rs1150339
rs36109901
rs655641
rs374039502
rs9438594
rs2057149
rs655029
rs12459847
rs9376060
rs191157391
rs573589
rs6993770
rs10811664
rs34847357
rs6070697
rs34592828
rs516051
rs8107037
rs1155577
rs2297066
rs16979901
rs7178196
rs198812
rs9266658
rs11734099
rs3184504
rs11627485
rs13336575
rs1801689
rs151233
rs2068888
rs4148435
rs1716505
rs7874405
rs1182180
rs7221186
rs114968084
rs7249692
rs10769960
rs11082304
rs2046823
rs4670779
rs11553699
rs17145750
rs1050316
rs4470077
rs6425521
rs7641175
rs7146395
rs167924
rs1047891
rs73109811
rs10820606
rs457648
rs62160676
rs71633359
rs1184547
rs140584594
rs4334315
rs61966631
rs6444472
rs928392
rs11556924
rs10818964
rs905358
rs72952320
rs78265569
rs619460
rs1474745
rs10220411
rs1819663
rs4434553
rs1406833
rs147412694
rs1697553
rs3804749
rs116052829
rs11121559
rs2255531
rs35427
rs4704734
rs113825134
rs12266014
rs6860138
rs17580
rs2042974
rs3916720
All − MR Egger
All − Inverse variance weighted
−0.02
0.00
0.02
MR effect size for
'Platelet count || id:ebi−a−GCST004603' on 'Diagnoses − main ICD10: H40 Glaucoma || id:ukb−d−H40'
