# Supplementary figures and images for: Blood cell traits and risk of glaucoma: A two-sample mendelian randomization study
Source: Front Genet. 2023 Apr 12;14:1142773. doi: 10.3389/fgene.2023.1142773 (PMC10130872; doi:10.3389/fgene.2023.1142773)

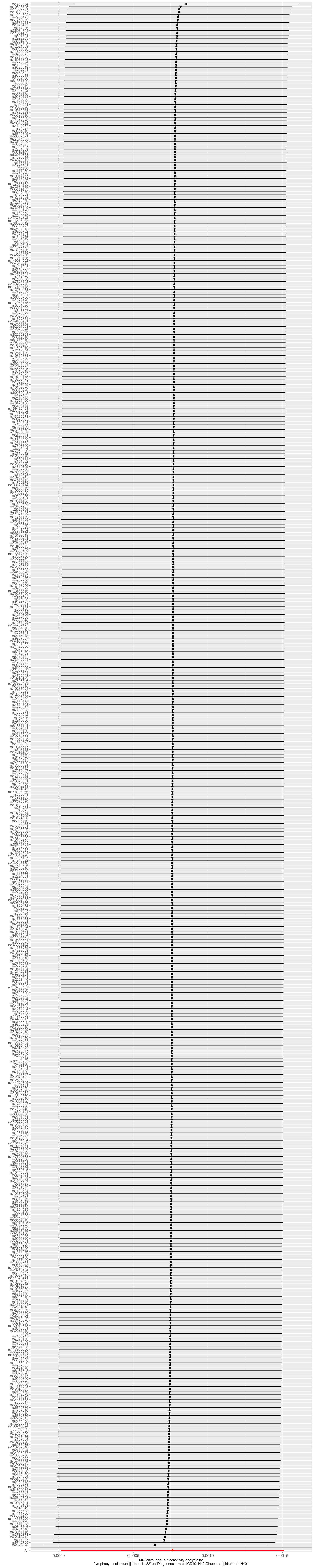

Supplement: Supplementary file 1 [file DataSheet1.ZIP › eFigure2. Leave-one-out permutation analysis plots for lymphocyte cell count on glaucoma..pdf.pdf]

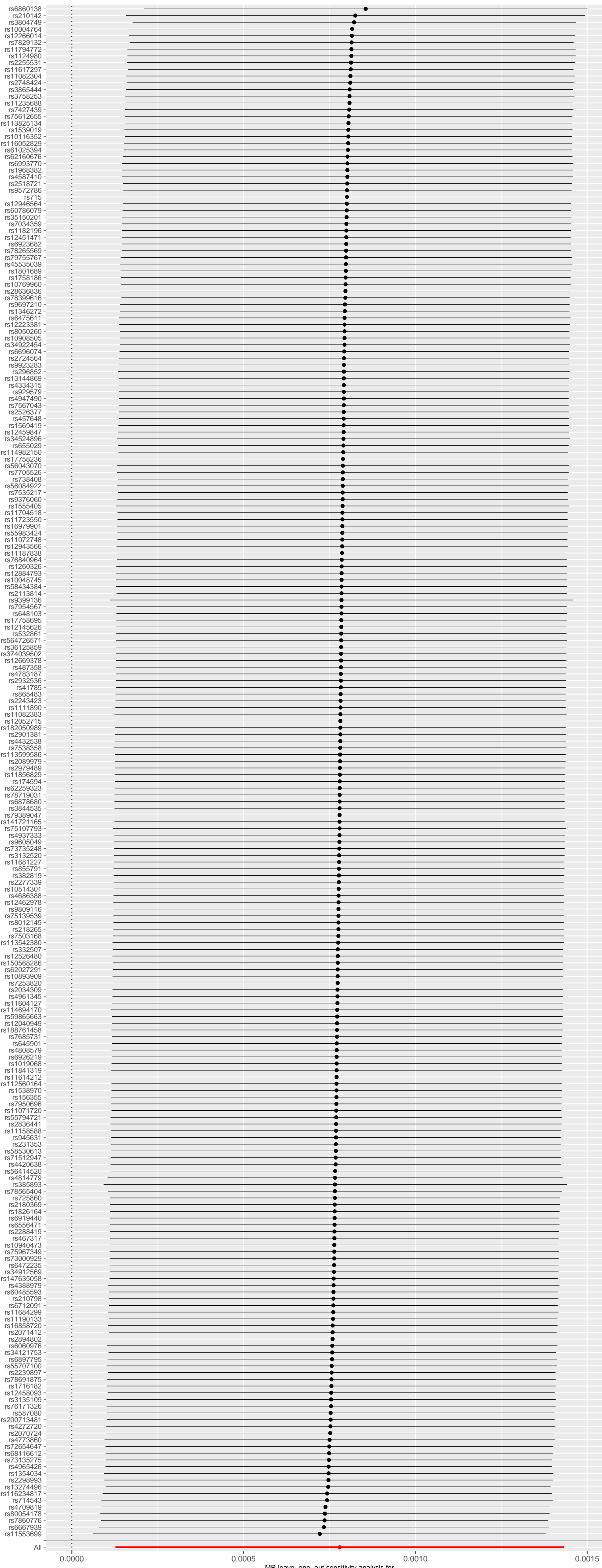

Supplement: Supplementary file 1 [file DataSheet1.ZIP › eFigure3. Leave-one-out permutation analysis plots for plateletcrit on glaucoma..pdf.pdf]

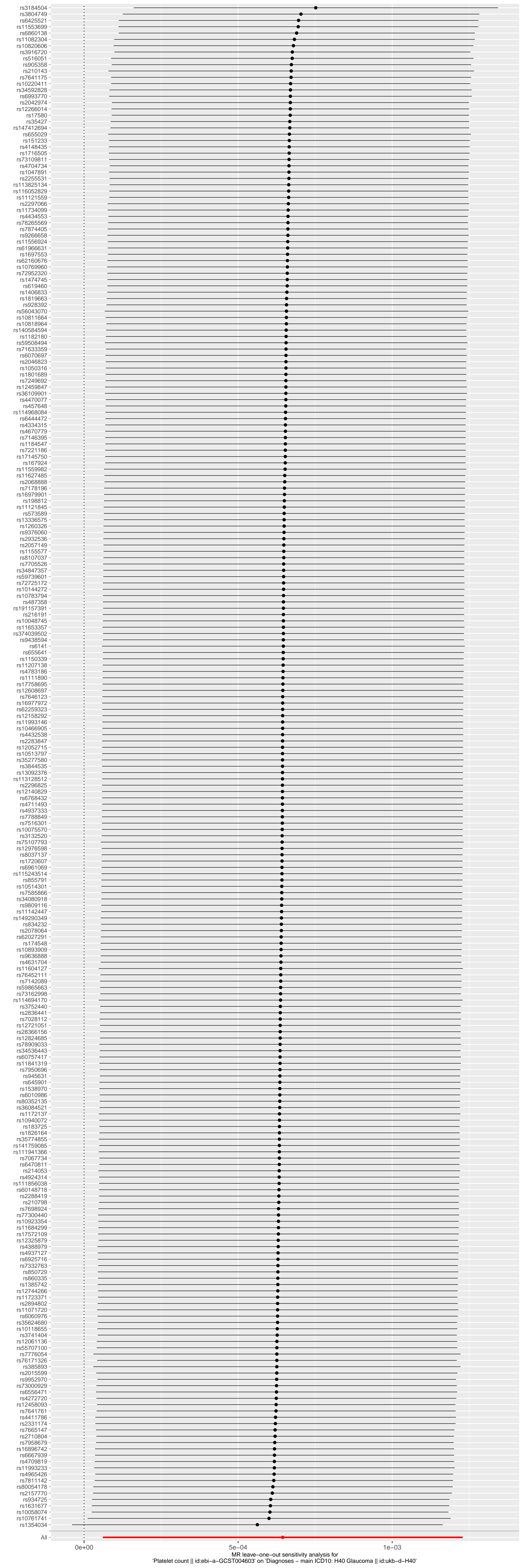

Supplement: Supplementary file 1 [file DataSheet1.ZIP › eFigure4. Leave-one-out permutation analysis plots for platelet count on glaucoma..pdf.pdf]

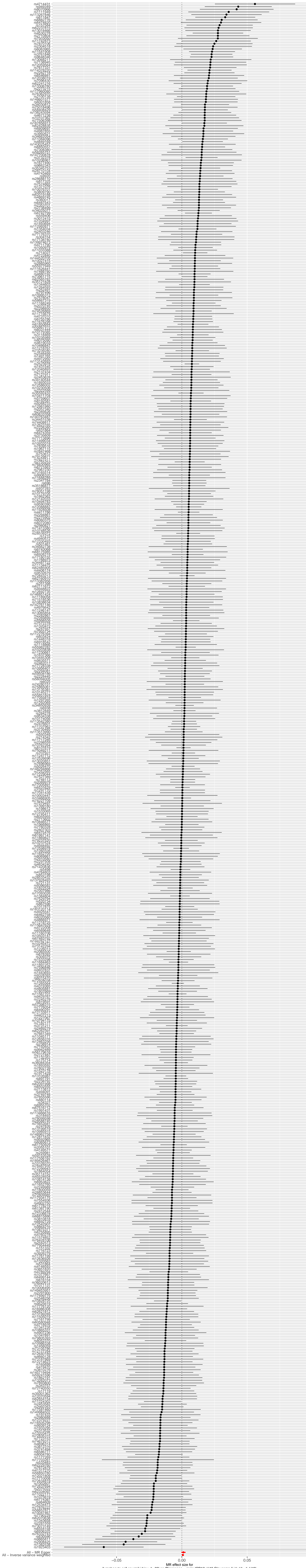

Supplement: Supplementary file 1 [file DataSheet1.ZIP › eFigure6. Forrest plot of the causal effects of lymphocyte cell count on glaucoma..pdf.pdf]

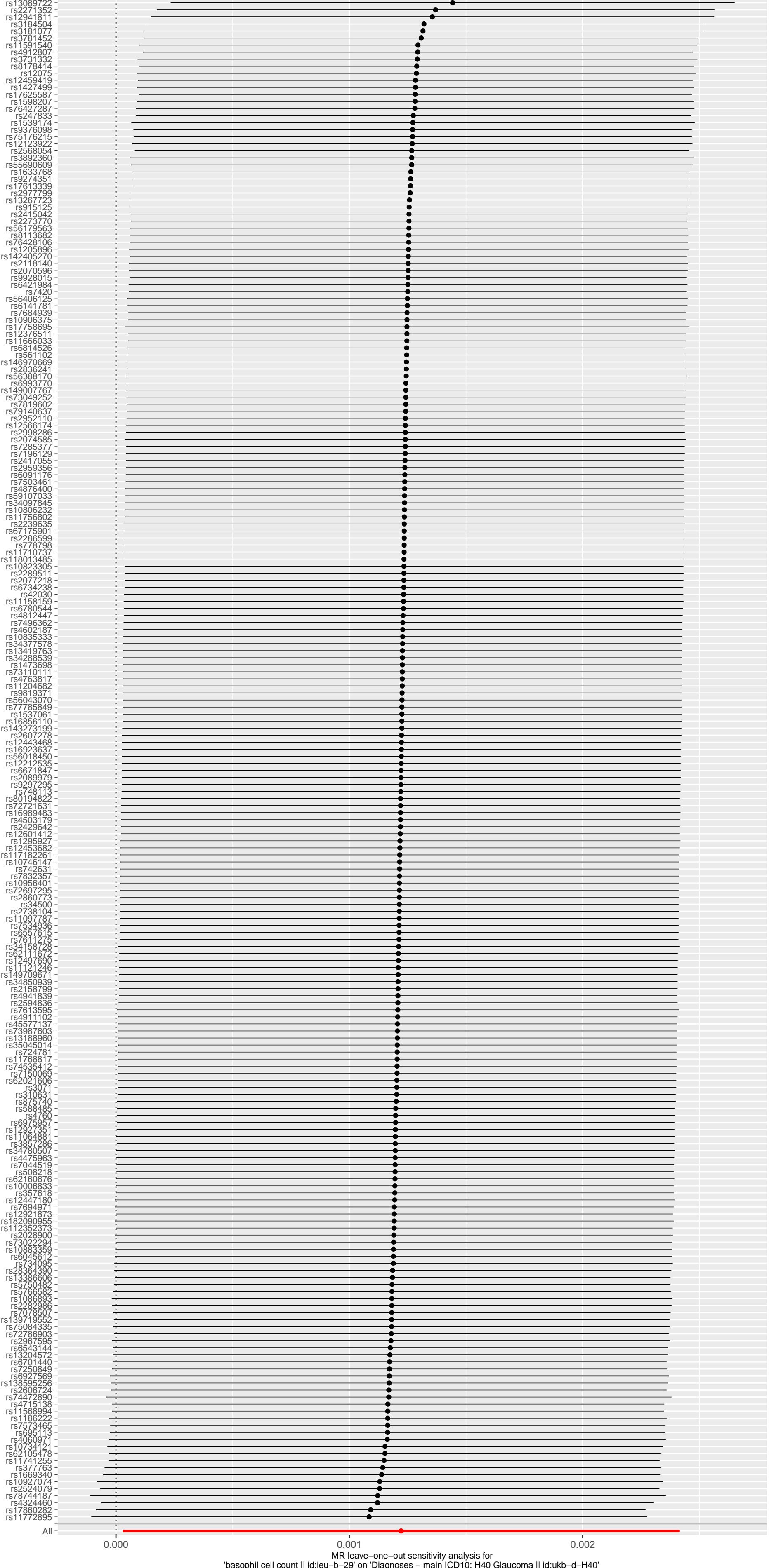

Supplement: Supplementary file 1 [file DataSheet1.ZIP › eFigure1. Leave-one-out permutation analysis plots for basophil cell count on glaucoma..pdf.pdf]
